# Stereodefined Synthesis of 3‑Difluoromethyl-Benzoxaboroles: Novel Antimicrobials with Unlocked H‑Bonding

**DOI:** 10.1021/acs.orglett.5c04602

**Published:** 2026-01-22

**Authors:** Alessandro Dimasi, Arianna Montoli, Claudio Lutti, Andrea Citarella, Paolo Ronchi, Francesco Castagnini, Valentina Mileo, Giovanni Macetti, Valerio Baldelli, Elio Rossi, Paolo Landini, Daniele Passarella, Valerio Fasano

**Affiliations:** † Università degli Studi di Milano, Via Camillo Golgi, 19, 20133 Milano, Italy; ‡ Chiesi Farmaceutici S.p.A., Largo Francesco Belloli 11/a, 43122 Parma, Italy; § Università di Parma, Parco Area delle Scienze 27/A, 43124 Parma, Italy

## Abstract

Benzoxaboroles, prominent scaffolds in medicinal chemistry, are typically modified on the benzene ring. In contrast, functionalization of the oxaborol ring is less common and often challenging. Indeed, 3-hydroxy-benzoxaboroles are virtually impossible to isolate due to their tautomeric equilibrium with the carbonyl form. In this work, we introduce a novel class of stereodefined 3-difluoromethyl-benzoxaboroles. The replacement of the hydroxy group with −CHF_2_ preserves stability while promoting bioactivity, owing to the lipophilic H-bond donor properties of the latter.

## Graphical abstract

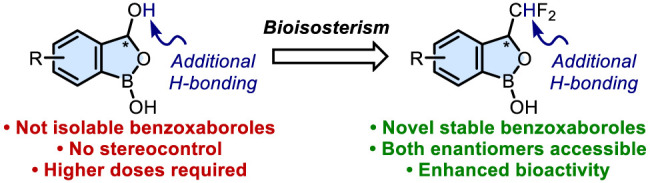

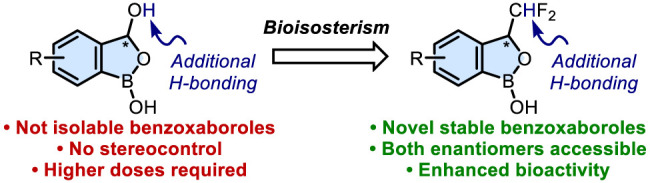

In the past decade, benzoxaboroles, cyclic hemiesters of phenylboronic acids, have emerged as important scaffolds in the development of boron-containing drugs.^1–4^ These compounds are particularly valuable due to their ability to bind diols such as ribose and glucose, thus finding applications in protein glycosylation or as sugar sensors or RNA binders.^5–7^ Additionally, benzoxaboroles can mimic phosphates by chelating metal ions in enzyme active sites, as elucidated for Toxoplasma CPSF3 (TgCPSF3), Autotaxin (ATX), and phosphodiesterase-4 (PDE4).^8–11^ Indeed, they can form boron-ate complexes, where the central boron adopts a tetrahedral geometry similar to that of phosphates. The pyramidalization process (shifting from 120° in planar B-sp^2^ to 109° in tetrahedral B-sp^3^) is favored in benzoxaboroles due to the strain release in the oxaborole ring. In fact, benzoxaboroles exhibit significantly higher acidity than phenylboronic acids, as demonstrated by the p*K*_a_ values of their corresponding water adducts (7.4 and 8.7, respectively).^12^ This enhanced acidity allows benzoxaboroles to predominantly exist in their anionic (boron-ate) forms in aqueous solutions at physiological pH, leading to higher solubility and improved pharmaceutical properties compared to those of phenylboronic acids. As a result, several benzoxaboroles have already been FDA approved, including Tavaborole (for treating onychomycosis) and Crisaborole (for treating atopic dermatitis), with Acoziborole under evaluation as an antiprotozoal drug for African trypanosomiasis (Scheme 1A).^13^ Given their relevance in medicinal chemistry, the search for novel benzoxaboroles typically focuses on peripheral modifications of the aromatic ring. A less explored approach targets the oxaborole unit itself, although only the C3 position allows for meaningful diversification. However, not all substituents can be introduced at this position without disrupting the oxaborole cycle, limiting functionalization to alkyl or aryl groups or secondary amines. For instance, 3-hydroxy-benzoxaboroles exhibit instability due to intramolecular hydrolysis of the hemiacetal (Scheme 1B). The equilibrium between 2-formyl phenylboronic acids and their corresponding 3-hydroxy-benzoxaboroles (*K*_cycl_) generally favors the open form, unless a strong electron-withdrawing group is present on the arene.^14,15^ This equilibrium is highly dependent on the solvent and temperature, and the pure cyclic form is virtually impossible to isolate. Furthermore, this equilibrium erases any stereochemical information at the C3 position of the benzoxaborole, which is the only potential stereogenic center of this unit. This limitation is significant, as the bioactivity of 2-formyl phenylboronic acids is largely attributed to trace amounts of the cyclic form rather than 2-formyl phenylboronic acids. This has been demonstrated for a series of 2-formyl fluoro-phenylboronic acids, where the 3-hydroxy form (an analog of Tavaborole with a hydrogen-bond donor) was responsible for high antifungal bioactivity.^16^ Therefore, introducing a stable H-bonding donor at the C3 position with stereocontrol enhances the bioactivity of benzoxaboroles by promoting their binding to enzymatic pockets. This remains an unsolved challenge in benzoxaborole chemistry as well as a limitation in the examples of chiral benzoxaboroles. To address this, we envisioned the introduction of a difluoromethyl group at the C3 position—a bioisostere of the hydroxy group known for its lipophilic H-bonding donor properties. Herein, we report the synthesis and properties of unprecedented 3-difluoromethyl-benzoxaboroles, including asymmetric variants, with an embedded H-bonding donor motif that promotes antimicrobial activity against *Escherichia coli* (Scheme 1C).

**Scheme 1:**
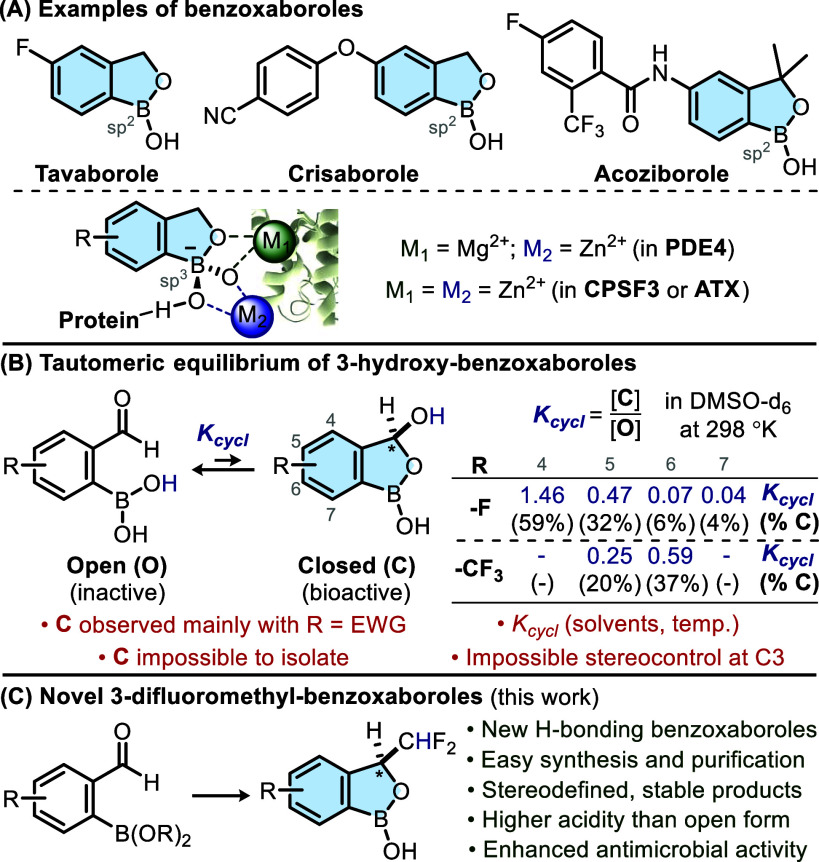
(A) Biologically Relevant Benzoxaboroles; (B) Equilibrium Involving 3-Hydroxy-benzoxaboroles;^14,15^ (C) Synthesis and Properties of 3-CHF_2_-Benzoxaboroles

Our investigation began by developing a synthetic method for the difluoromethylation of 2-formyl phenylboronic acid derivatives in analogy with the variety of nucleophilic additions to 2-formyl arylboronic acids or their derivatives already reported in the literature.^3^ It should be noted that, while trifluoromethylation reactions with the Ruppert–Prakash reagent (Me_3_SiCF_3_) are well-established, nucleophilic difluoromethylation additions to carbonyl compounds are less efficient because the Si–CHF_2_ bond is stronger than the Si–CF_3_ bond (bond order = 0.44 and 0.22, respectively).^17^ Nevertheless, inspired by the work of Hu and co-workers, we tested difluoromethylation reactions on substrate **I-[B]** using Me_3_SiCHF_2_ along with KO*t*Bu as an activator (Table 1).^18,19^ As with other syntheses of benzoxaboroles, upon nucleophilic addition (e.g., using NaBH_4_), the alcohol is not isolated but treated under acidic conditions to promote cyclization to the desired product. Similarly, we aimed to directly obtain **1** without isolating intermediate **Int-[B]**, hoping to quickly access novel 3-difluorobenzoxaboroles for further physical, chemical, and biological investigations (the main focus of the work).

**Table 1:**
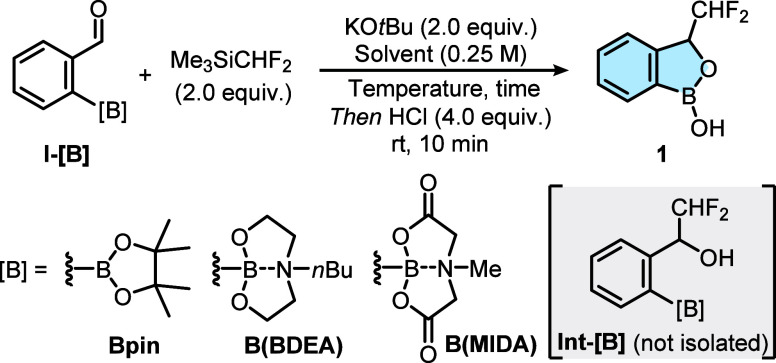
Optimization of the Reaction Forming **1**

Entry	[B]	Solvent	Temperature	Time	Yield^a^
1	Bpin	THF	–78 °C to rt	6 h	18%
2	B(BDEA)	THF	–78 °C to rt	6 h	20%
3	B(BDEA)	THF	–78 °C to rt	18 h	53%
4	B(BDEA)	THF:DME	–55 °C to rt	18 h	76% (73%)^b^
5	B(BDEA)	THF:diglyme	–55 °C to rt	18 h	57%
6^c^	B(BDEA)	THF:DME	–55 °C to rt	18 h	56%
7	B(BDEA)	THF:DME	–20 °C to rt	18 h	63%
8	B(MIDA) or B(OH)_2_	THF:DME	–55 °C to rt	18 h	0%

a NMR yield using CH_2_Br_2_ as internal standard.

b Isolated yield.

c Using 3 equiv of Me_3_SiCHF_2_ and KO*t*Bu.

Initial difluoromethylation reactions were attempted on substrate **I-[B]** equipped with pinacol boronic ester (Bpin) as the boron moiety. Dissolving **I-Bpin** and Me_3_SiCHF_2_ in THF and adding KO*t*Bu at −78 °C, followed by slow warming to room temperature and rapid treatment with HCl, yielded desired product **1** in 18% NMR yield (entry 1). The ^1^H NMR spectrum of this compound showed diagnostic signals at 5.74 ppm (td, ^2^ *J*_H–F1/2_ = 55.6 Hz, ^3^ *J*_H–H_ = 4.6 Hz, −CHF_2_), 5.29 ppm (ddd, ^3^ *J*_H–F1_ = 10.7 Hz, ^3^ *J*_H–F2_ = 9.2 Hz, ^3^ *J*_H–H_ = 4.6 Hz, C_3_–H), and 4.83 ppm (bs, OH), thus confirming correct cyclization of the intermediate. Moreover, the ^19^F NMR spectrum showed signals at −124.61 ppm (ddd, ^2^ *J*_F1–F2_ = 291.6 Hz, ^2^ *J*_F1–H_ = 55.9 Hz, ^3^ *J*_F1–H_ = 9.2 Hz) and −129.39 ppm (ddd, ^2^ *J*_F2–F1_ = 291.8 Hz, ^2^ *J*_F2–H_ = 55.2 Hz, ^3^ *J*_F2–H_ = 10.7 Hz), implying diastereotopic fluorine atoms due to the presence of a stereocenter on C3. However, optimization of the reaction conditions by varying the temperature or reaction time did not lead to further improvements nor did the use of other activators, such as CsF or KO*t*Pent (see Supporting Information for full screening). We hypothesized that protecting the electrophilic boron center could promote the desired nucleophilic addition to the carbonyl. Inspired by other syntheses of benzoxaboroles,^20^ we used **I–B­(BDEA)** (BDEA = *N*-butyldiethanolamine) as the substrate. This modification led to a significant improvement when the reaction was left for 18 h with slow warming from −78 °C to room temperature (entry 3). The addition of the cosolvent dimethoxyethane (DME), a strategy employed in related difluoromethylation reactions,^21^ further increased the yield to 76% with clean product **1** isolated in 73% yield by column chromatography (entry 4). In this case, the minimum temperature was set to −55 °C to avoid freezing of the cosolvent, whereas the use of DME as the only solvent was not possible due to the insolubility of KO*t*Bu in DME. The latter also proved superior to diglyme as the cosolvent, while increasing the equivalents of reagents or the temperature was not beneficial (entries 5–7). Finally, replacing the boron moiety of the substrate with B­(MIDA) or B­(OH)_2_ gave no product, highlighting their incompatibility under nucleophilic conditions. With the optimized conditions in hand, a series of 3-difluoromethyl-benzoxaboroles was rapidly synthesized (Scheme 2A). All the products were isolated by column chromatography, thus showing stability on silica gel. First, the synthesis of **1** was scaled up starting from one gram of **I–B­(BDEA)** with minimal erosion of the isolated yield (65%).

**Scheme 2:**
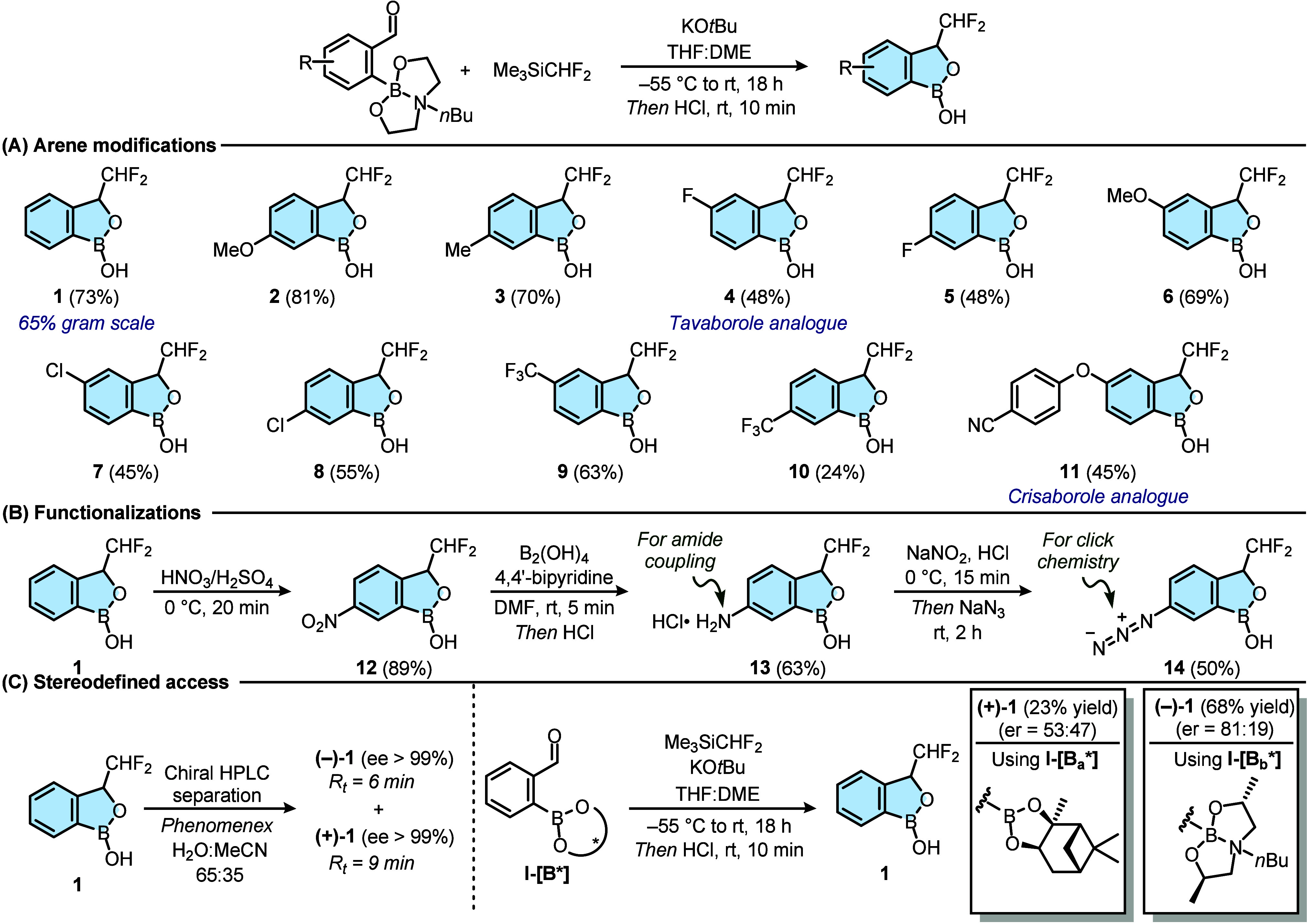
Series of Substituted 3-Difluoromethyl-benzoxaboroles^a^

a Isolated yields in brackets.

Second, in contrast to 3-hydroxy-benzoxaboroles, 3-difluoromethyl analogs were not limited to strong electron-withdrawing groups on the arene, as shown for compounds bearing a methoxy (**2** and **6**) or a methyl (**3**) group. Moreover, the C3-difluoromethyl analogue of Tavaborole (**4**) and its regioisomer **5** could be accessed too. Other functionalities were also tolerated, such as −Cl in **7**–**8**, −CF_3_ in **9**–**10**, and −CN in **11**, the latter representing the C3-difluoromethyl analogue of Crisaborole. However, substituents *ortho* to the aldehyde complicated the installation of the BDEA ligand (a similar buttressing effect has been observed in other benzoxaboroles),^14^ whereas no boronic acids with a substituent *ortho* to the boron moiety could be easily synthesized due to steric congestion. Nevertheless, functionalization of the benzoxaborole unit allows for strategic decoration (Scheme 2B). Indeed, a high-yield nitration of **1** produced nitro-derivative **12**, which could then be reduced to the corresponding aniline (conveniently isolated in its protonated form as **13**). The latter could also be converted to aryl azide **14**, thus representing, together with **13**, highly valuable motifs for bioconjugation, a common strategy to link benzoxaboroles to biomolecules.^22,23^ With several examples demonstrating the tolerance of electron-withdrawing and electron-donating groups, our attention focused on obtaining enantioenriched compound **1**. First, a chiral resolution allowed the isolation of both **(+)-1** and **(−)-1** in excellent enantiomeric ratios through chiral HPLC on the amylose stationary phase (Scheme 2C). Second, asymmetric difluoromethylation reactions were also attempted by introducing a chiral auxiliary on the boron unit. Using (+)-pinanediol (in **I-[B**_**a**_ *]), a well-established ligand in boron chemistry, negligible chiral induction was observed. However, the use of **I-[B**_**b**_ *] (ligand synthesized in one step from commercial chiral sources) resulted in an 81:19 enantiomeric ratio. This result highlights the superior performance of boronates and represents a promising proof of concept for future asymmetric developments.

At this point, our attention has focused on the physical properties of this novel class of benzoxaboroles. First, compound **1** showed no signs of degradation after months of exposure to air in solid form or after a week in DMSO solution. Second, X-ray diffraction (XRD) analysis revealed that **1** crystallizes as a centrosymmetric dimer of two coplanar enantiomers, stabilized by hydrogen bonds between the hydroxy groups and the oxaborole oxygen atoms (red dotted lines in Figure 1). Such flat dimeric structures, with the OH in the *syn*-conformation, are typical of benzoxaboroles, often forming layers stabilized by hydrogen bonds with trapped water molecules.^2,24^ In contrast, no water is present in the crystal structure of **1**; instead, the layers are stabilized by interactions involving the −CHF_2_ groups (blue dotted lines), suggesting that the difluoromethyl group serves as an effective hydrogen bond donor, comparable to water. These additional stabilizations, observed only in 3-difluoromethyl benzoxaboroles, may reinforce the importance of incorporating a stable H-bond donor in the C3-position. Moreover, the endocyclic C–B–O angle in **1** (measured at 107.8°) reflects the strain of the five-membered oxaborole ring (reported at 108.6° for the unsubstituted benzoxaborole).^2^ Third, the acidity (p*K*_a_ of **1-H**_**2**_ **O**) was measured potentiometrically, following established methods for benzoxaboroles (see Supporting Information).^25^ The introduction of a difluoromethyl group at C3 had a minimal impact on the acidity of the oxaborole core. This is important, as the inherently higher acidity of benzoxaboroles relative to aryl boronic acids can be advantageous for pharmaceutical applications (*vide infra*); thus, acidity changes that are not beyond expectation are a positive outcome. The acidity of other 3-difluoromethyl-benzoxaboroles can instead be predicted knowing that the effects of a substituent on the aromatic ring follow a Hammett relationship.^26,27^ Direct assessment of the Lewis acidity of **1** was instead measured by Gutmann–Beckett tests. Specifically, coordination of Et_3_PO to **1** was found to be similar to that of the unsubstituted benzoxaborole (δ^31^P NMR = 4.8 and 5.8 ppm, respectively), thus confirming unaltered electronic properties around the boron atom. It has to be noted that the complexation observed may be an equilibrium; thus, the δ^31^P NMR values may be affected by this (or by the instauration of hydrogen bonding).^28^

**Figure 1:**
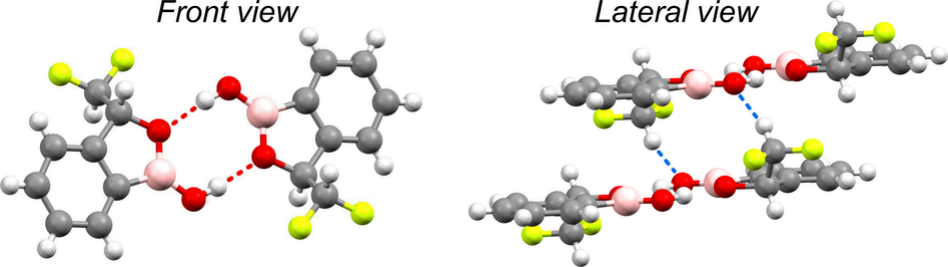
XRD analysis of **1**.

Finally, the effect of the difluoromethyl group on the biological activities was investigated. It has been shown that benzoxaboroles can directly act as antimicrobials (mainly against *Escherichia coli*) by inhibiting Penicillin Binding Proteins or Leucyl-tRNA synthetase (LeuRS) or, alternatively, by reversing antibacterial resistance through the inhibition of β-lactamases.^29,30^ Thus, the novel compounds were further tested for their ability to reduce 50% of the bacterial growth (IC_50_) of *E. coli* MG1655 in a Minimal Inhibitory Concentration (MIC) assay (Table 2). Benzoxaborole **15**, bearing a −CH_3_ group instead of −CHF_2_, was also tested as a mimic of **1**, with a similar steric hindrance but without an H-bonding donor on C3. Additionally, 2-formyl phenyl boronic acid was tested to confirm the bioactivity of its cyclic form (3-hydroxy-benzoxaborole **16a**, as measured by ^1^H NMR analysis in DMSO-*d*_6_). First, the effect of the C3 substituent was evaluated: compound **1** was found to be the most potent benzoxaborole with an activity 7 times stronger than **15**, highlighting the importance of the electronic properties (rather than steric) of the C3 substituent. Indeed, **16a**, bearing an OH group, demonstrated greater activity than **15** despite being present at lower concentrations; however, its tautomeric equilibrium with the open form diminishes its effectiveness, resulting in a lower activity than **1**. Second, compound **4** was compared with other 5-fluoro-benzoxaboroles, as Sporzyński and co-workers showed that a fluorine atom in this position is crucial for antimicrobial activity.^15,31^ Also in this case, 3-difluoromethyl-benzoxaborole outperformed 3-hydroxy-benzoxaborole (**4** vs **17a**), with the latter being present in 32% in solution. Impressively, compound **4** was only slightly less potent than Tavaborole and was as potent as compound **1**, demonstrating that the effect of the −CHF_2_ group at the C3 position is now as important as the fluorine atom at the C5 position. This helps partly counteract the drop in bioactivity caused by the absence of the fluorine atom at position 5 (e.g., compound **5**) or, in some cases, even overrides this effect, as demonstrated by the similar potency of **3** and Tavaborole. In addition, subinhibitory concentrations of these compounds showed an antibiofilm activity with compounds **6**, **5**, and **3** being more effective than Tavaborole (Figure S8), hence further supporting their bioactivity against *E. coli* MG1655. Altogether, the SAR study opens the possibility of exploring a broader chemical space for new antimicrobials that lack a fluorine atom on the aromatic ring but incorporate an −CHF_2_ group at C3.

**Table 2:**
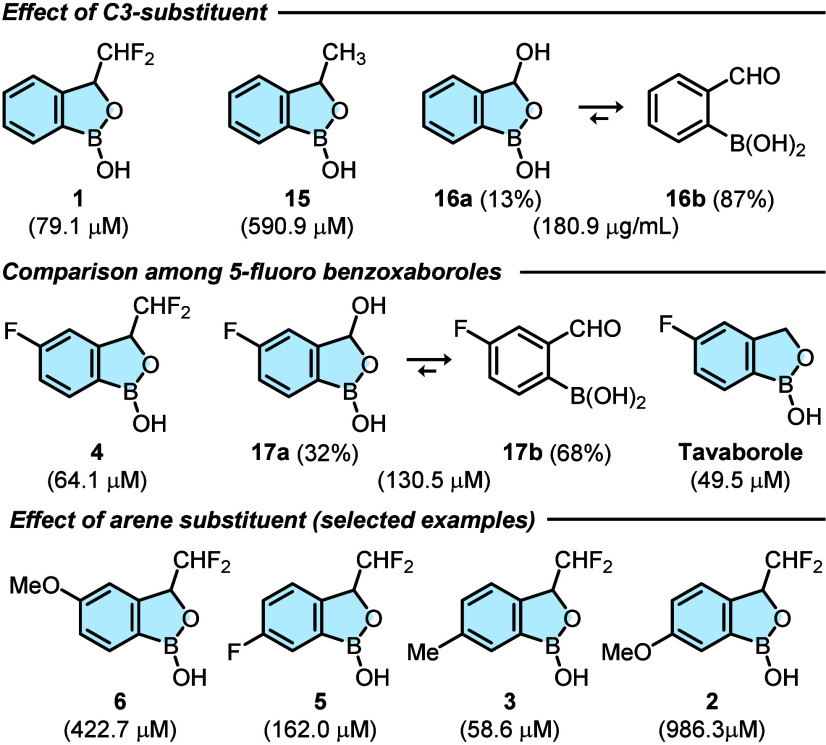
Antimicrobial Activity against *E. coli* MG1655 of Benzoxaboroles^a^

a IC_50_ in brackets, expressed in μM.

In summary, 3-difluoromethyl-benzoxaboroles were developed as practical alternatives to synthetically challenging 3-hydroxy analogs. A one-pot difluoromethylation reaction, also demonstrated on a gram scale, enables the synthesis of diverse derivatives and provides access to both enantiomers through chiral resolution. The −CHF_2_ group preserves benzoxaborole stereoelectronics while enhancing the bioactivity via strong hydrogen bonding.

## Supplementary Material



## Data Availability

The data underlying this study are available in the published article and its Supporting Information.

## Notes

NMR characterizations, acidity measurements, XRD analysis, and SAR studies (PDF)
