# Supplementary material for: Stereodefined Synthesis of 3‑Difluoromethyl-Benzoxaboroles: Novel Antimicrobials with Unlocked H‑Bonding
Source: Org Lett. 2026 Jan 22;28(5):1527–32. doi: 10.1021/acs.orglett.5c04602 (PMC12887987; doi:10.1021/acs.orglett.5c04602)
Supplement: Supplementary file 1 [file ol5c04602_si_001.pdf]

Supporting Information for

Stereodefined Synthesis of 3-Difluoromethyl-Benzoxaboroles: Novel Antimicrobials with Unlocked H-Bonding

Alessandro Dimasi,[a] Arianna Montoli,[a] Claudio Lutti,[a] Andrea Citarella,[a] Paolo Ronchi,[b] Francesco Castagnini,[c] Valentina Mileo,[b] Giovanni Macetti,[a] Valerio Baldelli,[a] Elio Rossi,[a] Paolo Landini,[a] Daniele Passarella,[a] and Valerio Fasano[a]*

Correspondence to: valerio.fasano@unimi.it

www.fasanolab.com

[a] A. Dimasi, A. Montoli, C. Lutti, A. Citarella, G. Macetti, V. Baldelli, E. Rossi, P. Landini, D. Passarella, V. Fasano
Università degli Studi di Milano, Via Camillo Golgi, 19, 20133 Milano, Italy

[b] P. Ronchi, V. Mileo
Chiesi Farmaceutici S.p.A., Largo Francesco Belloli 11/a, 43122 Parma, Italy

[c] F. Castagnini
Università di Parma, Parco Area delle Scienze 27/A, 43124 Parma, Italy

TABLE OF CONTENTS

1. MATERIALS AND GENERAL METHODS 2
1.1. General considerations........ 2
1.2. Naming of Compounds........ 2
2. EXPERIMENTAL DATA........ 3
2.1. General Procedures. 3
2.1.1 General Procedure A (GP-A): Synthesis of B(BDEA) complexes 3
2.1.2 General Procedure B (GP-B): Synthesis of 3-difluoromethylated benzoxaboroles 3
2.2 Conditions Optimization........ 4
2.2.1 Condition optimizations using 2-formylphenyl boronic acid pinacol ester........ 4
2.2.2 Conditions optimization using 2-formylphenyl boronate complexes 5
2.3 Substrate Scope 6
2.3.1 Synthesis of B(BDEA) complexes. 6
2.3.2 Synthesis of 3-difluoromethylated benzoxaboroles 9
2.3.3 Synthesis of 3-difluromethyl Crisaborole analogue........ 13
2.3.4 Functionalisation of 3-difluoromethyl benzoxaboroles 15
2.3.5 Enantioenriched synthesis 17
2.3.6 Synthesis of other benzoxaboroles 20
2.4 Stability test 20
2.5 pK_a determination 21
2.6 Gutmann-Beckett method measurements........ 21
2.7 Single-crystal X-ray diffraction analysis........ 23
2.7.1 Single-crystal X-ray diffraction of racemic mixture 23
2.8 Biological evaluation........ 28
2.8.1 Minimal Inhibitory Concentration........ 28
2.8.2 Biofilm formation assay 30
3. SPECTROSCOPIC DATA 31
4. HPLC TRACES........ 87
5. MINIMAL INHIBITORY CONCENTRATION ASSAYS........ 89
6. BIOFILM FORMATION ASSAYS 91
7. REFERENCES 92

1. MATERIALS AND GENERAL METHODS

1.1. General considerations

Unless stated, all starting materials and anhydrous solvents were obtained from commercial sources and used without purification. Reactions were carried out under an inert atmosphere of nitrogen unless stated. Reaction progress was monitored by TLC, with ^{1}H NMR or LC-MS analyses taken from reaction samples. Column chromatography was performed on silica gel (230-400 mesh). NMR spectra were recorded with a Bruker AV-400 spectrometer (400 MHz ^{1}H; 101 MHz ^{13}C; 128 MHz ^{11}B; 376 MHz ^{19}F). ^{1}H NMR chemical shifts are reported in ppm relative to protio impurities in the deuterated solvents and reported as follow: chemical shift (multiplicity, coupling constants, number of protons). ^{13}C NMR chemical shifts are reported in ppm using the solvent resonance. In the ^{13}C NMR spectra of all compounds the carbon adjacent to the boron atom was not observed due to long relaxation time. ^{11}B NMR spectra were recorded using $BF_3{\cdot}Et_2O$ as an external reference, while ^{19}F NMR spectra were recorded using Cl_3CF. Coupling constants J are given in Hertz (Hz), while the multiplicity of the signals are indicated as “s”, “d”, “t”, “q”, “pent”, “sept” or “m” for singlet, doublet, triplet, quartet, pentet, septet or multiplet, respectively. Mass spectra were recorded on a Waters QTOF mass spectrometer by Electrospray Ionization (ESI). Chiral HPLC analysis was performed on an Agilent 1100 Series System using a Lux 5 µm i-Amylose-3 (Phenomenex) column (250 x 4.6 mm) in isocratic conditions of H_2O/ACN 65:35 in 12 min (flux of 1.0 mL/min and sample injection of 25 µL), choosing 254 nm as the wavelength for the detection of compounds. Optical rotations were measured on a JASCO P-1030 polarimeter at 589 nm of a sodium (Na) lamp using a 1 mL cell, with a length of 1 dm. X-ray diffraction analysis were obtained on a Rigaku XtaLAB Synergy-S 4-circle diffractometer using a microfocus sealed tube as a source and Hybrid Photon Counting (HPC) as a detector. The Half maximal inhibitory concentration (IC_{50}) of the compounds **1-17b** was evaluated with the standard microdilution method as previously described, with minor modifications.[1,2]

Note. In boron-containing compounds, the carbon directly attached to the boron could not be detected in the ^{13}C NMR, likely due to quadrupole relaxation.

1.2. Naming of Compounds

Compound names are those generated by ChemDraw Professional 20.0 software (PerkinElmer), following the IUPAC nomenclature.

2. EXPERIMENTAL DATA

2.1. General Procedures.

2.1.1 General Procedure A (GP-A): Synthesis of B(BDEA) complexes

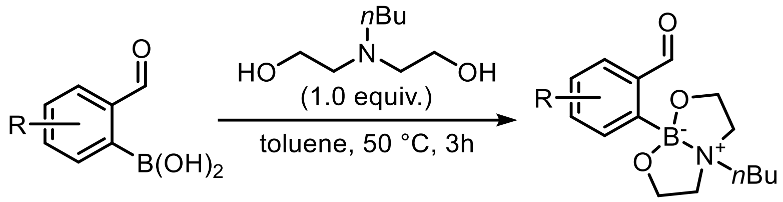

The procedure has been adapted from the literature.[3] A round bottom flask equipped with a stir bar is charged with 2-formyl phenyl boronic acid derivatives (1.0 equiv.) and N-Butyldiethanolamine (1.0 equiv.), Then, toluene (0.25 M) is added, and the reaction is stirred at 50 °C in an oil bath for 3 hours. After that, the reaction is concentrated under reduced pressure, and the remaining yellow oil is triturated in n-Hexane. The solid that precipitated is collected by filtration, washed with n-Hexane, and dried in vacuum to provide pure **B(BDEA)** complexes as a white amorphous solid.

2.1.2 General Procedure B (GP-B): Synthesis of 3-difluoromethylated benzoxaboroles

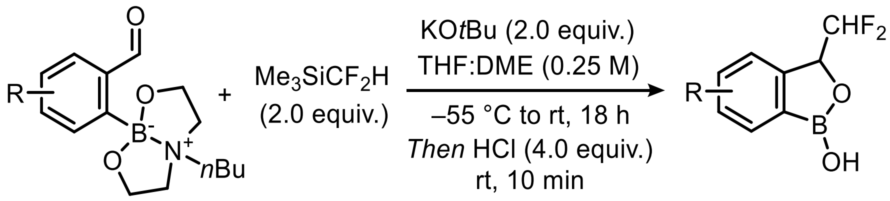

A double necked round bottom flask equipped with a stir bar is dried with Schlenk technique. Under N_2 atmosphere, **B(BDEA)** complex (1.0 equiv.) is added and dissolved in DME (0.50 M). Then, mixture is brought to −55°C and Me_3SiCF_2H (2.0 equiv.) is added, followed by KO*t*Bu (1.0 M in THF, 2.0 equiv., 0.50 M) and the reaction is stirred from −55°C to rt for 18 hours. After that, 1.0 M HCl (4.0 equiv.) is added and the reaction is stirred at room temperature for 10 minutes. The reaction is diluted with AcOEt and the two phases are separated. The aqueous phase is extracted 3 times with AcOEt. Organic phases are collected, dried over Na_2SO_4, filtered and concentrated under reduced pressure. The obtained crude is purified on flash chromatography column to provide **3-difluromethylated benzoxaboroles** as white crystalline solids.

2.2 Conditions Optimization

2.2.1 Condition optimizations using 2-formylphenyl boronic acid pinacol ester

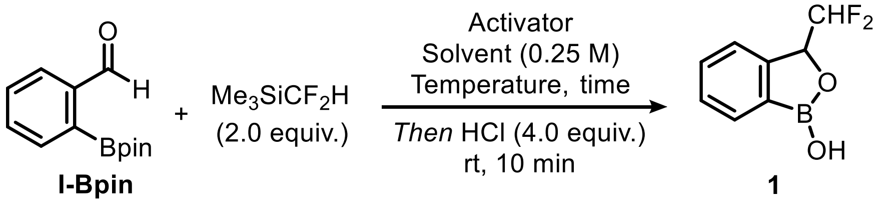

I-Bpin **1**

**Entry**	**Activator**	**Solvent**	**Temperature**	**Time**	**Yield**[a]
**1**	KO*t*Bu (2.0 equiv)	THF	–78 °C to rt	6 h	18%
**2**	KO*t*Bu (2.0 equiv)	THF	–60 °C to rt	6 h	12%
**3**	KO*t*Bu (2.0 equiv)	THF	–40 °C to rt	6 h	22%
**4**	KO*t*Bu (2.0 equiv)	THF	–20 °C to rt	6 h	16%
**5**	KO*t*Bu (2.0 equiv)	THF	0 °C to rt	6 h	traces
**6**	KO*t*Bu (2.0 equiv)	THF	–78 °C to rt	18 h	22%
**7**	CsF (14%mol)	DMF	rt	18 h	0%
**8**	CsF (50%mol)	DMF	rt	18 h	0%
**9**	CsF (2.0 equiv))	DMF	rt	18 h	0%
**10**[b]	KO*t*Bu (2.0 equiv)	THF	–78 °C to rt	6h	0%

[a]NMR yield using CH_2Br_2 as internal standard.
[b]Using 2-formylphenyl boronic acid as substrate.

2.2.2 Conditions optimization using 2-formylphenyl boronate complexes

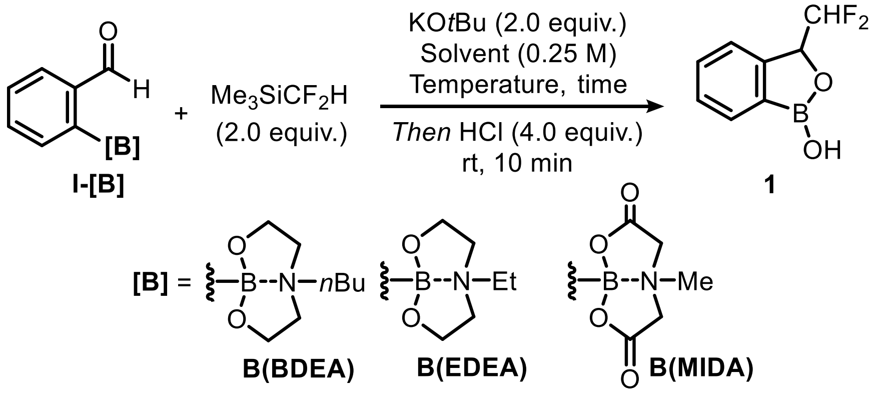

Entry	[B]	Solvent	Temperature	Time	Yield[a]
1	B(BDEA)	THF	–78 °C to rt	6 h	20%
2	B(BDEA)	THF	–78 °C to rt	18 h	53%
3	B(BDEA)	THF:DME	–55 °C to rt	18 h	76% (73%)[b]
4	B(BDEA)	THF:Toluene	–78 °C to rt	18 h	20%
5	B(BDEA)	THF:DCM	–78 °C to rt	18 h	0%
6	B(BDEA)	THF:DMF	–78 °C to rt	18 h	40%
7	B(BDEA)	THF:diglyme	–55 °C to rt	18 h	57%
8[c]	B(BDEA)	THF:DME	–55 °C to rt	18 h	56%
9	B(BDEA)	THF:DME	–20 °C to rt	18 h	63%
10	B(BDEA)	THF:DME	–40 °C to rt	18 h	51%
11	B(BDEA)	DME	–55 °C to rt	18 h	0%
12	B(EDEA)	THF	–78 °C to rt	18 h	14%
13	B(EDEA)	THF:DME	–55 °C to rt	18 h	50%
14	B(MIDA)	THF	–78 °C to rt	18 h	0%
15	B(MIDA)	THF:DME	–55 °C to rt	18 h	0%

[a]NMR yield using CH_2Br_2 as internal standard. [b]Isolated yield.
[c]Using 3 equivalents of Me_3SiCF_2H and KO*t*Bu.

2.3 Substrate Scope

2.3.1 Synthesis of B(BDEA) complexes.

Synthesis of 2-(4-butyltetrahydro-2H-4λ^4,8λ^4-[1,3,2]oxazaborolo[2,3-*b*][1,3,2]oxazaborol-8-yl)benzaldehyde **I-B(BDEA)**

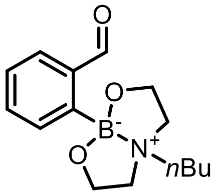

I-B(BDEA)

Prepared according to general procedure A (GP-A) using (2-formylphenyl)boronic acid (500 mg, 3,33 mmol, 1.0 equiv.) N-butyldiethanolamine (536 mg, 3.33 mmol, 1.0 equiv) and toluene (13 mL) to obtain the title compound **I-B(BDEA)** as a white amorphous solid (890 mg, **97% yield**). ^{1}H NMR (400 MHz, Acetone-*d*$_6$) δ 10.91 (s, 1H), 7.88 (dd, J = 7.5, 1.4 Hz, 1H), 7.79 (dd, J = 7.7, 1.4 Hz, 1H), 7.47 (td, J = 7.4, 1.4 Hz, 1H), 7.39 – 7.31 (m, 1H), 4.24 – 4.00 (m, 4H), 3.40 – 3.16 (m, 4H), 2.45 – 2.36 (m, 2H), 1.72 – 1.55 (m, 2H), 1.10 (h, J = 7.4 Hz, 2H), 0.78 (t, J = 7.4 Hz, 3H). ^{13}C NMR (101 MHz, Acetone-*d*$_6$) δ 197.4, 143.3, 136.9, 132.5, 128.3, 126.5, 64.2, 61.4, 58.8, 27.6, 20.8, 13.9.^{11}B NMR (128 MHz, Acetone-*d*$_6$) δ 12.9. HRMS (ESI), m/z [M+H]$^+$: Calculated for $[C_{15}H_{23}BNO_3]^+$ 276.1766, Found 276.1776 (see spectrum)

Synthesis of 2-(4-butyltetrahydro-2H-4λ^4,8λ^4-[1,3,2]oxazaborolo[2,3-*b*][1,3,2]oxazaborol-8-yl)-4-methoxybenzaldehyde **II-B(BDEA)**

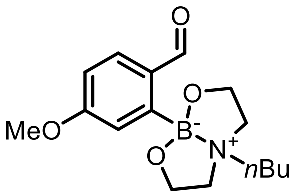

II-B(BDEA)

Prepared according to general procedure A (GP-A) using (2-formyl-5-methoxyphenyl)boronic acid (180 mg, 1.0 mmol, 1.0 equiv.) N-butyldiethanolamine (161 mg, 1.0 mmol, 1.0 equiv) and toluene (4 mL) to obtain the title compound **II-B(BDEA)** as a white amorphous solid (274 mg, **90% yield**). ^{1}H NMR (400 MHz, Acetone-*d*$_6$) δ 10.77 (s, 1H), 7.86 (d, J = 8.6 Hz, 1H), 7.41 (d, J = 2.8 Hz, 1H), 6.89 (dd, J = 8.8, 2.7 Hz, 1H), 4.12 (dt, J = 15.3, 9.3 Hz, 4H), 3.85 (s, 3H), 3.41 – 3.18 (m, 4H), 2.48 – 2.36 (m, 2H), 1.64 (tt, J = 8.0, 6.2 Hz, 2H), 1.11 (h, J = 7.5 Hz, 2H), 0.79 (t, J = 7.6 Hz, 3H). ^{13}C NMR (101 MHz, Acetone-*d*$_6$) δ 195.5, 163.7, 136.6, 129.2, 121.5, 113.8, 64.2, 61.2, 58.7, 55.4, 27.6, 20.8, 14.0. ^{11}B NMR (128 MHz, Acetone-*d*$_6$) δ 12.6. HRMS (ESI), m/z [M+H]$^+$: Calculated for $[C_{16}H_{25}BNO_4]^+$ 306.1871, Found 306.1875 (see spectrum)

Synthesis of 2-(4-butyltetrahydro-2H-4λ^4,8λ^4-[1,3,2]oxazaborolo[2,3-*b*][1,3,2]oxazaborol-8-yl)-4-methylbenzaldehyde **III-B(BDEA)**

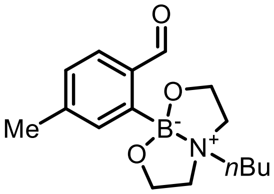

III-B(BDEA)

Prepared according to general procedure A (GP-A) using (2-formyl-5-methylphenyl)boronic acid (164 mg, 1.0 mmol, 1.0 equiv.) N-butyldiethanolamine (161 mg, 1.0 mmol, 1.0 equiv) and toluene (4 mL) to obtain the title compound **III-B(BDEA)** as a white amorphous solid (288 mg, **99% yield**). ^{1}H NMR (400 MHz, Acetone-*d*$_6$) δ 10.85 (d, J = 0.9 Hz, 1H), 7.73 (d, J = 7.9 Hz, 1H), 7.72 – 7.69 (m, 1H), 7.20 – 7.13 (m, 1H), 4.25 – 3.99 (m, 4H), 3.33 (ddd, J = 11.6, 5.2, 2.2 Hz, 2H), 3.30 – 3.16 (m, 2H), 2.45 – 2.38 (m, 2H), 2.35 (s, 3H), 1.69 – 1.55 (m, 2H), 1.11 (dt, J = 15.0, 7.5 Hz, 2H), 0.78 (t, J = 7.4 Hz, 3H). ^{13}C NMR (101 MHz, Acetone-*d*$_6$) δ 196.8, 142.6, 140.9, 137.5,

129.1, 126.8, 64.2, 61.3, 58.7, 27.6, 21.9, 20.8, 14.0. ^{11}B NMR (128 MHz, Acetone-d_6) δ 12.88. HRMS (ESI), m/z $[M+H]^+$: Calculated for $[C_{16}H_{25}BNO_3]^+$ 290.1922, Found 290.1933. (see spectrum)

Synthesis of 2-(4-butyltetrahydro-2H-4λ^4,8λ^4-[1,3,2]oxazaborolo[2,3-*b*][1,3,2]oxazaborol-8-yl)-5-fluorobenzaldehyde **IV-B(BDEA)**

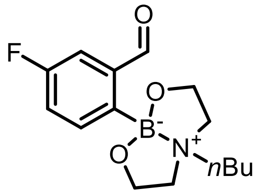

IV-B(BDEA)

Prepared according to general procedure A (GP-A) using (4-fluoro-2-formylphenyl)boronic acid (164 mg, 1.0 mmol, 1.0 equiv.) N-butyldiethanolamine (161 mg, 1.0 mmol, 1.0 equiv) and toluene (4 mL) to obtain the title compound **IV-B(BDEA)** as a white amorphous solid (276 mg, **94% yield**). ^{1}H NMR (400 MHz, Acetone-d_6) δ 10.87 (d, J = 3.6 Hz, 1H), 7.92 (dd, J = 8.4, 6.4 Hz, 1H), 7.45 (dd, J = 10.1, 2.8 Hz, 1H), 7.24 (td, J = 8.5, 2.8 Hz, 1H), 4.26 – 4.03 (m, 4H), 3.45 – 3.19 (m, 4H), 2.47 – 2.36 (m, 2H), 1.72 – 1.58 (m, 2H), 1.12 (h, J = 7.4 Hz, 2H), 0.79 (t, J = 7.4 Hz, 3H). ^{13}C NMR (151 MHz, Acetone-d_6) δ 196.1 (d, J = 2.1 Hz), 163.6 (d, J = 245.2 Hz), 145.1 (d, J = 5.2 Hz), 139.5 (d, J = 6.5 Hz), 119.3 (d, J = 19.7 Hz), 112.1 (d, J = 20.8 Hz), 64.2, 61.5, 58.8, 27.6, 20.7, 13.9. ^{11}B NMR (128 MHz, Acetone-d_6) δ 12.28. ^{19}F NMR (376 MHz, Acetone-d_6) δ -116.6. HRMS (ESI), m/z $[M+H]^+$: Calculated for $[C_{15}H_{22}BFNO_3]^+$ 294.1671, Found 294.1665 (see spectrum)

Synthesis of 2-(4-butyltetrahydro-2H-4λ^4,8λ^4-[1,3,2]oxazaborolo[2,3-*b*][1,3,2]oxazaborol-8-yl)-4-fluorobenzaldehyde **V-B(BDEA)**

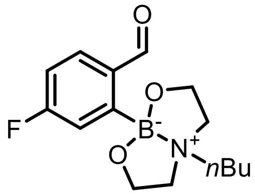

V-B(BDEA)

Prepared according to general procedure A (GP-A) using (5-fluoro-2-formylphenyl)boronic acid (168 mg, 1.0 mmol, 1.0 equiv.) N-butyldiethanolamine (161 mg, 1.0 mmol, 1.0 equiv) and toluene (4 mL) to obtain the title compound **V-B(BDEA)** as a white amorphous solid (286 mg, **78% yield**). ^{1}H NMR (400 MHz, Acetone-d_6) δ 10.82 (d, J = 0.8 Hz, 1H), 7.89 (dd, J = 8.6, 5.9 Hz, 1H), 7.55 (dd, J = 10.7, 2.8 Hz, 1H), 7.09 (tdd, J = 8.5, 2.8, 0.9 Hz, 1H), 4.20 – 4.03 (m, 4H), 3.39 (ddd, J = 12.0, 5.3, 2.5 Hz, 2H), 3.35 – 3.26 (m, 2H), 2.51 – 2.42 (m, 2H), 1.71 – 1.61 (m, 2H), 1.13 (h, J = 7.4 Hz, 2H), 0.79 (t, J = 7.4 Hz, 3H). ^{13}C NMR (101 MHz, Acetone-d_6) δ 195.5, 166.2 (d, J = 252.1 Hz), 139.7, 129.8 (d, J = 8.7 Hz), 122.6 (d, J = 18.9 Hz), 115.4 (d, J = 22.5 Hz), 64.3, 61.5, 58.9, 27.6, 20.7, 14.0. ^{11}B NMR (128 MHz, Acetone-d_6) δ 12.27. ^{19}F NMR (376 MHz, Acetone-d_6) δ -109.3. HRMS (ESI), m/z $[M+H]^+$: Calculated for $[C_{15}H_{22}BFNO_3]^+$ 294.1671, Found 294.1673 (see spectrum)

Synthesis of 2-(4-butyltetrahydro-2H-4λ^4,8λ^4-[1,3,2]oxazaborolo[2,3-*b*][1,3,2]oxazaborol-8-yl)-5-methoxybenzaldehyde **VI-B(BDEA)**

Prepared according to general procedure A (GP-A) using (2-formyl-4-methoxyphenyl)boronic acid (180 mg, 1.0 mmol, 1.0 equiv.) N-butyldiethanolamine (161 mg, 1.0 mmol, 1.0 equiv) and toluene (4

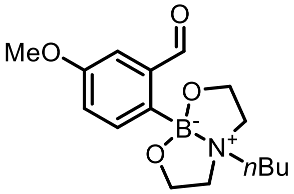

VI-B(BDEA)

mL) to obtain the title compound **VI-B(BDEA)** as a white amorphous solid (301 mg, **98% yield**). ^{1}H NMR (400 MHz, Acetone-d_6) δ 10.88 (s, 1H), 7.79 (d, J = 8.3 Hz, 1H), 7.34 (d, J = 2.7 Hz, 1H), 7.06 (dd, J = 8.4, 2.8 Hz, 1H), 4.19 – 4.00 (m, 4H), 3.82 (s, 3H), 3.38 – 3.28 (m, 2H), 3.28 – 3.15 (m, 2H), 2.46 – 2.33 (m, 2H), 1.70 – 1.51 (m, 2H), 1.11 (h, J = 7.4 Hz, 2H), 0.79 (t, J = 7.4 Hz, 3H). ^{13}C NMR (101 MHz, Acetone-d_6) δ 197.0, 160.3, 144.3, 138.5, 119.5, 110.0, 64.1, 61.4, 58.6, 55.3, 27.6, 20.8, 14.0. ^{11}B NMR (128 MHz, Acetone-d_6) δ 13.03. HRMS (ESI), m/z $[M+H]^+$: Calculated for $[C_{16}H_{25}BNO_4]^+$ 306.1871, Found 306.1877 (see spectrum)

Synthesis of 2-(4-butyltetrahydro-2H-4λ^4,8λ^4-[1,3,2]oxazaborolo[2,3-*b*][1,3,2]oxazaborol-8-yl)-5-chlorobenzaldehyde **VII-B(BDEA)**

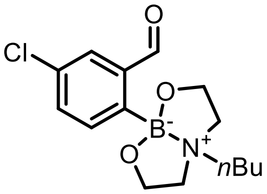

VII-B(BDEA)

Prepared according to general procedure A (GP-A) using (4-chloro-2-formylphenyl)boronic acid (184 mg, 1.0 mmol, 1.0 equiv.) N-butyldiethanolamine (161 mg, 1.0 mmol, 1.0 equiv) and toluene (4 mL) to obtain the title compound **VII-B(BDEA)** as a white amorphous solid (290 mg, **94% yield**). ^{1}H NMR (600 MHz, Acetone-d_6) δ 10.85 (s, 1H), 7.88 (d, J = 8.1 Hz, 1H), 7.72 (d, J = 2.3 Hz, 1H), 7.48 (dd, J = 8.1, 2.3 Hz, 1H), 4.14 (td, J = 10.1, 5.4 Hz, 2H), 4.09 (ddd, J = 9.9, 7.3, 2.3 Hz, 2H), 3.38 (ddd, J = 12.0, 5.3, 2.3 Hz, 2H), 3.29 (td, J = 11.1, 7.4 Hz, 2H), 2.51 – 2.38 (m, 2H), 1.76 – 1.60 (m, 2H), 1.12 (h, J = 7.5 Hz, 2H), 0.79 (t, J = 7.4 Hz, 3H). ^{13}C NMR (151 MHz, Acetone-d_6) δ 196.1, 144.7, 139.1, 134.4, 132.2, 126.1, 64.2, 61.5, 58.8, 27.6, 20.7, 14.0. ^{11}B NMR (128 MHz, Acetone-d_6) δ 12.14. HRMS (ESI), m/z $[M+H]^+$: Calculated for $[C_{15}H_{22}BClNO_3]^+$ 310.1376, Found 310.1382 (see spectrum)

Synthesis of 2-(4-butyltetrahydro-2H-4λ^4,8λ^4-[1,3,2]oxazaborolo[2,3-*b*][1,3,2]oxazaborol-8-yl)-4-chlorobenzaldehyde **VIII-B(BDEA)**

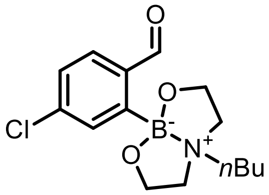

VIII-B(BDEA)

Prepared according to general procedure A (GP-A) using (5-chloro-2-formylphenyl)boronic acid (184 mg, 1.0 mmol, 1.0 equiv.) N-butyldiethanolamine (161 mg, 1.0 mmol, 1.0 equiv) and toluene (4 mL) to obtain the title compound **VIII-B(BDEA)** as a white amorphous solid (290 mg, **94% yield**). ^{1}H NMR (400 MHz, Acetone-d_6) δ 10.83 (d, J = 0.8 Hz, 1H), 7.87 (d, J = 2.3 Hz, 1H), 7.79 (d, J = 8.3 Hz, 1H), 7.38 (ddd, J = 8.3, 2.3, 0.8 Hz, 1H), 4.20 – 4.11 (m, 2H), 4.14 – 4.05 (m, 2H), 3.40 (ddd, J = 11.9, 5.3, 2.4 Hz, 2H), 3.38 – 3.26 (m, 2H), 2.53 – 2.43 (m, 2H), 1.73 – 1.61 (m, 2H), 1.13 (h, J = 7.4 Hz, 2H), 0.80 (t, J = 7.4 Hz, 3H). ^{13}C NMR (101 MHz, $CDCl_3$) δ 196.0, 141.6, 138.9, 136.5, 128.7, 128.5, 64.3, 61.5, 58.9, 27.6, 20.7, 14.0. ^{11}B NMR (128 MHz, Acetone-d_6) δ 12.1. HRMS (ESI), m/z $[M+H]^+$: Calculated for $[C_{15}H_{22}BClNO_3]^+$ 310.1376, Found 310.1379 (see spectrum)

Synthesis of 2-(4-butyltetrahydro-2H-4λ^4,8λ^4-[1,3,2]oxazaborolo[2,3-*b*][1,3,2]oxazaborol-8-yl)-5-(trifluoromethyl)benzaldehyde **IX-B(BDEA)**

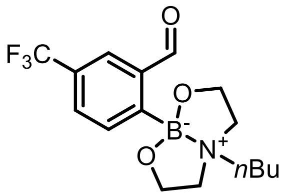

IX-B(BDEA)

Prepared according to general procedure A (GP-A) using (2-formyl-4-(trifluoromethyl)phenyl)boronic acid (218 mg, 1.0 mmol, 1.0 equiv.) N-butyldiethanolamine (161 mg, 1.0 mmol, 1.0 equiv) and toluene (4 mL) to obtain the title compound **IX-B(BDEA)** as a white amorphous solid (325 mg, 95% yield). ^1H NMR (400 MHz, Acetone-*d*$_6$) δ 10.92 (s, 1H), 8.10 (d, J = 7.9 Hz, 1H), 8.06 – 7.97 (m, 1H), 7.77 (ddd, J = 7.9, 2.1, 0.8 Hz, 1H), 4.26 – 4.04 (m, 4H), 3.59 – 3.29 (m, 4H), 2.67 – 2.35 (m, 2H), 1.84 – 1.58 (m, 2H), 1.11 (h, J = 7.4 Hz, 2H), 0.78 (t, J = 7.4 Hz, 3H). ^{13}C NMR (101 MHz, Acetone) δ 196.1, 143.6, 138.1, 130.1 (q, J = 32.2 Hz), 128.2 (q, J = 3.6 Hz), 125.4 (q, J = 271.0 Hz), 123.1 (q, J = 4.0 Hz), 64.3, 61.5, 59.0, 27.6, 20.7, 13.9. ^{11}B NMR (128 MHz, Acetone-*d*$_6$) δ 12.17. HRMS (ESI), $[M+H]^+$: Calculated for $[C_{16}H_{22}BF_3NO_3]^+$ 344.1639, Found 344.1641. (see spectrum)

Synthesis of 2-(4-butyltetrahydro-2H-4λ^4,8λ^4-[1,3,2]oxazaborolo[2,3-*b*][1,3,2]oxazaborol-8-yl)-4-(trifluoromethyl)benzaldehyde **X-B(BDEA)**

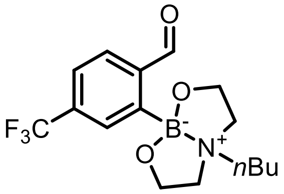

X-B(BDEA)

Prepared according to general procedure A (GP-A) using (2-formyl-5-(trifluoromethyl)phenyl)boronic acid (78mg, 0.35 mmol, 1.0 equiv.) N-butyldiethanolamine (58 mg, 0.35 mmol, 1.0 equiv) and toluene (1,5 mL) to obtain the title compound **X-B(BDEA)** as a white amorphous solid (109 mg, **89% yield**). ^1H NMR (400 MHz, Acetone-*d*$_6$) δ 10.65 (s, 1H), 7.94 (s, 1H), 7.65 (d, J = 8.1 Hz, 1H), 7.42 (d, J = 8.1 Hz, 1H), 4.03 – 3.68 (m, 4H), 3.21 – 3.00 (m, 4H), 2.26 – 2.10 (m, 2H), 1.54 – 1.30 (m, 2H), 0.85 (h, J = 7.5 Hz, 2H), 0.52 (t, J = 7.4 Hz, 3H). ^{13}C NMR (101 MHz, Acetone-*d*$_6$) δ 196.5, 146.1, 133.4 (q, J = 3.8 Hz), 133.0 (q, J = 32.0 Hz) 125.6 (q, J = 272.0 Hz), 125.1 (q, J = 3.8 Hz), 64.4, 61.6, 59.1, 27.6, 20.7, 13.9. ^{11}B NMR (128 MHz, Acetone-*d*$_6$) δ 12.3. ^{19}F NMR (376 MHz, Acetone-*d*$_6$) δ -63.3. HRMS (ESI), $[M+H]^+$: Calculated for $[C_{16}H_{22}BF_3NO_3]^+$ 344.1639, Found 344.1643 (see spectrum)

2.3.2 Synthesis of 3-difluoromethylated benzoxaboroles

Synthesis of 3-(difluoromethyl)benzo[*c*][1,2]oxaborol-1(3*H*)-ol **1**

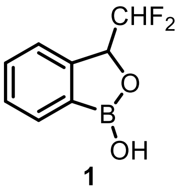

1

Prepared according to general procedure B (GP-B) using **I-B(BDEA)** complex (69 mg, 0.25 mmol, 1.0 equiv.), Me_3SiCF_2H (71 μL, 0.50 mmol, 2.0 equiv.), KO*t*Bu (1.0M in THF, 0.50 mL, 0.50 mmol, 2.0 equiv) and DME (0.50 mL). The obtained crude is purified on flash column chromatography (Hex:AcOEt 7:3 + 1.0% formic acid) to obtain the title compound **1** as a white crystalline solid (33 mg, **73% yield**). The same reaction was repeated in gram scale using **IB-BDEA** complex (1000 mg, 3.63 mmol, 1.0 equiv.), Me_3SiCF_2H (1.03 mL, 7.27 mmol, 2.0 equiv.), KO*t*Bu (1.0M in THF, 7.27 mL, 7.27 mmol, 2.0 equiv) and DME (7.27 mL), obtaining the title compound **1** as a white crystalline solid (436 mg, **65% yield**). ^1H NMR (400 MHz,

$CDCl_3$) δ 7.77 (d, J = 7.3 Hz, 1H), 7.59 – 7.53 (m, 1H), 7.52 (d, J = 6.6 Hz, 1H), 7.47 (td, J = 7.0, 1.9 Hz, 1H), 5.74 (td, J = 55.6, 4.6 Hz, 1H), 5.29 (ddd, J = 10.7, 9.2, 4.6 Hz, 1H), 4.83 (bs, 1H). ^{13}C NMR (101 MHz, $CDCl_3$) δ 149.2, 131.8, 131.0, 129.0, 123.1 (d, J = 2.9 Hz), 115.1 (dd, J = 246.4, 244.2 Hz), 79.65 (dd, J = 27.7, 25.1 Hz). ^{11}B NMR (128 MHz, $CDCl_3$) δ 32.38. ^{19}F NMR (376 MHz, $CDCl_3$) δ -124.61 (ddd, J = 291.6, 55.9, 9.2 Hz), -129.39 (ddd, J = 291.8, 55.2, 10.7 Hz). HRMS (ESI), m/z [M-H]$^-$: Calculated for $[C_8H_6BF_2O_2]^-$ 183.0434, Found 183.0438 (see spectrum) (see HPLC)

Synthesis of 3-(difluoromethyl)-6-methoxybenzo[*c*][1,2]oxaborol-1(*3H*)-ol **2**

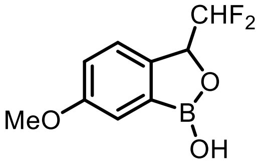

2

Prepared according to general procedure B (GP-B) using **II-B(BDEA)** complex (76 mg, 0.25 mmol, 1.0 equiv.), Me_3SiCF_2H (71 µL, 0.50 mmol, 2.0 equiv.), KO*t*Bu (1.0M in THF, 0.50 mL, 0.50 mmol, 2.0 equiv) and DME (0.50 mL). The obtained crude is purified on flash column chromatography (Hex:AcOEt 7:3 + 1.0% formic acid) to obtain the title compound **2** as a white crystalline solid (44 mg, **81% yield**). ^{1}H NMR (400 MHz, $CDCl_3$) δ 7.41 (d, J = 8.3 Hz, 1H), 7.25 (d, J = 2.5 Hz, 1H), 7.11 (dd, J = 8.4, 2.5 Hz, 1H), 5.69 (td, J = 56.0, 4.8 Hz, 1H), 5.30 (bs, 1H), 5.24 (td, J = 9.5, 4.7 Hz, 1H), 3.85 (s, 3H). ^{13}C NMR (101 MHz, $CDCl_3$) δ 160.2, 141.1, 123.8, 119.4, 115.0 (t, J = 246.7 Hz), 113.4, 79.0 (t, J = 25.8 Hz), 55.3. ^{11}B NMR (128 MHz, $CDCl_3$) δ 32.33. ^{19}F NMR (376 MHz, $CDCl_3$) δ -124.46 (ddd, J = 291.4, 56.1, 9.0 Hz), -129.45 (ddd, J = 291.4, 55.5, 10.3 Hz). HRMS (ESI), m/z [M-H]$^-$: Calculated for $[C_9H_8BF_2O_3]^-$ 213.0540, Found 213.0534 (see spectrum)

Synthesis of 3-(difluoromethyl)-6-methylbenzo[*c*][1,2]oxaborol-1(*3H*)-ol **3**

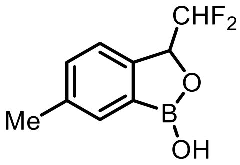

3

Prepared according to general procedure B (GP-B) using **III-B(BDEA)** complex (72 mg, 0.25 mmol, 1.0 equiv.), Me_3SiCF_2H (71 µL, 0.50 mmol, 2.0 equiv.), KO*t*Bu (1.0M in THF, 0.50 mL, 0.50 mmol, 2.0 equiv) and DME (0.50 mL). The obtained crude is purified on flash column chromatography (Hex:AcOEt 7:3 + 1.0% formic acid) to obtain the title compound **3** as a white crystalline solid (35 mg, **70% yield**). ^{1}H NMR (400 MHz, $CDCl_3$) δ 7.59 (s, 1H), 7.41 (d, J = 8.0 Hz, 1H), 7.36 (dd, J = 7.9, 1.6 Hz, 1H), 5.71 (dt, J = 56.0, 4.1 Hz, 1H), 5.27 (dt, J = 9.7, 4.6 Hz, 1H), 2.42 (s, 3H). ^{13}C NMR (151 MHz, $CDCl_3$) δ 146.4, 138.8, 132.9, 131.2, 122.8 (d, J = 2.8 Hz), 115.2 (dd, J = 246.6, 244.1 Hz), 79.5 (dd, J = 27.8, 25.0 Hz), 21.4. ^{11}B NMR (128 MHz, $CDCl_3$) δ 32.5. ^{19}F NMR (376 MHz, $CDCl_3$) δ -124.57 (d, J = 291.3 Hz), -129.42 (d, J = 291.4 Hz). HRMS (ESI), m/z [M-H]$^-$: Calculated for $[C_9H_8BF_2O_2]^-$ 197.0591, Found 197.0585 (see spectrum)

Synthesis of 3-(difluoromethyl)-5-fluorobenzo[*c*][1,2]oxaborol-1(*3H*)-ol **4**

Prepared according to general procedure B (GP-B) using **IV-B(BDEA)** complex (73 mg, 0.25 mmol, 1.0 equiv.), Me_3SiCF_2H (71 µL, 0.50 mmol, 2.0 equiv.), KO*t*Bu (1.0M in THF, 0.50 mL, 0.50 mmol, 2.0 equiv) and DME (0.50 mL). The obtained crude is purified on flash column chromatography (Hex:Et_2O

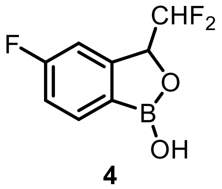
4

1:1 + 1.0% formic acid) to obtain the title compound **4** as a white crystalline solid (24 mg, **48% yield**). ^{1}H NMR (400 MHz, $CDCl_3$) δ 7.75 (dd, J = 8.1, 5.5 Hz, 1H), 7.21 (d, J = 8.7 Hz, 1H), 7.19 – 7.14 (m, 1H), 5.76 (td, J = 55.6, 4.7 Hz, 1H), 5.30 (bs, 1H), 5.26 (td, J = 8.0, 4.1 Hz, 1H). ^{13}C NMR (101 MHz, $CDCl_3$) δ 165.5 (d, J = 251.8 Hz), 151.7, 132.9 (d, J = 9.3 Hz), 117.0 (d, J = 22.2 Hz), 114.8 (t, J = 245.5 Hz), 110.6 (d, J = 23.3 Hz), 79.1 (t, J = 26.8 Hz). ^{11}B NMR (128 MHz, $CDCl_3$) δ 31.95. ^{19}F NMR (376 MHz, $CDCl_3$) δ -107.21, -123.89 – -126.18 (m), -128.69 – -131.11 (m). HRMS (ESI), m/z $[M-H]^-$: Calculated for $[C_8H_5BF_3O_2]^-$ 201.0340, Found 201.0336 (see spectrum)

Synthesis of 3-(difluoromethyl)-6-fluorobenzo[*c*][1,2]oxaborol-1(*3H)*-ol **5**

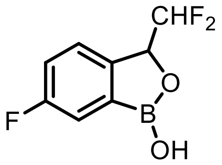
5

Prepared according to general procedure B (GP-B) using **V-B(BDEA)** complex (73 mg, 0.25 mmol, 1.0 equiv.), Me_3SiCF_2H (71 µL, 0.50 mmol, 2.0 equiv.), KO*t*Bu (1.0M in THF, 0.50 mL, 0.50 mmol, 2.0 equiv) and DME (0.50 mL). The obtained crude is purified on flash column chromatography (Hex:AcOEt 7:3 + 1.0% formic acid) to obtain the title compound **5** as a white crystalline solid (24 mg, **48% yield**). ^{1}H NMR (400 MHz, $CDCl_3$) δ 7.48 (dd, J = 8.6, 4.4 Hz, 1H), 7.43 (dd, J = 7.8, 2.4 Hz, 1H), 7.24 (td, J = 8.7, 3.0 Hz, 1H), 5.73 (td, J = 56.2, 4.4 Hz, 1H), 5.30 (bs, 1H), 5.27 (ddd, J = 12.3, 8.6, 4.6 Hz, 1H). ^{13}C NMR (101 MHz, $CDCl_3$) δ 163.6 (d, J = 248.1 Hz), 144.5, 124.9 (d, J = 8.4 Hz), 119.5 (d, J = 23.6 Hz), 117.0 (d, J = 20.9 Hz), 114.9 (t, J = 245.5 Hz), 79.3 (t, J = 26.8 Hz). ^{11}B NMR (128 MHz, $CDCl_3$) δ 31.98. ^{19}F NMR (376 MHz, $CDCl_3$) δ -113.32, -124.76 (d, J = 292.4 Hz), -129.91 (d, J = 292.4 Hz). HRMS (ESI), m/z $[M-H]^-$: Calculated for $[C_8H_5BF_3O_2]^-$ 201.0340, Found 201.0337. (see spectrum)

Synthesis of 3-(difluoromethyl)-5-methoxybenzo[*c*][1,2]oxaborol-1(*3H*)-ol **6**

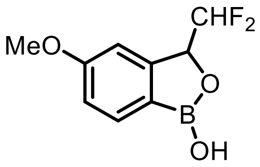
6

Prepared according to general procedure B (GP-B) using **VI-B(BDEA)** complex (76 mg, 0.25 mmol, 1.0 equiv.), Me_3SiCF_2H (71 µL, 0.50 mmol, 2.0 equiv.), KO*t*Bu (1.0M in THF, 0.50 mL, 0.50 mmol, 2.0 equiv) and DME (0.50 mL). The obtained crude is purified on flash column chromatography (Hex:Et_2O 7:3 + 1.0% formic acid) to obtain the title compound **6** as a white crystalline solid (37 mg, **69% yield**). ^{1}H NMR (400 MHz, $CDCl_3$) δ 7.41 (d, J = 8.3 Hz, 1H), 7.25 (d, J = 2.5 Hz, 1H), 7.11 (dd, J = 8.4, 2.5 Hz, 1H), 5.69 (td, J = 56.0, 4.8 Hz, 1H), 5.54 (bs, 1H), 5.24 (td, J = 9.5, 4.7 Hz, 1H), 3.85 (s, 3H). ^{13}C NMR (101 MHz, $CDCl_3$) δ 160.2, 141.1, 123.8, 119.4, 115.0 (t, J = 246.7 Hz), 113.4, 79.0 (t, J = 25.8 Hz), 55.3. ^{11}B NMR (128 MHz, $CDCl_3$) δ 32.33. ^{19}F NMR (376 MHz, $CDCl_3$) δ -124.46 (ddd, J = 291.4, 56.1, 9.0 Hz), -129.45 (ddd, J = 291.4, 55.5, 10.3 Hz). HRMS (ESI), m/z $[M-H]^-$: Calculated for $[C_9H_8BF_2O_3]^-$ 213.0540, Found 213.0534. (see spectrum)

Synthesis of 5-chloro-3-(difluoromethyl)benzo[*c*][1,2]oxaborol-1(*3H*)-ol **7**

Prepared according to general procedure B (GP-B) using **VII-B(BDEA)** complex (77 mg, 0.25 mmol, 1.0 equiv.), Me_3SiCF_2H (71 µL, 0.50 mmol, 2.0 equiv.), KO*t*Bu (1.0M in THF, 0.50 mL, 0.50 mmol, 2.0

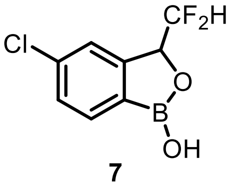

7

equiv) and DME (0.50 mL). The obtained crude is purified on flash column chromatography (Hex:AcOEt 6:4 + 1.0% formic acid) to obtain the title compound **7** as a white crystalline solid (24 mg, **45% yield**). ^{1}H NMR (400 MHz, $CDCl_3$) δ 7.70 (d, J = 7.9 Hz, 1H), 7.51 (s, 1H), 7.45 (d, J = 7.9 Hz, 1H), 5.76 (td, J = 54.5, 3.6 Hz, 1H), 5.30 (bs, 1H), 5.26 (td, J = 10.4, 4.4 Hz, 1H). ^{13}C NMR (101 MHz, $CDCl_3$) δ 150.9, 138.5, 132.1, 129.7, 123.6, 114.7 (t, J = 245.7 Hz), 79.2 (t, J = 26.9 Hz). ^{11}B NMR (128 MHz, $CDCl_3$) δ 32.3. ^{19}F NMR (282 MHz, $CDCl_3$) δ -124.79 (ddd, J = 293.0, 55.4, 8.4 Hz), -129.88 (ddd, J = 293.0, 55.4, 11.6 Hz). HRMS (ESI), m/z $[M-H]^-$: Calculated for $[C_8H_5BClF_2O_2]^-$ 217.0045, Found 217.0035 (see spectrum)

Synthesis of 6-chloro-3-(difluoromethyl)benzo[*c*][1,2]oxaborol-1(*3H*)-ol **8**

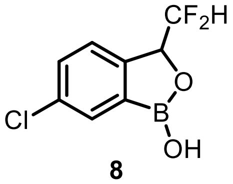

8

Prepared according to general procedure B (GP-B) using **VIII-B(BDEA)** complex (77 mg, 0.25 mmol, 1.0 equiv.), Me_3SiCF_2H (71 μL, 0.50 mmol, 2.0 equiv.), KO*t*Bu (1.0M in THF, 0.50 mL, 0.50 mmol, 2.0 equiv) and DME (0.50 mL). The obtained crude is purified on flash column chromatography (Hex:AcOEt 7:3 + 1.0% formic acid) to obtain the title compound **8** as a white crystalline solid (32 mg, **55% yield**). ^{1}H NMR (400 MHz, $CDCl_3$) δ 7.74 (s, 1H), 7.51 (dd, J = 8.2, 2.0 Hz, 1H), 7.45 (d, J = 8.3 Hz, 1H), 5.75 (td, J = 55.6, 4.3 Hz, 1H), 5.40 (bs, 1H), 5.27 (ddd, J = 12.1, 8.4, 4.0 Hz, 1H). ^{13}C NMR (101 MHz, $CDCl_3$) δ 147.3, 135.6, 132.1, 130.9, 124.5, 114.8 (t, J = 245.6 Hz), 79.4 (t, J = 26.9 Hz). ^{11}B NMR (128 MHz, $CDCl_3$) δ 31.8. ^{19}F NMR (282 MHz, CDCl3) δ -124.76 (ddd, J = 292.8, 55.5, 8.5 Hz), -129.99 (ddd, J = 292.6, 55.5, 11.4 Hz). HRMS (ESI), m/z $[M-H]^-$: Calculated for $[C_8H_5BClF_2O_2]^-$ 217.0045, Found 217.0037 (see spectrum)

Synthesis of 3-(difluoromethyl)-5-(trifluoromethyl)benzo[*c*][1,2]oxaborol-1(*3H*)-ol **9**

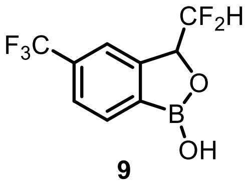

9

Prepared according to general procedure B (GP-B) using **IX-B(BDEA)** complex (86 mg, 0.25 mmol, 1.0 equiv.), Me_3SiCF_2H (71 μL, 0.50 mmol, 2.0 equiv.), KO*t*Bu (1.0M in THF, 0.50 mL, 0.50 mmol, 2.0 equiv) and DME (0.50 mL). The obtained crude is purified on flash column chromatography (Hex:AcOEt 1:1 + 1.0% formic acid) to obtain the title compound **9** as a white crystalline solid (40 mg, **63% yield**). ^{1}H NMR (400 MHz, $CDCl_3$) δ 7.91 (d, J = 7.7 Hz, 1H), 7.77 (s, 1H), 7.74 (d, J = 7.6 Hz, 1H), 5.82 (td, J = 55.7, 4.5 Hz, 1H), 5.36 (ddd, J = 11.9, 7.8, 4.1 Hz, 1H). ^{13}C NMR (101 MHz, $CDCl_3$) δ 149.7, 131.6, 130.8 (q, J = 32.0 Hz),126.1 (q, J = 3.5 Hz), 125.4 (q, J = 276.3 Hz), 120.0 (q, J = 4.3 Hz), 114.6 (t, J = 245.8 Hz), 79.6 (t, J = 26.9 Hz). ^{11}B NMR (128 MHz, $CDCl_3$) δ 31.9. ^{19}F NMR (282 MHz, $CDCl_3$) δ -62.70, -124.99 (ddd, J = 293.6, 55.2, 8.3 Hz), -130.03 (ddd, J = 293.5, 55.3, 11.8 Hz). HRMS (ESI), m/z $[M-H]^-$: Calculated for $[C_9H_5BF_5O_2]^-$ 251.0308, Found 251.0304 (see spectrum)

Synthesis of 3-(difluoromethyl)-6-(trifluoromethyl)benzo[*c*][1,2]oxaborol-1(*3H*)-ol **10**

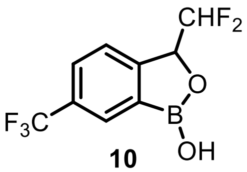

10

Prepared according to general procedure B (GP-B) using **X-B(BDEA)** complex (86 mg, 0.25 mmol, 1.0 equiv.), Me_3SiCF_2H (71 µL, 0.50 mmol, 2.0 equiv.), KO*t*Bu (1.0M in THF, 0.50 mL, 0.50 mmol, 2.0 equiv) and DME (0.50 mL). The obtained crude is purified on flash column chromatography (Hex:AcOEt 1:1 + 1.0% formic acid) to obtain the title compound **10** as a white crystalline solid (11 mg, **24% yield.** ^{1}H NMR (400 MHz, $CDCl_3$) δ 8.05 (s, 1H), 7.80 (d, J = 8.1 Hz, 1H), 7.65 (d, J = 8.1 Hz, 1H), 5.81 (td, J = 55.5, 4.4 Hz, 1H), 5.43 – 5.27 (m, 1H), 5.15 (bs, 1H). ^{13}C NMR (101 MHz, $CDCl_3$) δ 150.4, 131.6, 131.4 (q, J = 29.0 Hz), 128.7 (q, J = 3.5 Hz), 125.4 (q, J = 276.3 Hz), 128.0 (q, J = 3.3 Hz), 114.6 (t, J = 246.1 Hz), 79.6 (t, J = 27.0 Hz). ^{11}B NMR (128 MHz, $CDCl_3$) δ 31.8. ^{19}F NMR (376 MHz, $CDCl_3$) δ -62.43, -124.94 (ddd, J = 292.6, 55.2, 7.9 Hz), -130.13 (ddd, J = 292.8, 55.5, 12.3 Hz). HRMS (ESI), m/z $[M-H]^-$: Calculated for $[C_9H_5BF_5O_2]^-$ 251.0308, Found 251.0310 (see spectrum)

2.3.3 Synthesis of 3-difluromethyl Crisaborole analogue

Synthesis of 4-(4-bromo-3-formylphenoxy)benzonitrile **I-C**

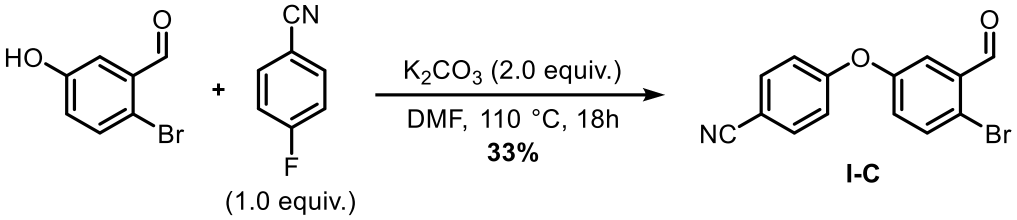

The procedure has been adapted from the literature.[4] A 30 mL Schlenk tube equipped with a stir bar was dried with Schlenk technique. Under N_2 atmosphere, 2-bromo-5-hydroxybenzaldehyde (1660 mg, 8.26 mmol, 1.0 equiv.) and 4-fluorobenzonitrile (1000 mg, 8.26 mmol, 1.0 equiv) were added. Then, K_2CO_3 (2282 mg, 16.51 mmol, 2.0 equiv.), previously dried in oven, was added. The solids were dissolved in dry DMF (10.7 mL, 0.77 M) and the reaction was stirred at 110 °C in an oil bath for 18 hours. Then, reaction is concentrated un vacuo and diluted whit AcOEt/H_2O. the two phases were separated and the aqueous phase is extracted 3 times with AcOEt. Organic phases are collected, dried over Na_2SO_4, filtered and concentrated under reduced pressure. The obtained crude is purified on flash chromatography column (100% DCM) to provide the compound **I-C** as white crystalline solid (815 mg, **33% yield**). ^{1}H NMR (400 MHz, $CDCl_3$) δ 10.30 (s, 1H), 7.69 (dd, J = 8.6, 1.0 Hz, 1H), 7.64 (d, J = 8.9 Hz, 2H), 7.56 (dd, J = 3.2, 1.3 Hz, 1H), 7.19 (ddd, J = 8.7, 3.1, 0.8 Hz, 1H), 7.04 (d, J = 9.0 Hz, 2H). (see spectrum). The data are in agreement with those reported in the literature.[4]

Synthesis of 4-(3-formyl-4-(4,4,5,5-tetramethyl-1,3,2-dioxaborolan-2-yl)phenoxy)benzonitrile **II-C**

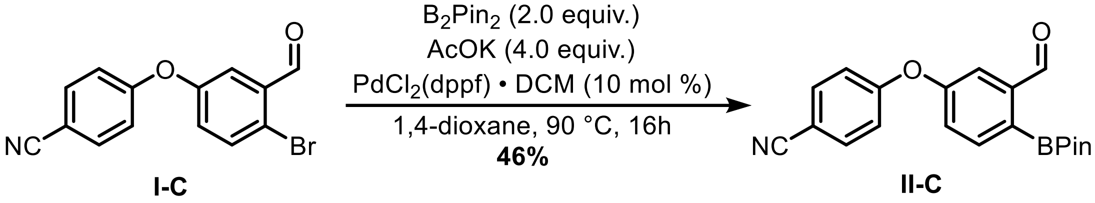

The procedure has been adapted from the literature.[4] A 30 mL Schlenk tube equipped with a stir bar was dried with Schlenk technique. Under N_2 atmosphere, **I-C** (811 mg, 2.68 mmol, 1,0 equiv.) was added, followed by with B_2Pin_2 (1363 mg, 5.37 mmol, 2.0 equiv.), previously dried under vacuum for 2 hours. Then AcOK (previously dried in oven for 24h ,1054 mg, 10.74 mmol, 4,0 equiv.), and $PdCl_2$(dppf) (219 mg, 0.27 mmol, 10 mol%) were added to the Schlenk tube. Solids were subjected to three rapid cycles of vacuum/nitrogen backfill and then dissolved in previously degassed 1,4-dioxane (15 mL, 0.18 M). The reaction was stirred at 90 °C in an oil bath for 16 hours. The reaction mixture was filtered on celite with DCM and then concentrated under reduced pressure. The obtained crude is purified on flash chromatography column (Hex:AcOEt 7:3 + 1.0% formic acid) to obtain the compound **II-C** as a pale yellow solid (431 mg, **46% yield**). ^{1}H NMR (400 MHz, $CDCl_3$) δ 10.63 (s, 1H), 7.98 (d, J = 8.2 Hz, 1H), 7.64 (d, J = 8.9 Hz, 2H), 7.61 (d, J = 2.5 Hz, 1H), 7.28 (dd, J = 8.2, 2.5 Hz, 1H), 7.05 (d, J = 8.9 Hz, 2H), 1.39 (s, 12H). ^{11}B NMR (128 MHz, $CDCl_3$) δ 30.50. (see spectrum). The data are in agreement with those reported in the literature.[4]

Synthesis of (4-(4-cyanophenoxy)-2-formylphenyl)boronic acid **III-C**

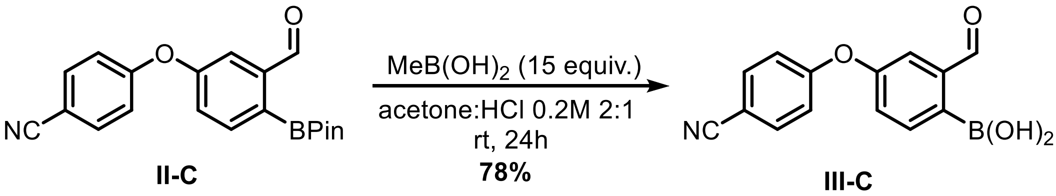

The procedure has been adapted from the literature.[4] A 50 mL round bottom flask equipped with a stir bar was charged with **II-C** (250 mg, 0.72 mmol, 1 equiv.) and $MeB(OH)_2$ (643 mg, 10.74 mmol, 15 equiv.). Then a 2:1 mixture of acetone:HCl 0.2 M (5.4 mL, 0.1 M) was added to reaction flask and the reaction was stirred for 24 hours at room temperature. Then, reaction mixture was concentrated under reduced pressure and the obtained crude was purified on flash chromatography column (Hex:AcOEt 7:3 + 1.0% formic acid) to obtain the compound **III-C** as a brown solid (150 mg, **78% yield**). 1H NMR (400 MHz, CD_3OD) δ 8.47 (s, 1H), 7.69 (d, J = 8.3 Hz, 2H), 7.54 (s, 1H), 7.07 (d, J = 8.5 Hz, 2H), 7.04 – 6.93 (m, 2H). The additional signal at 5.91 ppm (s, 1H) refers to the hemiacetalic form in equilibrium with the titled compound III-C. ^{11}B NMR (128 MHz, CD_3OD) δ 28.27. (see spectrum). The data are in agreement with those reported in the literature.[4]

Synthesis of 4-(4-(4-butyltetrahydro-2H-4l4,8l4-[1,3,2]oxazaborolo[2,3-b][1,3,2]oxazaborol-8-yl)-3-formylphenoxy)benzonitrile **XI-B(BDEA)**

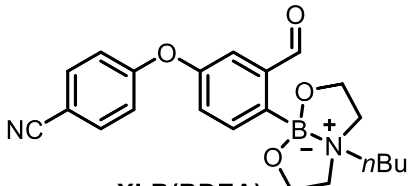

XI-B(BDEA)

Prepared according to general procedure A (GP-A) using **III-C** (149 mg, 0.56 mmol, 1.0 equiv.) N-butyldiethanolamine (90 mg, 0.56 mmol, 1.0 equiv) and toluene (2.2 mL) to obtain the title compound **XI-B(BDEA)** as a white amorphous solid (290 mg, **94% yield**). ^{1}H NMR (400 MHz, Acetone-d_6) δ 10.90 (s, 1H), 7.98 (d, J = 8.3 Hz, 1H), 7.77 (d, J = 8.6 Hz, 2H), 7.47 (d, J = 2.6 Hz, 1H), 7.26 (dd, J = 8.3, 2.6 Hz, 1H), 7.13 (d, J = 8.5 Hz, 2H), 4.29 – 4.03 (m, 4H), 3.44 – 3.34 (m, 2H), 3.30 (td, J = 11.1, 7.5 Hz, 2H), 2.55 – 2.36 (m, 2H), 1.66 (ddd, J = 11.9, 9.9, 6.3 Hz, 2H), 1.15 (h, J = 7.5 Hz, 2H), 0.81 (t, J = 7.4 Hz, 3H).^{13}C NMR (101 MHz, Acetone-d_6) δ 196.3, 162.3, 155.8, 145.1, 139.5, 135.3, 135.1, 124.3, 119.2, 118.8, 117.3, 64.2, 61.5, 58.8, 27.6, 20.8, 14.0.^{11}B NMR (128 MHz, Acetone-d_6) δ 12.58. HRMS (ESI), m/z $[M+H]^+$: Calculated for $[C_{22}H_{26}BN_2O_4]^+$ 393.1980, Found 393.1981. (see spectrum)

Synthesis of 4-((3-(difluoromethyl)-1-hydroxy-1,3-dihydrobenzo[c][1,2]oxaborol-5-yl)oxy)benzonitrile **11**

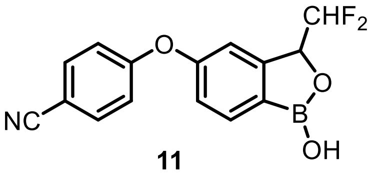

11

Prepared according to general procedure B (GP-B) using **XI-B(BDEA)** complex (98 mg, 0.25 mmol, 1.0 equiv.), Me_3SiCF_2H (71 µL, 0.50 mmol, 2.0 equiv.), KO*t*Bu (1.0M in THF, 0.50 mL, 0.50 mmol, 2.0 equiv) and DME (0.50 mL). The obtained crude is purified on flash column chromatography (Hex:AcOEt 1:1 + 1.0% formic acid) to obtain the title compound **11** as a white crystalline solid (34 mg, **45% yield**). ^{1}H NMR (400 MHz, $CDCl_3$) δ 7.81 (d, J = 8.0 Hz, 1H), 7.65 (d, J = 8.8 Hz, 2H), 7.18 (s, 1H), 7.17 – 7.11 (m, 1H), 7.07 (d, J = 8.7 Hz, 2H), 5.78 (td, J = 54.9, 4.3 Hz, 1H), 5.52 (bs, 1H), 5.27 (ddd, J = 12.3, 8.4, 4.4 Hz, 1H).^{13}C NMR (101 MHz, $CDCl_3$) δ 160.7, 158.5, 151.8, 134.5, 133.0, 120.9, 118.9, 118.7, 114.75 (t, J = 245.5 Hz), 114.5, 106.9, 79.15 (t, J = 26.7 Hz).^{11}B NMR (128 MHz, $CDCl_3$) δ 31.9. ^{19}F NMR (376 MHz, $CDCl_3$) δ -124.82 (ddd, J = 292.5, 55.2, 8.6 Hz), -130.09 (ddd, J = 293.0, 55.9, 11.9 Hz). HRMS (ESI) m/z $[M-H]^-$: Calculated for $[C_{15}H_9BF_2NO_3]^-$ 300.0649, Found 300.0650. (see spectrum)

2.3.4 Functionalisation of 3-difluoromethyl benzoxaboroles

Synthesis of 3-(difluoromethyl)-6-nitrobenzo[c][1,2]oxaborol-1(3H)-ol **12**

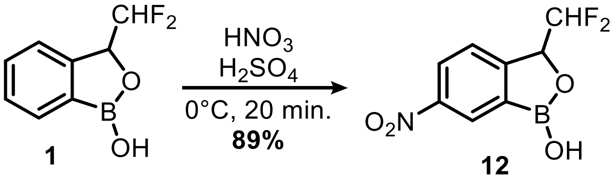

1 **12**

A 10 mL single necked round bottom flask equipped with a stir bar is brought to 0°C and charged with

H_2SO_4 (98%, 1mL) and HNO_3 (65%, 1 mL). 3-(difluoromethyl)benzo[c][1,2]oxaborol-1(3H)-ol **1** (50 mg, 0.27 mmol, 1.0 equiv.) is added. The reaction is left stirr and monitored via TLC. After 20 minutes reaction is quenched with ice and water, and diluted with AcOEt. The two phases are separated, and aqueous phase is extracted three times with AcOEt. Organic phases are collected, dried over Na_2SO_4, filtered and concentrated under reduced pressure. The obtained crude is purified on flash chromatography column (from Hex:AcOEt 9:1 + 1.0% formic acid to Hex:AcOEt 7:3 + 1.0% formic acid) to provide the title compound **12** as yellow amorphous solid (55 mg, **89% yield**). ^1H NMR (400 MHz, Acetone-d_6) δ 8.88 (bs, 1H), 8.61 (d, J = 2.2 Hz, 1H), 8.42 (dd, J = 8.4, 2.2 Hz, 1H), 7.83 (d, J = 8.4 Hz, 1H), 6.22 (td, J = 54.8, 3.2 Hz, 1H), 5.58 (ddd, J = 13.0, 10.2, 3.4 Hz, 1H). ^{13}C NMR (101 MHz, Acetone-d_6) δ 156.4, 149.8, 127.0, 126.3, 125.2, 115.7 (t, J = 243.6 Hz), 79.8 (t, J = 25.5 Hz). ^{11}B NMR (128 MHz, Acetone-d_6) δ 31.6. ^{19}F NMR (376 MHz, $CDCl_3$) δ -125.12 (d, J = 294.1 Hz), -130.58 (d, J = 294.0 Hz). HRMS (ESI), m/z $[M-H]^-$: Calculated for $[C_8H_5BF_2NO_4]^-$ 228.0285, Found 228.0290 (see spectrum)

Synthesis of 3-(difluoromethyl)-1-hydroxy-1,3-dihydrobenzo[c][1,2]oxaborol-6-aminium chloride **13**

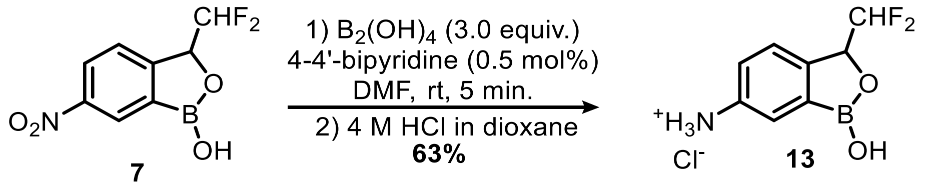

The procedure has been adapted from the literature.[5] A 10 mL single necked round bottom flask equipped with a stir bar is charged with 3-(difluoromethyl)-6-nitrobenzo[c][1,2]oxaborol-1(3H)-ol **7** (115 mg, 0.5 mmol. 1.0 equiv.) and $B_2(OH)_4$ (137 mg, 1.5 mmol, 3.0 equiv.). 4,4-bipyridine solution (2.8 mL of a 0.75 mM solution in DMF, 2.5 µmol of 4,4-bipyridine, 0.5 mol % relative to compound **7**) is added and the reaction is stirred at room temperature for 5 min. After reaction completion, as monitored via TLC, the reaction mixture is concentrated under reduced pressure and diluted with water and AcOEt. The two phases are separated, and aqueous phase is extracted three times with AcOEt. Organic phases are collected, dried over Na_2SO_4, filtered and concentrated under reduced pressure. The obtained crude is treated with 4 M HCl in dioxane (2mL) for 10 minutes. The obtained mixture is concentrated in vacuo and triturated with Et_2O:Hex 1:1 solution. The obtained precipitate is filtered to afford the title compound **13** as a yellow amorphous solid (74 mg, **63% yield**). ^1H NMR (400 MHz, CD_3OD) δ 7.73 – 7.66 (m, 2H), 7.56 (dd, J = 8.3, 2.0 Hz, 1H), 6.03 (td, J = 55.2, 3.0 Hz, 1H), 5.46 (ddd, J = 12.7, 9.6, 3.4 Hz, 1H). ^{13}C NMR (101 MHz, CD_3OD) δ 151.0, 132.8, 126.9, 125.9, 125.6, 116.1 (t, J = 243.9 Hz), 80.6 (t, J = 26.1 Hz). ^{11}B NMR (128 MHz, CD_3OD) δ 30.9. ^{19}F NMR (376 MHz, CD_3OD) δ -129.51 (d, J = 290.6 Hz), -132.50 (d, J = 290.9 Hz). HRMS (ESI), m/z $[M+H]^+$: Calculated for $[C_8H_9BF_2NO_2]^+$ 200.0689, Found 200.0680 (see spectrum)

Synthesis of 6-azido-3-(difluoromethyl)benzo[c][1,2]oxaborol-1(3H)-ol **14**

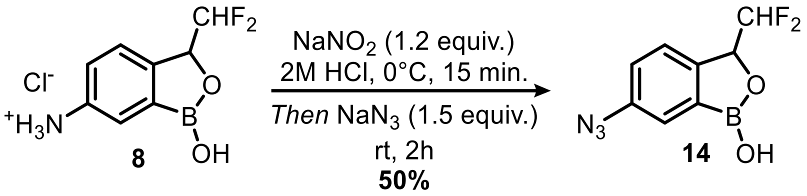

The procedure has been adapted from the literature.[6] A 10 mL single necked round bottom flask equipped with a stir bar is charged with 3-(difluoromethyl)-1-hydroxy-1,3-dihydrobenzo[c][1,2]oxaborol-6-aminium chloride **8** (74 mg, 0.32 mmol, 1.0 equiv) and dissolved in 2 M HCl (3mL, 0.1 M). The flask is brought to 0°C and $NaNO_2$ (26 mg, 0.28 mmol, 1.2 equiv.) is added. Reaction is left stir at 0°C for 15 minutes. Then, NaN_3 (31 mg, 0.47 mmol, 1.5 equiv) is dissolved in 1mL of water and added to the flask. The reaction is brought to room temperature and left to stir for 2 hours. The obtained precipitate is filtered and washed with water to afford the title compound **14** as a yellow amorphous solid (36 mg, **50% yield**). ^{1}H NMR (400 MHz, CD_3OD) δ 7.30 (d, J = 8.2 Hz, 1H), 7.05 (d, J = 2.4 Hz, 1H), 6.95 (dd, J = 8.3, 2.4 Hz, 1H), 5.80 (td, J = 55.4, 4.1 Hz, 1H), 5.21 (td, J = 10.4, 4.1 Hz, 1H). ^{13}C NMR (101 MHz, CD_3OD) δ 159.2, 141.5, 124.8, 120.2, 116.8, 116.8 (t, J = 242.8 Hz), 80.5 (t, J = 25.8 Hz). ^{11}B NMR (128 MHz, CD_3OD) δ 31.5. ^{19}F NMR (376 MHz, CD_3OD) δ -128.42 (d, J = 289.1 Hz), -131.58 (d, J = 288.9 Hz). HRMS (ESI), m/z $[M-H]^-$: Calculated for $[C_8H_5BF_2N_3O_2]^-$ 224.0448, Found 224.0448. (see spectrum)

2.3.5 Enantioenriched synthesis

Synthesis of 2-((3aS,4S,6S,7aR)-3a,5,5-trimethylhexahydro-4,6-methanobenzo[d][1,3,2]dioxaborol-2-yl)benzaldehyde **I-[B$_a$*]**

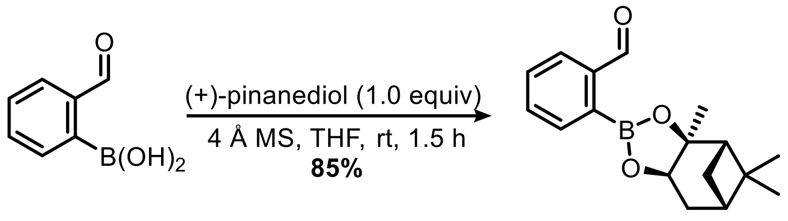

I-B(OH)$_2$ **I-[B$_a$*]**

A 25 mL double necked round bottom flask equipped with a stir bar is dried with Schlenk technique. Under N_2 atmosphere, 2-formyl phenylboronic acid **I-B(OH)$_2$** (300 mg, 2.0 mmol, 2.0 equiv.) and (1S,2S,3R,5S)-(+)-pinanediol (340 mg, 2.0 mmol, 2.0 equiv.) are added and dissolved in THF (4 mL, 0.5M). Activated 4Å molecular sieves are added and the reaction is stirred at rt for 1.5 hour. Then, reaction is filtered and concentrated under reduced pressure. The obtained crude is purified on flash chromatography column (from 100% Hex to Hex:AcOEt 85:15) to provide the title compound **I-[B$_a$*]** as a white amorphous solid (484 mg, **85% yield**). ^{1}H NMR (400 MHz, $CDCl_3$) δ 10.55 (d, J = 0.6 Hz, 1H), 8.00 – 7.93 (m, 1H), 7.93 – 7.85 (m, 1H), 7.63 – 7.53 (m, 2H), 4.53 (dd, J = 8.8, 1.9 Hz, 1H),

2.49 – 2.39 (m, 1H), 2.29 (dtd, J = 11.0, 6.3, 2.5 Hz, 1H), 2.17 (dd, J = 6.0, 5.0 Hz, 1H), 2.05 – 1.93 (m, 2H), 1.54 (s, 3H), 1.33 (m, 4H), 0.91 (s, 3H). ^{13}C NMR (101 MHz, $CDCl_3$) δ 194.8, 141.5, 135.8, 133.1, 130.9, 128.1, 87.0, 78.7, 51.6, 39.8, 38.4, 35.6, 28.8, 27.2, 26.7, 24.2. ^{11}B NMR (128 MHz, $CDCl_3$) δ 30.4. (see spectrum)

Synthesis of (R)-3-(difluoromethyl)benzo[c][1,2]oxaborol-1(3H)-ol **(+)-1**

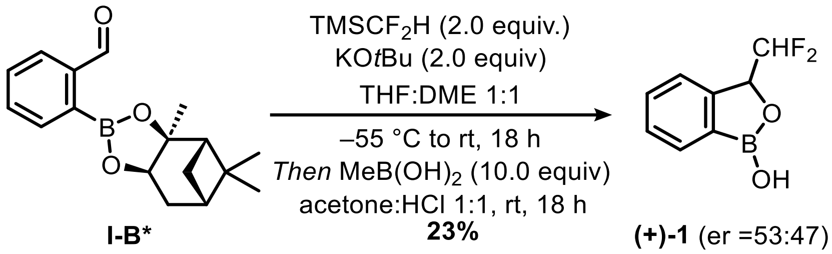

A 10 mL double necked round bottom flask equipped with a stir bar is dried with Schleck technique. Under N_2 atmosphere, **I-B*** (71 mg, 0.25 mmol, 1.0 equiv.) was added and dissolved in DME (0.5 mL, 0.50 M). Then, the mixture is brought to -55°C and Me_3SiCF_2H (71 µL, 0.50 mmol, 2.0 equiv.) was added, followed by KO*t*Bu (1.0 M in THF, 0.50 mL, 2.0 equiv., 0.50 M) and the reaction was stirred from −55°C to rt for 18 hours. After that, the reaction is diluted with AcOEt and 1.0 M HCl (4.0 equiv.). The two phases were separated. The aqueous phase is extracted 3 times with AcOEt. Organic phases are collected, dried over Na_2SO_4, filtered and concentrated under reduced pressure. The obtained crude is transferred to a single necked round bottom flask and charged with $MeB(OH)_2$ (150 mg, 2.5 mmol, 10 equiv.). The crude is dissolved in acetone: 0.2 M HCl 1:1 (2,5 mL, 0.1 M) and the reaction is stirred at rt for 18 hours. Then, the mixture is concentrated under reduced pressure until all the volatiles are removed and a constant weight is obtained. The obtained solid is redissolved in acetone and concentrated under reduced pressure. The obtained crude is purified on flash chromatography column (Hex:AcOEt 7:3 + 1% formic acid) to obtain the title compound **(+)-1** as a white crystalline solid (10 mg, **23% yield**). The enantiomeric ratio is obtained through HPLC analysis using a Lux 5 µm i-Amylose-3 (Phenomenex) column (250 x 4.6 mm) in isocratic conditions of H_2O/ACN 65:35 in 12 min (flux of 1.0 mL/min and sample injection of 25 µL), choosing 254 nm as the wavelength for the detection of compounds. The obtained chromatogram is compared with the one obtained from the racemic mixture. (see HPLC)

Synthesis of (2R,2'R)-1,1'-(n-butylazanediyl)bis(propan-2-ol) ***n*-BDIA**

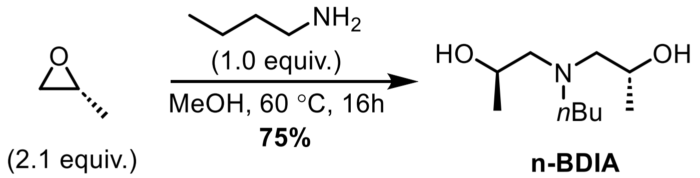

In a 10 mL vial, (R)-propylene oxide (73.2 mg, 1.26 mmol, 2.1 equiv.) was added with n-butylamine

(60 µL, 0.60 mmol, 1,0 equiv.). The reagents were dissolved in MeOH (0.37 mL) and the reaction is stirred at 60°C in an oil bath for 16h. The reaction mixture was concentrated under low pressure. The obtained crude is purified on flash chromatography column (DCM/MeOH 9:1 with $KMnO_4$ as stain) to provide the compound ***n*-BDIA** as yellow oil (85 mg, **75% yield**). ^{1}H NMR (400 MHz, $CDCl_3$) δ 3.76 (dqd, J = 8.2, 6.2, 4.5 Hz, 2H), 3.47 – 3.03 (m, 2H), 2.57 – 2.46 (m, 1H), 2.42 – 2.33 (m, 1H), 2.33 – 2.28 (m, 4H), 1.47 – 1.33 (m, 2H), 1.32 – 1.16 (m, 2H), 1.08 (s, 3H), 1.06 (s, 3H), 0.86 (t, J = 7.3 Hz, 3H). (see spectrum). The data are in agreement with those reported in the literature.[7]

Synthesis of 2-((2R,6R)-4-butyl-2,6-dimethyltetrahydro-2H-4l4,8l4-[1,3,2]oxazaborolo[2,3-b][1,3,2]oxazaborol-8-yl)benzaldehyde **I-[B$_b$*]**

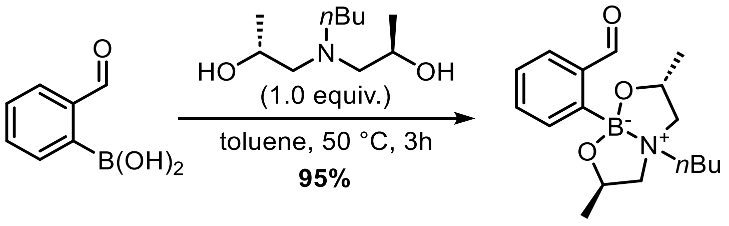

I-[B$_b$*]

Prepared according to general procedure A (GP-A) using 2-formyl phenyl boronic acid (68 mg, 0.45 mmol, 1.0 equiv.) and ***n*-BDIA** (86 mg, 0.45 mmol, 1.0 equiv.) and toluene (1.8 mL) to obtain the title compound **I-[B$_b$*]** as a white amorphous solid (130 mg, **95% yield**). ^{1}H NMR (400 MHz, Acetone-d$_6$) δ 10.94 (d, J = 0.9 Hz, 1H), 7.83 (dd, J = 7.6, 1.4 Hz, 1H), 7.80 (dd, J = 7.7, 1.4 Hz, 1H), 7.48 (td, J = 7.4, 1.4 Hz, 1H), 7.39 – 7.31 (m, 1H), 4.33 (dtd, J = 12.0, 6.0, 3.7 Hz, 1H), 4.28 – 4.11 (m, 1H), 3.49 (dd, J = 12.1, 4.5 Hz, 1H), 3.21 (dd, J = 11.4, 3.6 Hz, 1H), 2.58 (td, J = 10.8, 8.5 Hz, 2H), 2.49 – 2.27 (m, 2H), 1.65 – 1.46 (m, 2H), 1.31 (d, J = 6.1 Hz, 3H), 1.28 (d, J = 5.9 Hz, 3H), 1.13 – 0.96 (m, 2H), 0.74 (t, J = 7.4 Hz, 3H). ^{13}C NMR (101 MHz, Acetone-*d*$_6$) δ 197.0, 143.3, 136.4, 132.6, 128.3, 126.7, 68.7, 68.2, 66.3, 62.5, 58.4, 27.5, 20.7, 20.3, 19.4, 13.9. ^{11}B NMR (128 MHz, Acetone- d$_6$) δ 13.2. HRMS (ESI), m/z $[M+H]^+$: Calculated for $[C_{17}H_{27}BNO_3]^+$ 304.2079, Found 304.2073. (see spectrum)

Synthesis of enantioenriched (R)-3-(difluoromethyl)benzo[c][1,2]oxaborol-1(3H)-ol **(-)-1**

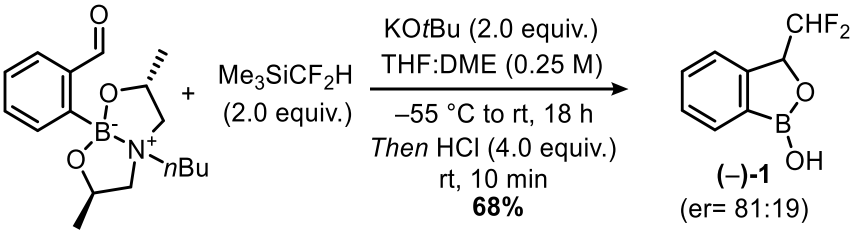

(–)-1
(er= 81:19)

Prepared according to general procedure B (GP-B) using **I-[B$_b$*]** complex (76 mg, 0.25 mmol, 1.0 equiv.), Me_3SiCF_2H (71 µL, 0.50 mmol, 2.0 equiv.), KO*t*Bu (1.0M in THF, 0.50 mL, 0.50 mmol, 2.0 equiv) and DME (0.50 mL). The obtained crude is purified on flash column chromatography (Hex:AcOEt 7:3 + 1.0% formic acid) to obtain the title compound **(-)-1** as a white crystalline solid (31

mg, **68% yield**). The enantiomeric ratio is obtained through HPLC analysis using a Lux 5 μm i-Amylose-3 (Phenomenex) column (250 x 4.6 mm) in isocratic conditions of H_2O/ACN 65:35 in 12 min (flux of 1.0 mL/min and sample injection of 25 μL), choosing 254 nm as the wavelength for the detection of compounds. The obtained chromatogram is compared with the one obtained from the racemic mixture. (see HPLC)

2.3.6 Synthesis of other benzoxaboroles

Synthesis of 3-methylbenzo[c][1,2]oxaborol-1(3H)-ol **15**

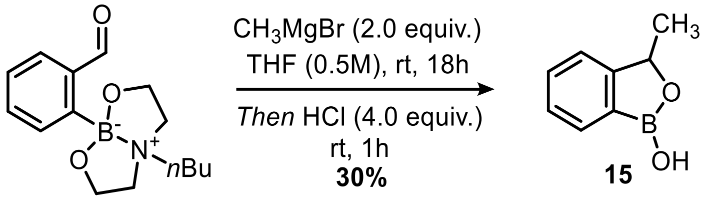

A 10 mL double necked round bottom flask equipped with a stir bar was dried with Schlenk technique. Under N_2 atmosphere, **I-B(BDEA)** complex (69 mg, 0.25 mmol, 1.0 equiv.) was added and dissolved in THF (0.33 mL). Then, CH_3MgBr (3.0M in Et_2O, 0166 mL, 0.50 mmol, 2.0 equiv,) and the reaction was stirred at rt for 18 hours. After that, 1.0M HCl (1mL, 1.0 mmol, 4.0 equiv.) was added and the reaction was stirred at rt for 1 hour. Reaction is diluted with AcOEt and the two phases are separated. The aqueous phase is extracted 3 times with AcOEt. Organic phases are collected, dried over Na_2SO_4, filtered and concentrated under reduced pressure. The obtained crude is purified on flash chromatography column (Hex:AcOEt 7:3 + 1.0% formic acid) to provide the title compound **15** as white crystalline solids (11 mg, **30% yield**). ^{1}H NMR (400 MHz, $CDCl_3$) δ 7.74 (d, J = 7.3 Hz, 1H), 7.49 (td, J = 7.5, 1.2 Hz, 1H), 7.37 (t, J = 7.3 Hz, 1H), 7.30 (dd, J = 7.6, 1.0 Hz, 1H), 5.59 (bs, 1H), 5.35 (q, J = 6.6 Hz, 1H), 1.54 (d, J = 6.7 Hz, 3H). ^{11}B NMR (128 MHz, $CDCl_3$) δ 32.0. (see spectrum). The data are in agreement with those reported in the literature.[8]

2.4 Stability test

An oven dried J Young NMR tube was charged with Compound **1** (5.0 mg, 0.03 mmol, 1.0 equiv.) dissolved in DMSO-*d*$_6$ (0.75 μL, 0.36 M) and an ^{1}H NMR spectrum was acquired after 5 minutes. The capped tube was left at room temperature for one week and, after this time, another ^{1}H-NMR spectrum was acquired. The comparison of the ^{1}H NMR spectra between **1** and **1** after one week confirmed the stability of the title compound in DMSO-*d*$_6$ (see spectrum). ^{1}H NMR (400 MHz, DMSO-*d*$_6$) δ 9.57 (bs, 1H), 7.77 (dt, J = 7.3, 1.1 Hz, 1H), 7.54 (m, 2H), 7.45 (td, J = 7.1, 1.4 Hz, 1H), 6.23 (td, J = 54.7, 3.4 Hz, 1H), 5.41 (td, J = 11.7, 3.4 Hz, 1H). (see spectrum)

2.5 pK_a determination

The pH glass electrode was calibrated with two buffer solutions (pH 4 and 7). A sample of boronic acid (0.03–0.05 g) was dissolved in 0.05 M aqueous KCl solution. After the complete dissolution of the sample, it was titrated with 0.05 M aq. NaOH. The inflection point on the curve corresponding to the neutralization point was determined from the derivative plot calculated as ΔpH/ΔV. The pK_a value was determined from the pH = f(V) curve as the pH value corresponding to the pH value for half the volume of the neutralization point.

2.6 Gutmann-Beckett method measurements

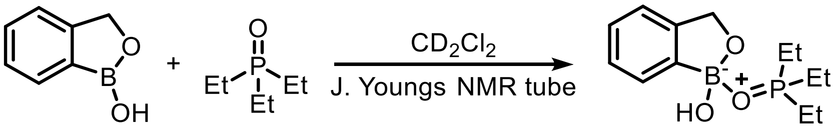

benzo[*c*][1,2]oxaborol-1(3*H*)-ol

The procedure has been adapted from the literature.[9] A J. Youngs NMR tube was loaded with triethyl phosphine oxide (3.0 mg, 0.022 mmol, 1.0 eq.) in CD_2Cl_2 (0.5 mL). After initial multinuclear NMR acquisition, benzo[c][1,2]oxaborol-1(3H)-ol (8.5 mg, 0.065 mmol, 3.0 eq.) was added. The ^{31}P-NMR spectrum of the mixture showed a downfield shift of the phosphorus resonance (from 50.8 to 55.6 ppm). The overall Δδ was determined to be 4.8 ppm. The ^{1}H-NMR spectrum confirms this interaction, with a downfield shift of the Et_3PO methylene resonances to δ1.75 ppm (cf. δ1.64 ppm for free Et_3PO).

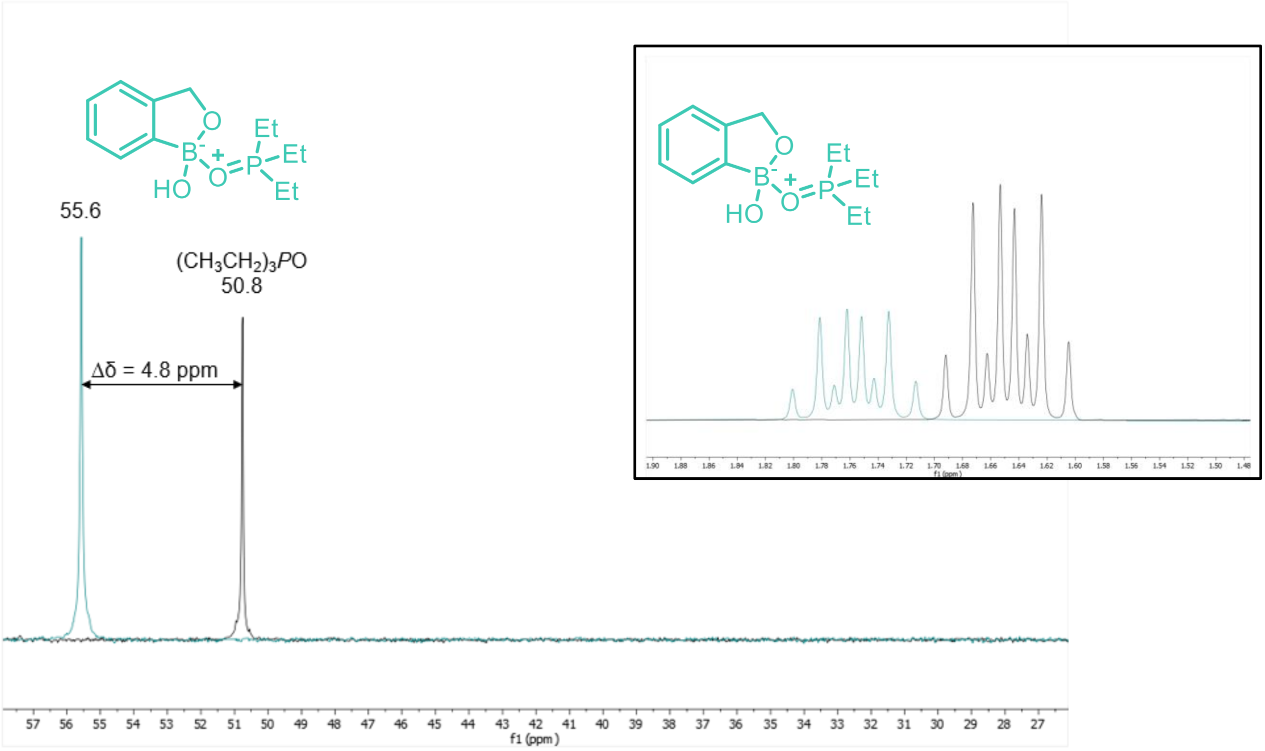

Figure S1: In situ ^{1}H and ^{31}P-NMR of Gutmann-Beckett method between Et_3PO and benzo[c][1,2]oxaborol-1(3H)-ol.

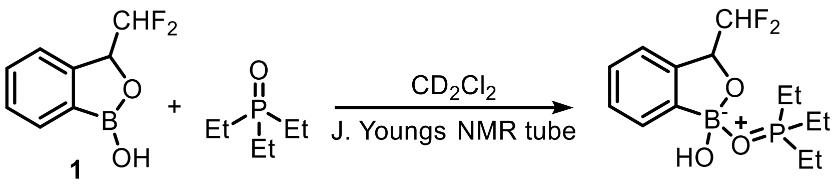

The procedure has been adapted from the literature.[9] A J. Youngs NMR tube was loaded with triethyl phosphine oxide (3.0 mg, 0.022 mmol, 1.0 eq.) in CD_2Cl_2 (0.5 mL). After initial multinuclear NMR acquisition, compound **1** (12 mg, 0.065 mmol, 3.0 eq.) was added. The ^{31}P-NMR spectrum of the mixture showed a downfield shift of the phosphorus resonance (from 50.8 to 56.7 ppm). The overall $\Delta\delta$ was determined to be 5.9 ppm. The ^{1}H-NMR spectrum confirms this interaction, with a downfield shift of the Et_3PO methylene resonances to δ1.78 ppm (cf. δ1.64 ppm for free Et_3PO in CD_2Cl_2).

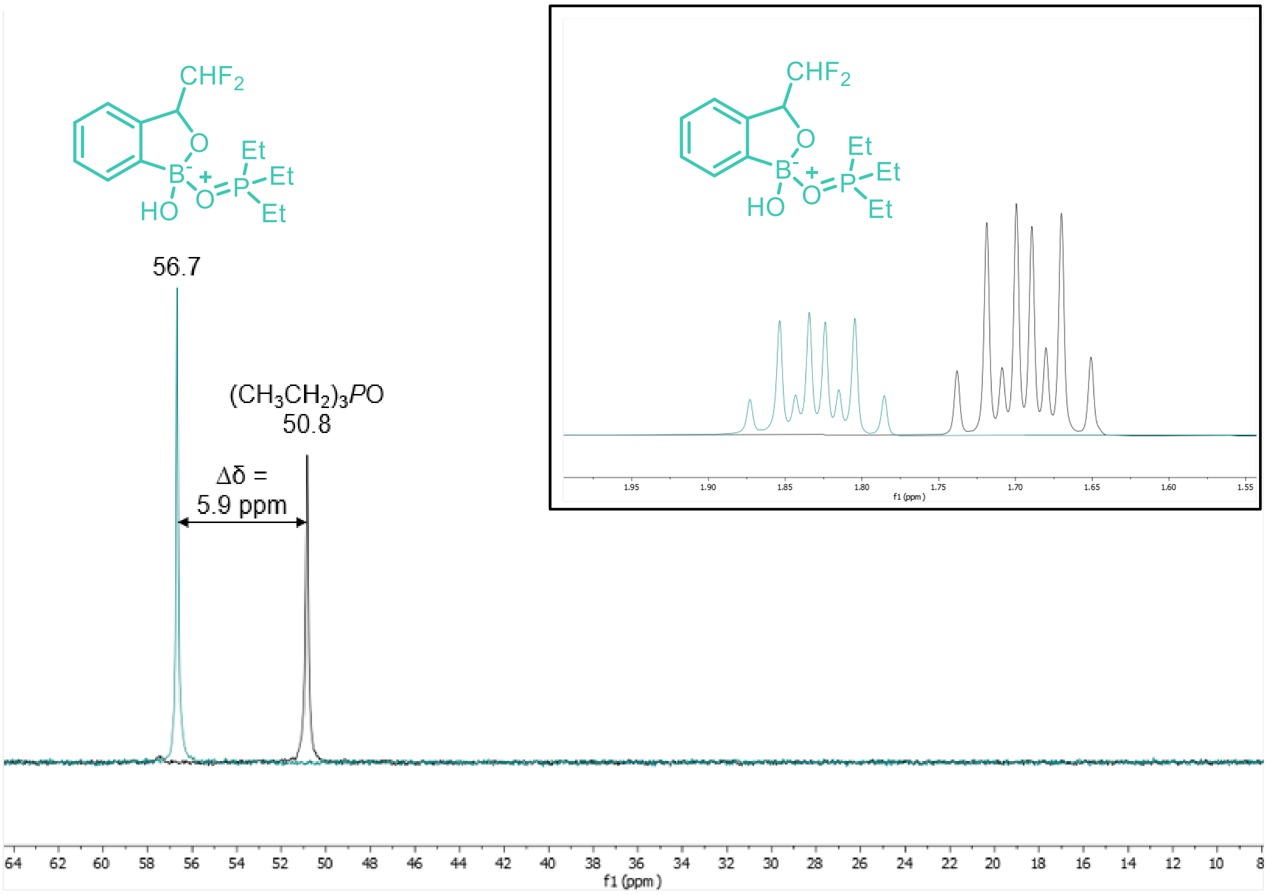

Figure S2: In situ ^{1}H and ^{31}P-NMR of Gutmann-Beckett method between Et_3PO and **1**.

2.7 Single-crystal X-ray diffraction analysis

2.7.1 Single-crystal X-ray diffraction of racemic mixture

Crystallization method: slow evaporation.

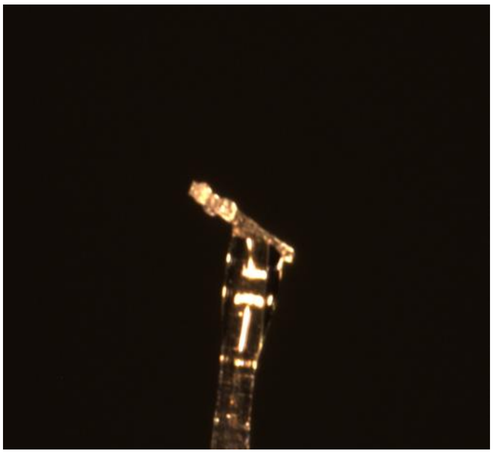

Figure S3: Sample description: prism/needles, transparent, colorless with dimensions: 0.34 x 0.07 x 0.07 mm.

Mounting: on a glass fiber, with perfluorinated oil

Comments: The selected sample was obtained from a slow-evaporation batch and was chosen from various fragments produced by cracking a block of epitaxially grown crystals using a micro-spade. The selected sample shows strong pleochroism under polarized light, from colorless to gray. Small fragments were observed on the crystal surface, exhibiting different pleochroism patterns. To remove them, the crystal was gently rubbed with needles in a drop of fluorinated oil. The final sample still showed slight heterogeneity, but its overall quality was deemed sufficient for X-ray diffraction experiment.

Instrumental specs:

Device: Rigaku XtaLAB Synergy-S 4-circle diffractometer

Source: microfocus sealed tube

Detector: Hybrid Photon Counting (HPC)

Experiment temperature: 298(2) K

Cryostat: not used

Wavelength: Cu K_α (1.54184 Å).

Data collection extent: full sphere within $\sin\vartheta/\lambda$ = 0.64 $Å^{-1}$

Data collections specs: Detector-to-sample distance: variable, several ω-scan run.

Measured reflections: 20166, 18088ndependents

Maximum resolution (ϑ): 79.488 °

Completeness: 98.2 % (at full sphere resolution)

Data reduction programs:

Integration: CrysalisPro

Reduction: CrystalsPro

Structure solution and refinement: Shelxs2019, Shelxl 2019

Unit cell, lattice and crystal system:

Bravais lattice: Monoclinic, Primitive

Space group: P -1, No. 2

Point group: -1

Laue group: -1, No. 1

Unit cell (Å, deg, $Å^3$): a = 5.2676(2), b = 7.5859(2), c = 10.9676(3), α = 91.395(2), β = 103.888(2), γ = 93.242(2), V = 424.44(2) as estimated from 10144 intense reflections among 4.1450 ° e 78.8290 ° of ϑ (final integration result).

Formula units in cell (Z): 2

Formula units in the asymmetric unit (Z'): 1

Number of electrons in cell (F_{000}): 188

Computed density: 1.439 g/cm^3

Linear absorption coefficient (μ): 1.116 mm^{-1}

Main statistical results:

Final stats for the spherical atom model (Shelxl):

Scale factor: 10.78(3)

BASF parameter: //

Secondary extinction coefficient: none

<Δ/σ> = 0.000

R1(F) = 0.0393 for 1424 > 4σ(F_o), 0.0518 for all the 1808 independent data

wR(F^2) = 0.1204 for all the measured data

Goodness-of-fit: 1.096

Flack's parameter: //

$\Delta\rho_{MAX/MIN}$ = +0.159 e/Å^3 at ~ 0.74 Å from the C4 carbon, –0.162 e/Å^3 at ~ 1.04 Å from the C8 carbon atom.

The least squares statistical analysis indicates that the overall quality of the collected data is high, as the R1 agreement parameter is below 4% for intense data, and around 5% for all the collected independent data. Non-structured small residual electron densities are observed in the nearby C atoms, and they can be neglected as no evidence of disorder is found. Further details can be found in the next sections. In general, refined geometry is reliable, both in terms of chemical connectivity and absolute stereochemistry.

Molecular schemes

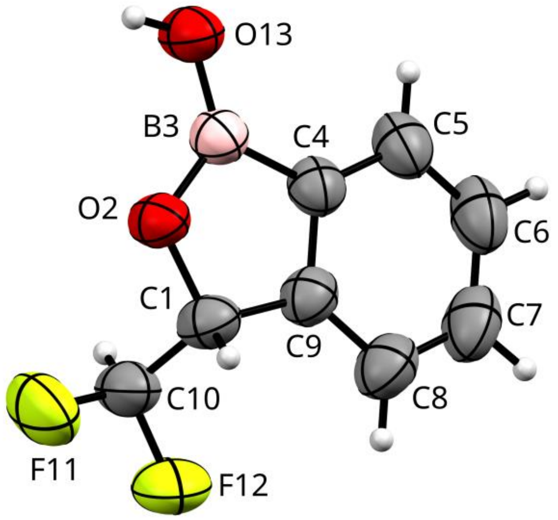

Figure S4: Absolute conformation of the (R)-enantiomer of the FGU molecule, with atom labels shown for non-hydrogen atoms. The (S)-enantiomer is also present in the primitive cell due to the inversion center of the P-1 space group. Thermal ellipsoids for non-H atoms are shown at the 50% probability level. The color code employed for atoms is the following: C, grey; H, white; O, red; B, pink.

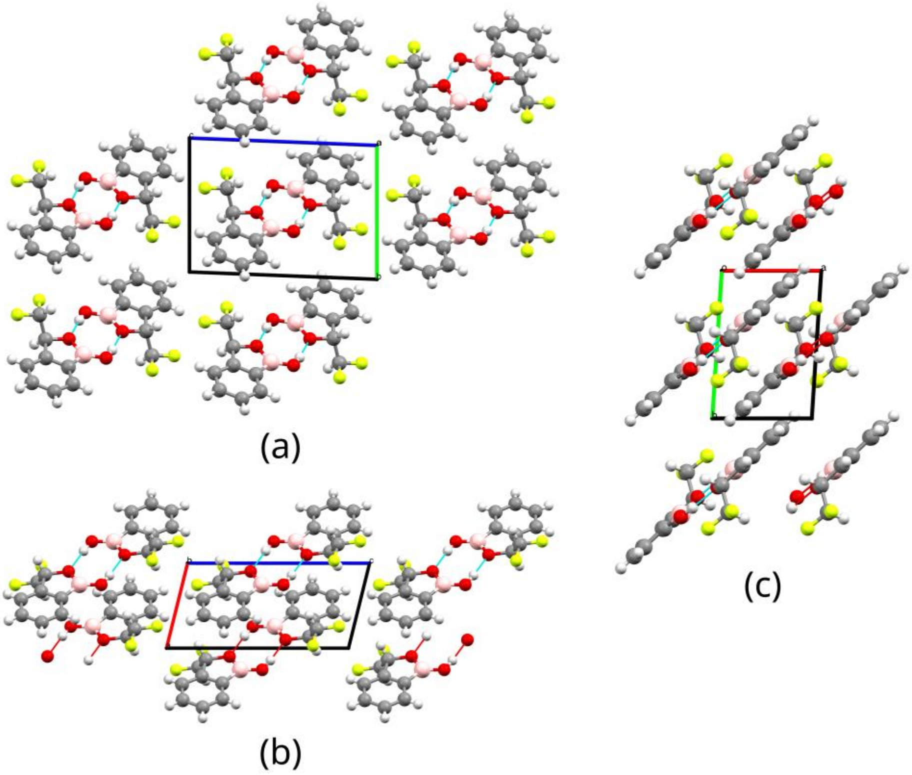

Figure S5: Crystal packing of FGU racemate at 298 K, viewed along the (a) the **a** cell axis, (b) the **b** cell axis, and (c) the **c** cell axis. The hydrogen bonds between the hydroxyl group and the heterocyclic oxygen are shown as solid red lines. Atomic color code as in Figure S4.

Discussion and conclusions:

(1) The compound crystallizes in the triclinic, achiral, and centrosymmetric space group P$\bar{1}$ (No. 2) as a racemate. The boron atom adopts a planar (sp^2-like) geometry, maintaining the bicyclic scaffold essentially flat. The hydroxyl hydrogen lies within the same plane as the rigid framework, as this conformation maximizes the strength of the hydrogen bond with the inversion-related molecule. The difluoromethyl substituent adopts a staggered conformation with respect to the hydrogen atom of the five-membered ring.

(2) Figure S5 illustrates the main packing motif of FGU. As expected, a strong hydrogen bond is set between the hydroxyl group (O13-H13) and the oxygen atom of the five-terms ring (O2). The presence of the inversion allows each molecule to behave both as donor and acceptor of hydrogen bonds, leading to the formation of cyclic dimers, that can be considered the molecular synthon forming the crystal. This packing motif is shown in Figure 3. This packing mode is particularly favorable and strongly contributes to the thermodynamical stabilization of the system.

On the other hand, the dimeric units pack together with less strong interactions: π-π interactions between the aromatic rings, approximately in the (111) crystallographic direction, and CH···O bonds between the difluoromethyl group and the same O2 oxygen involved in the stronger classical H-

interaction described above.

In contrast, the dimeric units are held together by weaker interactions, including π–π stacking between aromatic rings—approximately along the (111) crystallographic direction, a CH···O hydrogen bonds involving the difluoromethyl group and the hydroxyl, O13 atom—and a CH···F bond. Figure S6 also shows these interactions, while Table S1 reports the geometrical parameters of the shortest intermolecular contacts.

D–H···A	$d_{D–H}$, Å	$d_{H···A}$, Å	$d_{D···A}$, Å	α_{DHA}, °	Symmetry operation
O13–H13 ··· O2	0.90	1.89	2.7915(1)	175	-x, 1-y, 1-z
C6–H6 ··· F11	0.93	2.49	3.3009(1)	146	1+x, -1+y, z
C10–H10···O13	0.98	2.42	3.3408(1)	156	1-x, 1-y, 1-z

Table S1: Hydrogen bonds short contacts

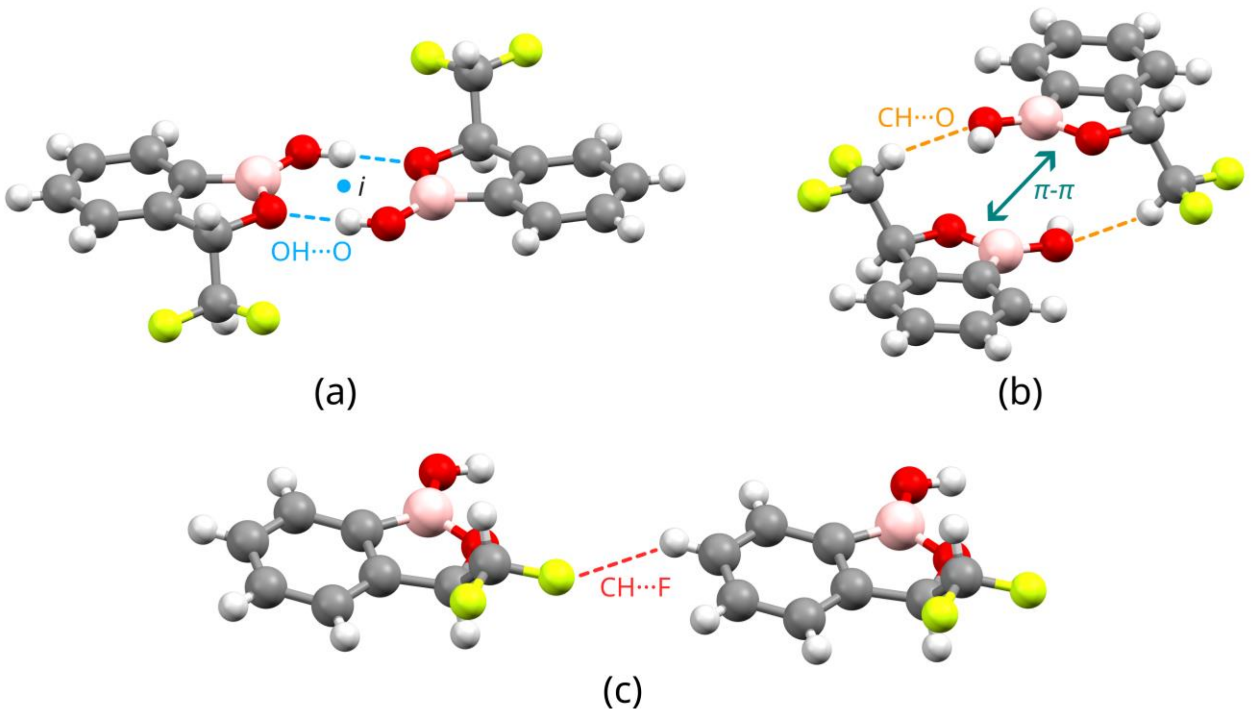

Figure S6: Main intermolecular interactions of FGU racemate. (a) The strong hydrogen bonds between the hydroxyl group and the heterocyclic oxygen (cyan dotted lines); (b) the weaker CH•••O (orange dotted lines) and the ring-ring π interactions (teal double arrow); (c) the CH•••F bond (red dotted lines).

2.8 Biological evaluation

2.8.1 Minimal Inhibitory Concentration

The Minimal Inhibitory Concentration (MIC) of the benzoxaboroles derivative-compounds, was evaluated with the standard microdilution method as previously described, with minor modifications.[1,2] Briefly, Escherichia coli MG1655 was grown at 37°C with shaking in Luria Bertani (LB) broth (10 g/L tryptone, 5 g/L yeast extract, 5 g/L NaCl).[10] After 16hrs of growth, the culture was diluted in 200 µL of LB to an optical density at 600 nm (OD600) of about 0.02 [ca. 2x107 Colony Forming Units per mL (CFU/mL)] in 96-well microtiter plates with increasing concentrations of the selected compounds. Since all the compounds were dissolved in dimethyl sulfoxide (DMSO), samples grown in the presence of the same amount of the solvent vehicle [corresponding to the maximum amount of 2.56% (v/v) DMSO] were used as controls in each microtiter plate, without affecting bacterial growth. The MIC values were evaluated after 24 hrs of static incubation at 37°C, by measuring the OD600 at the automated EnSight Multimode plate reader (PerkinElmer). (see Minimal Inhibitory Concentration assays)

Percentage of compounds **16b** and **17b** were detected by ^{1}H NMR in a DMSO-d_6 solution of their precursors, **16a** and **17a** (see spectrum)

Compound	Structure	IC_{50} *E. coli* (MG1655) (µM)
**Tavaborole**	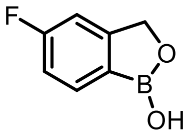	49.5
**1**	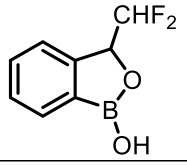	79.1
**2**	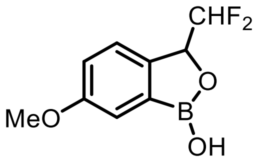	986.3
**3**	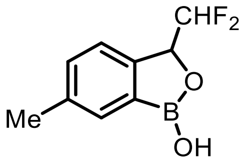	58.6
**4**	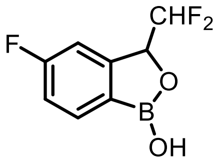	64.1

**5**	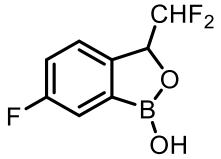	162.0
**6**	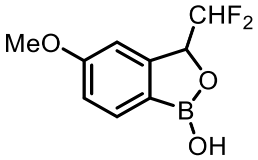	422.7
**12**	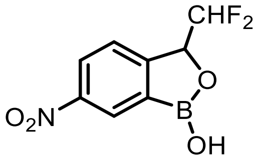	542.2
**13**	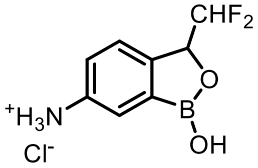	243.3
**14**	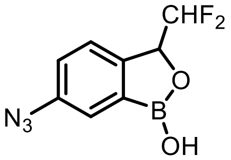	225.4
**15**	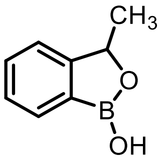	590.9
**16b**	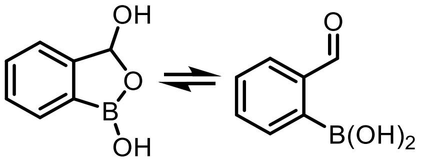	180.9
**17b**	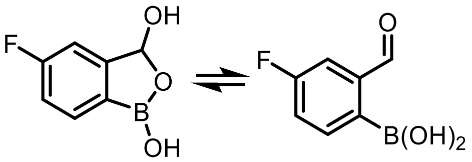	130.5

Table S2: Antimicrobial activity against E.ColiMG1655 for benzoxaboroles **1-17b**

2.8.2 Biofilm formation assay

The biofilm adhesion was evaluated with the crystal violet binding assay as previously described, with minor modifications.[11,12] Briefly, E. coli MG1655 was grown in LB at 30°C for 16 hours in shaking conditions and diluted to OD600 of about 0.02 in the same medium. Aliquots of 200 µl were then transferred to 96-well microtiter plates with increasing concentrations of the selected compounds. Samples grown in the presence of the same amount of the solvent vehicle [corresponding to the maximum amount of 2.56% (v/v) DMSO] were used as controls in each microtiter plate, without affecting bacterial growth and biofilm formation. Plates were incubated at 30°C for 20hrs in static conditions. After 20hrs, the liquid phase was removed, the OD600 was measured at the EnSight plate reader, and the attached cells were stained with 0.1% (w/v) crystal violet for 15 min. After washing the wells four times with distilled water, the surface-associated dye was solubilized with 200 µL of ethanol and the absorbance at 595nm (A595) of the dye solutions was measured at the EnSight. The biofilm attached at the surface of the wells was calculated as the adhesion units index corresponding to the A595/OD600 ratio. (see biofilm formation assays).

3. SPECTROSCOPIC DATA

^1H NMR (400 MHz, Acetone-d_6) of **I-B(BDEA)** (see procedure)

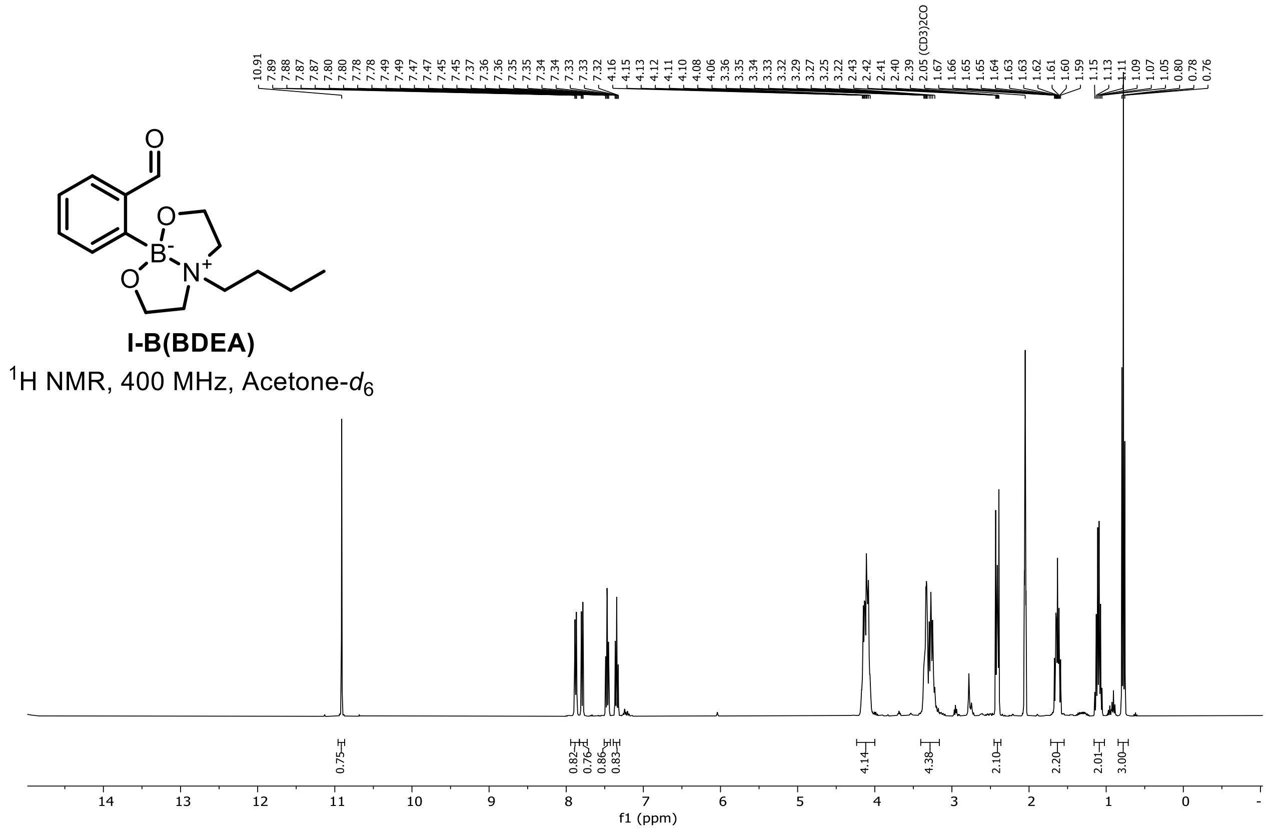

^{13}C NMR (101 MHz, Acetone-d_6) of **I-B(BDEA)**

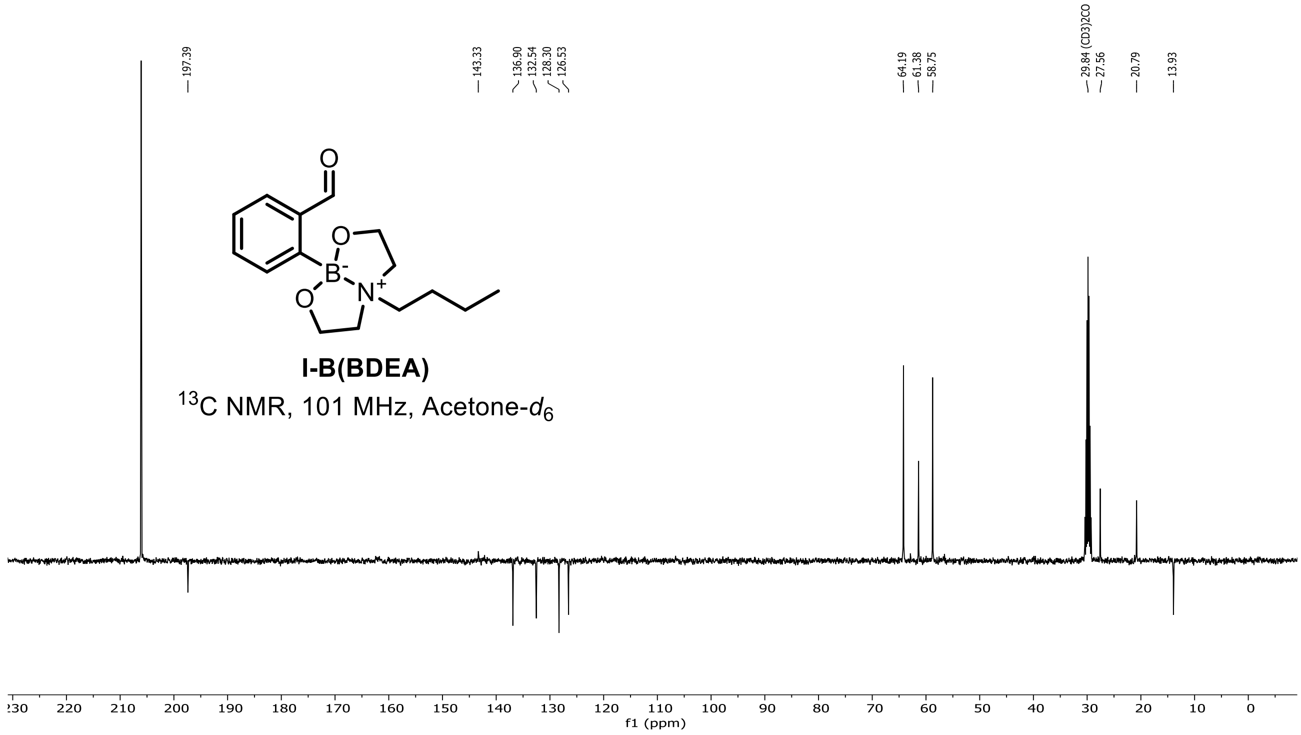

^{11}B NMR (128 MHz, Acetone-d_6) of **I-B(BDEA)**

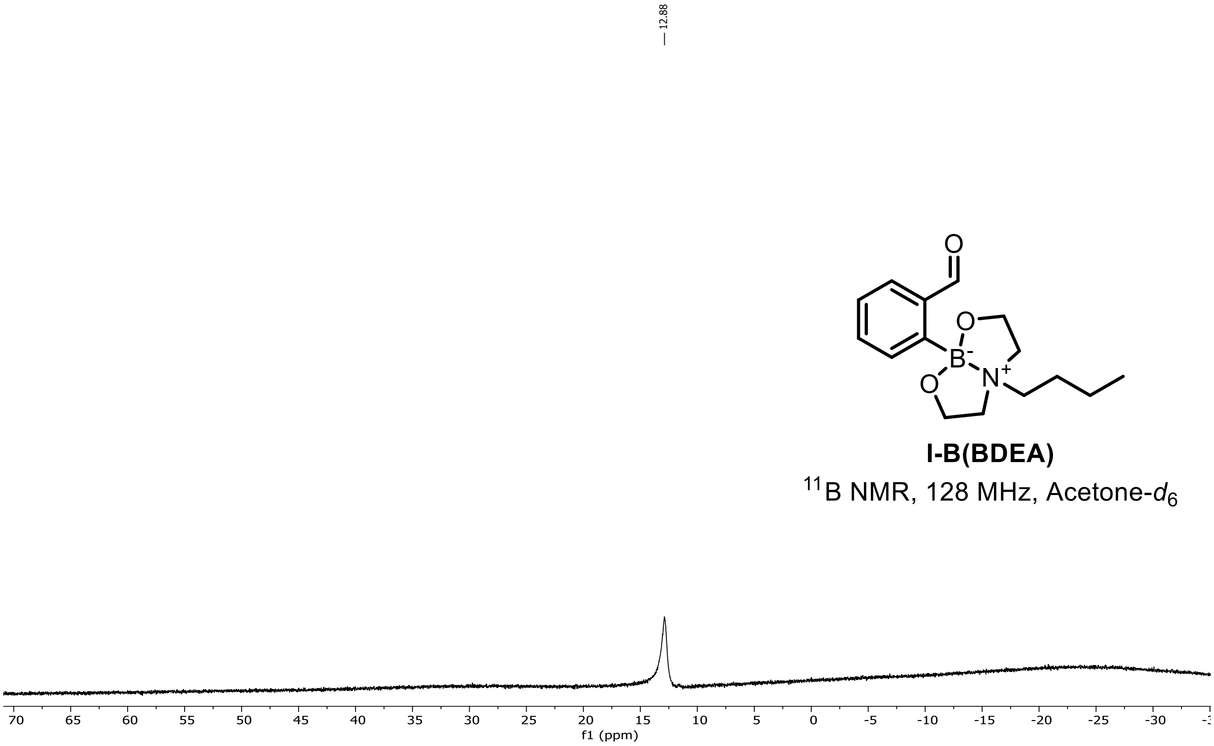

^{1}H NMR (400 MHz, Acetone-d_6) of **II-B(BDEA)** (see procedure)

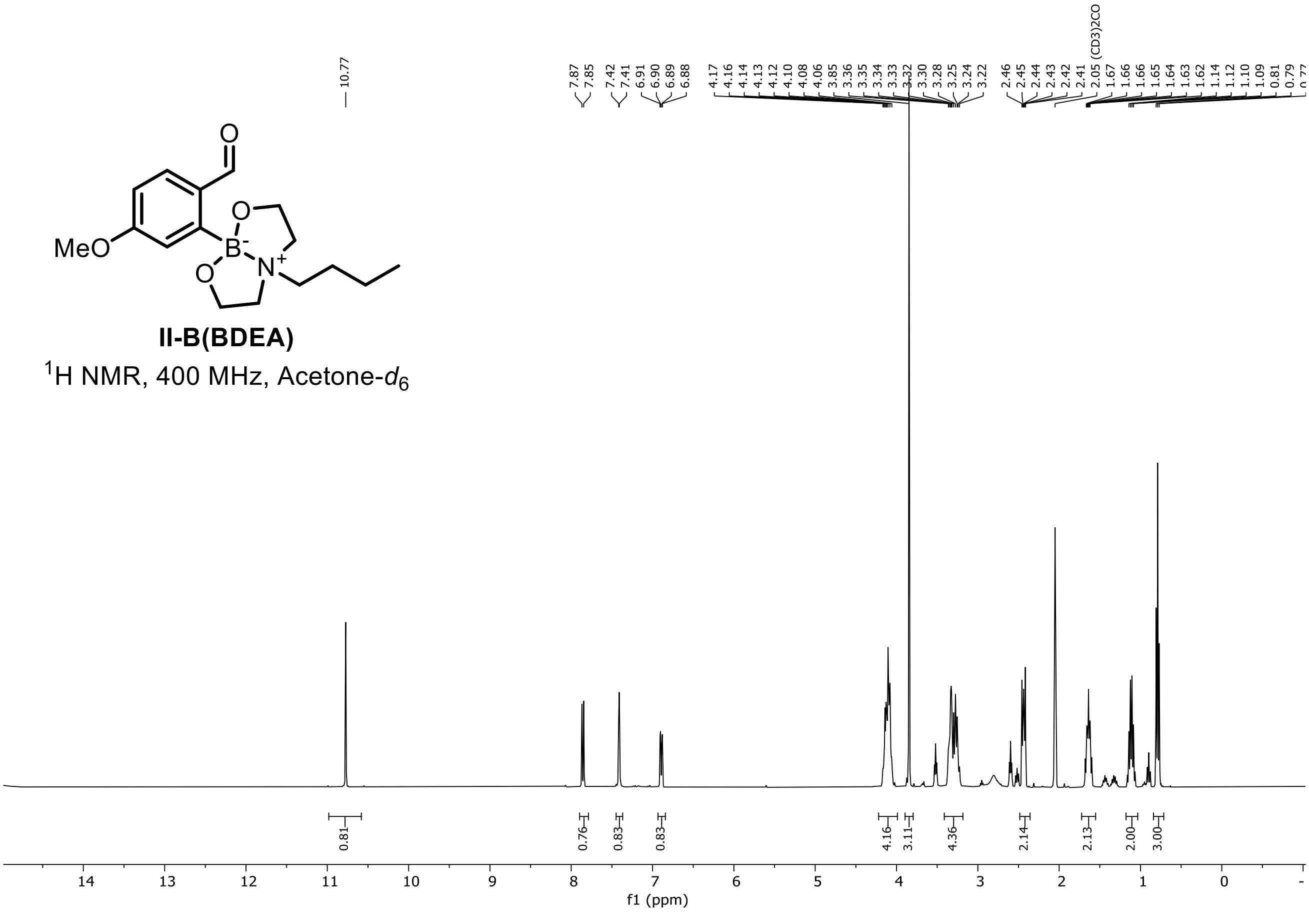

^{13}C NMR (101 MHz, Acetone-d_6) of **II-B(BDEA)**

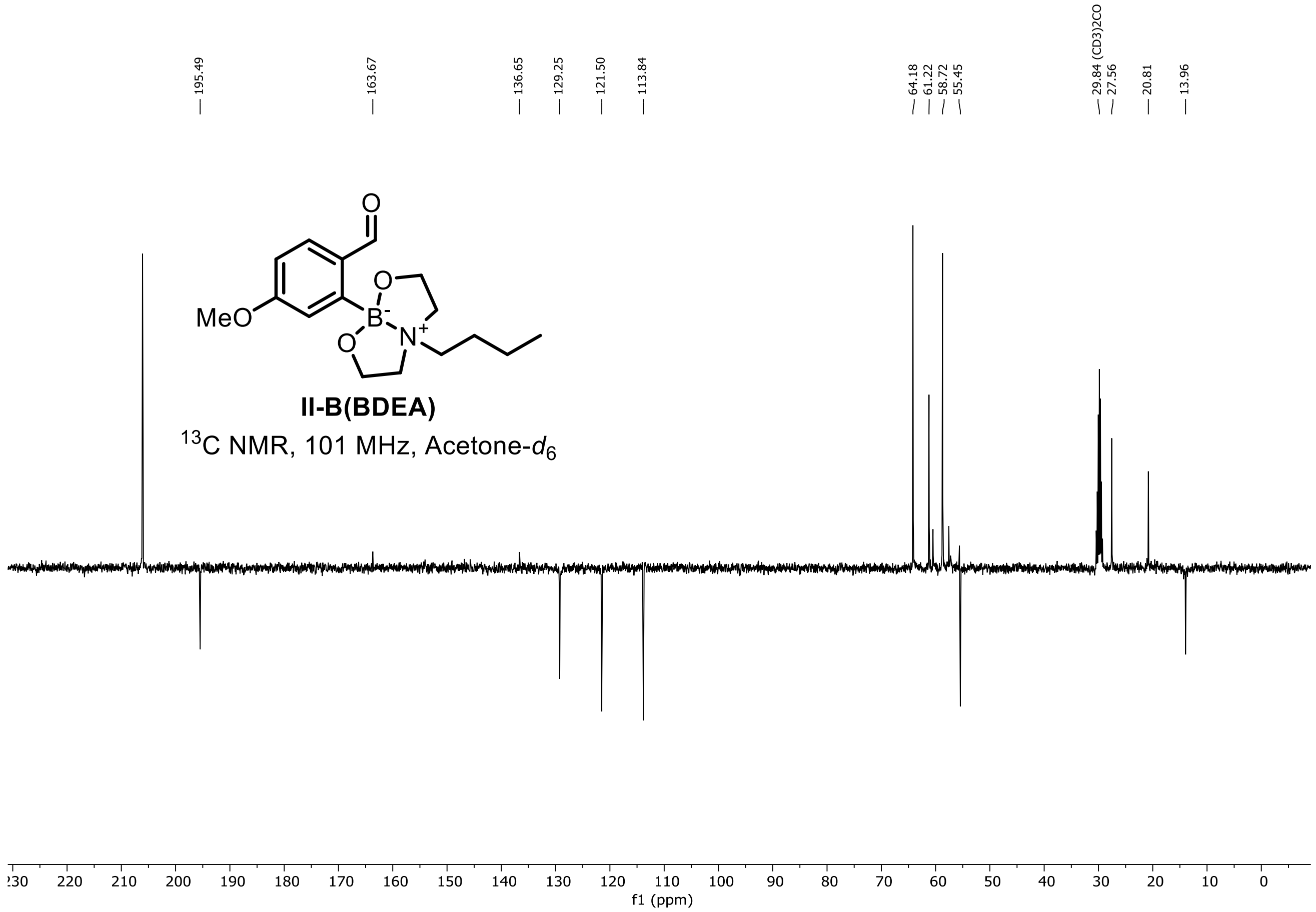

^{11}B NMR (151 MHz, Acetone-d_6) of **II-B(BDEA)**

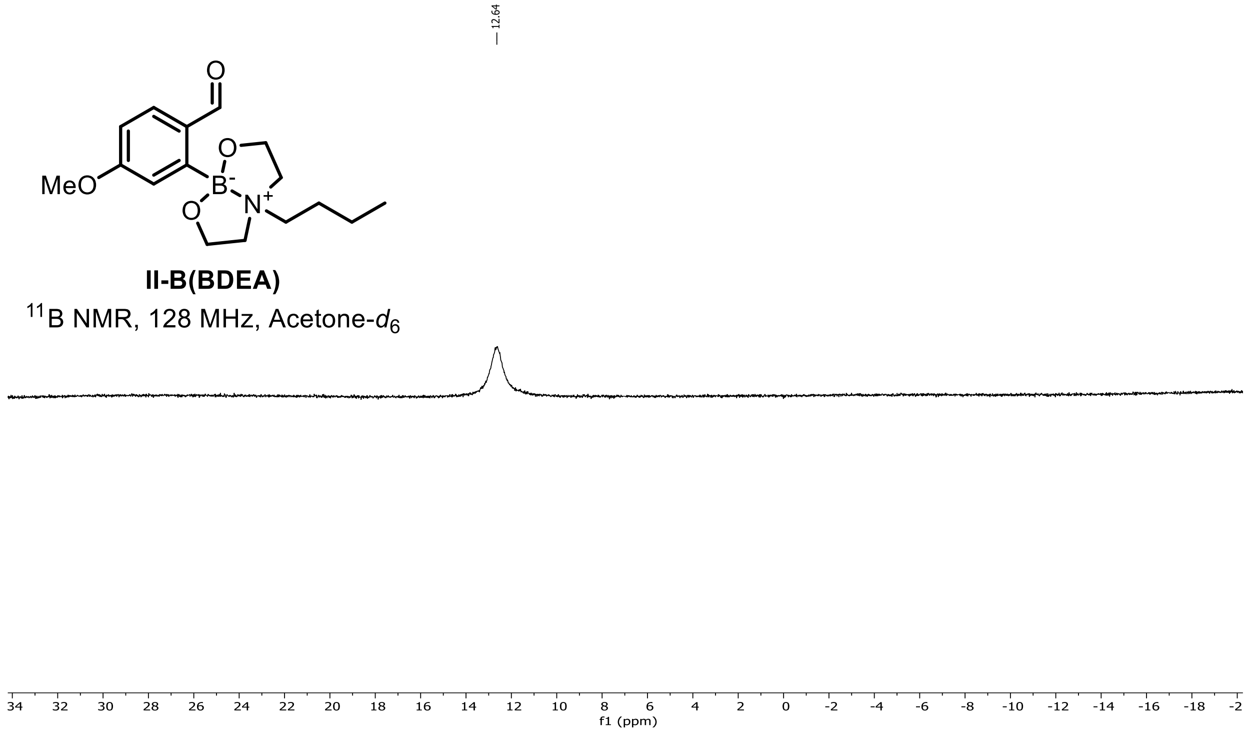

^{1}H NMR (400 MHz, Acetone-d_6) of **III-B(BDEA)** (see procedure)

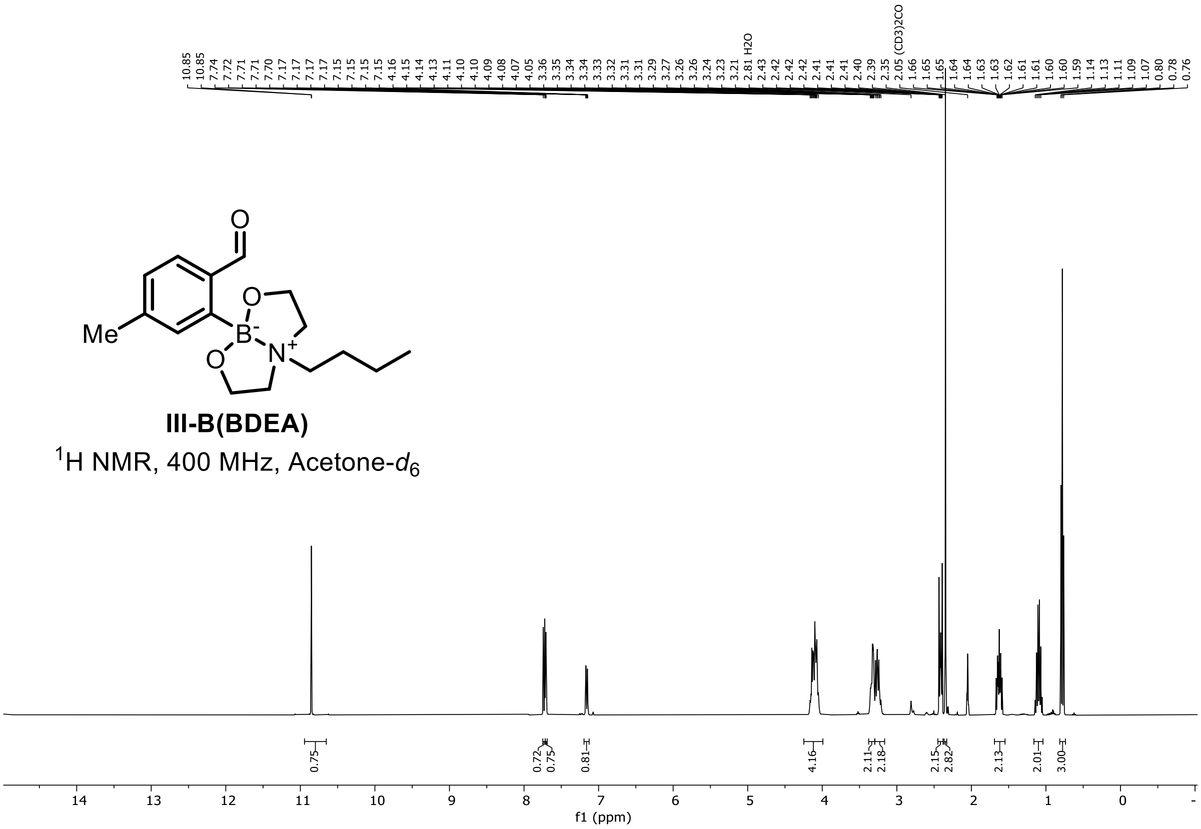

^{13}C NMR (101 MHz, Acetone-d_6) of **III-B(BDEA)**

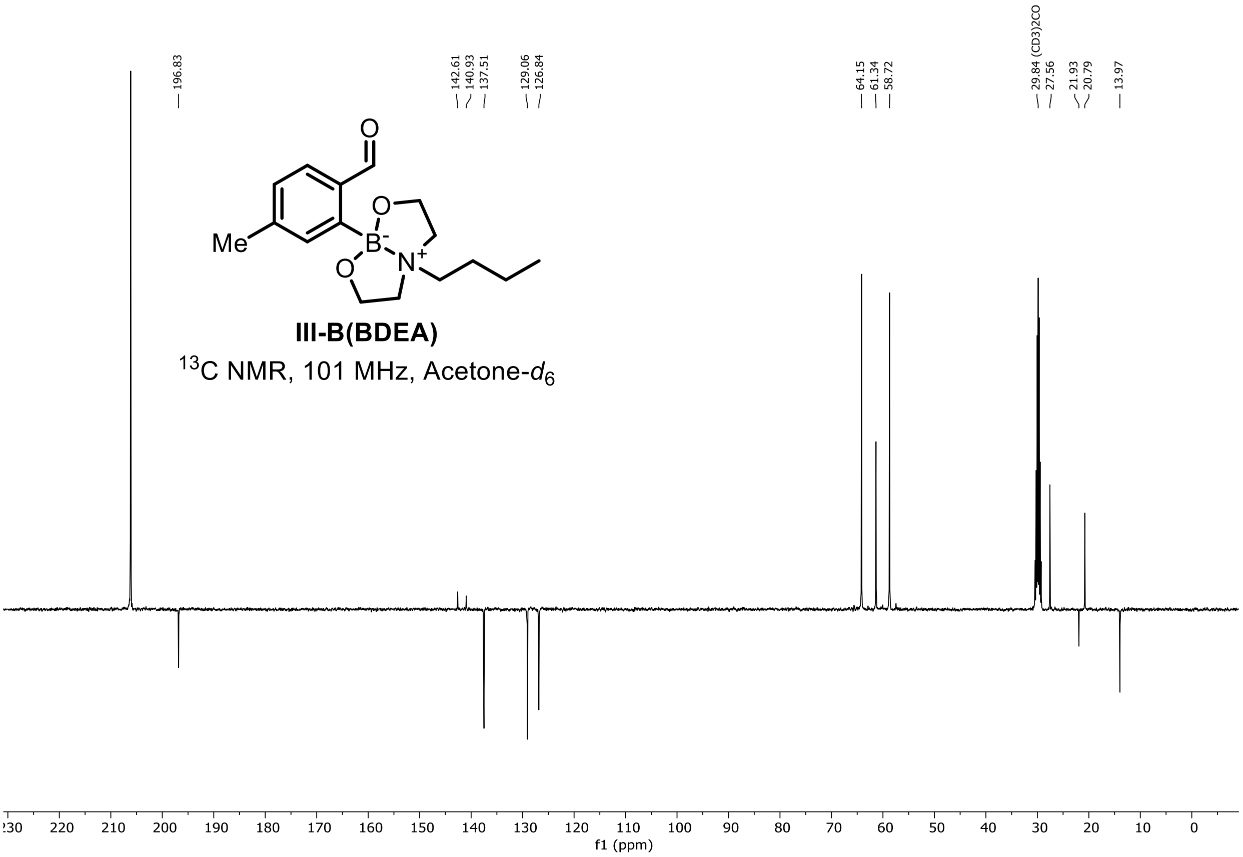

^{11}B NMR (151 MHz, Acetone-d_6) of **III-B(BDEA)**

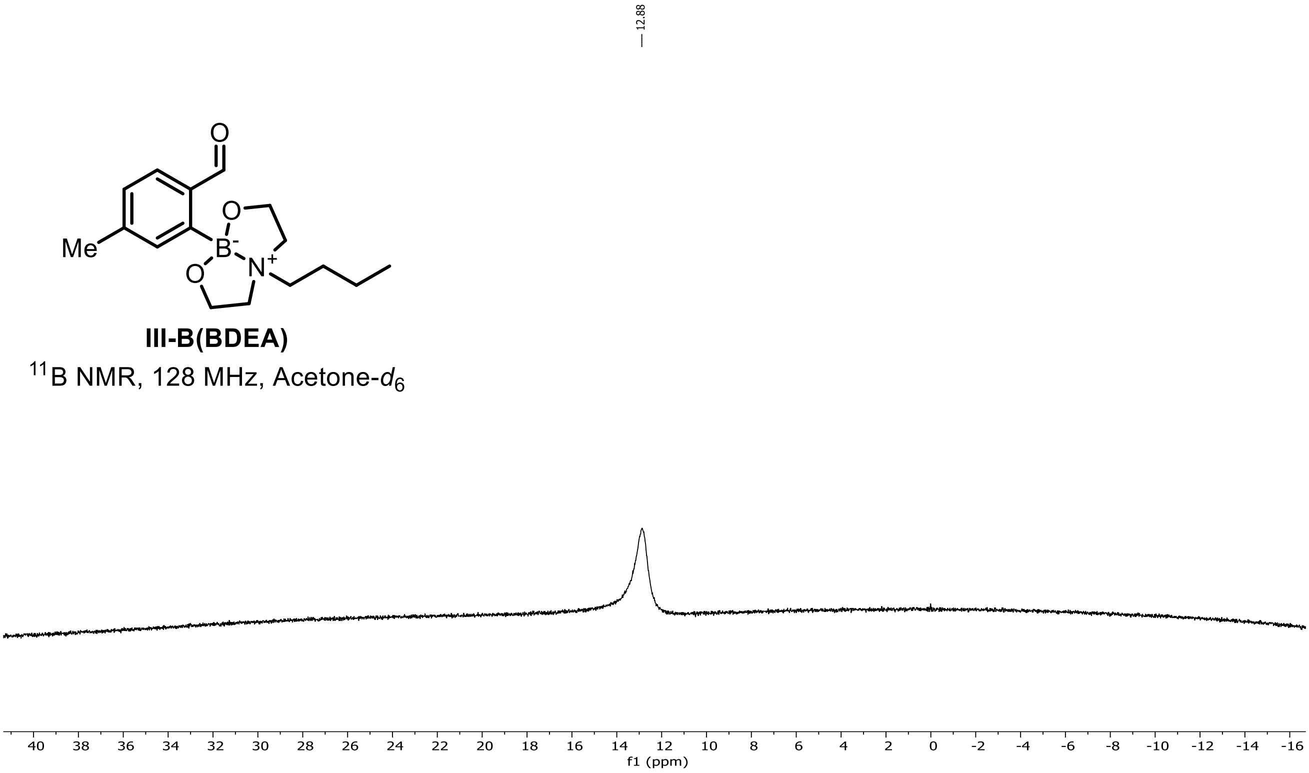

^{1}H NMR (400 MHz, Acetone-d_6) of **IV-B(BDEA)** (see procedure)

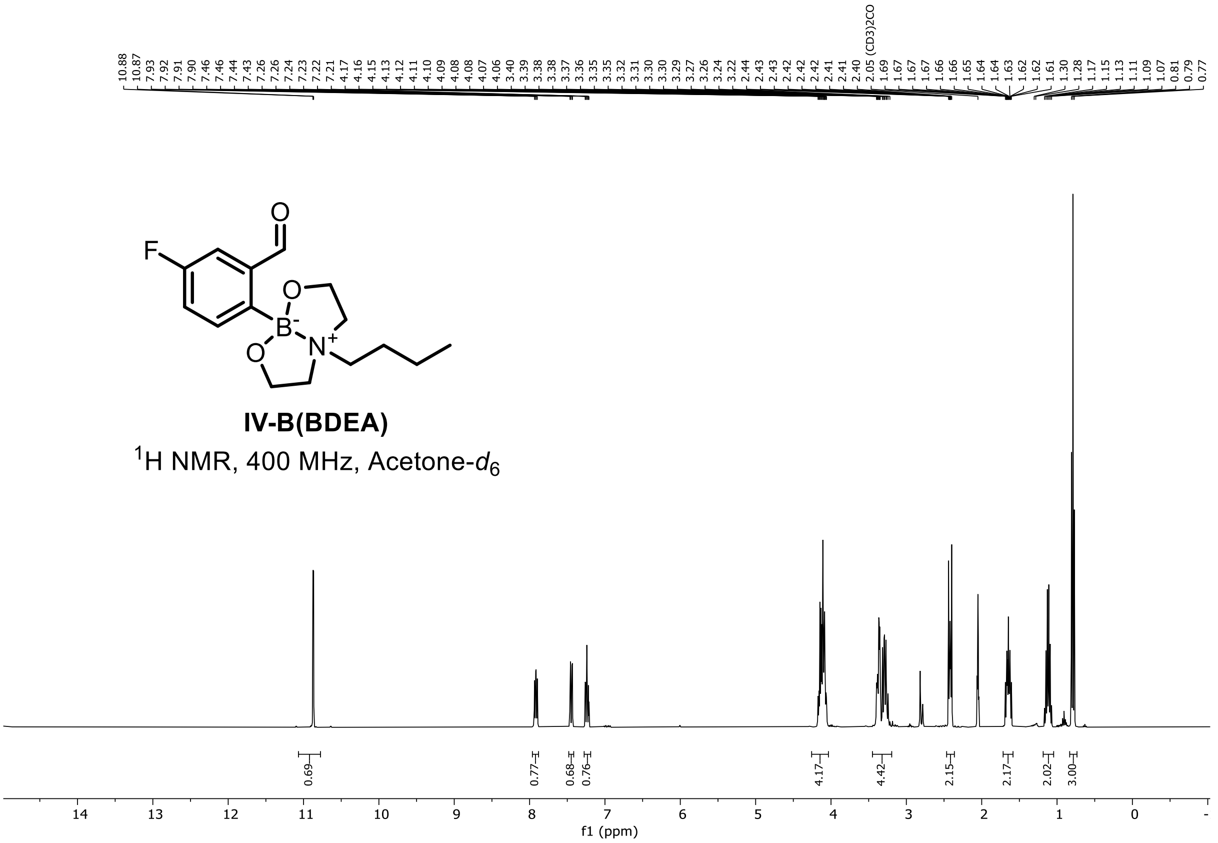

^{13}C NMR (101 MHz, Acetone-d_6) of **IV-B(BDEA)**

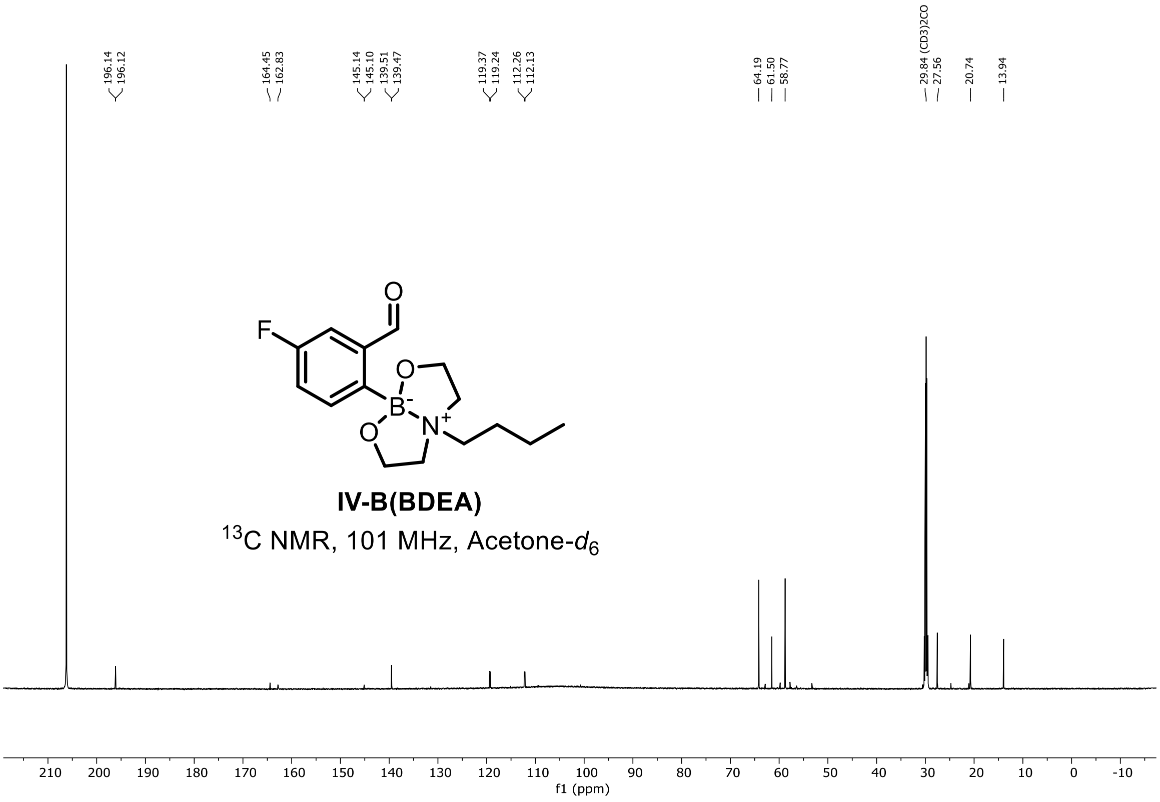

^{11}B NMR (128 MHz, Acetone-d_6) of **IV-B(BDEA)**

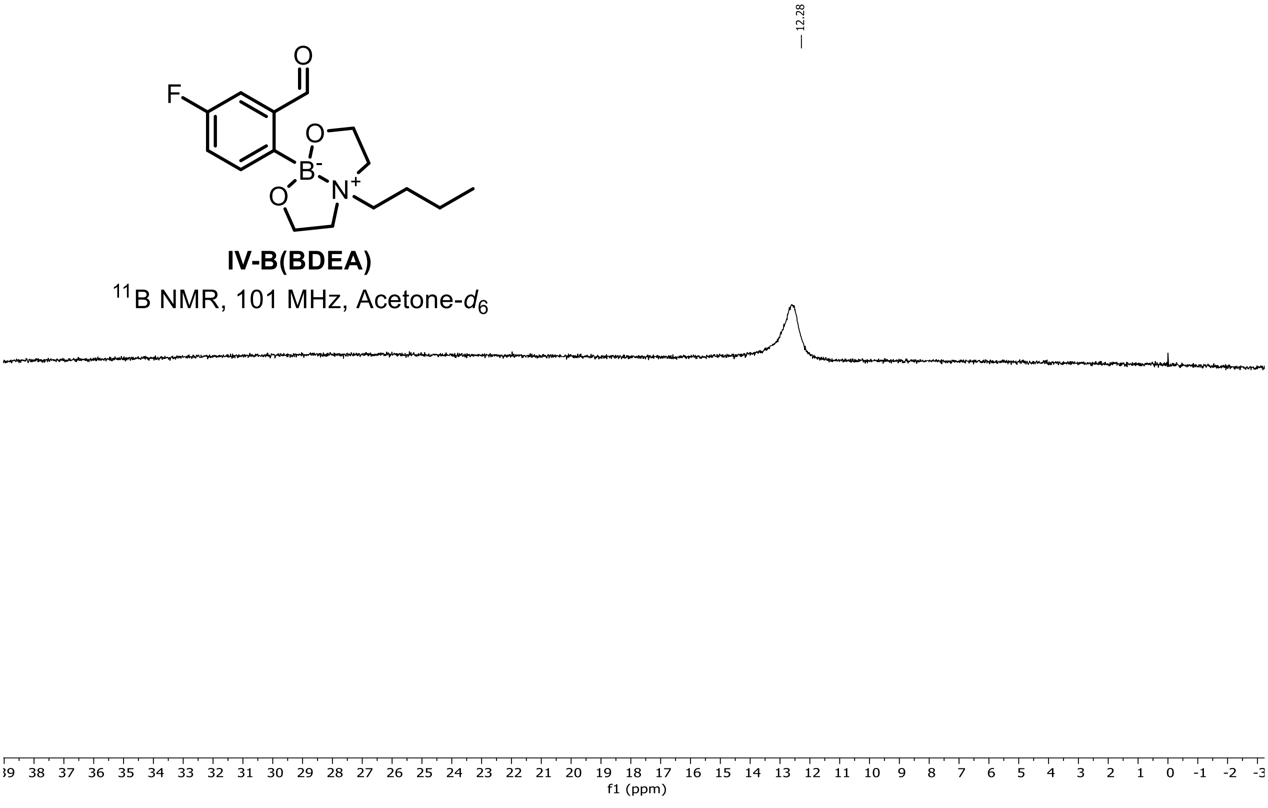

^{19}F NMR (376 MHz, Acetone-d_6) of **IV-B(BDEA)**

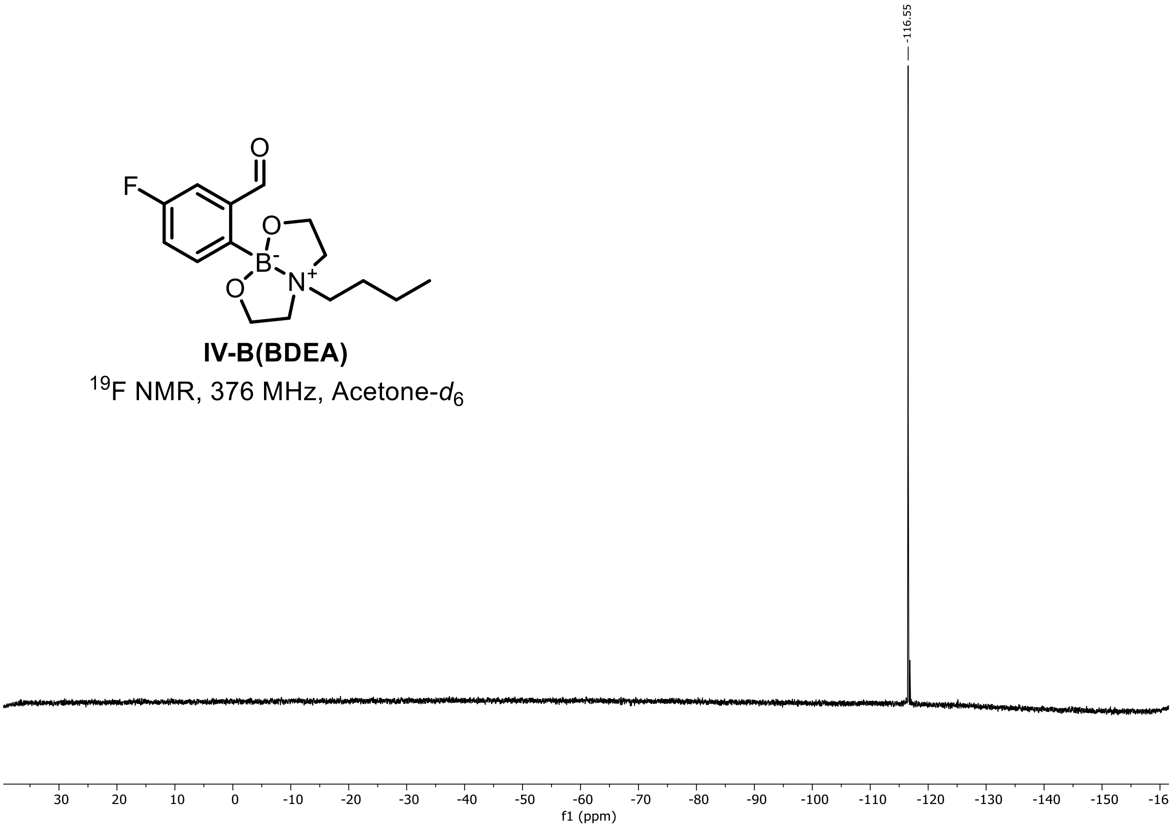

^{1}H NMR (400 MHz, Acetone-d_6) of **V-B(BDEA)** (see procedure)

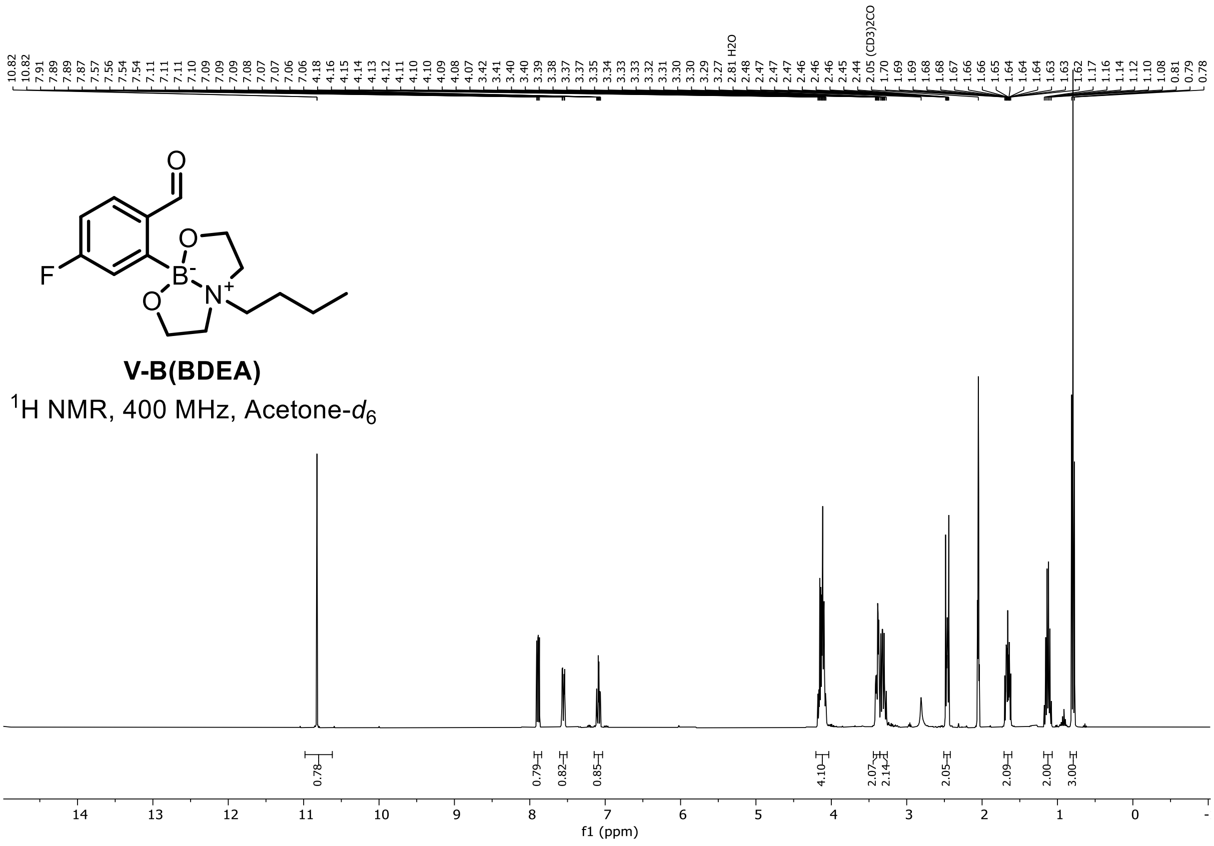

^{13}C NMR (101 MHz, Acetone-d_6) of **V-B(BDEA)**

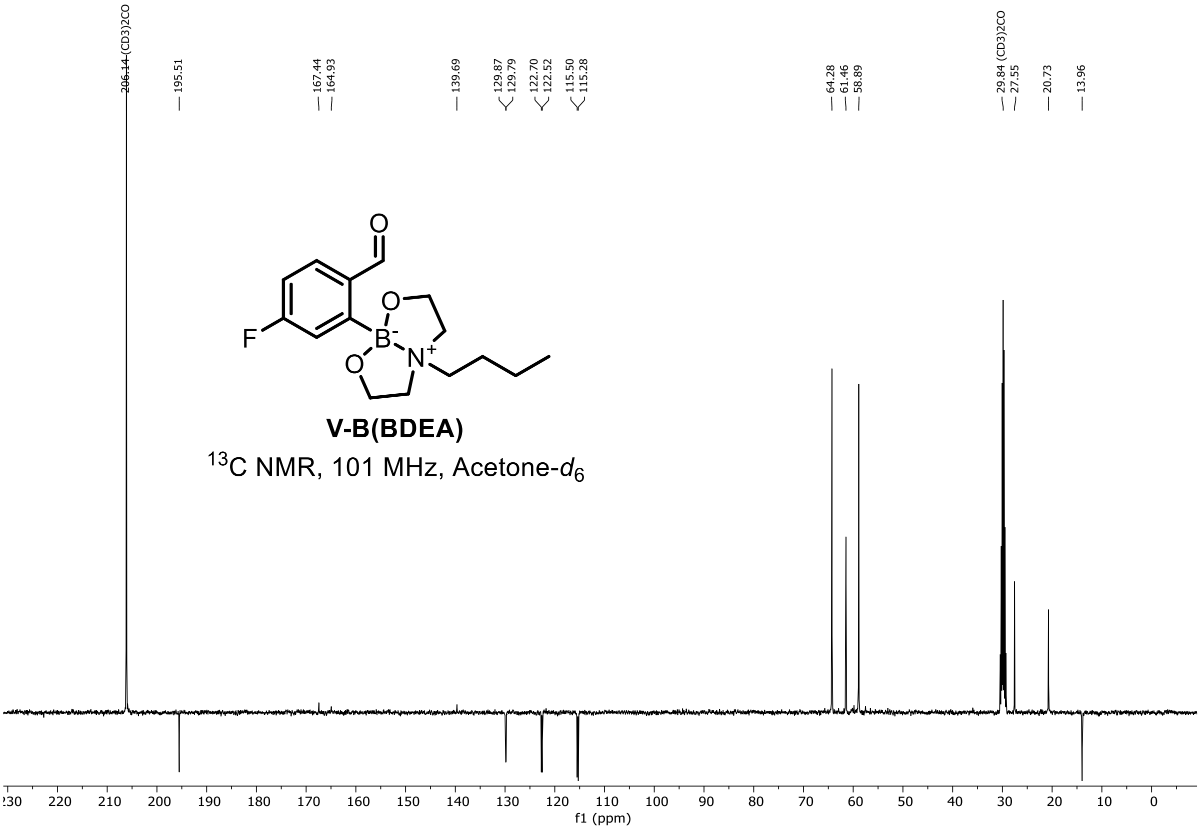

^{11}B NMR (128 MHz, Acetone-d_6) of **V-B(BDEA)**

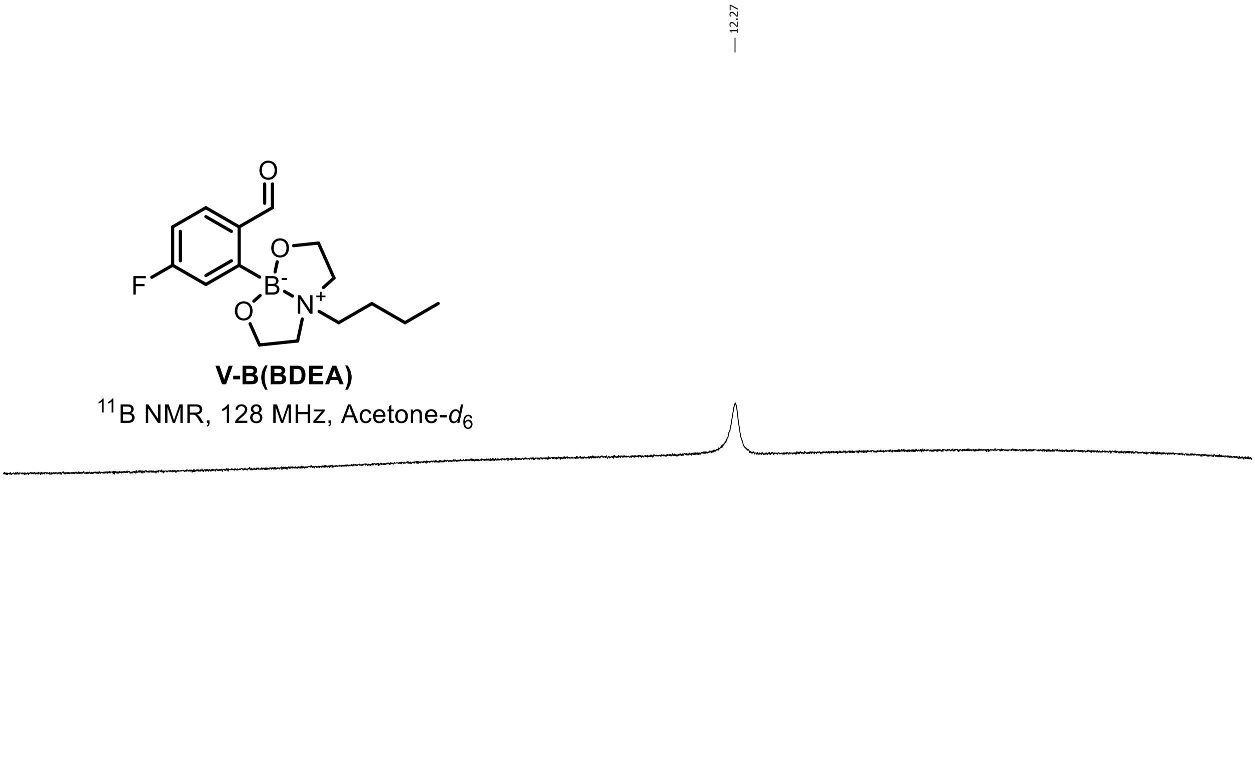

^{19}F NMR (376 MHz, Acetone-d_6) of **V-B(BDEA)**

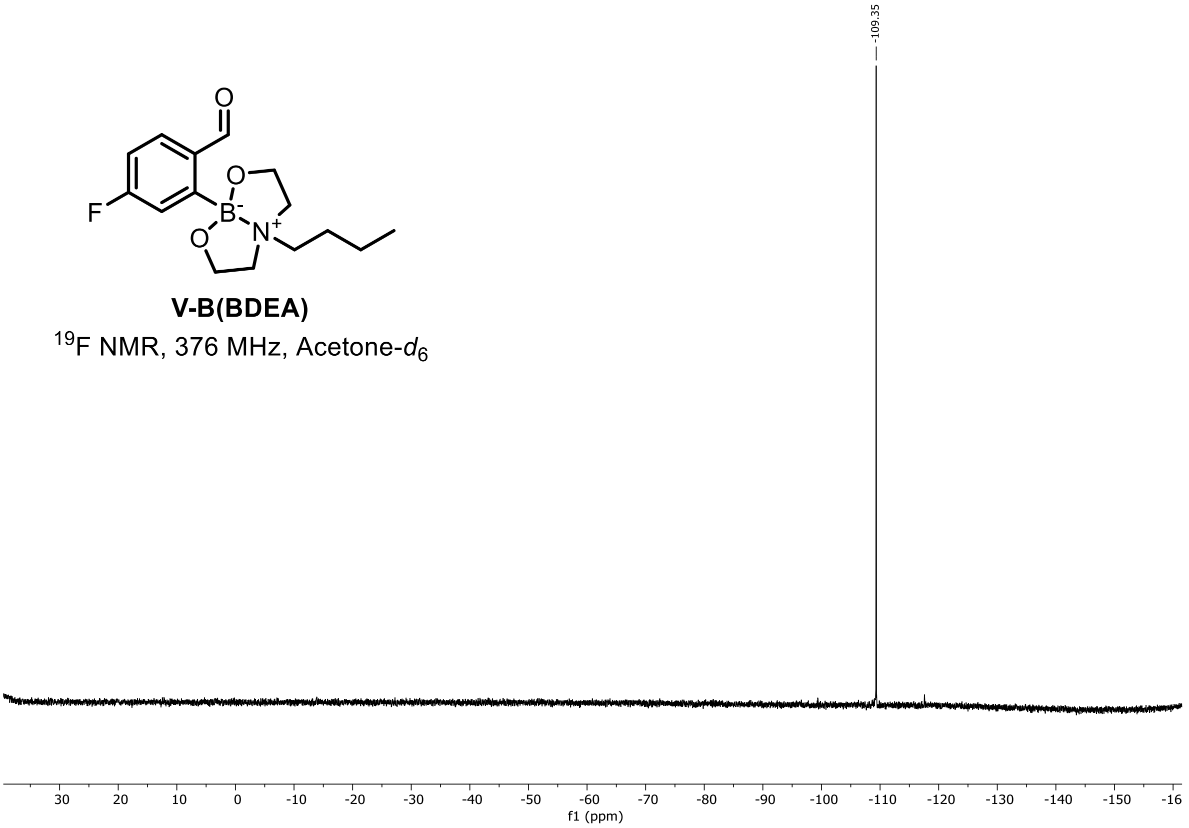

^{1}H NMR (400 MHz, Acetone-d_6) of **VI-B(BDEA)** (see procedure)

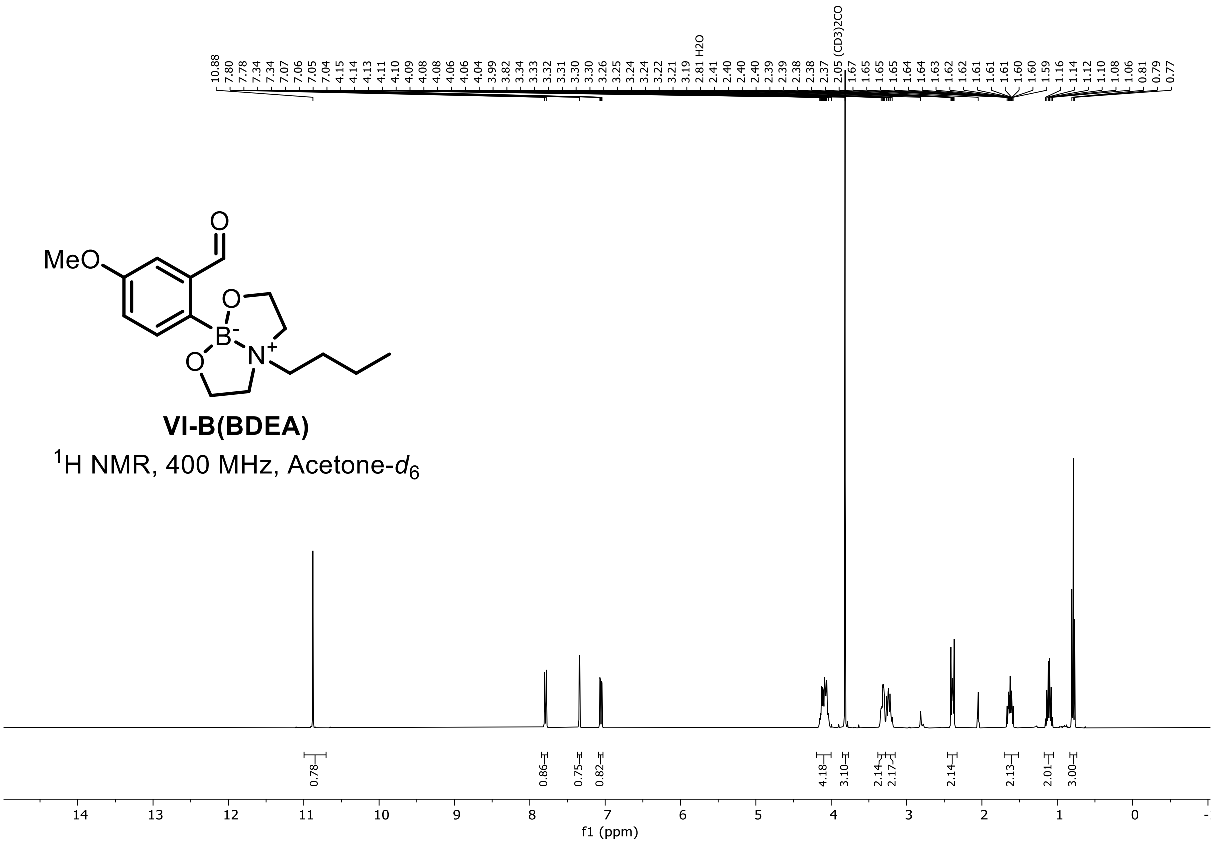

^{13}C NMR (101 MHz, Acetone-d_6) of **VI-B(BDEA)**

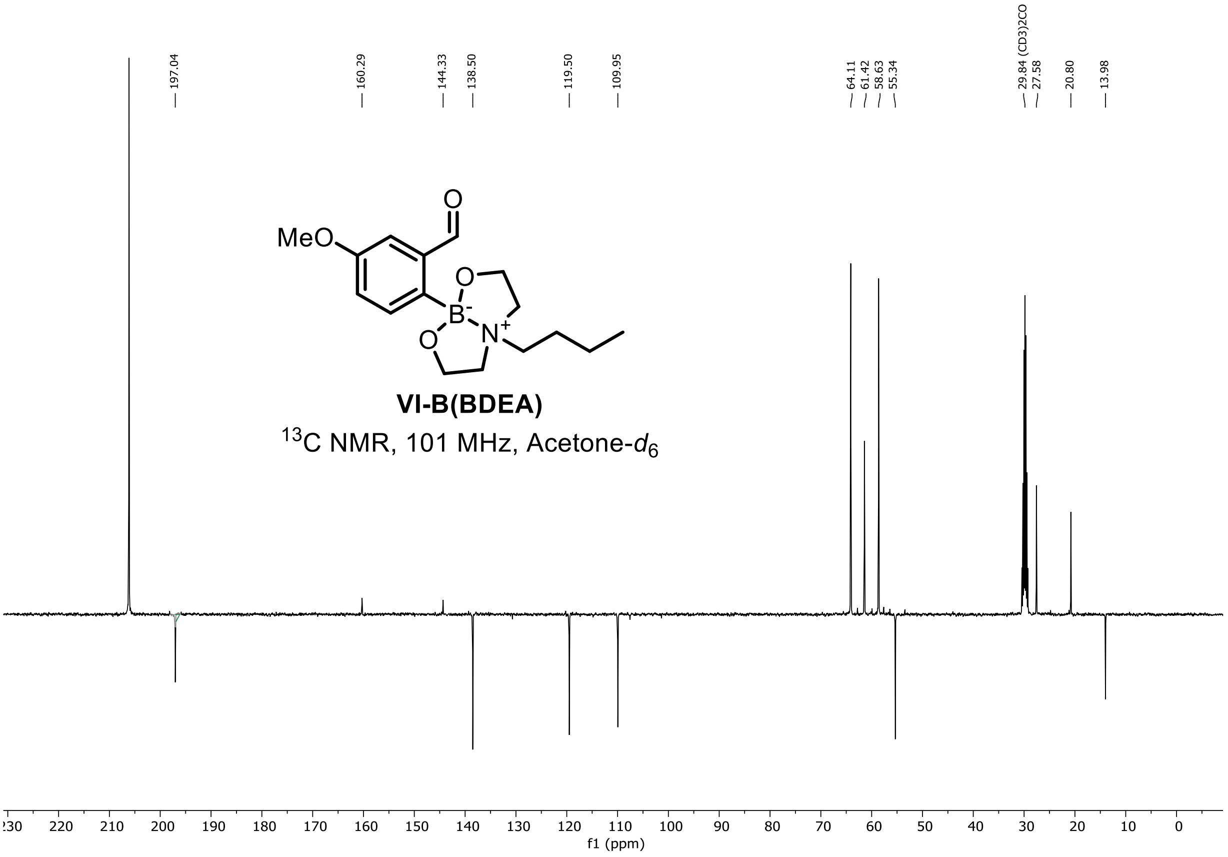

^{11}B NMR (128 MHz, Acetone-d_6) of **VI-B(BDEA)**

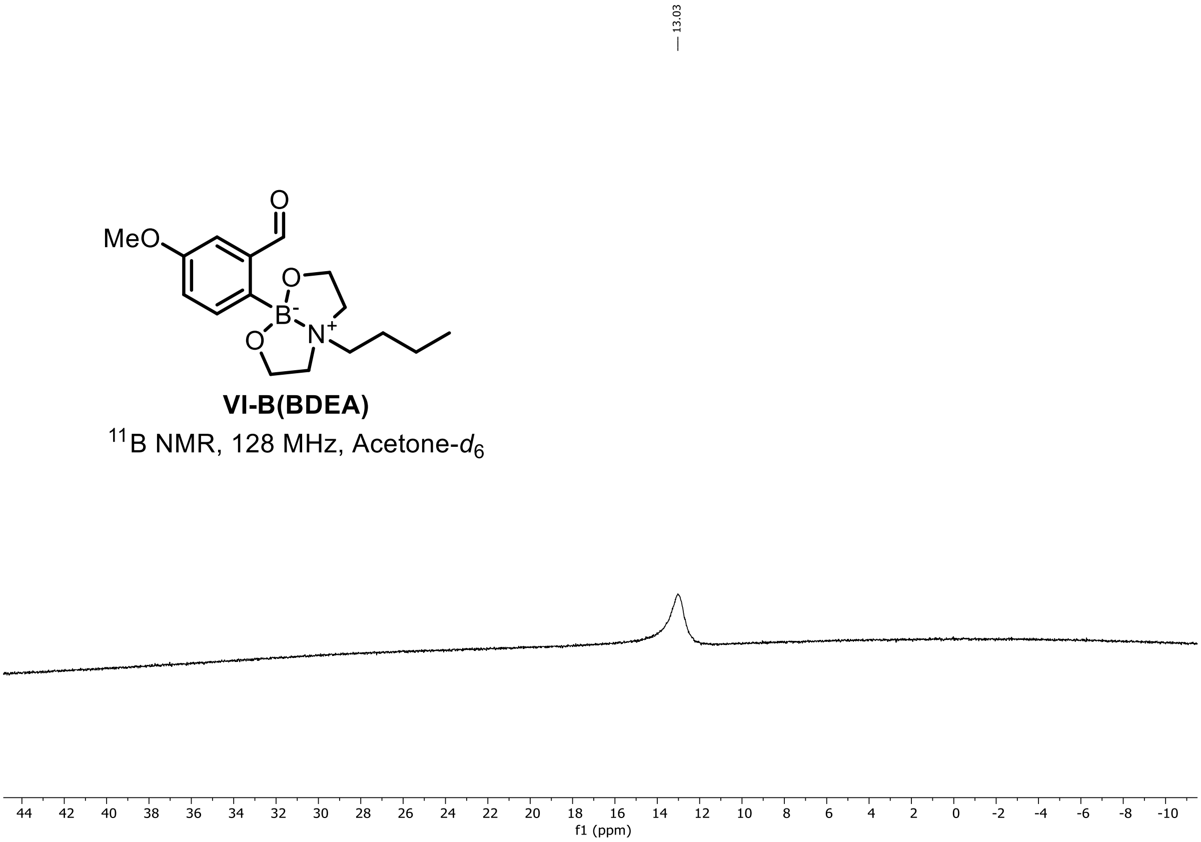

^{1}H NMR (400 MHz, Acetone-d_6) of **VII-B(BDEA)** (see procedure)

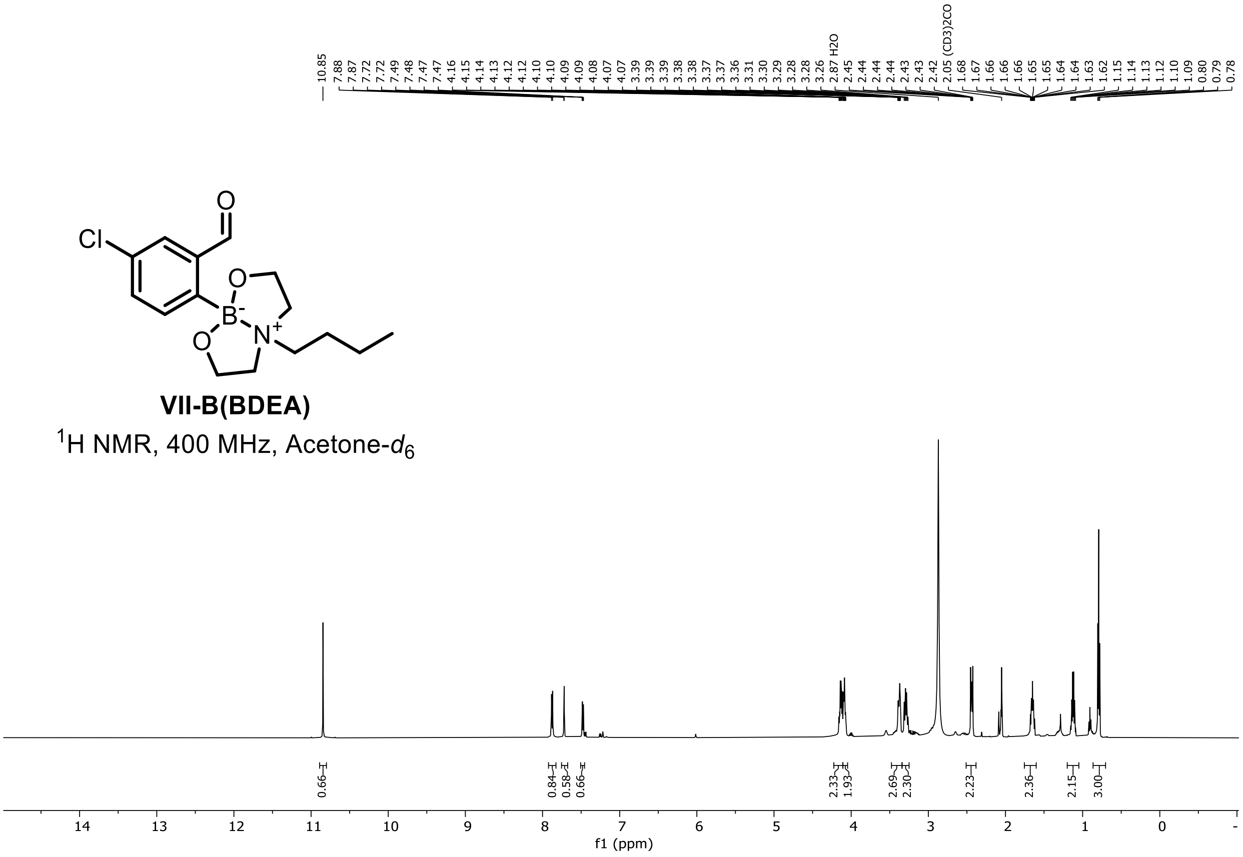

^{13}C NMR (101 MHz, Acetone-d_6) of **VII-B(BDEA)**

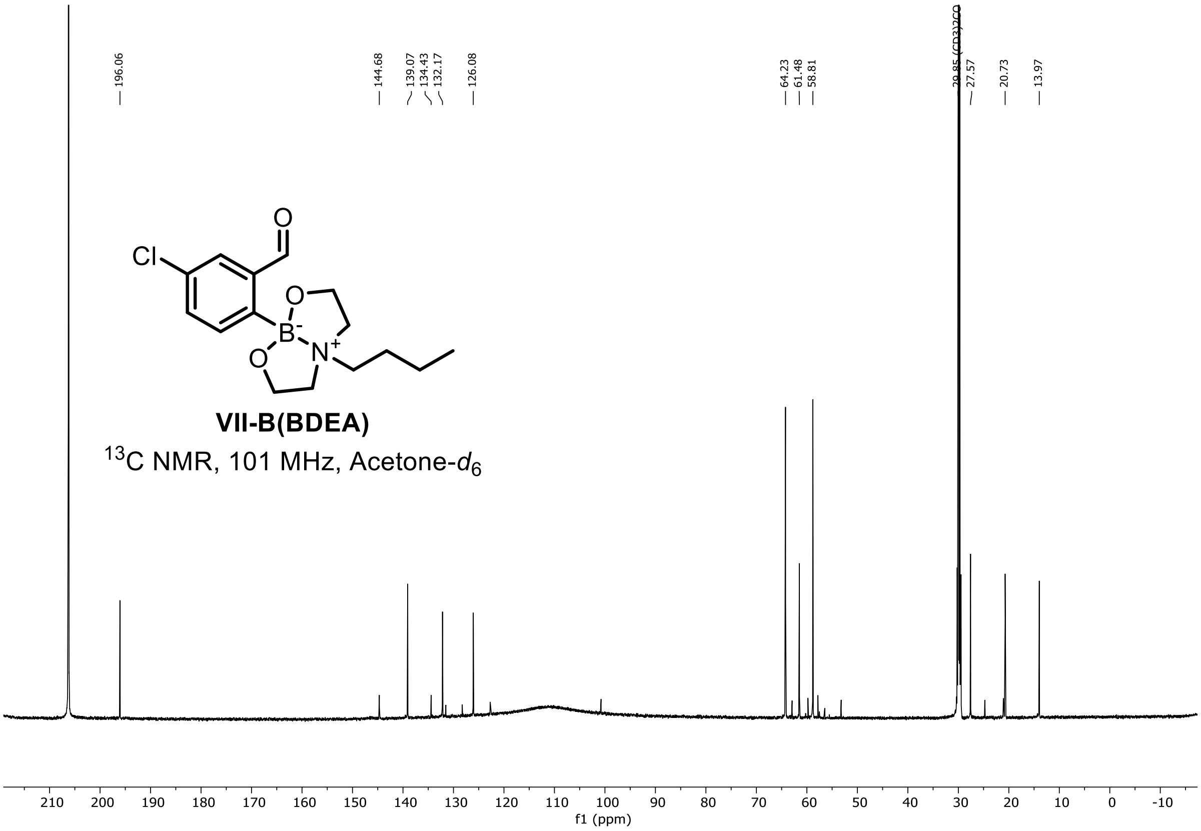

^{11}B NMR (128 MHz, Acetone-d_6) of **VII-B(BDEA)**

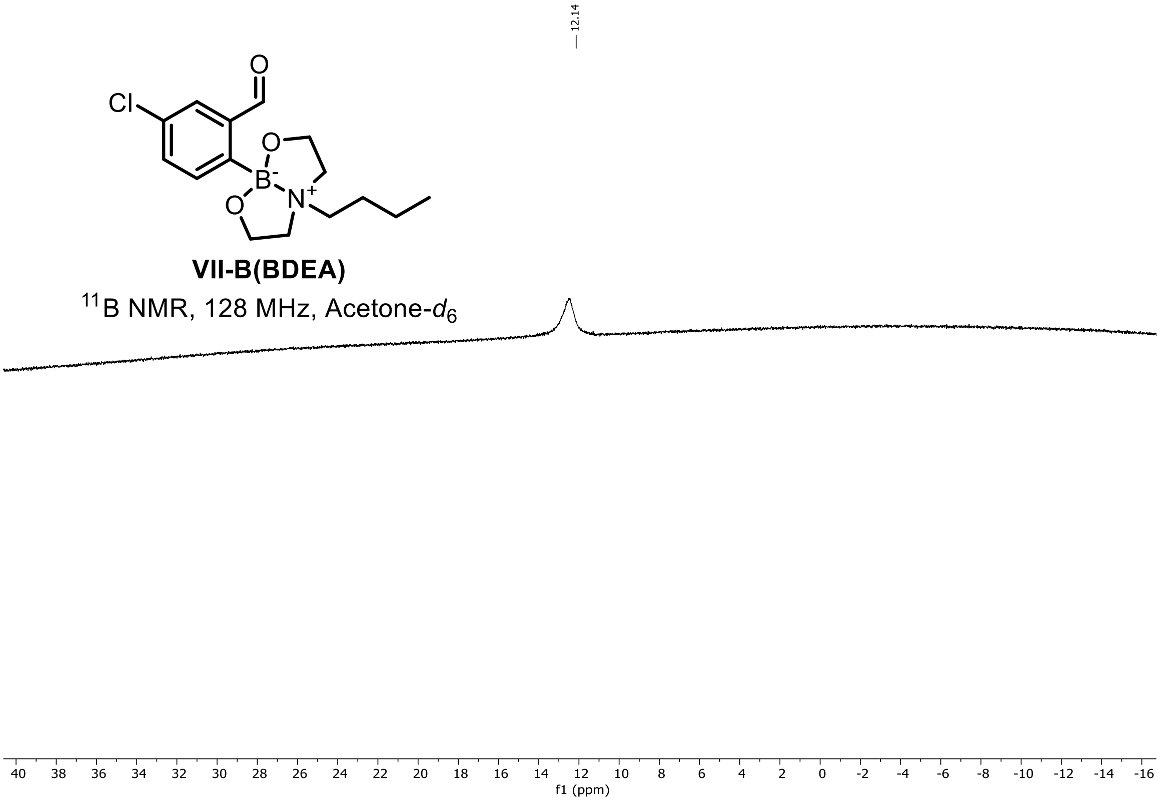

^{1}H NMR (400 MHz, Acetone-d_6) of **VIII-B(BDEA)** (see procedure)

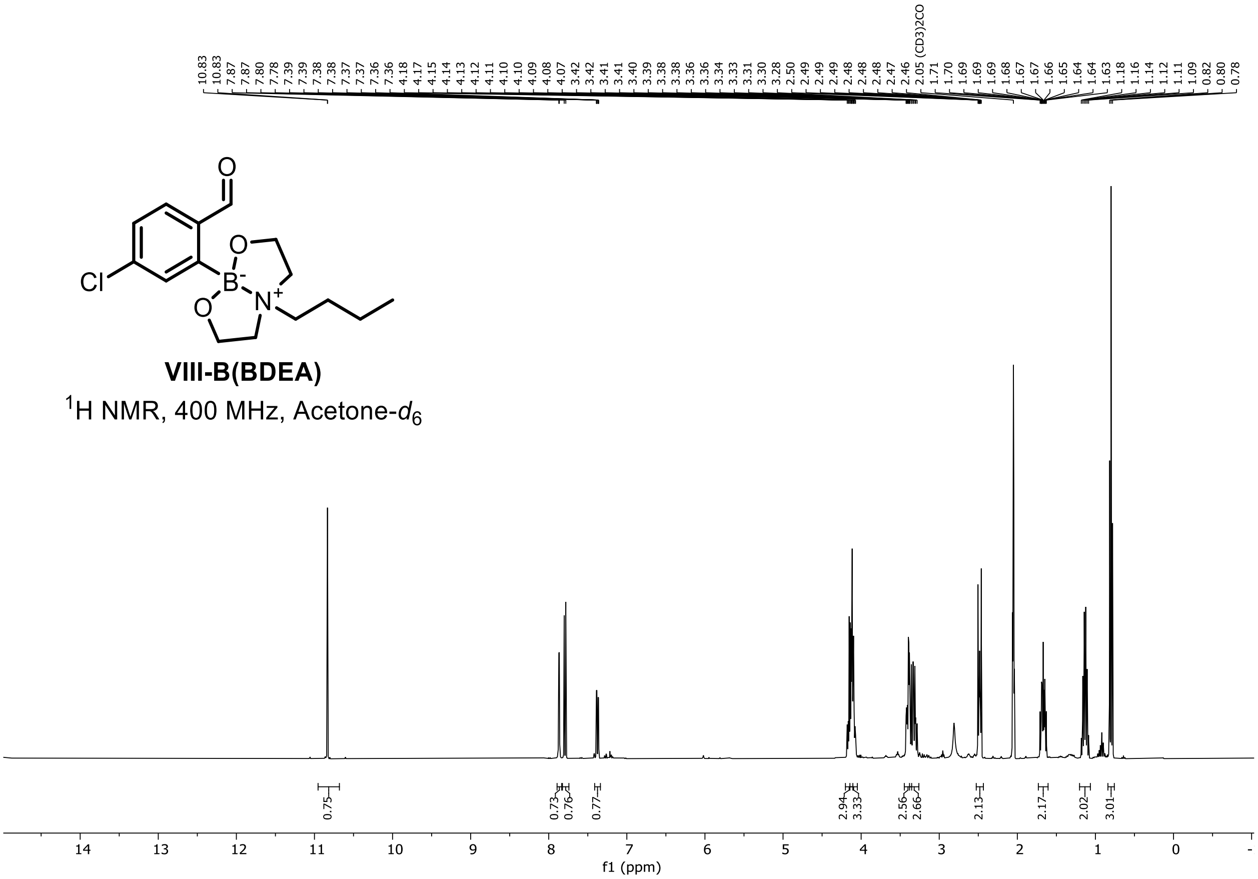

^{13}C NMR (101 MHz, Acetone-d_6) of **VIII-B(BDEA)**

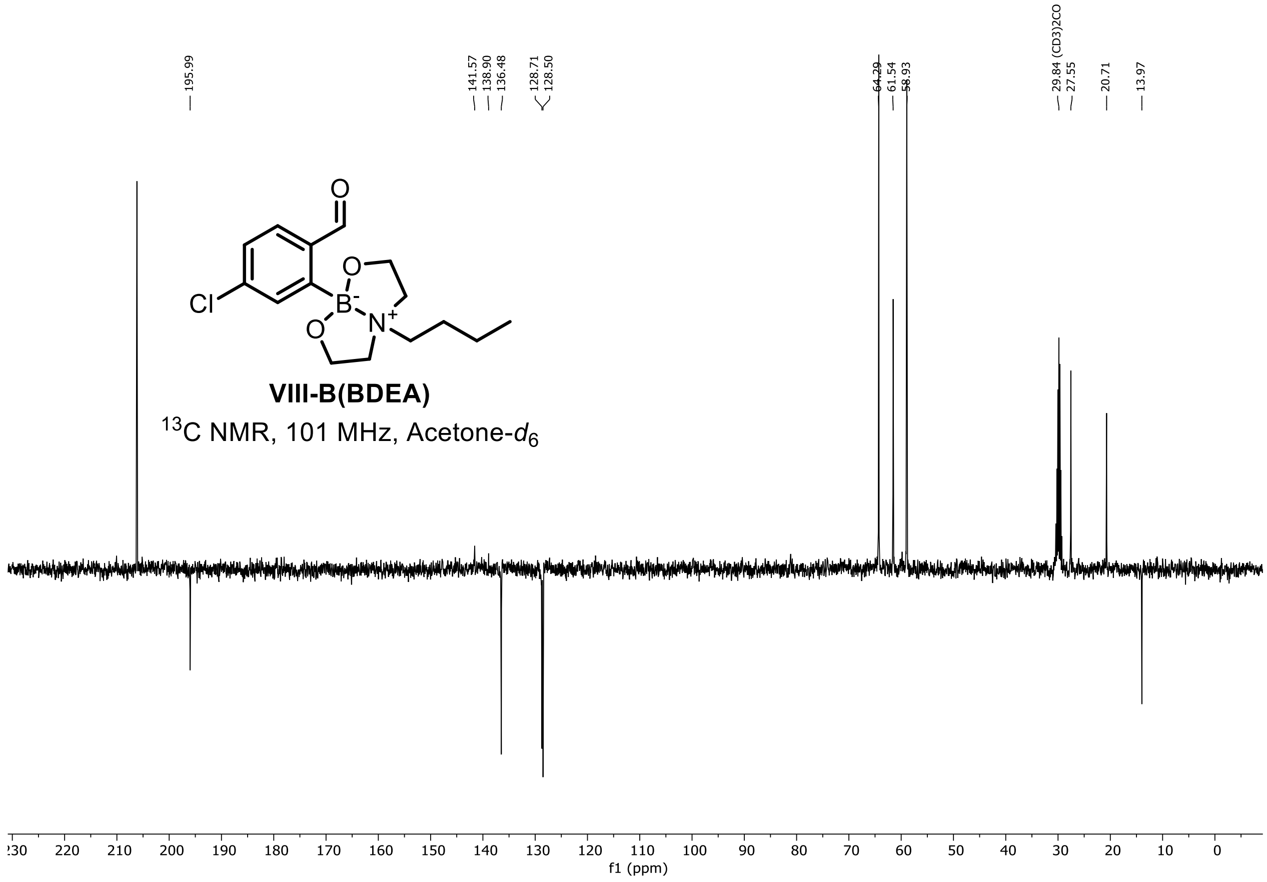

^{11}B NMR (128 MHz, Acetone-d_6) of **VIII-B(BDEA)**

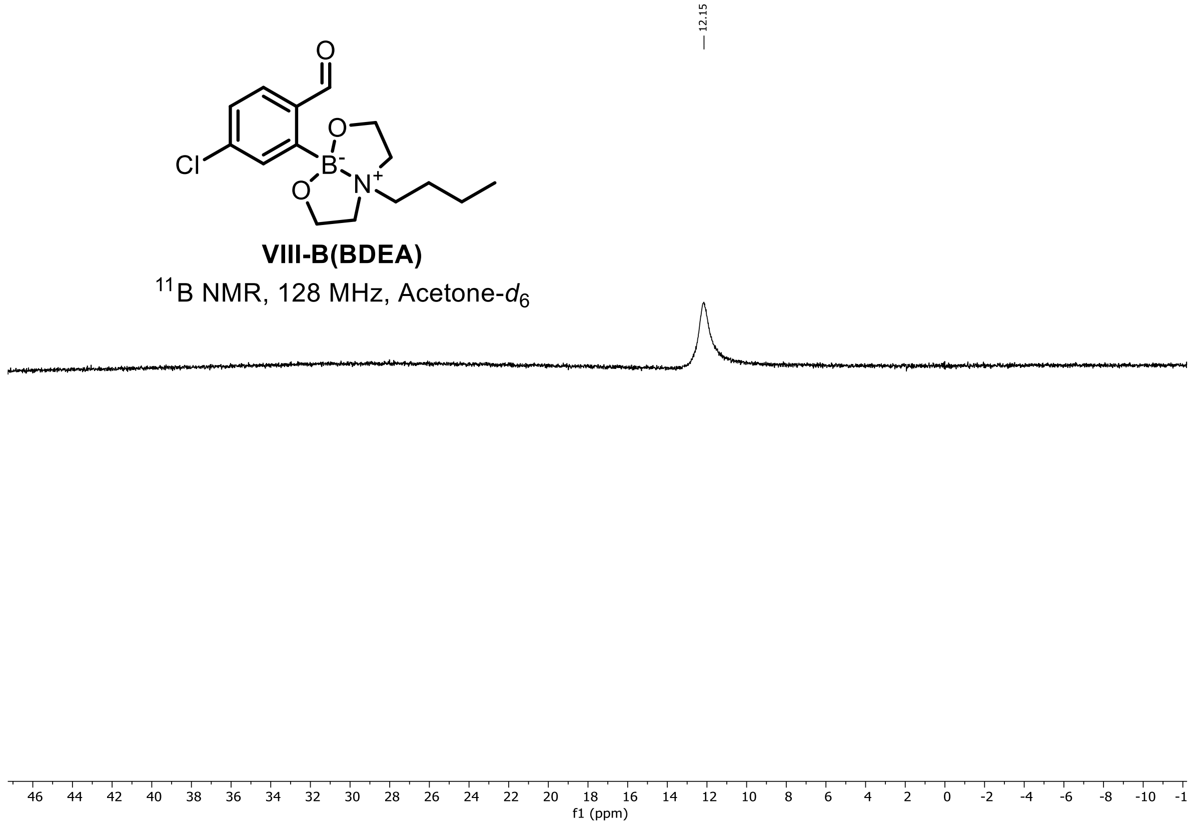

^{1}H NMR (400 MHz, Acetone-d_6) of **IX-B(BDEA)** (see procedure)

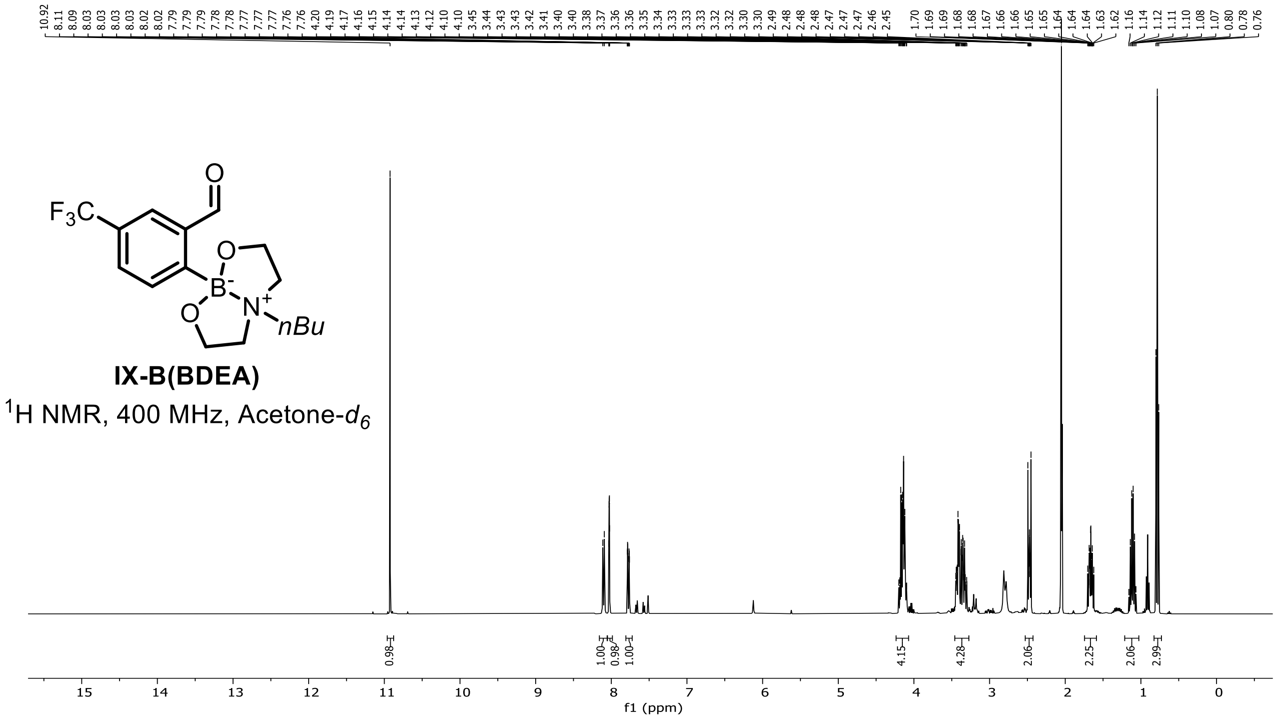

^{13}C NMR (101 MHz, Acetone-d_6) of **IX-B(BDEA)**

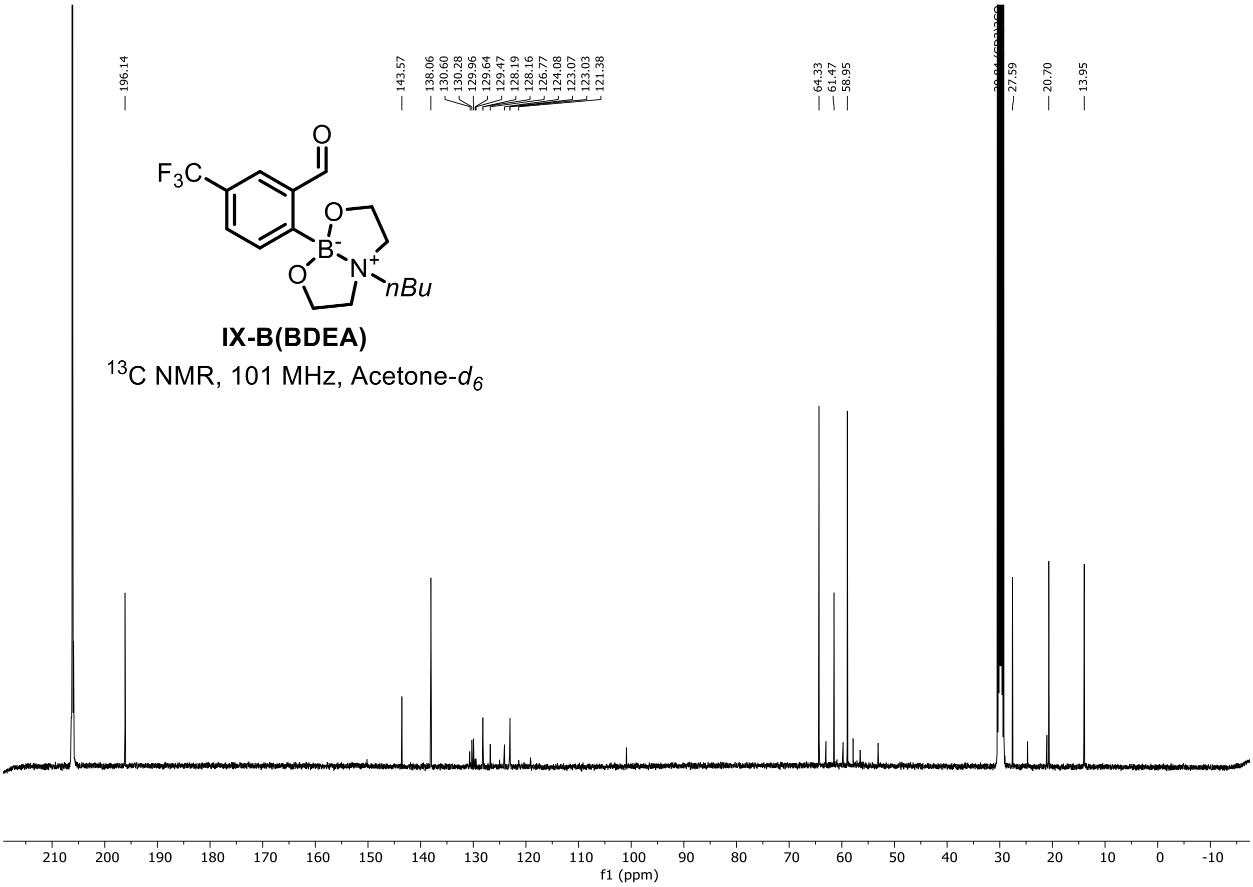

^{11}B NMR (128 MHz, Acetone-d_6) of **IX-B(BDEA)**

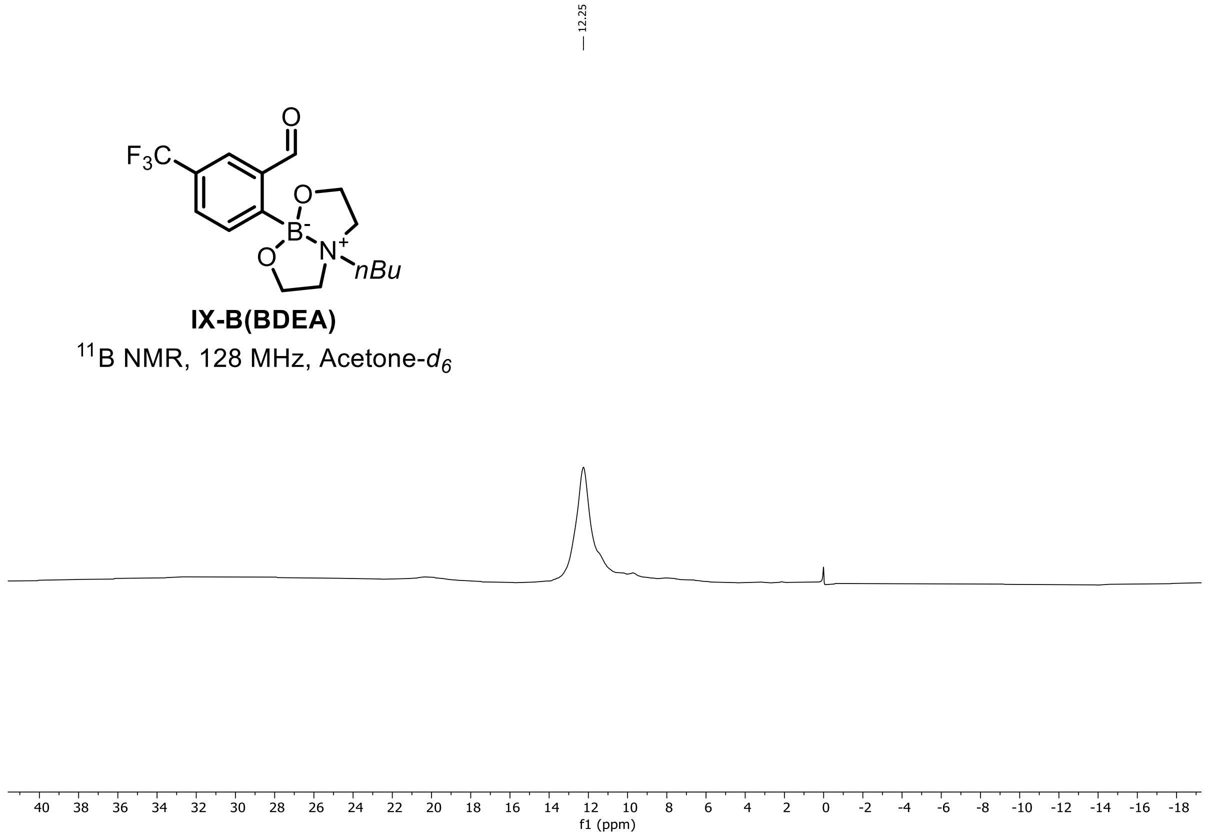

^{19}F NMR (376 MHz, Acetone-d_6) of **IX-B(BDEA)**

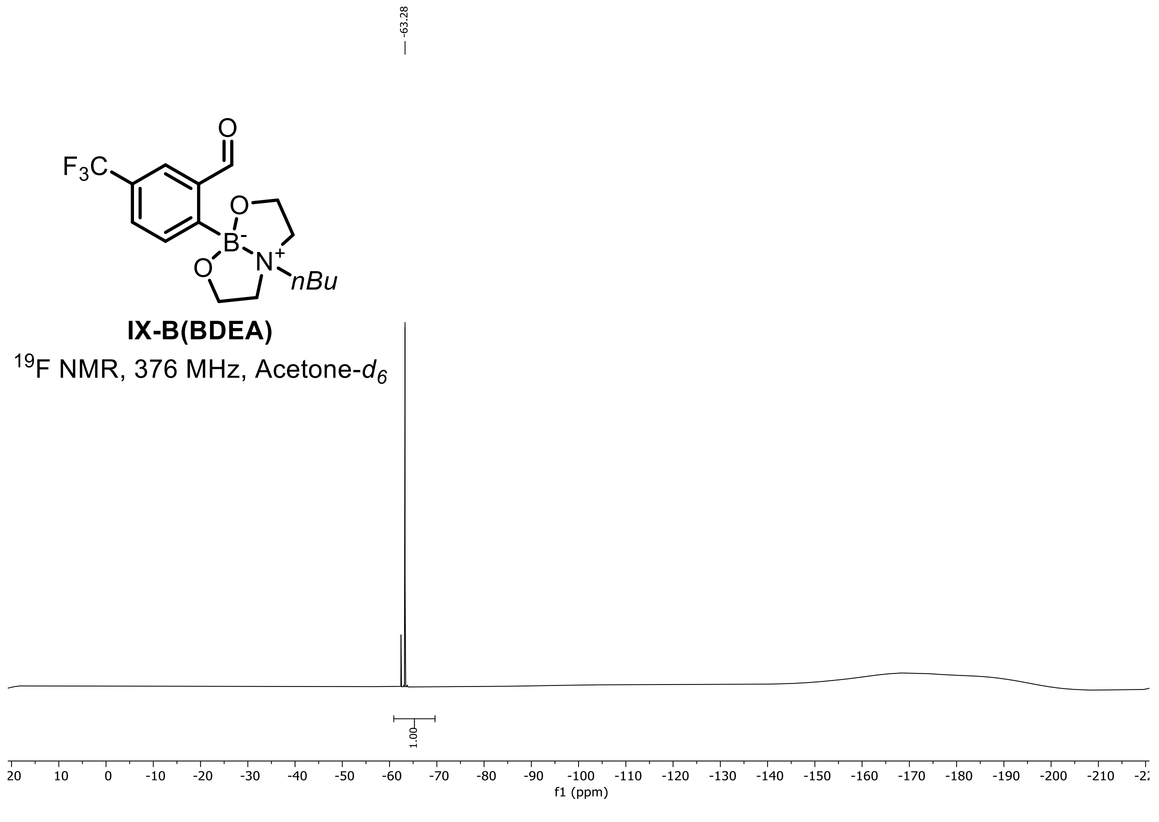

^{1}H NMR (400 MHz, Acetone-d_6) of **X-B(BDEA)** (see procedure)

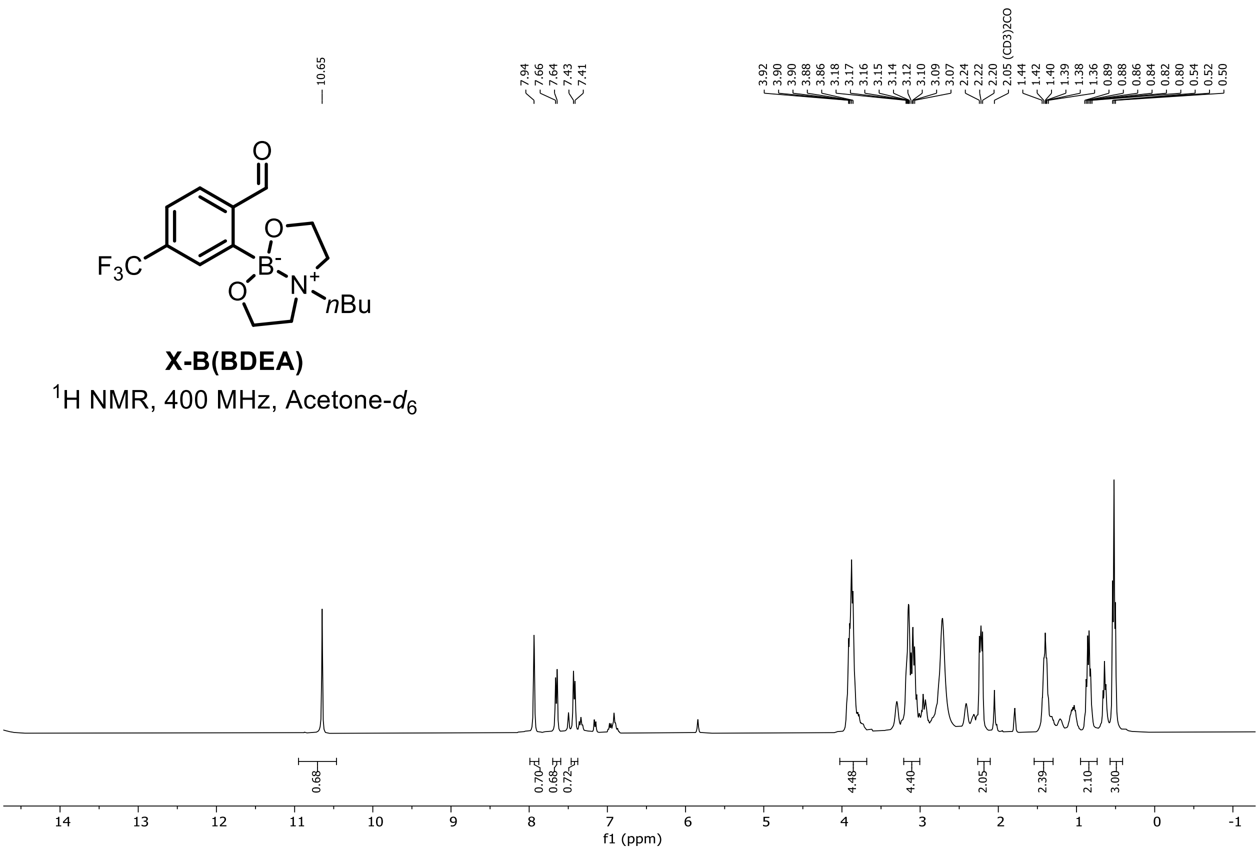

^{13}C NMR (101 MHz, Acetone-d_6) of **X-B(BDEA)**

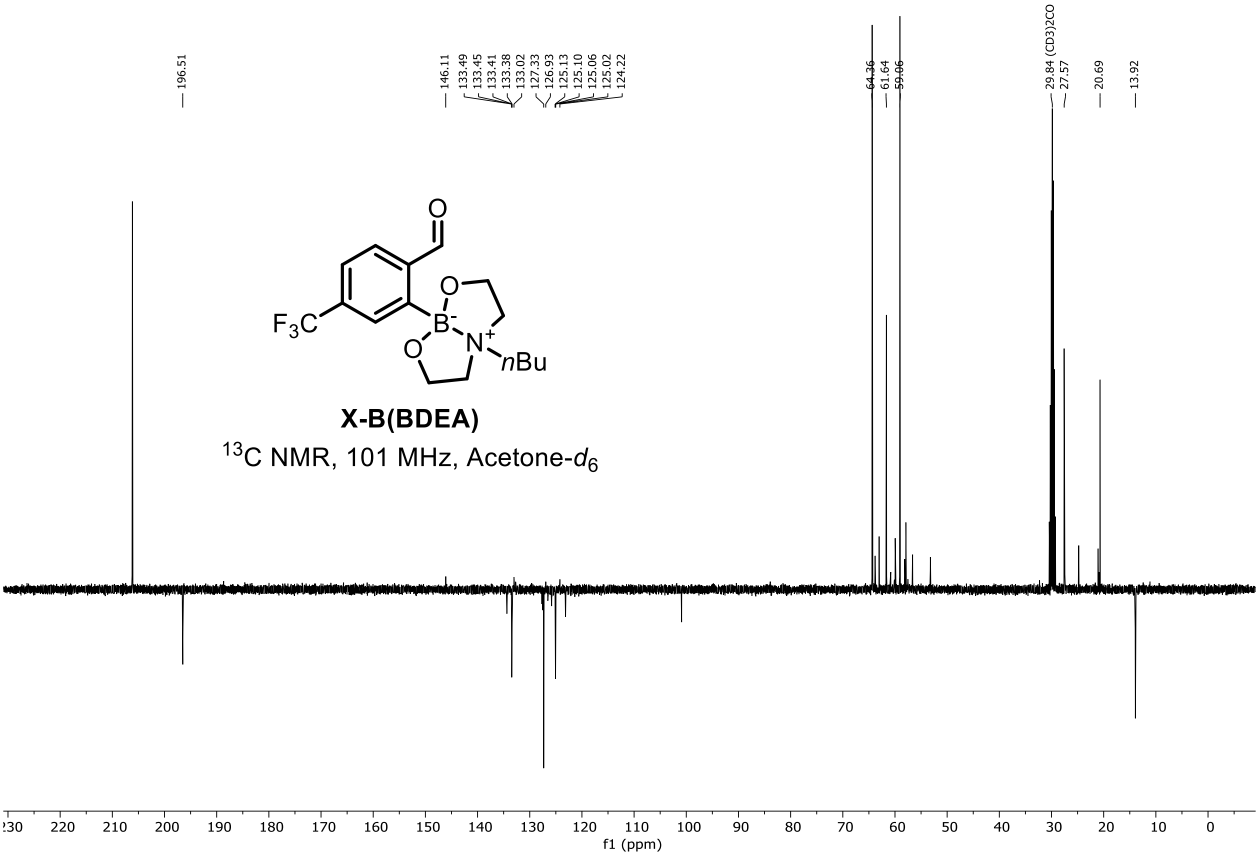

^{11}B NMR (128 MHz, Acetone-d_6) of **X-B(BDEA)**

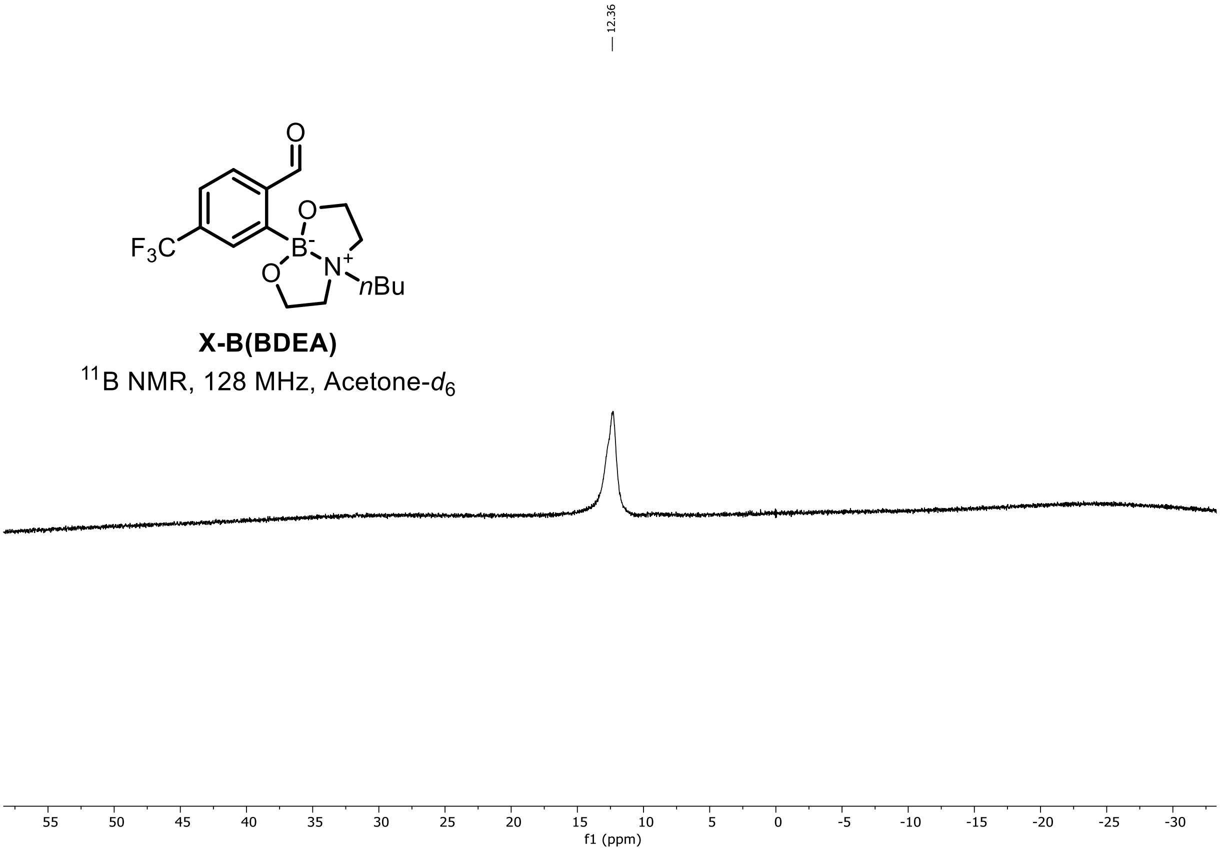

^{19}F NMR (376 MHz, Acetone-d_6) of **X-B(BDEA)**

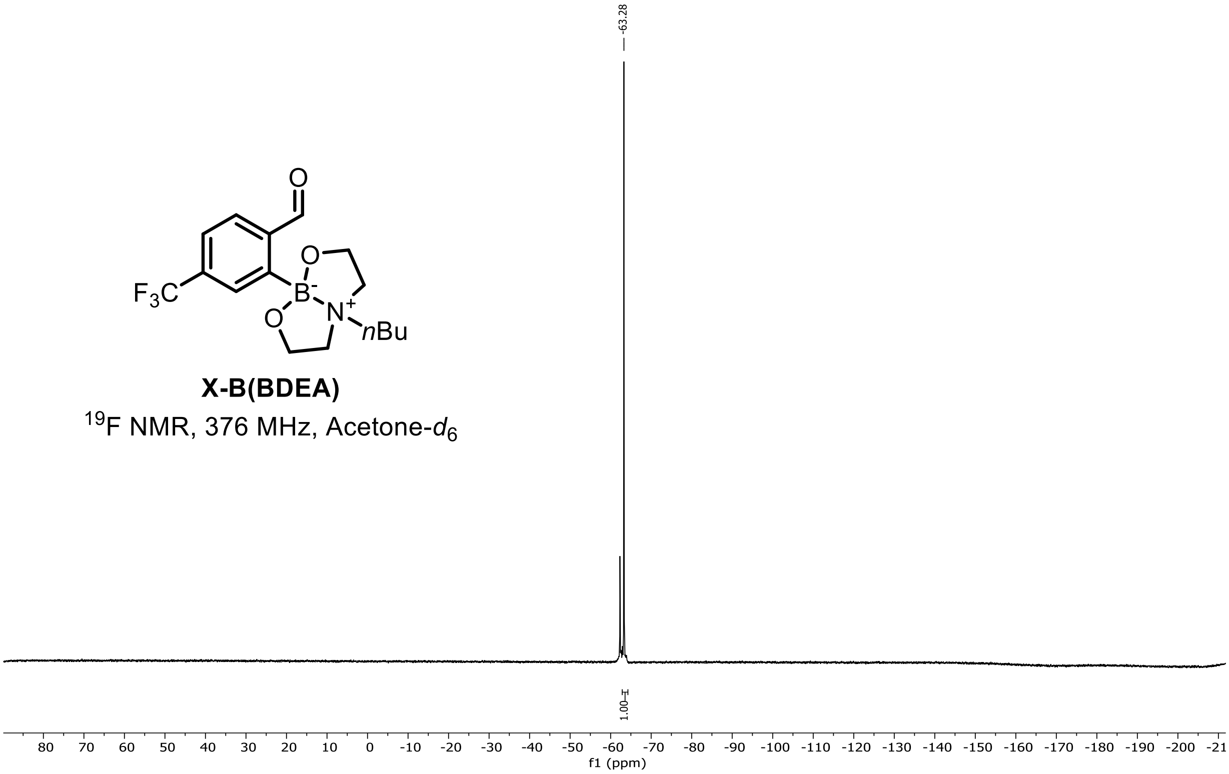

^{1}H NMR (400 MHz, $CDCl_3$) of **1** (see procedure)

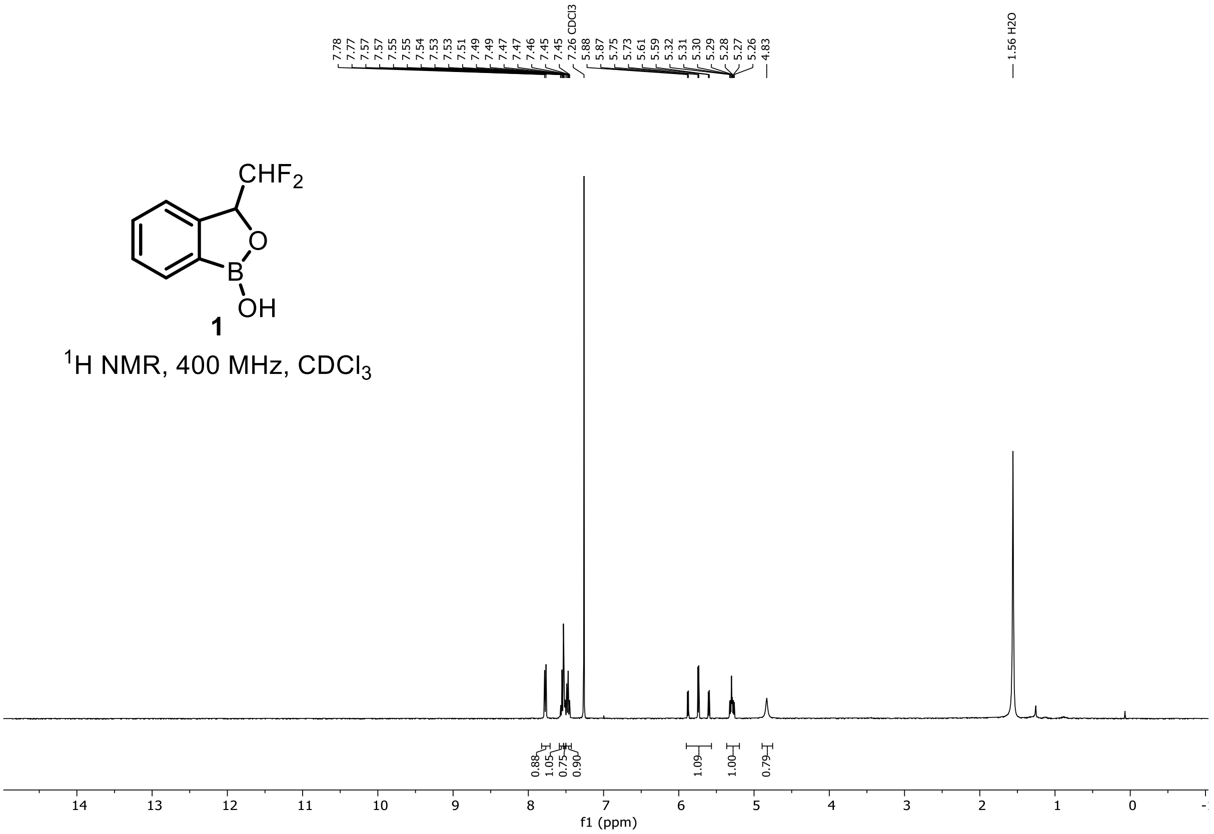

^{13}C NMR (101 MHz, $CDCl_3$) of **1**

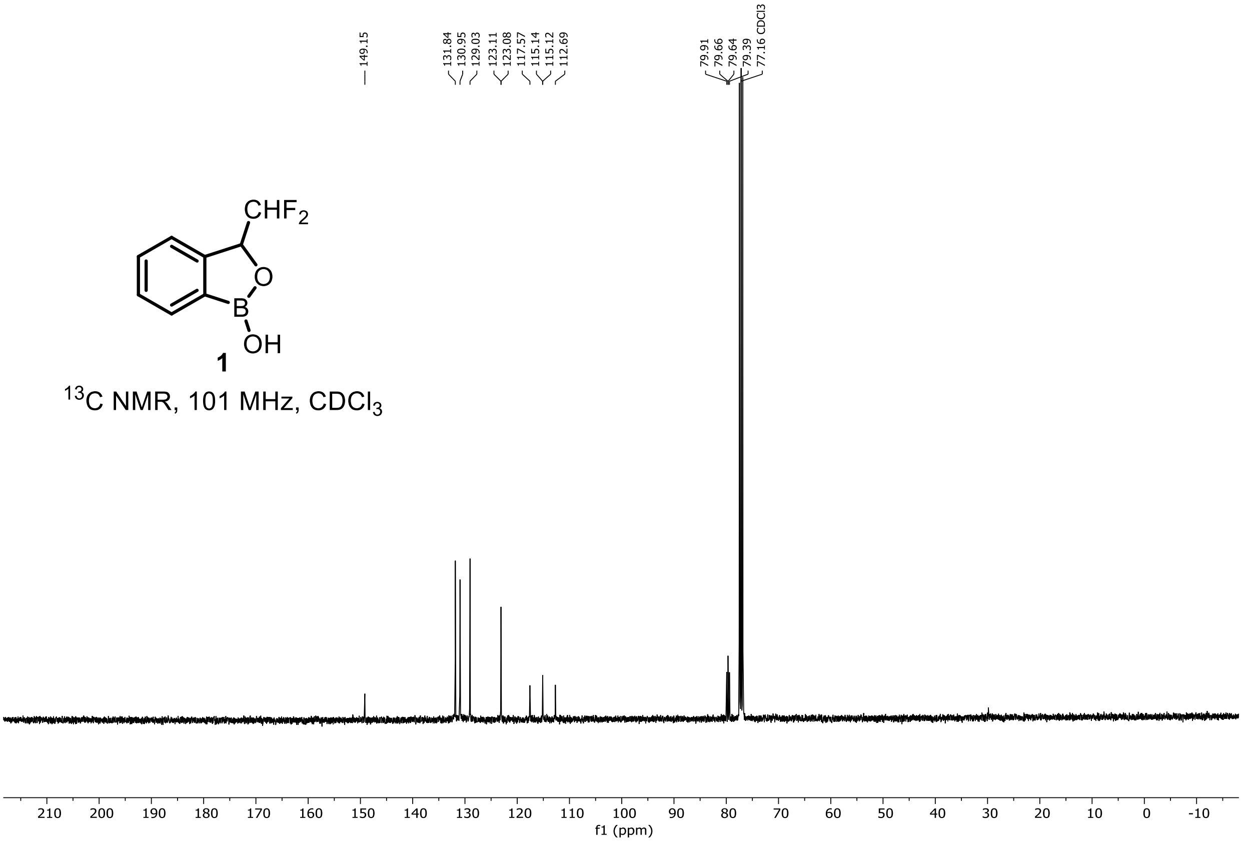

^{11}B NMR (128 MHz, $CDCl_3$) of **1**

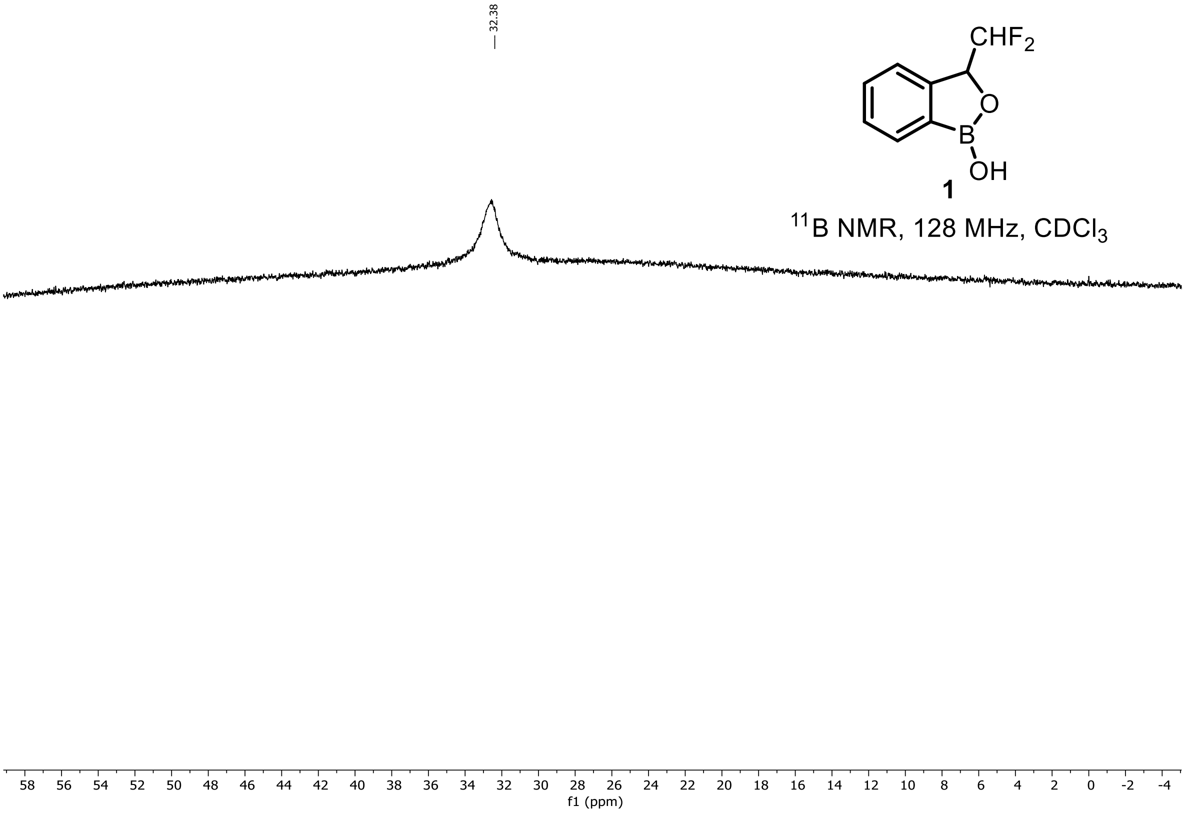

^{19}F NMR (376 MHz, $CDCl_3$) of **1**

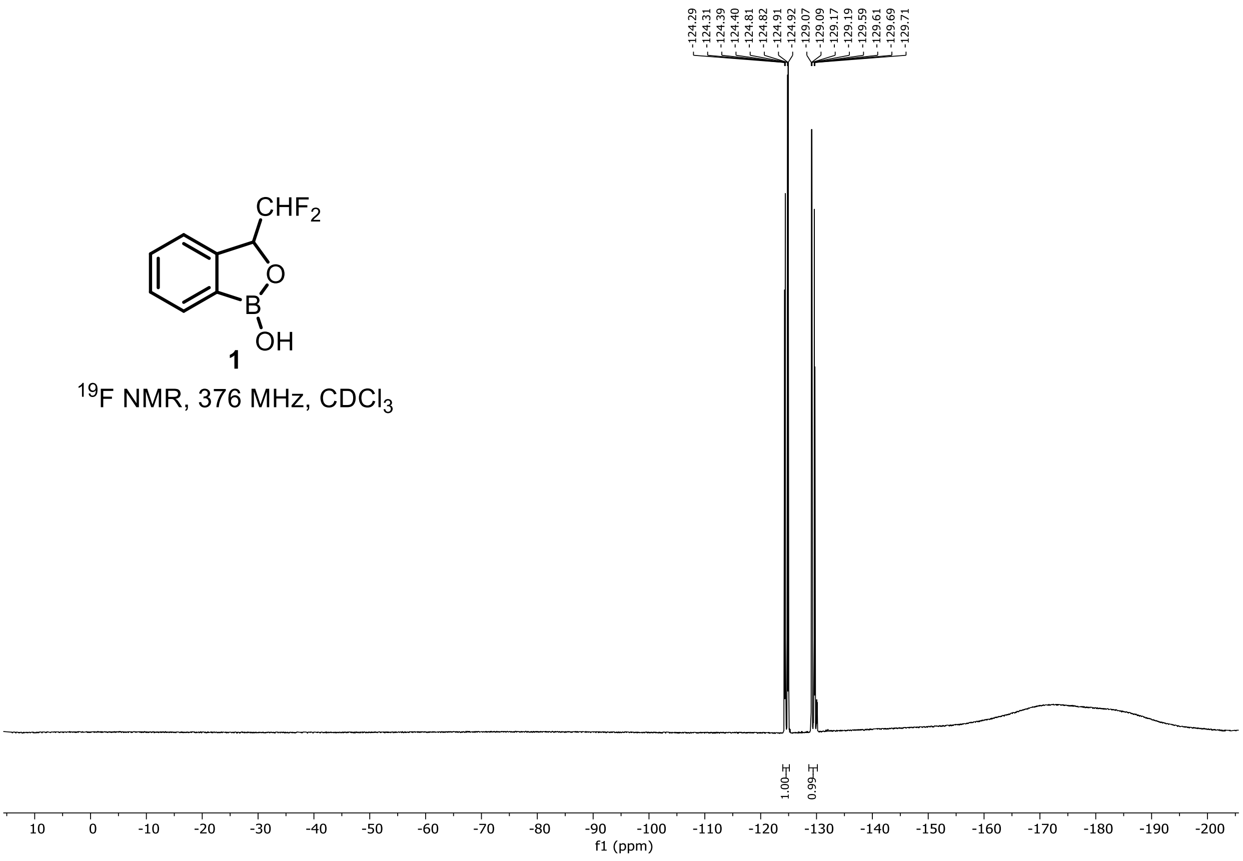

^{1}H NMR (400 MHz, $CDCl_3$) of **2** (see procedure)

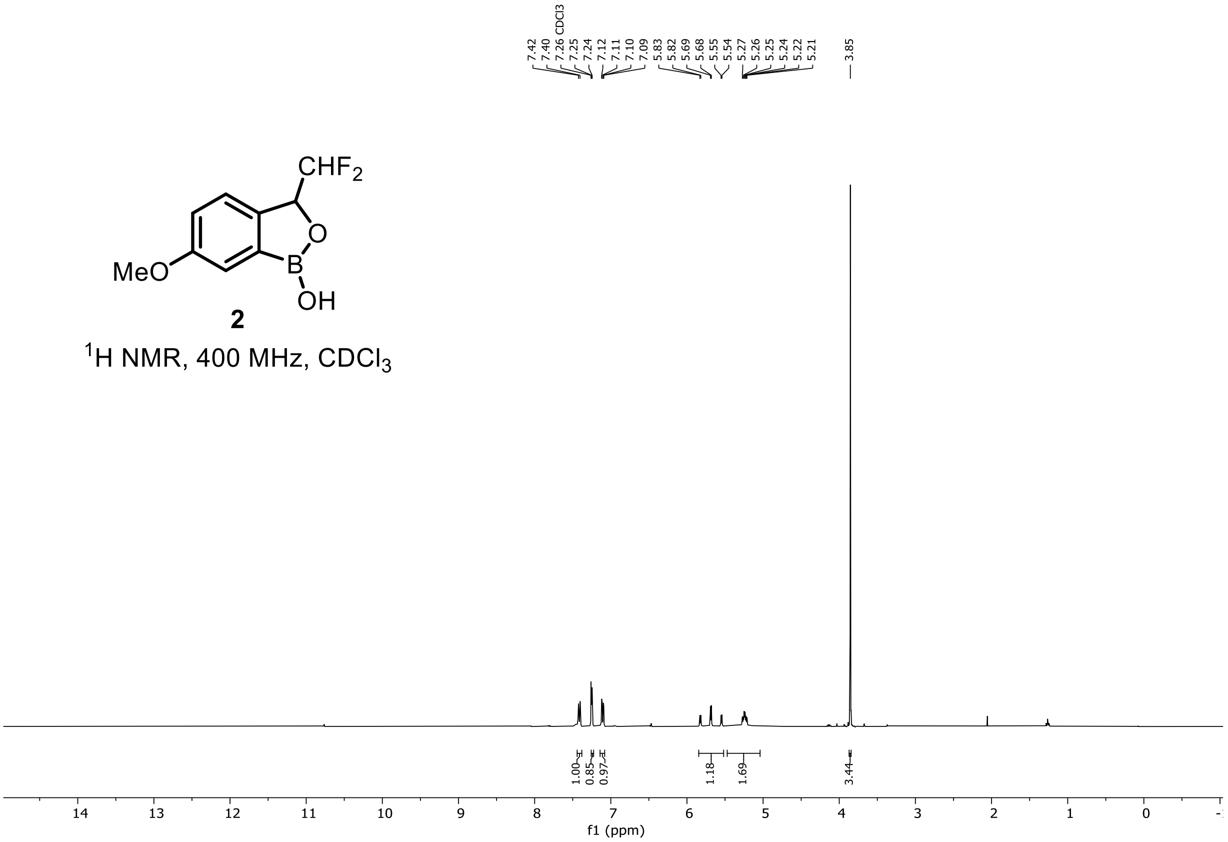

^{13}C NMR (101 MHz, $CDCl_3$) of **2**

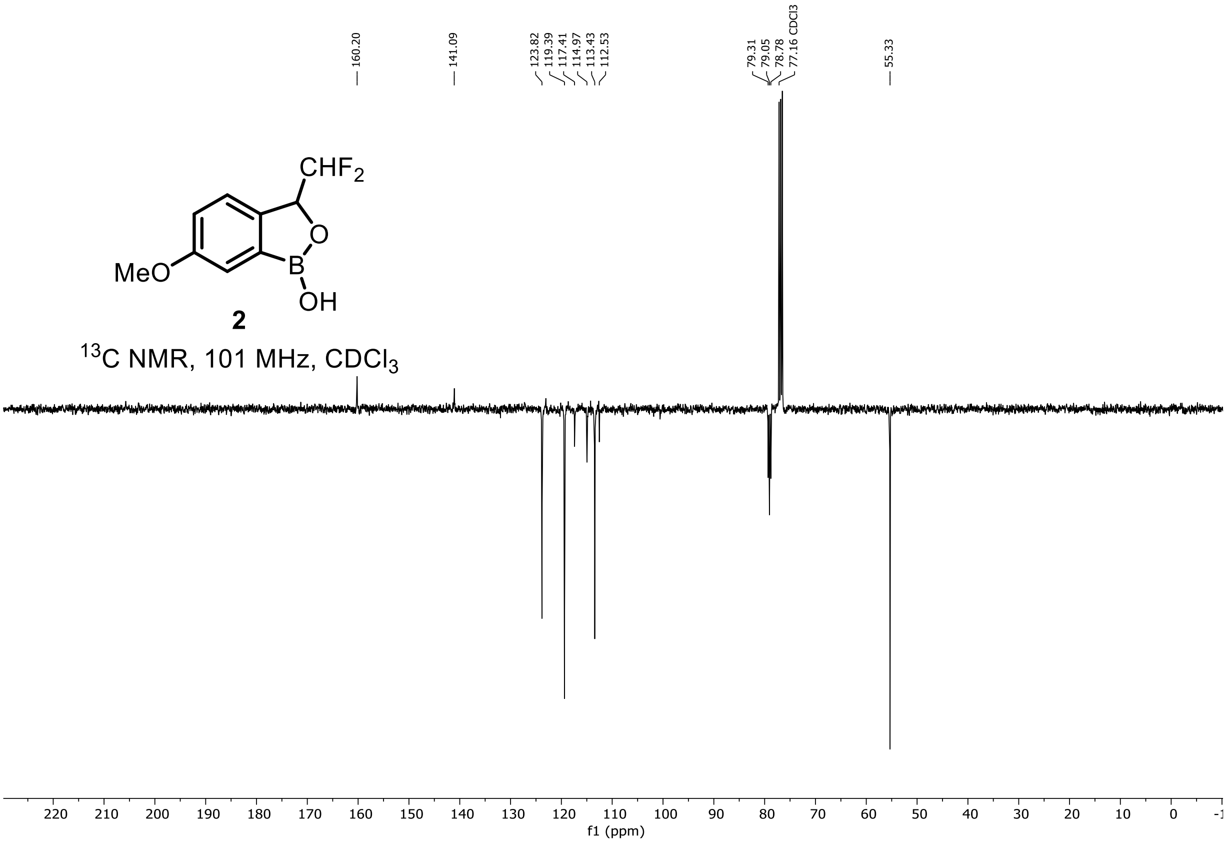

^{11}B NMR (128 MHz, $CDCl_3$) of **2**

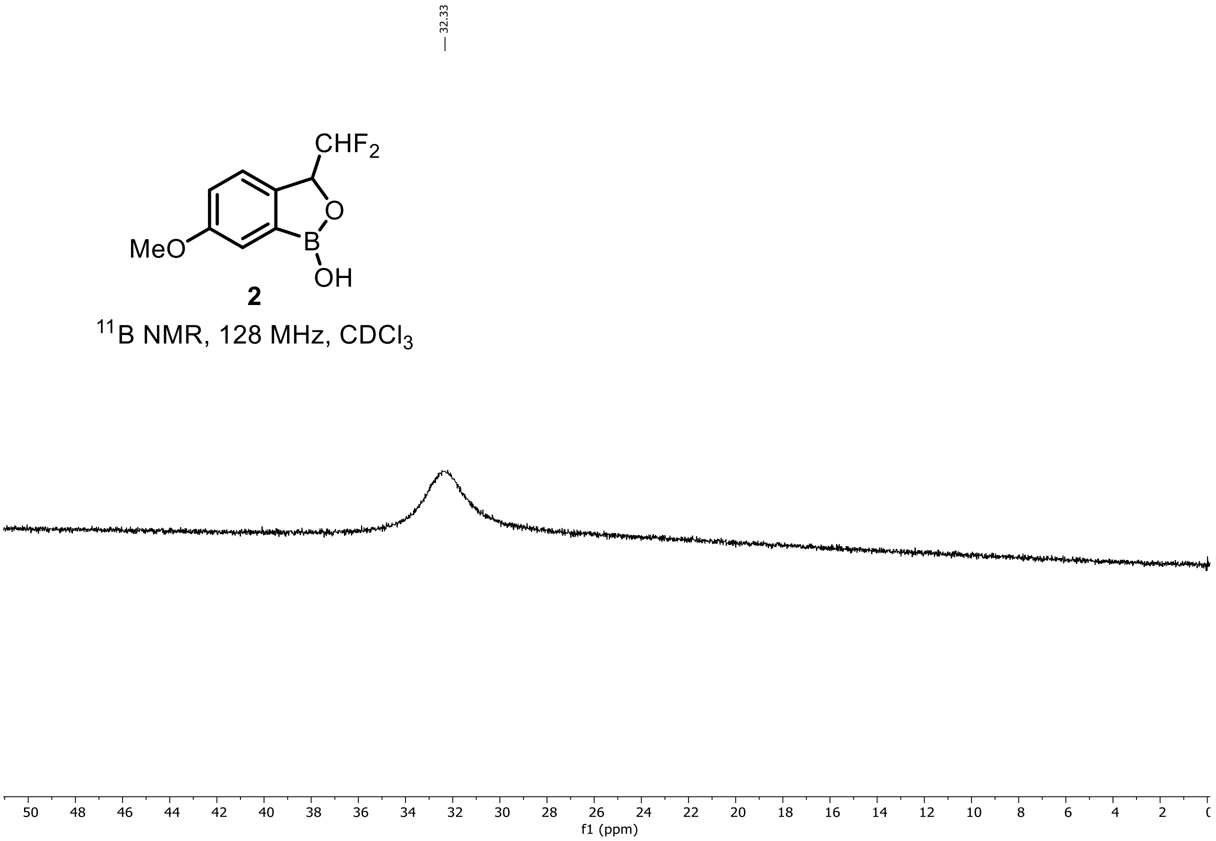

^{19}F NMR (376 MHz, $CDCl_3$) of **2**

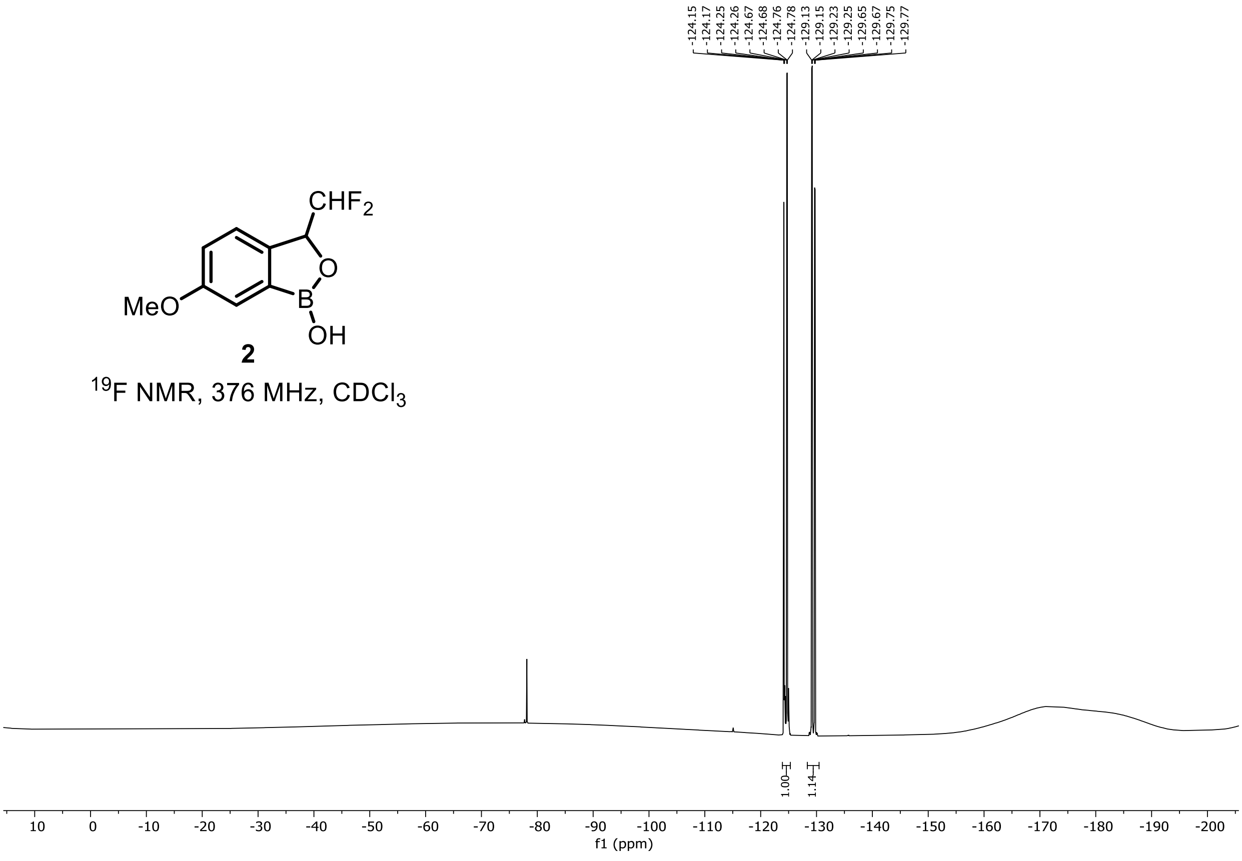

^{1}H NMR (400 MHz, $CDCl_3$) of **3** (see procedure)

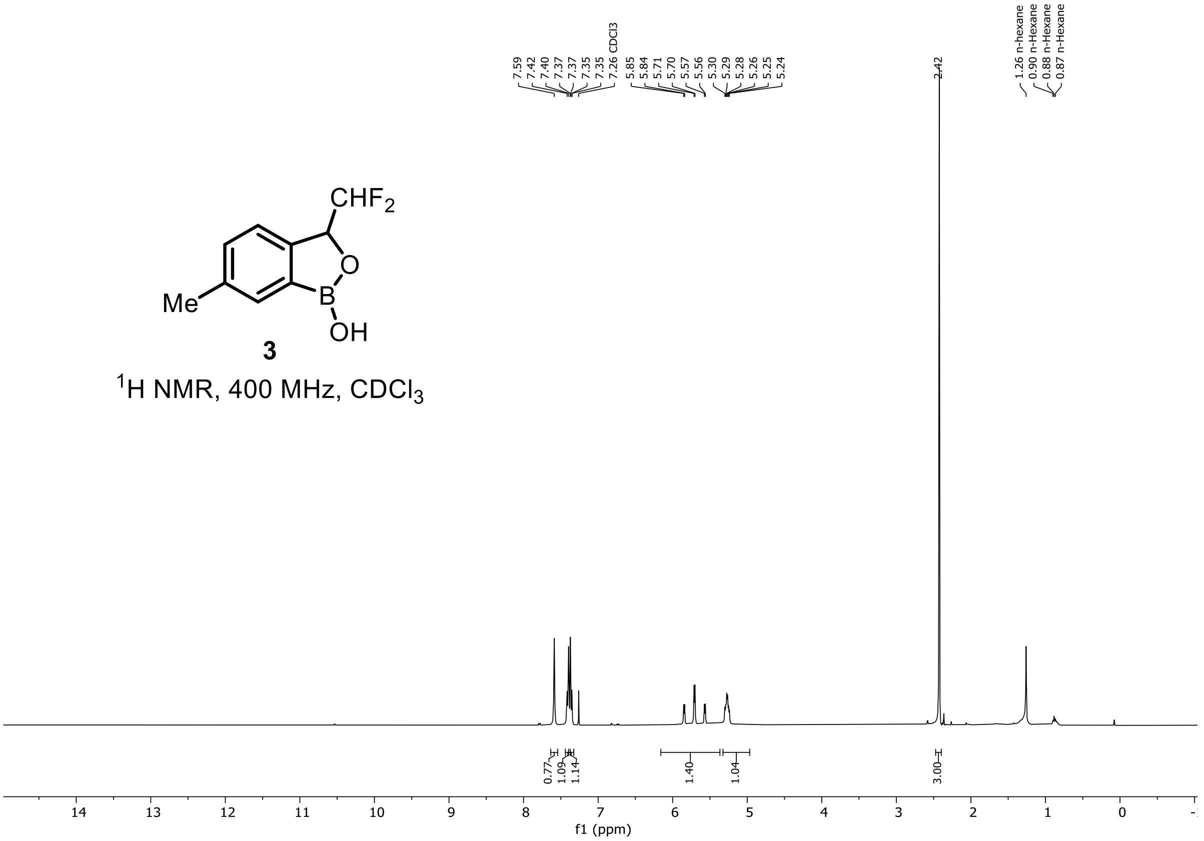

^{13}C NMR (101 MHz, $CDCl_3$) of **3**

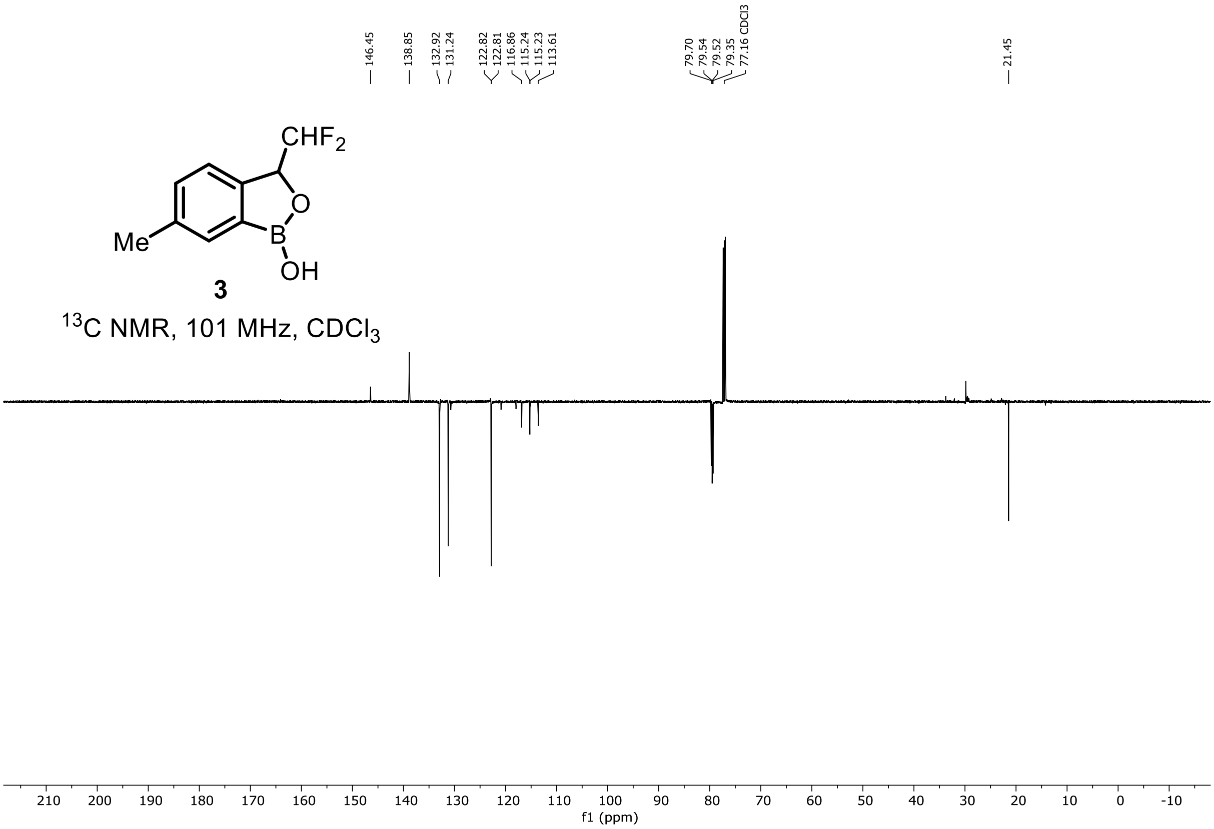

^{11}B NMR (128 MHz, $CDCl_3$) of **3**

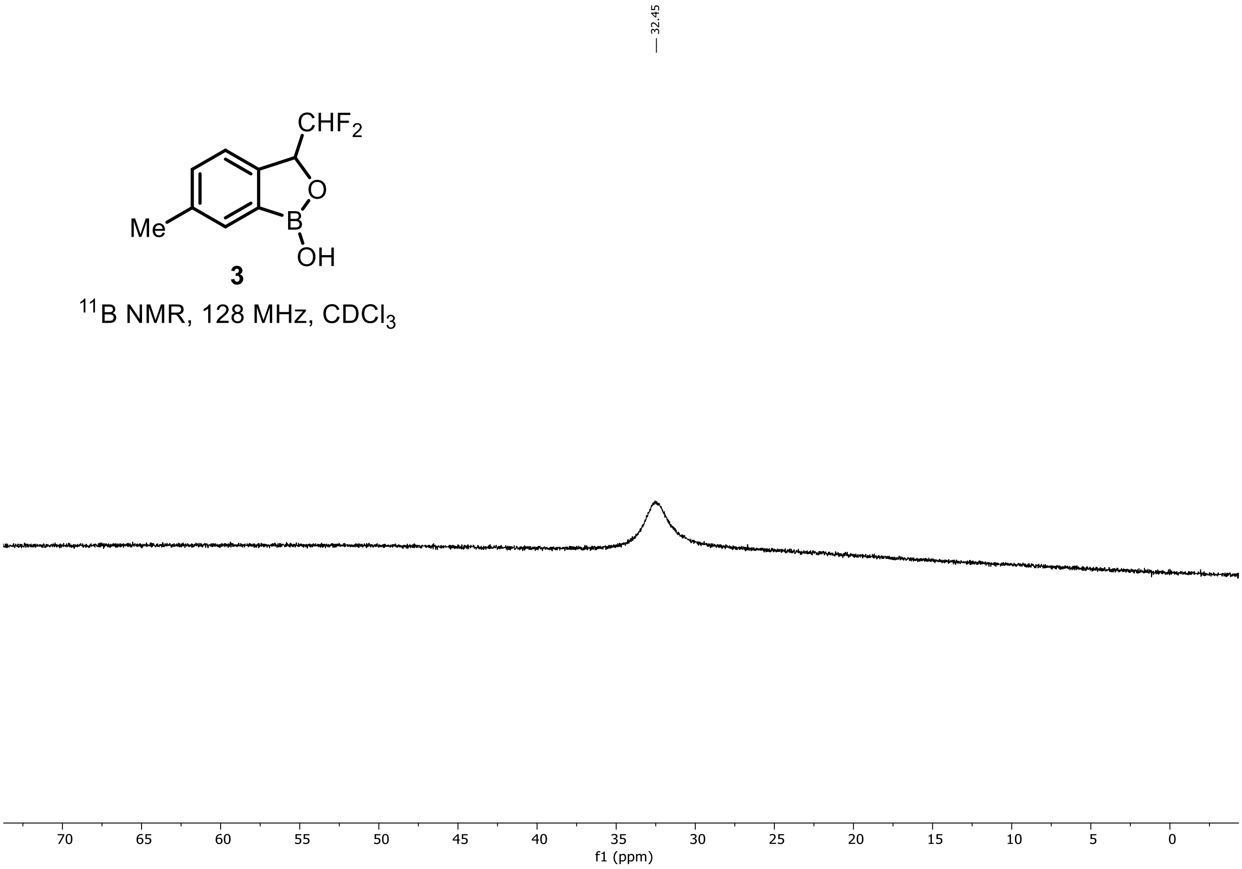

^{19}F NMR (376 MHz, $CDCl_3$) of **3**

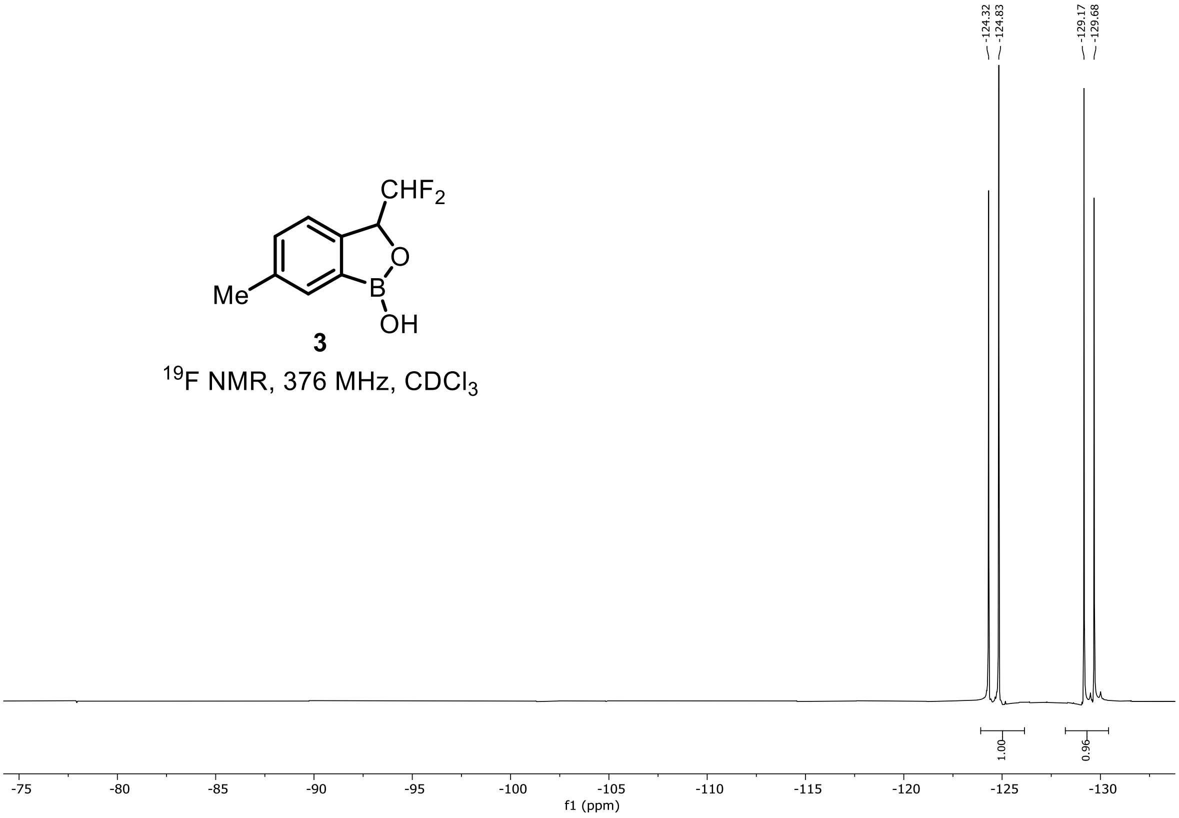

^{1}H NMR (400 MHz, $CDCl_3$) of **4** (see procedure)

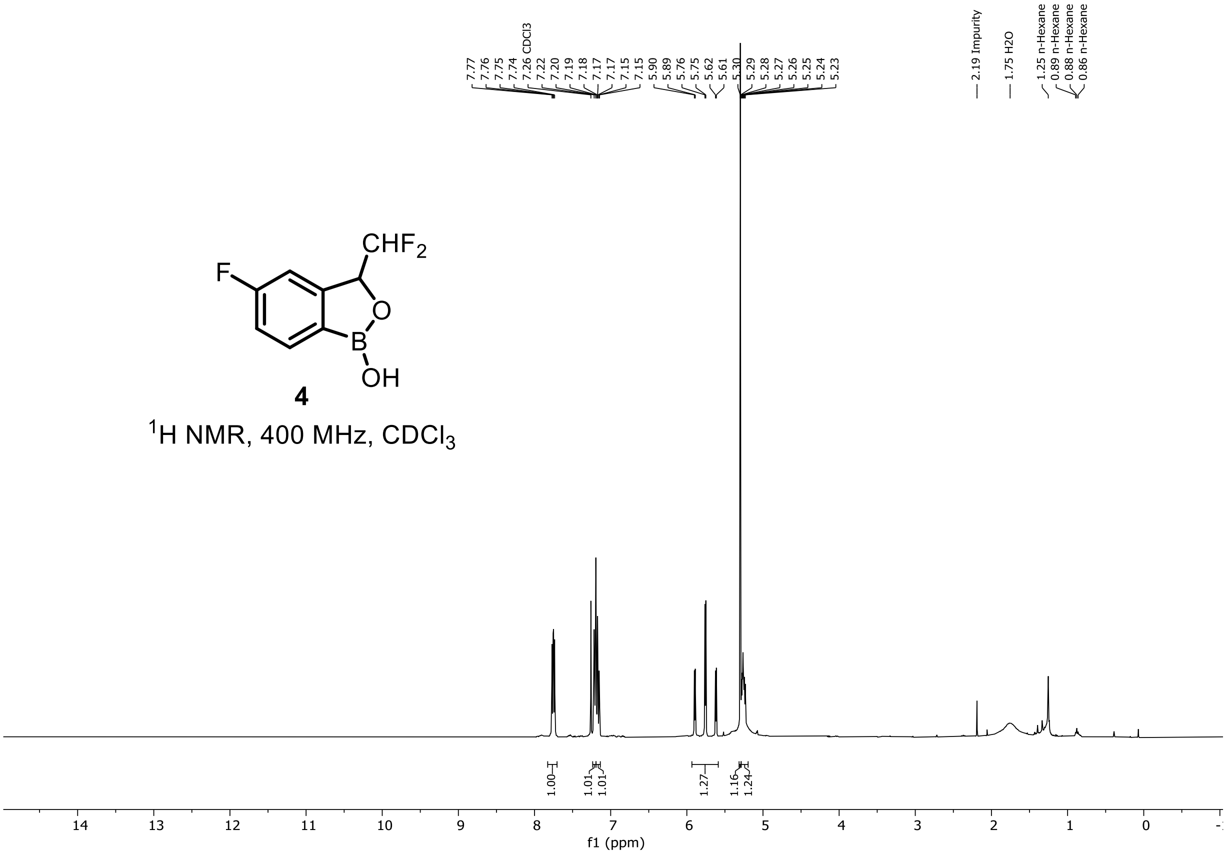

^{13}C NMR (101 MHz, $CDCl_3$) of **4**

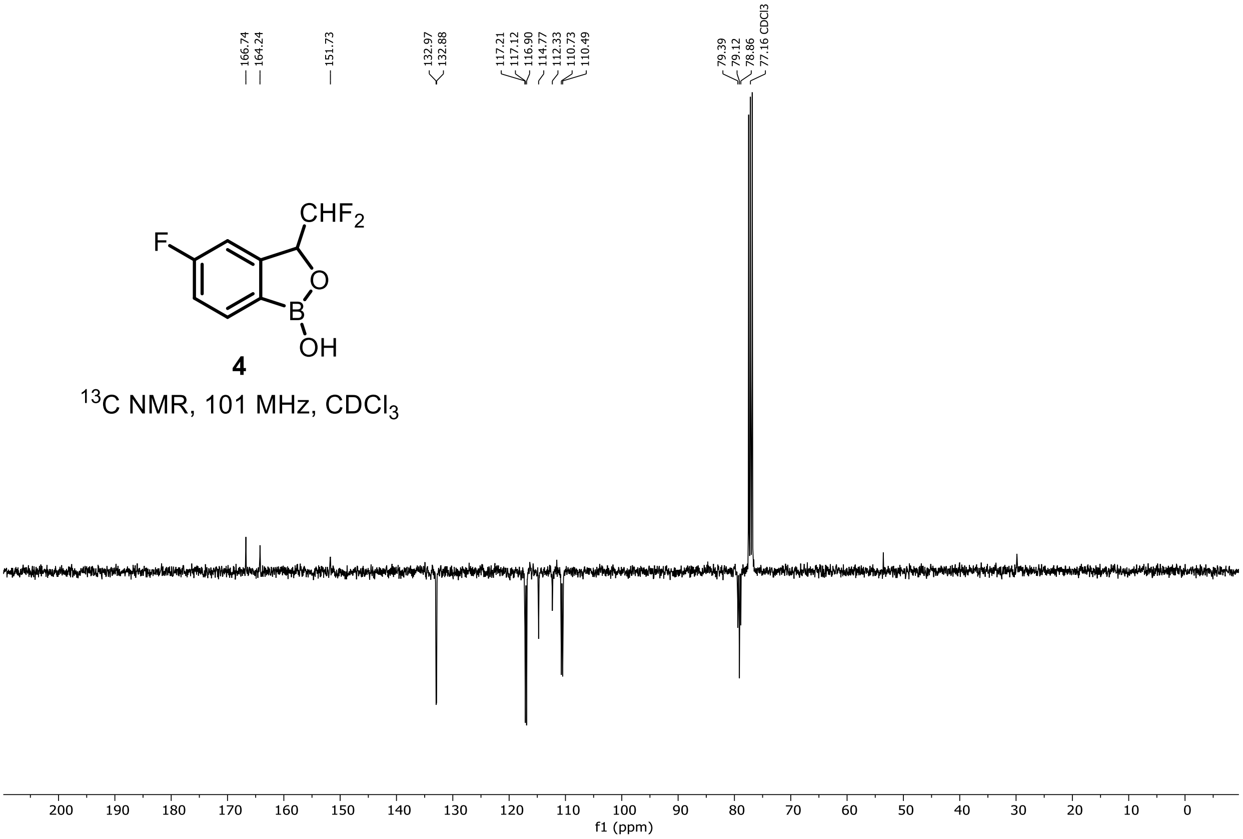

^{11}B NMR (128 MHz, $CDCl_3$) of **4**

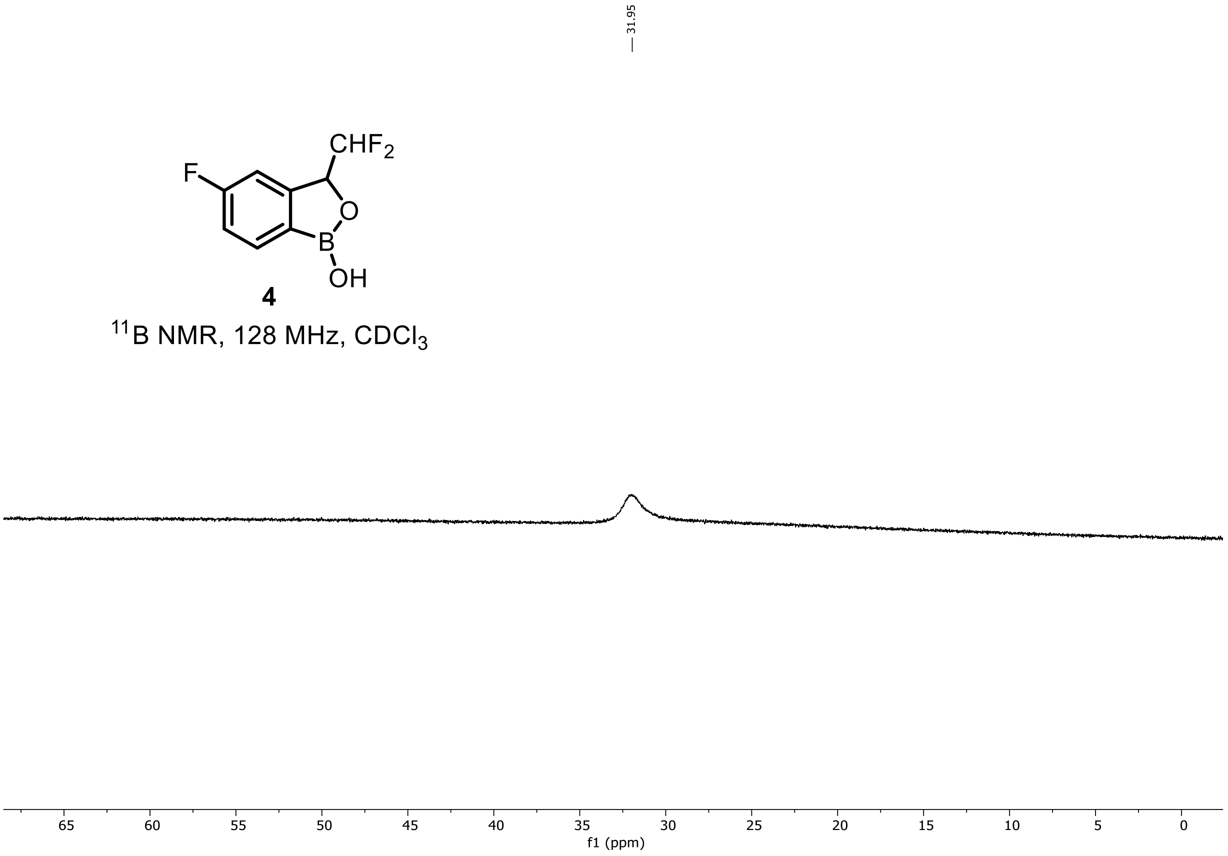

^{19}F NMR (376 MHz, $CDCl_3$) of **4**

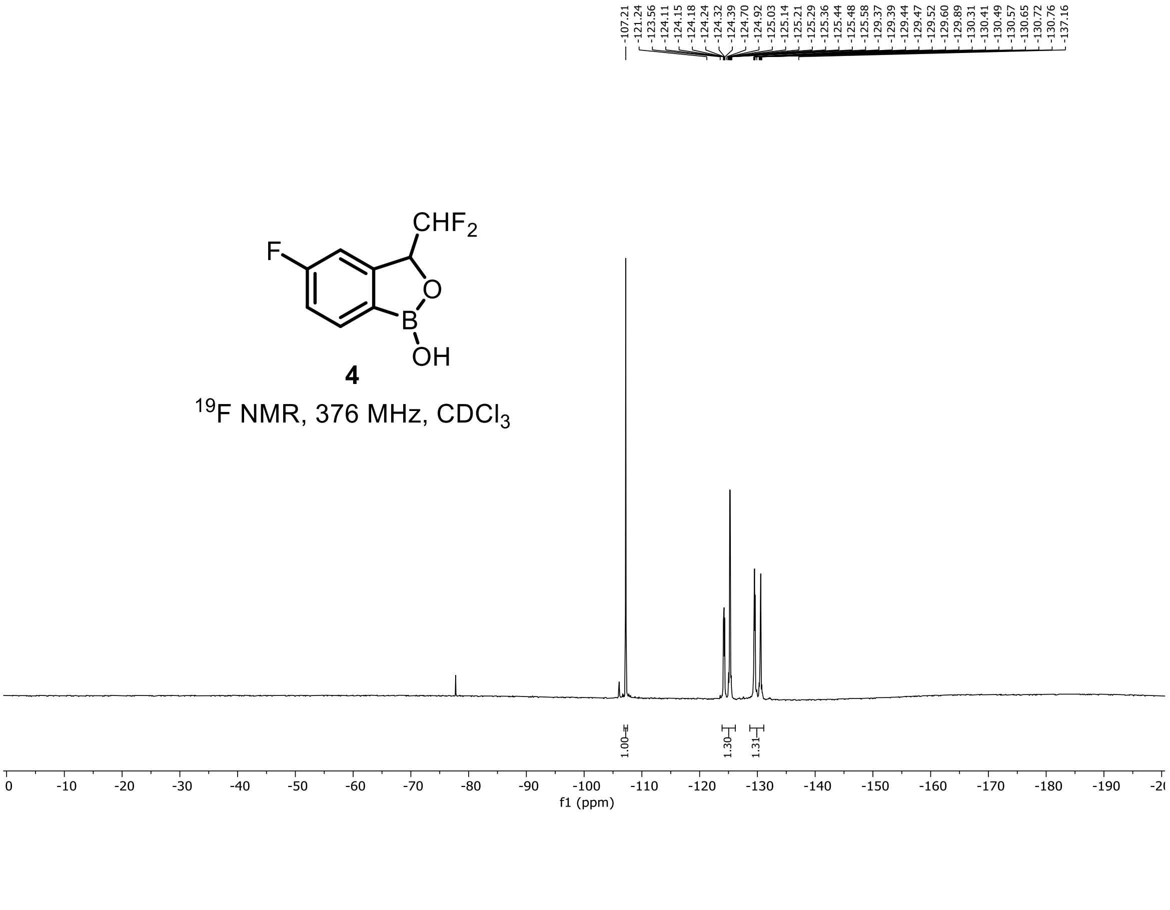

^{1}H NMR (400 MHz, $CDCl_3$) of **5** (see procedure)

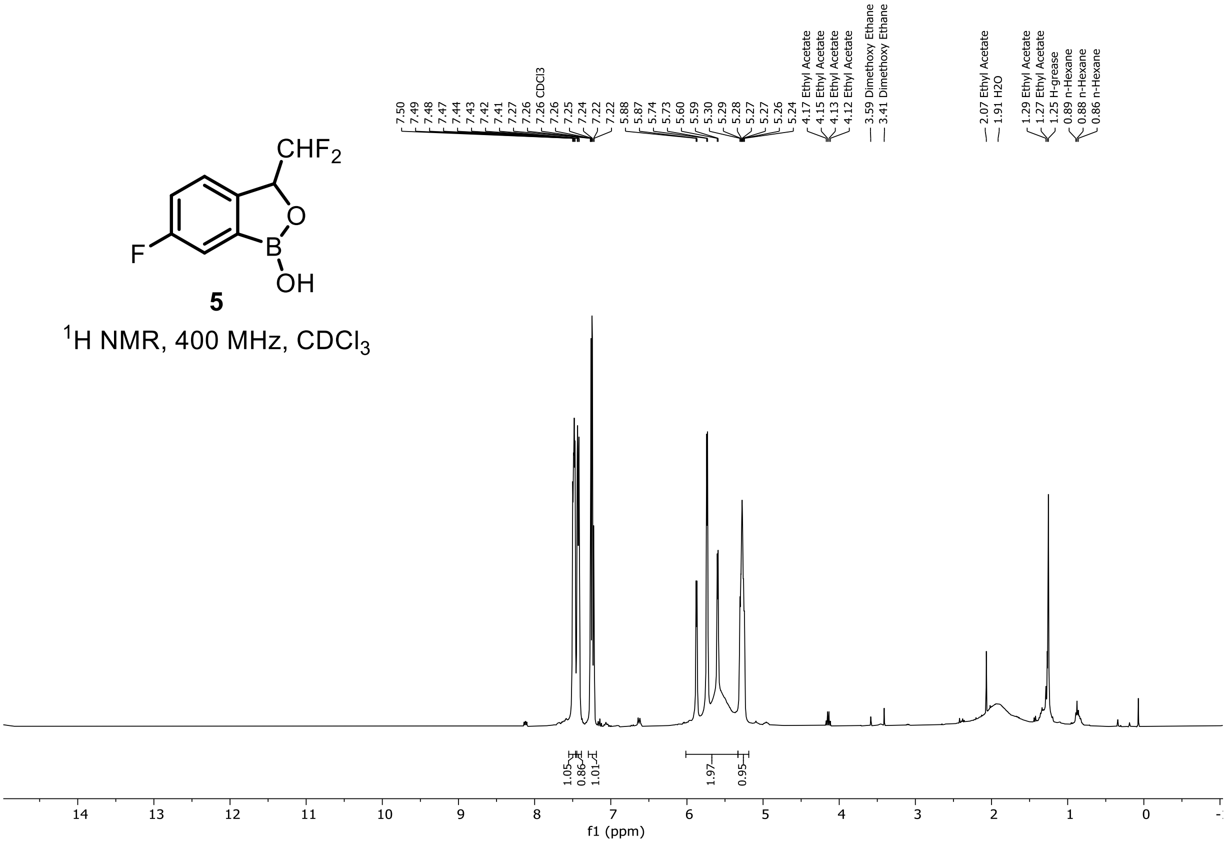

^{13}C NMR (101 MHz, $CDCl_3$) of **5**

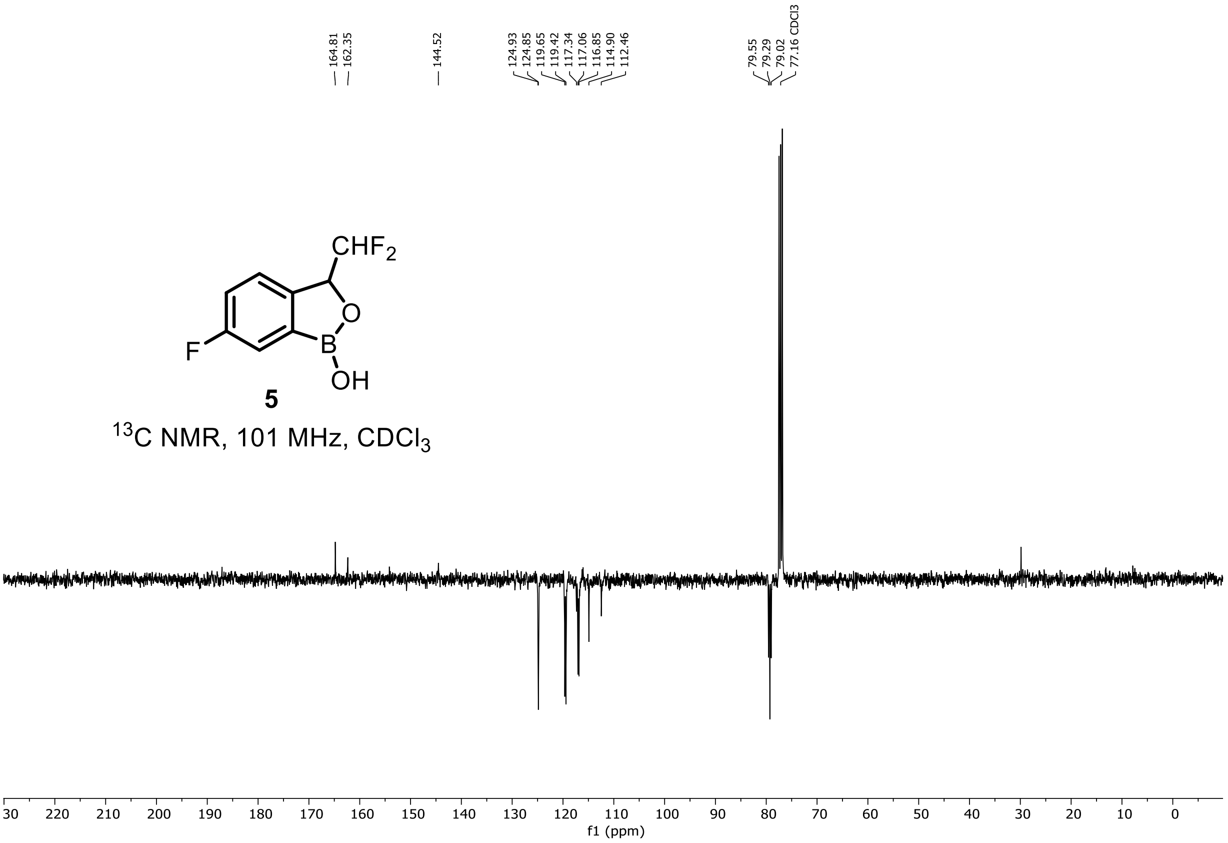

^{11}B NMR (128 MHz, $CDCl_3$) of **5**

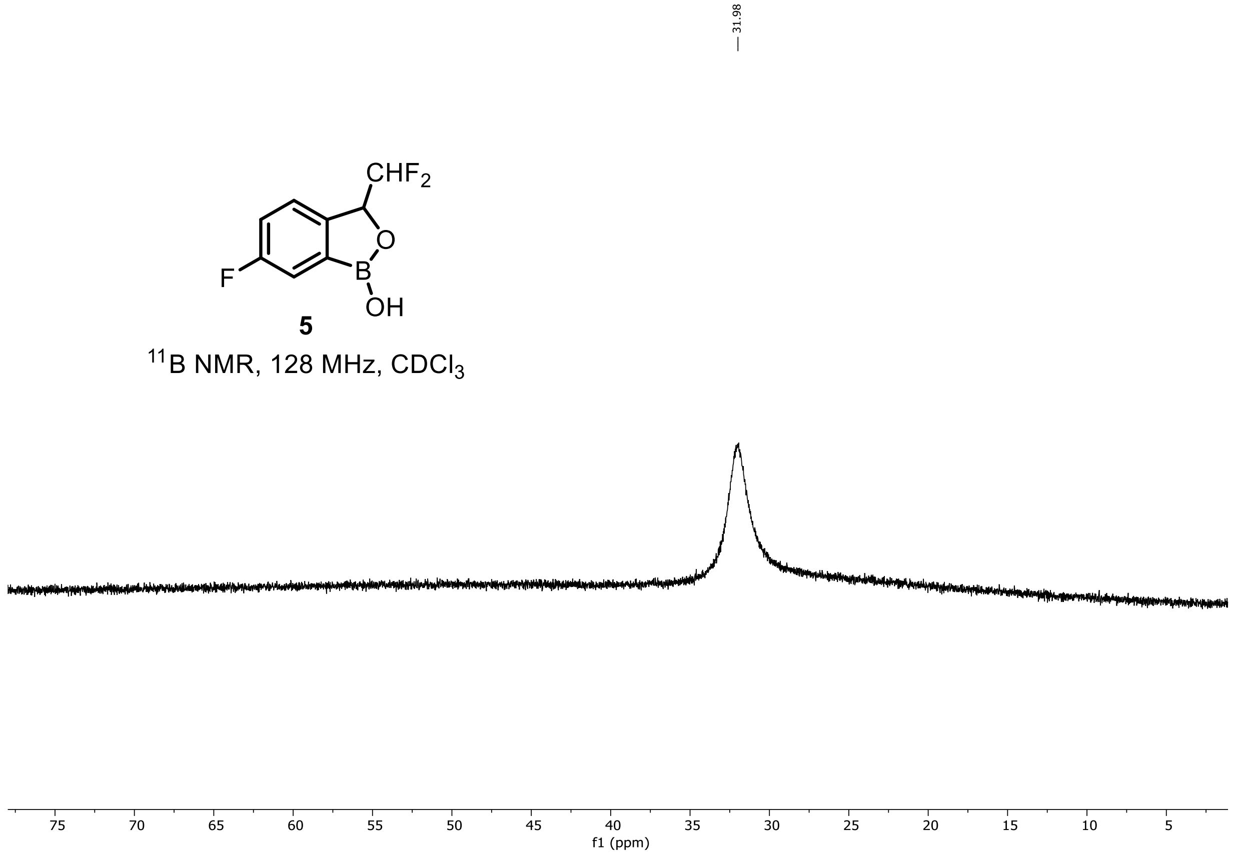

^{19}F NMR (376 MHz, $CDCl_3$) of **5**

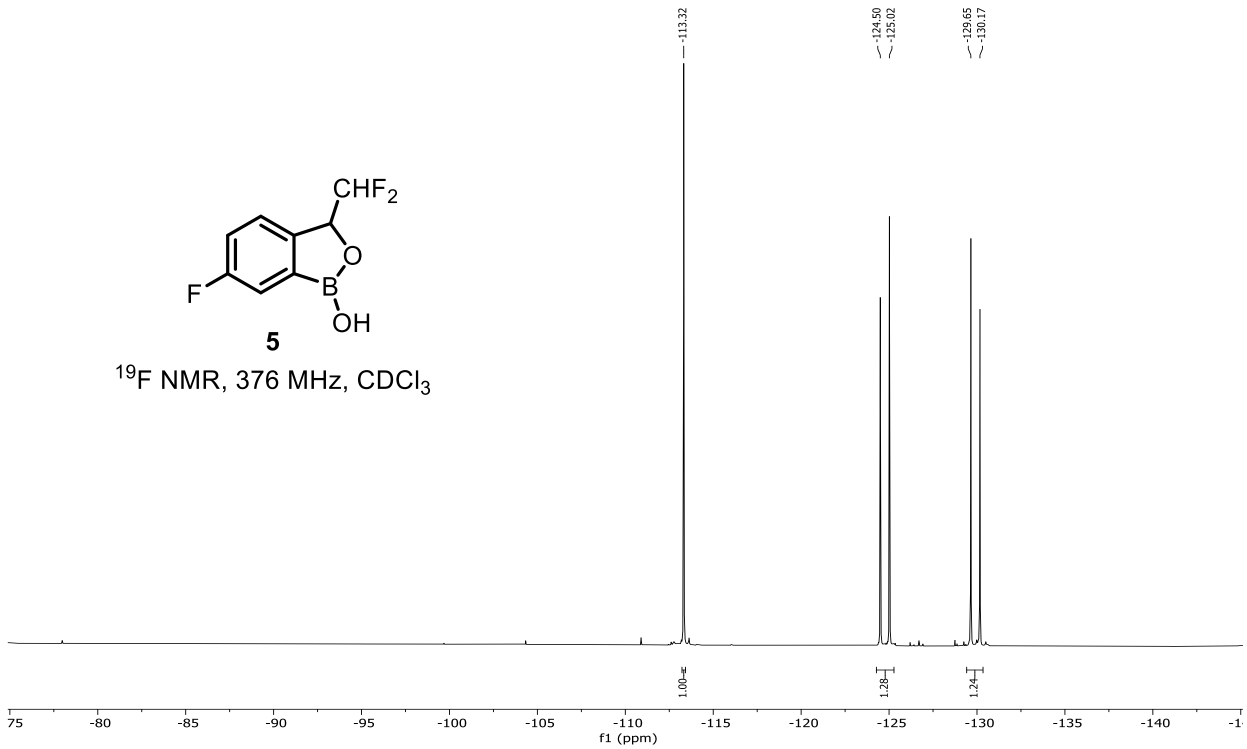

^{1}H NMR (400 MHz, $CDCl_3$) of **6** (see procedure)

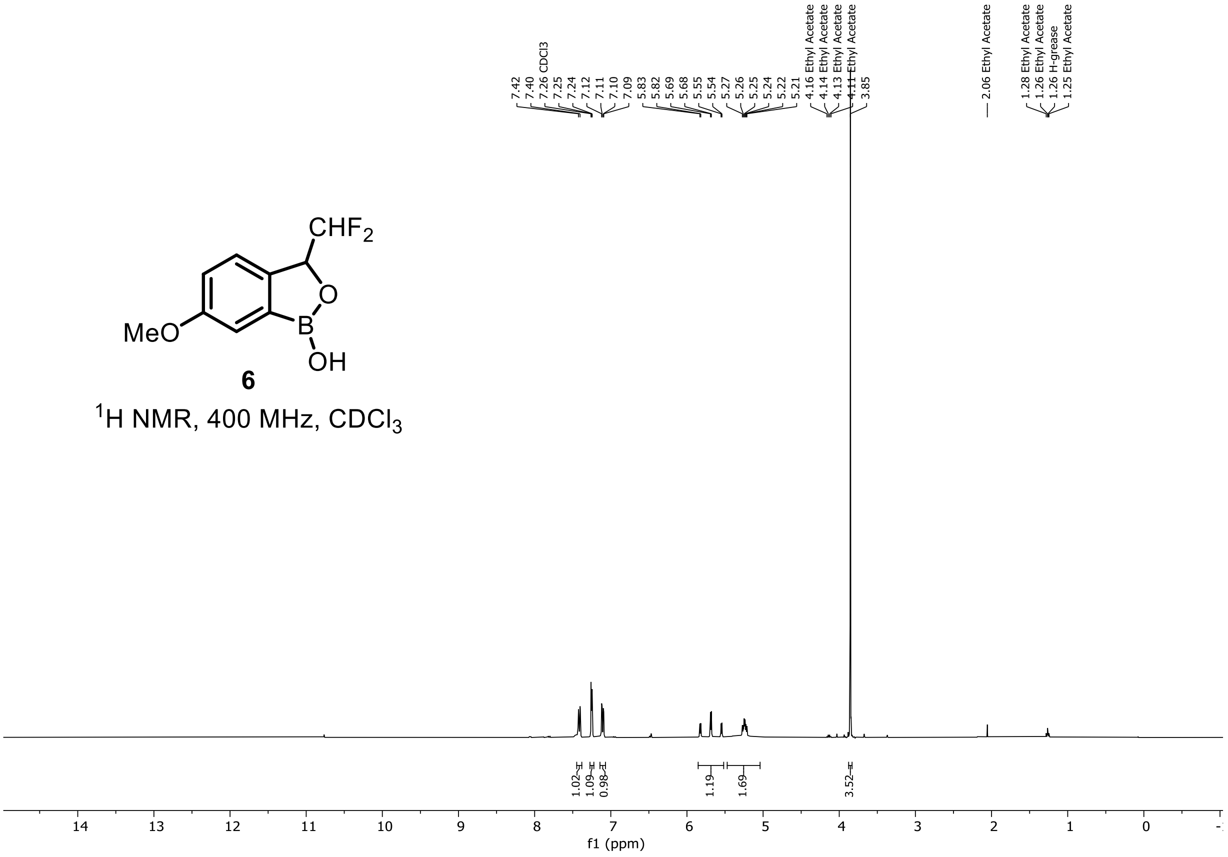

^{13}C NMR (101 MHz, $CDCl_3$) of **6**

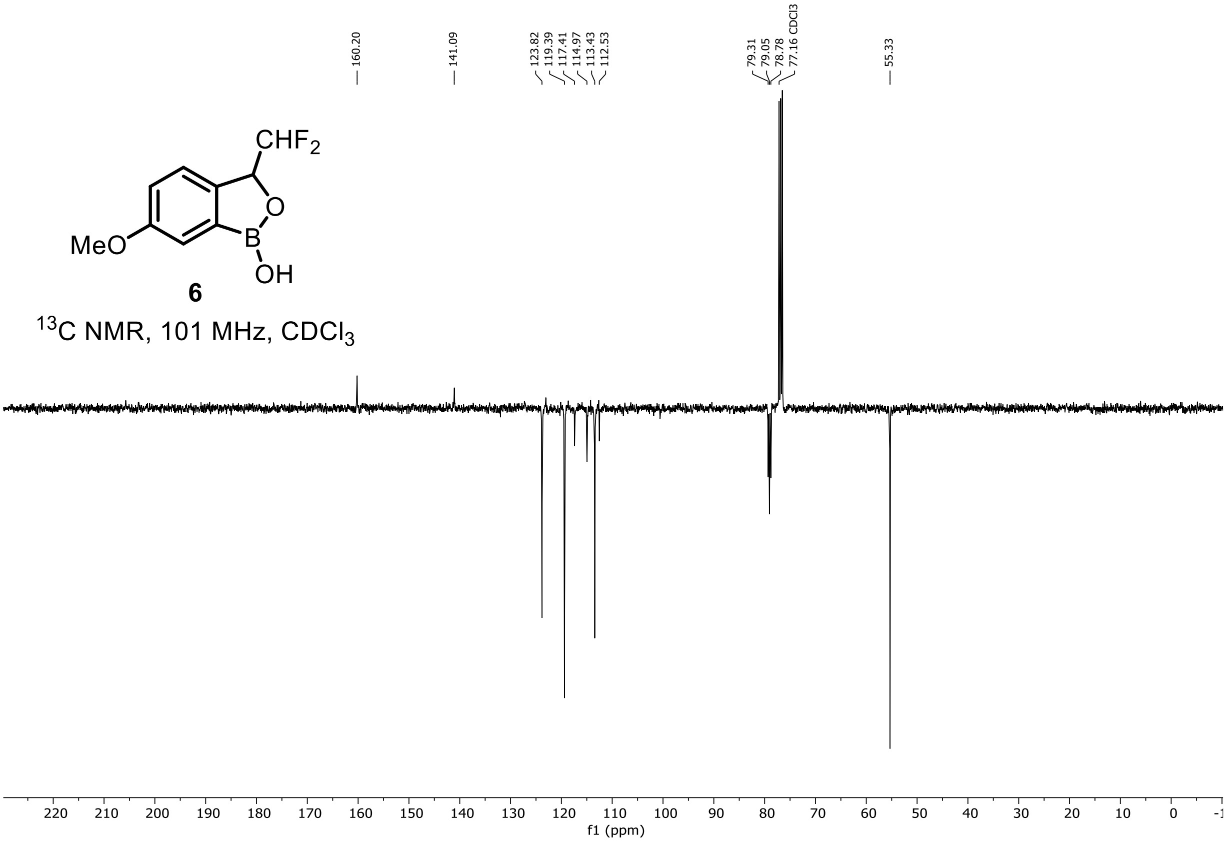

^{11}B NMR (128 MHz, $CDCl_3$) of **6**

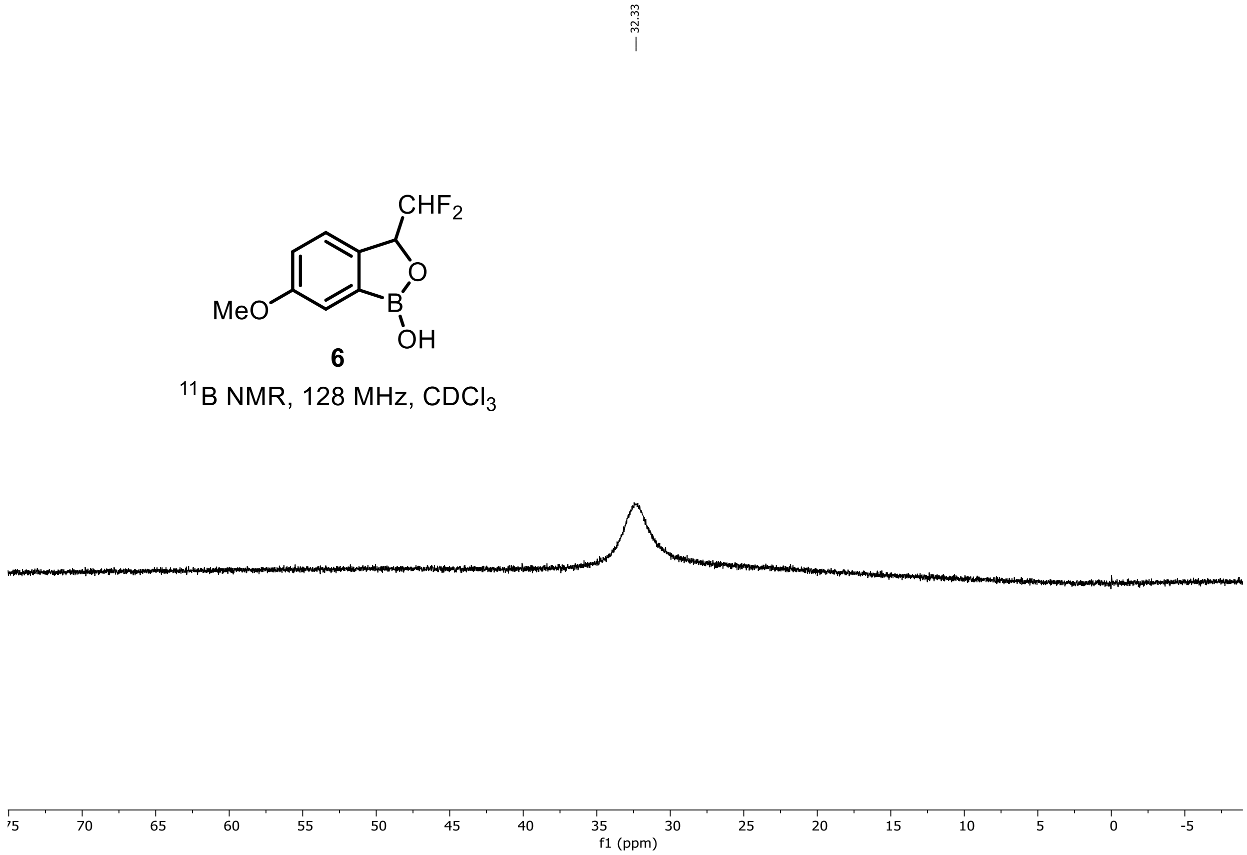

^{19}F NMR (376 MHz, $CDCl_3$) of **6**

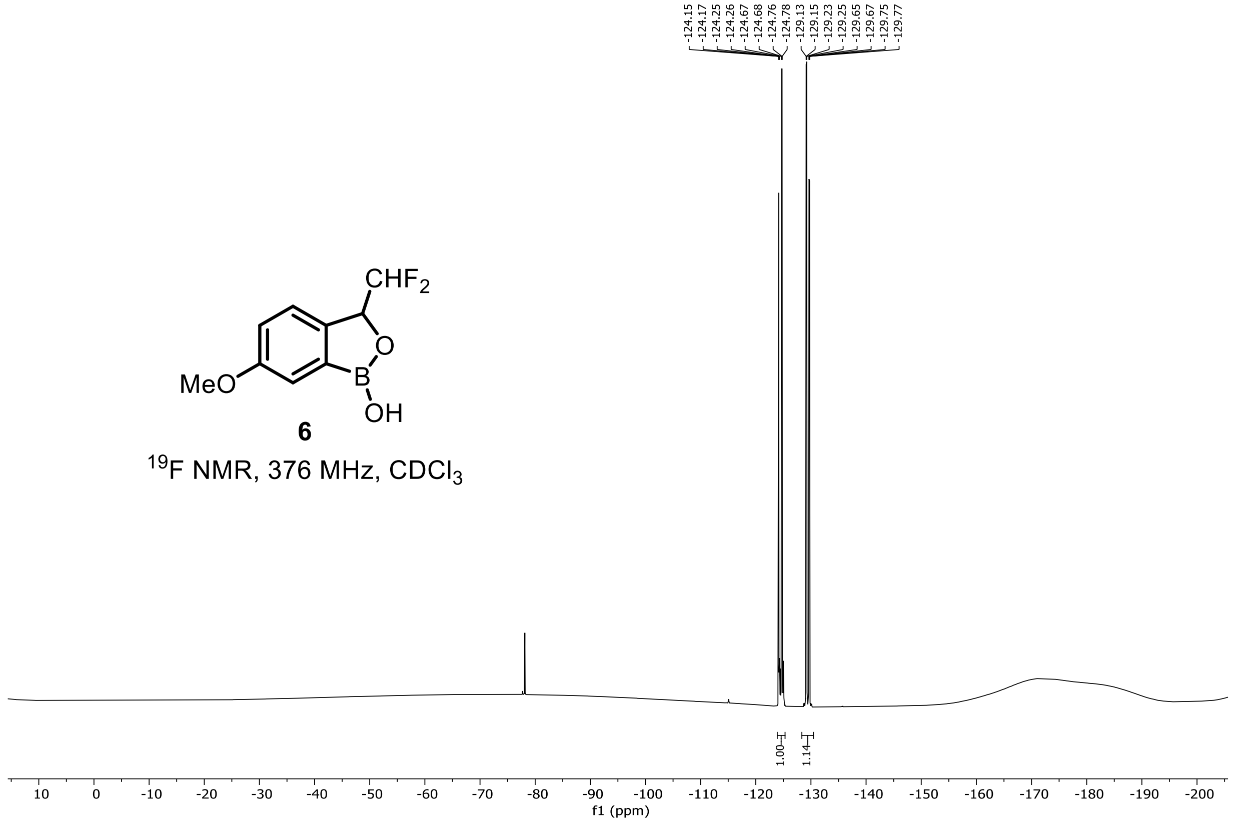

^1H NMR (400 MHz, $CDCl_3$) of **7** (see procedure)

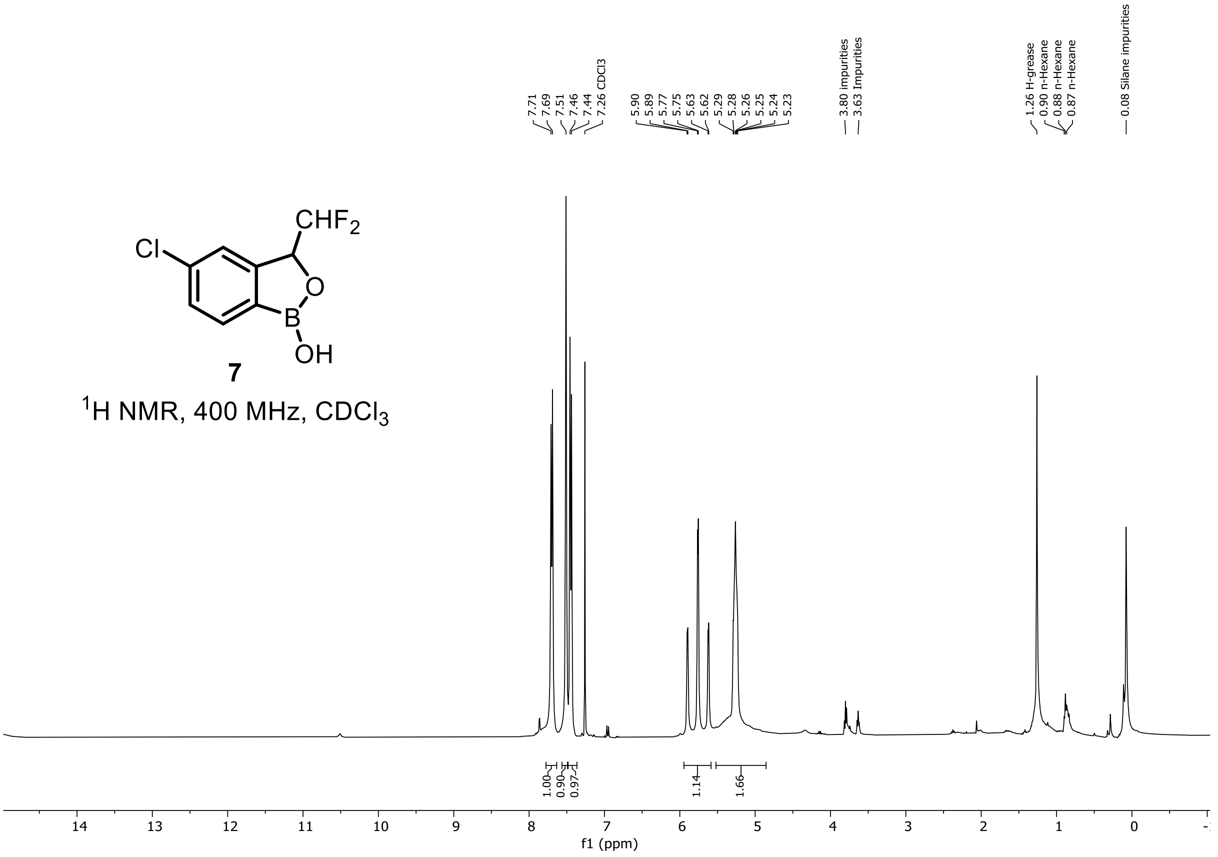

^{13}C NMR (101 MHz, $CDCl_3$) of **7**

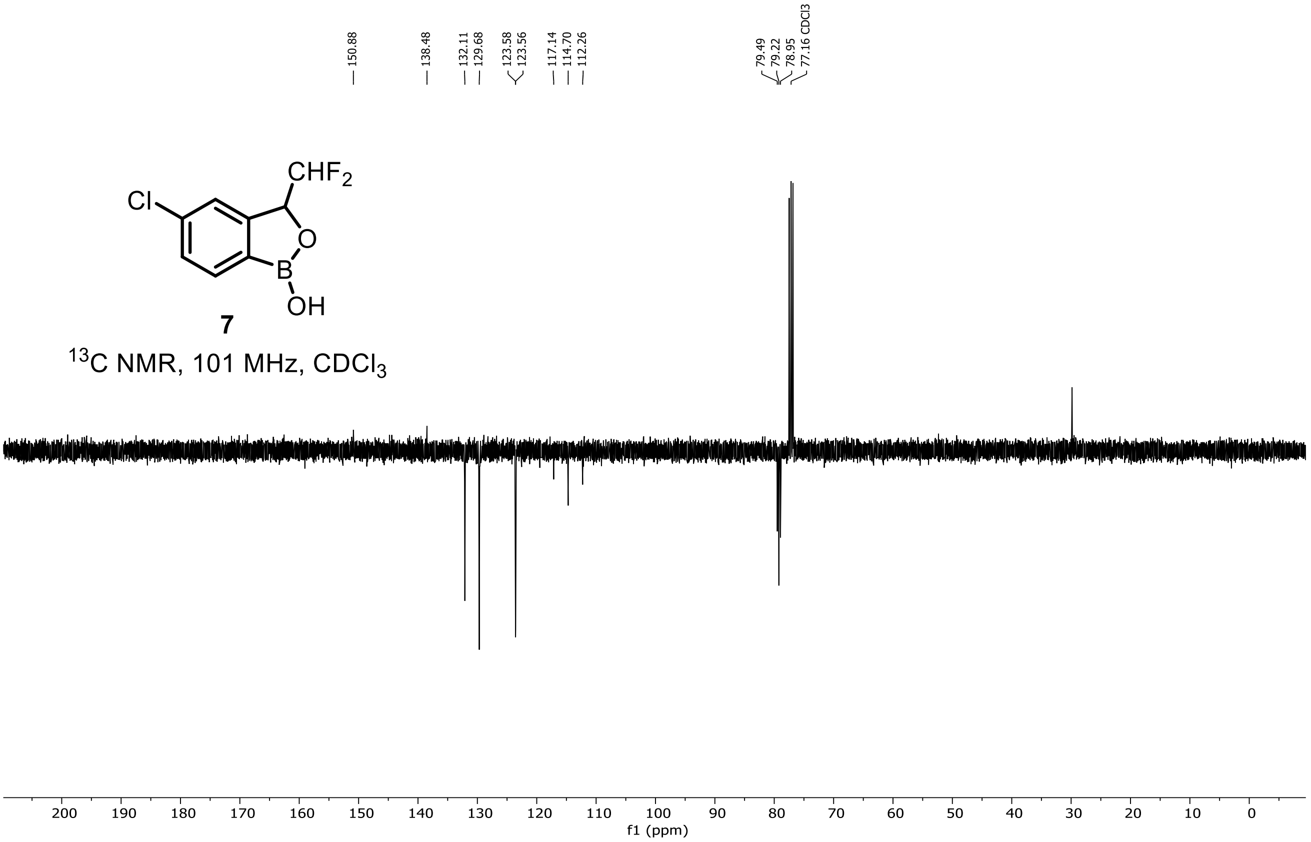

^{11}B NMR (128 MHz, $CDCl_3$) of **7**

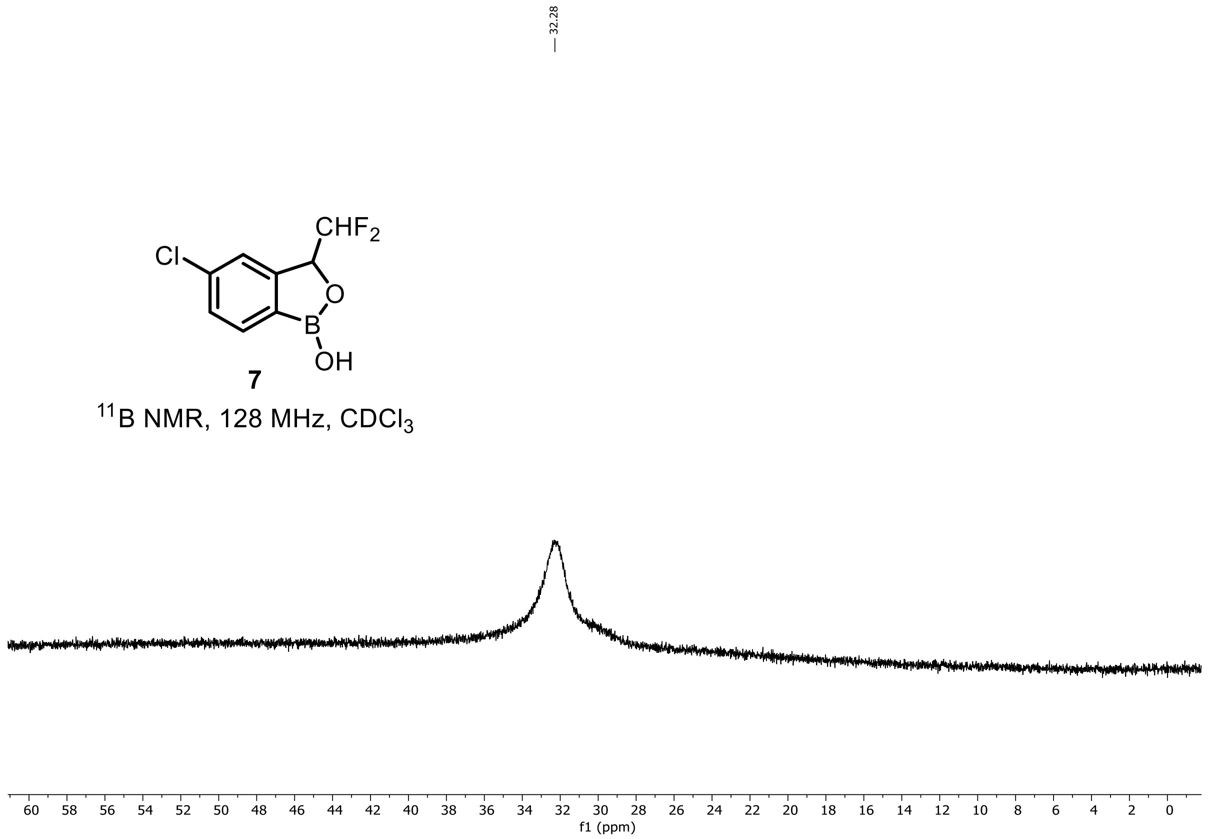

^{19}F NMR (376 MHz, $CDCl_3$) of **7**

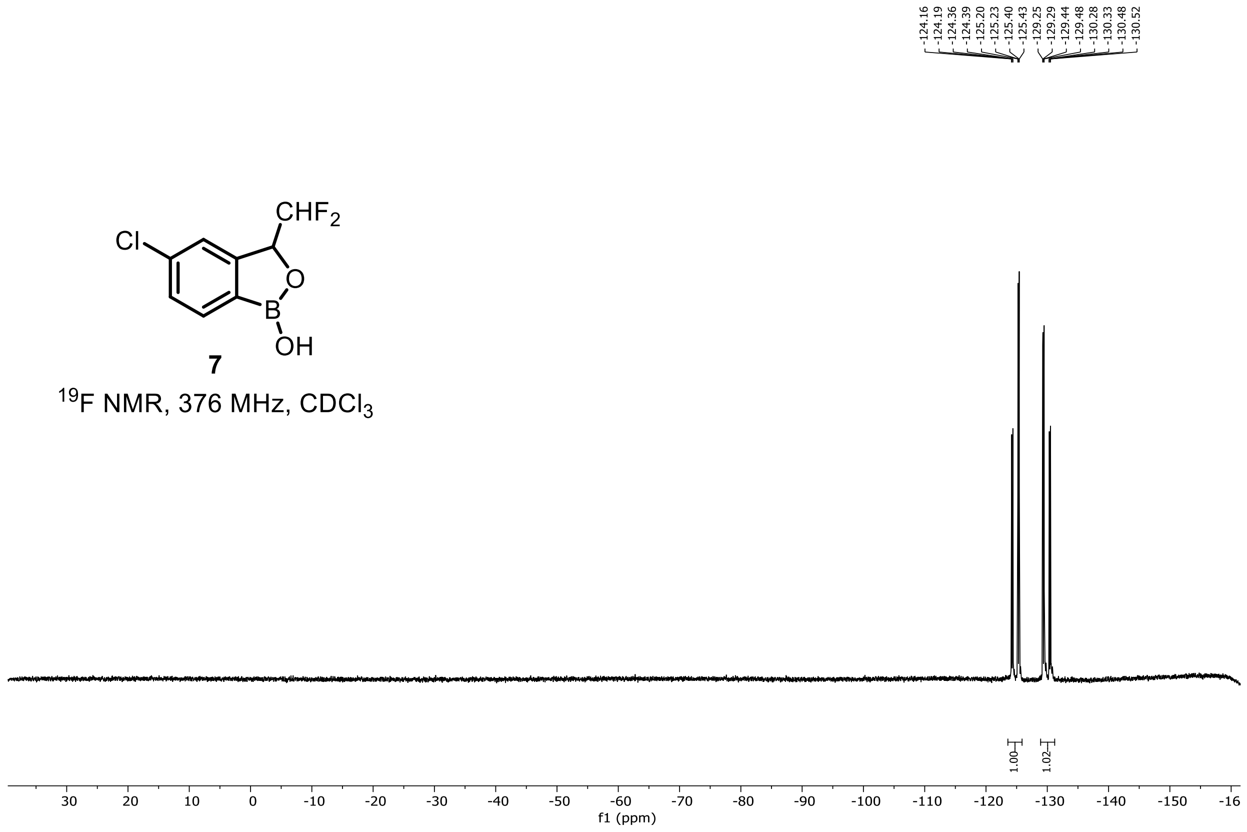

^{1}H NMR (400 MHz, $CDCl_3$) of **8** (see procedure)

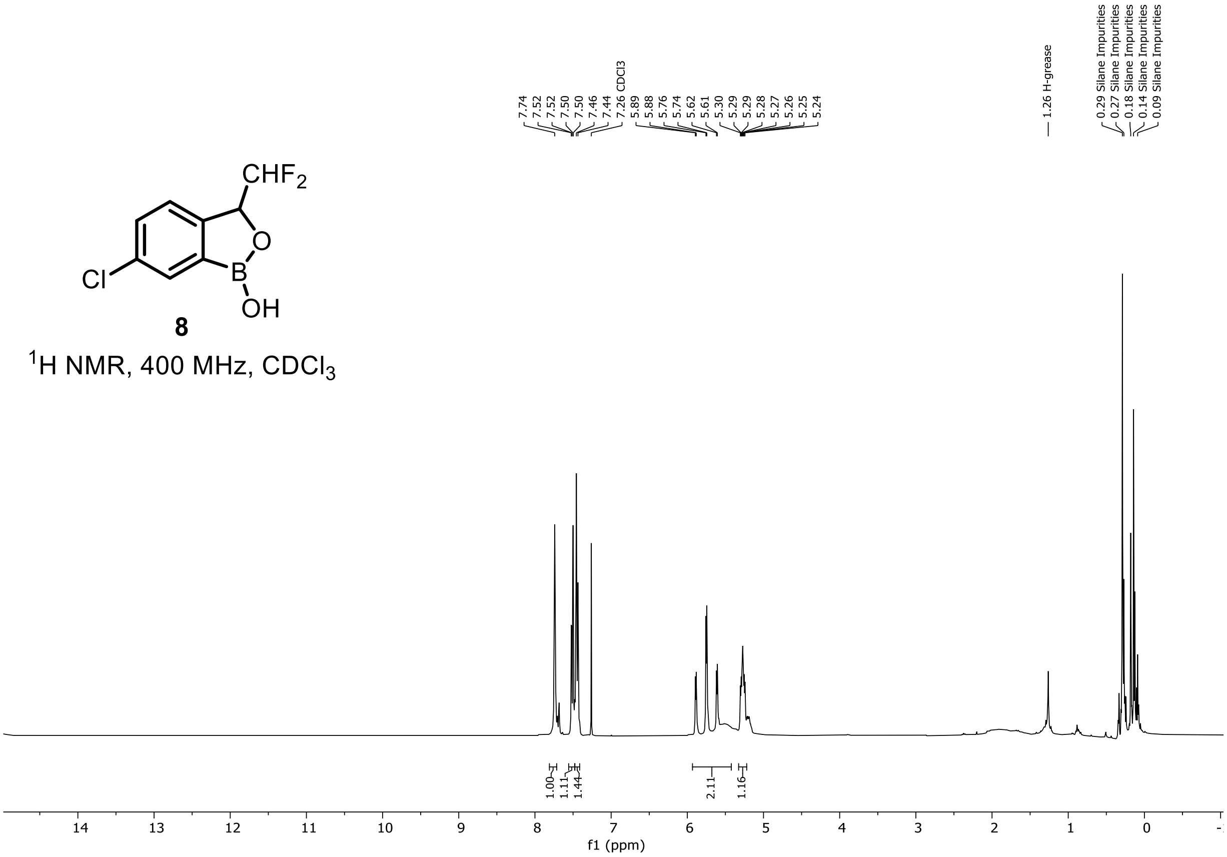

^{13}C NMR (101 MHz, $CDCl_3$) of **8**

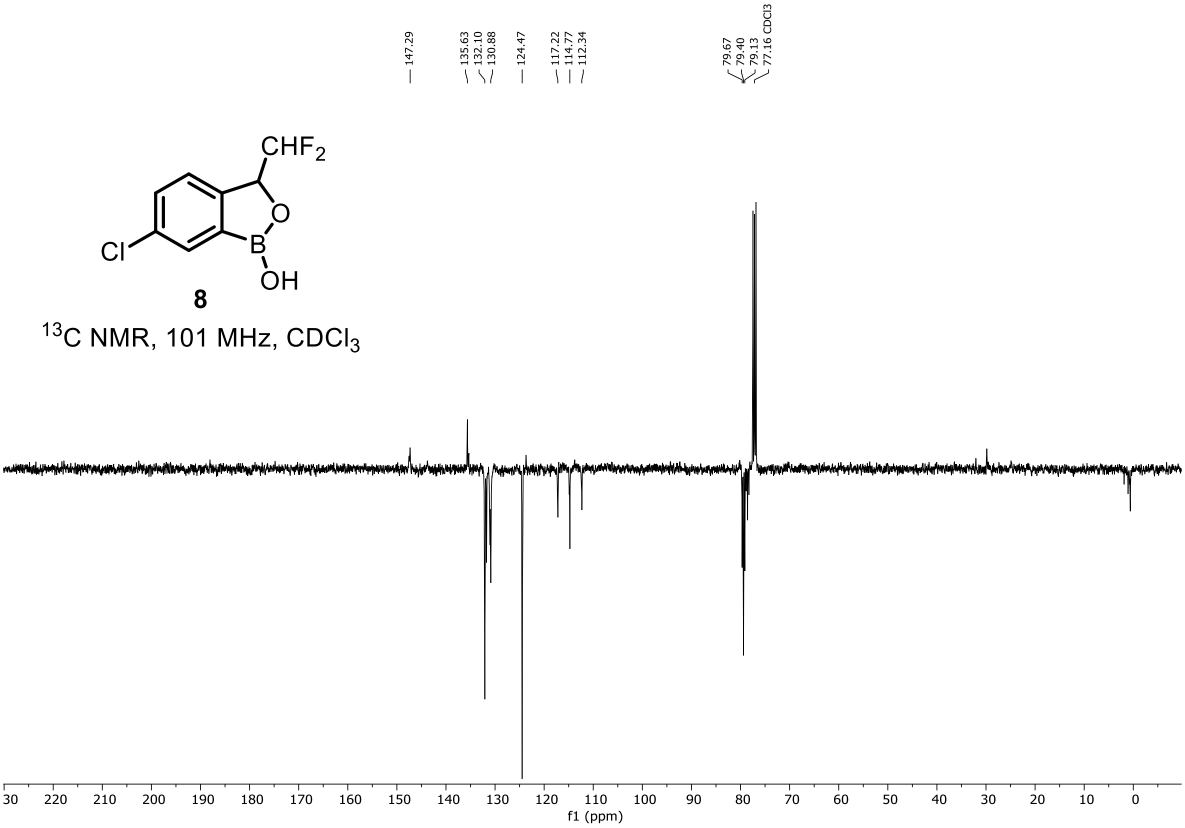

^{11}B NMR (128 MHz, $CDCl_3$) of **8**

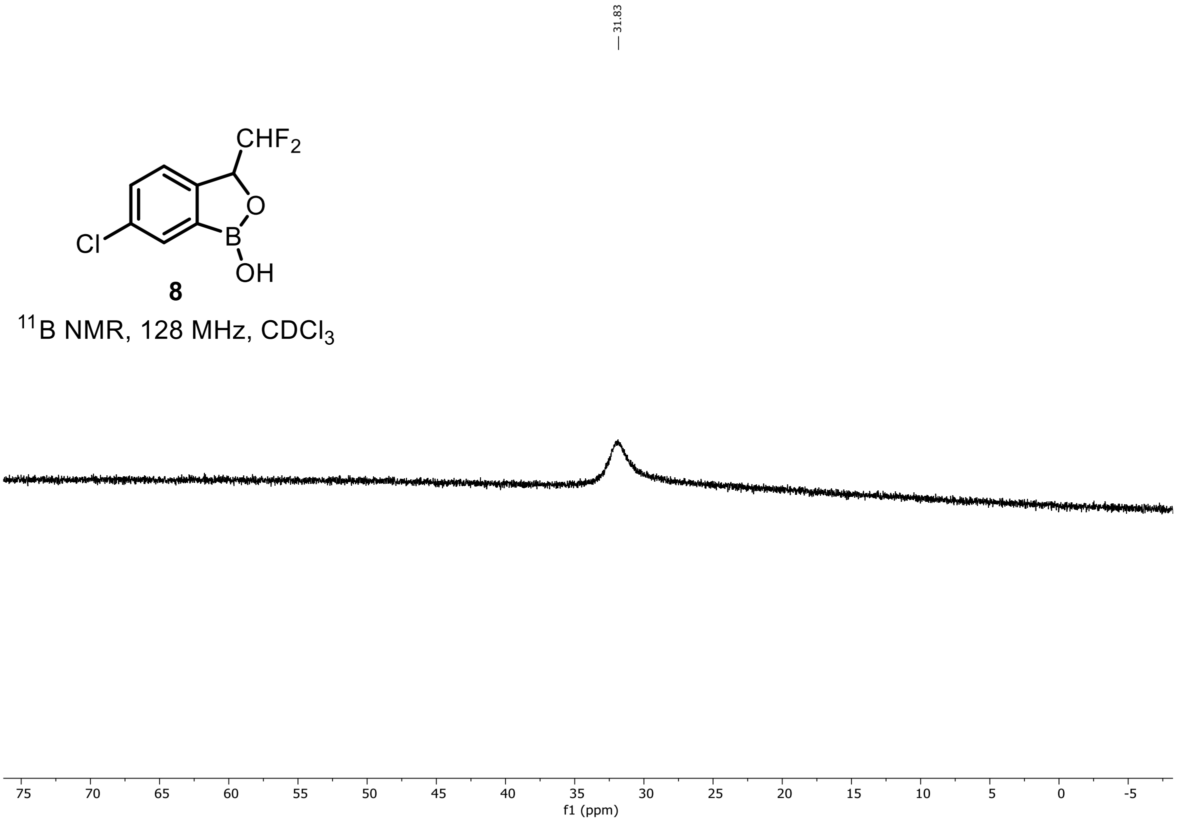

^{19}F NMR (376 MHz, $CDCl_3$) of **8**

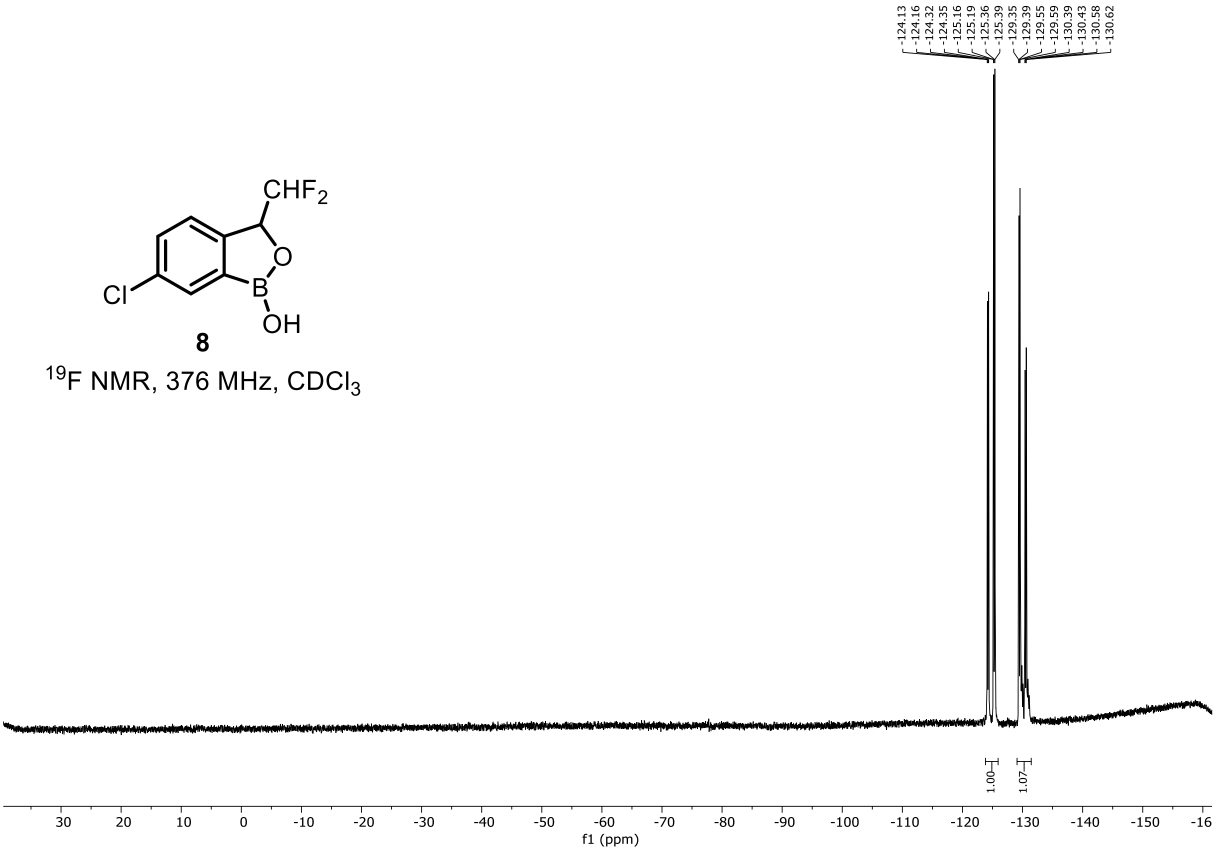

^1H NMR (400 MHz, $CDCl_3$) of **9** (see procedure)

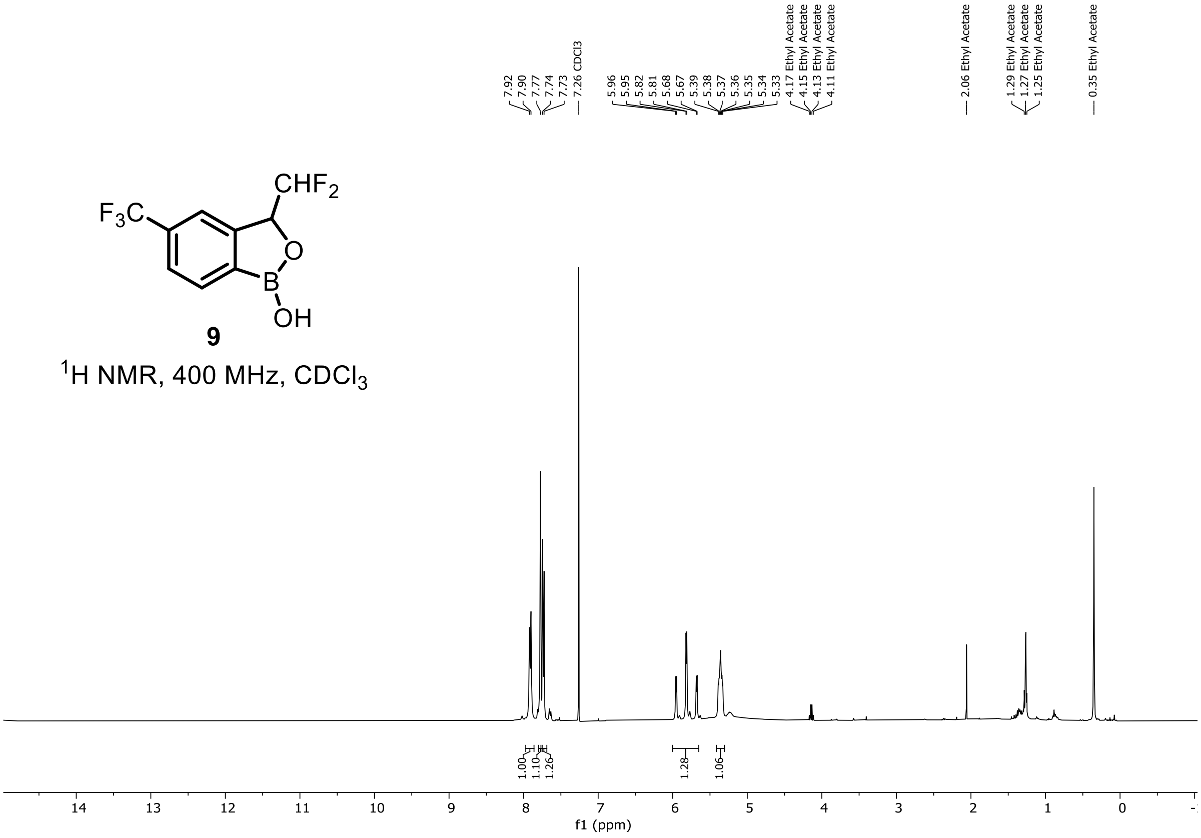

^{13}C NMR (101 MHz, $CDCl_3$) of **9**

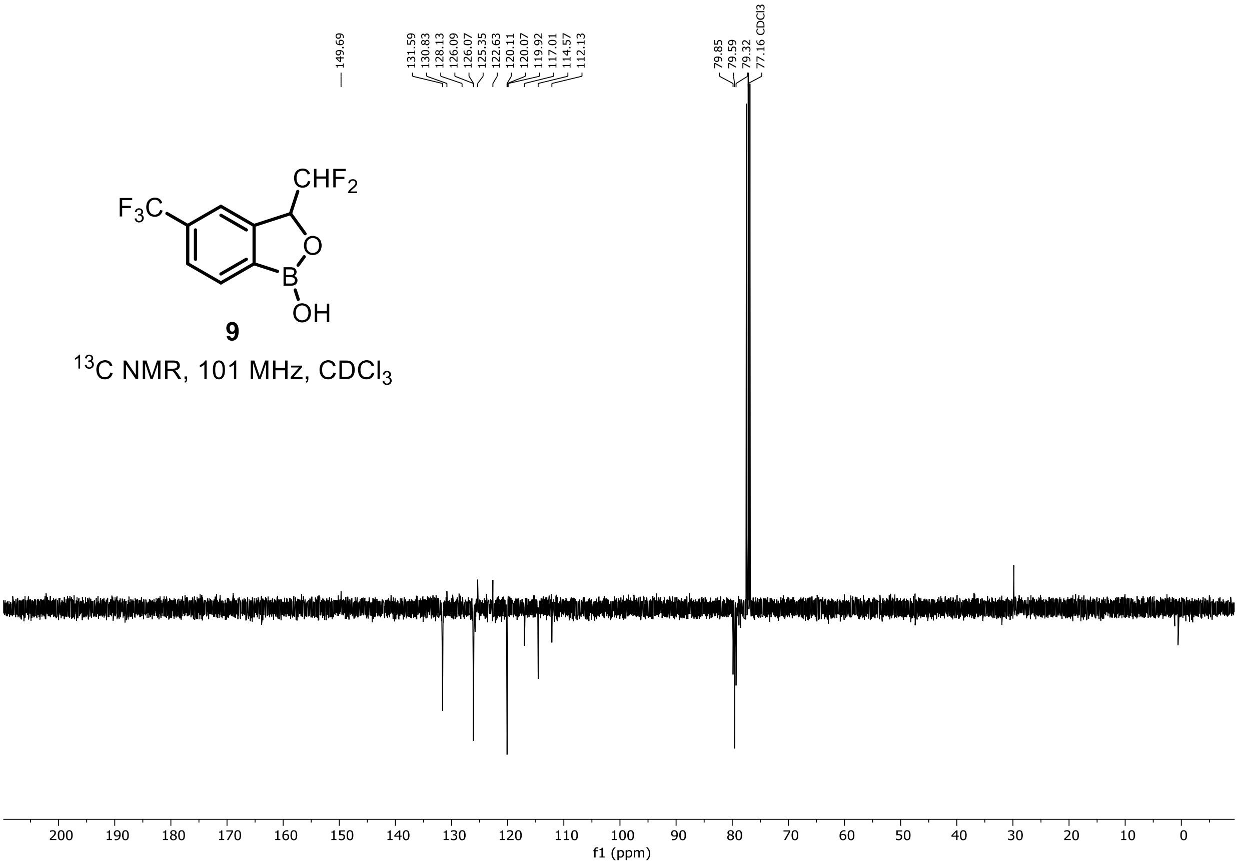

^{11}B NMR (128 MHz, $CDCl_3$) of **9**

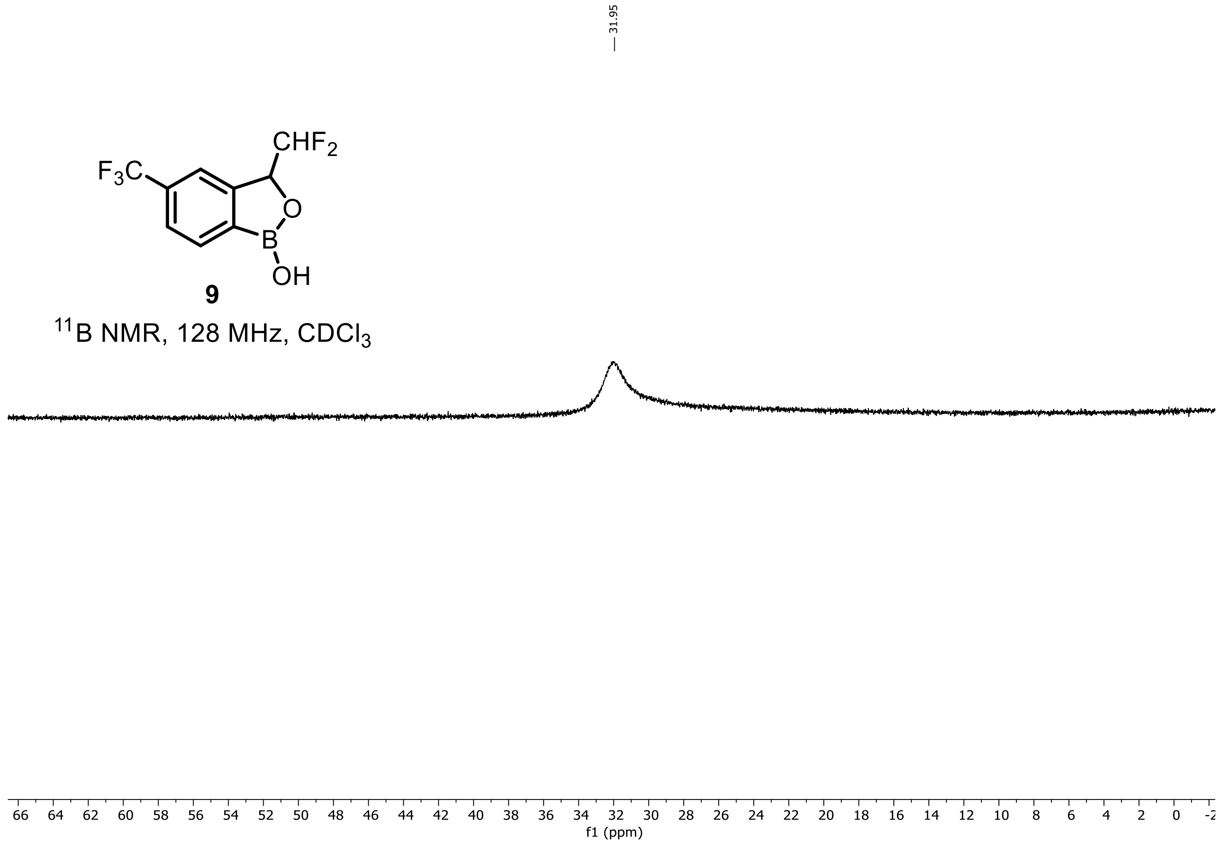

^{19}F NMR (376 MHz, $CDCl_3$) of **9**

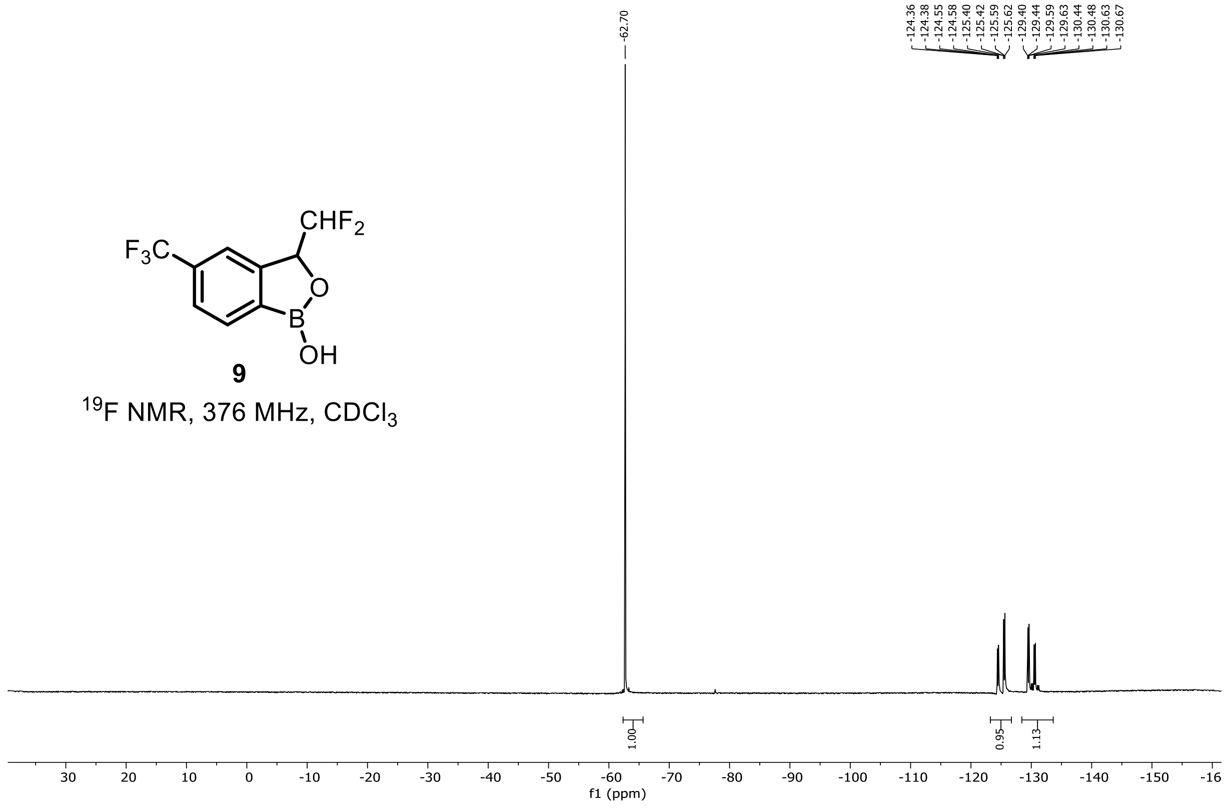

^{1}H NMR (400 MHz, $CDCl_3$) of **10** (see procedure)

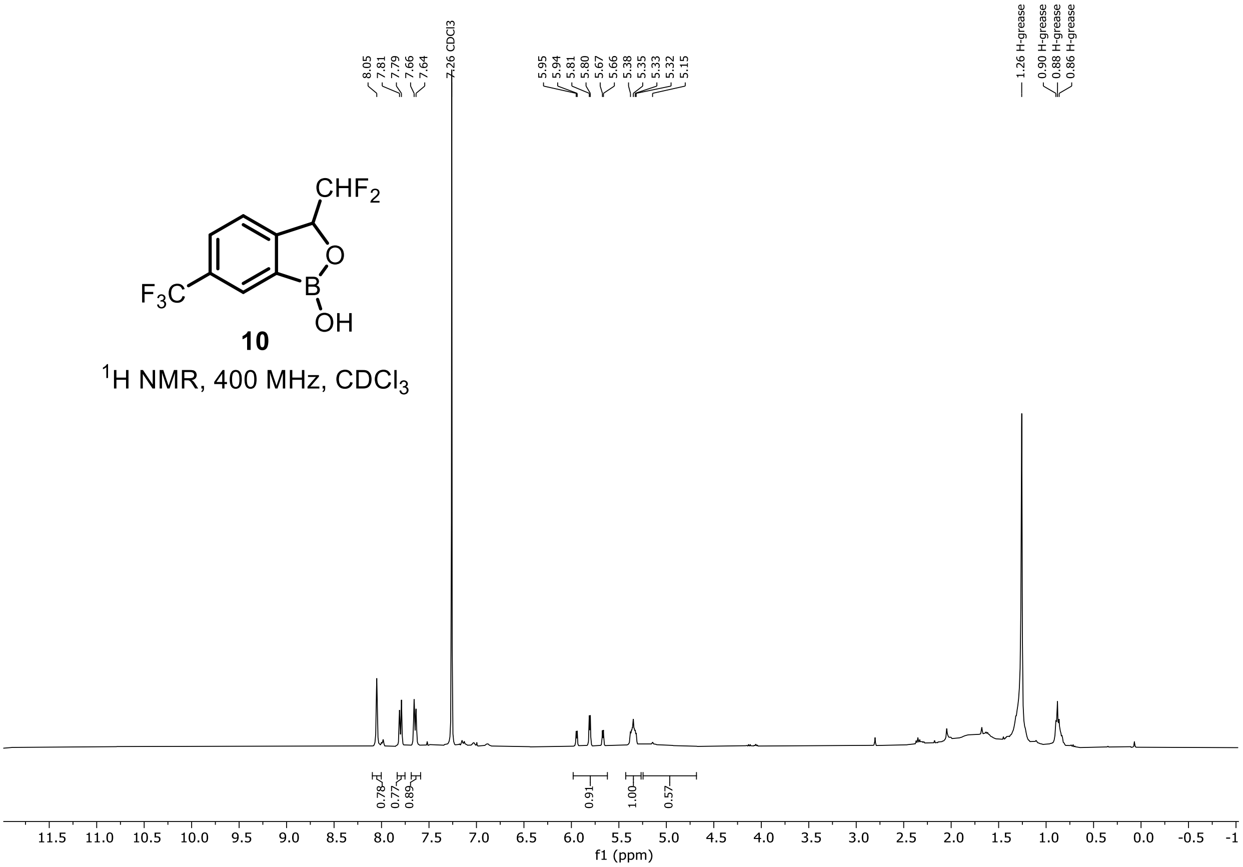

^{13}C NMR (101 MHz, $CDCl_3$) of **10**

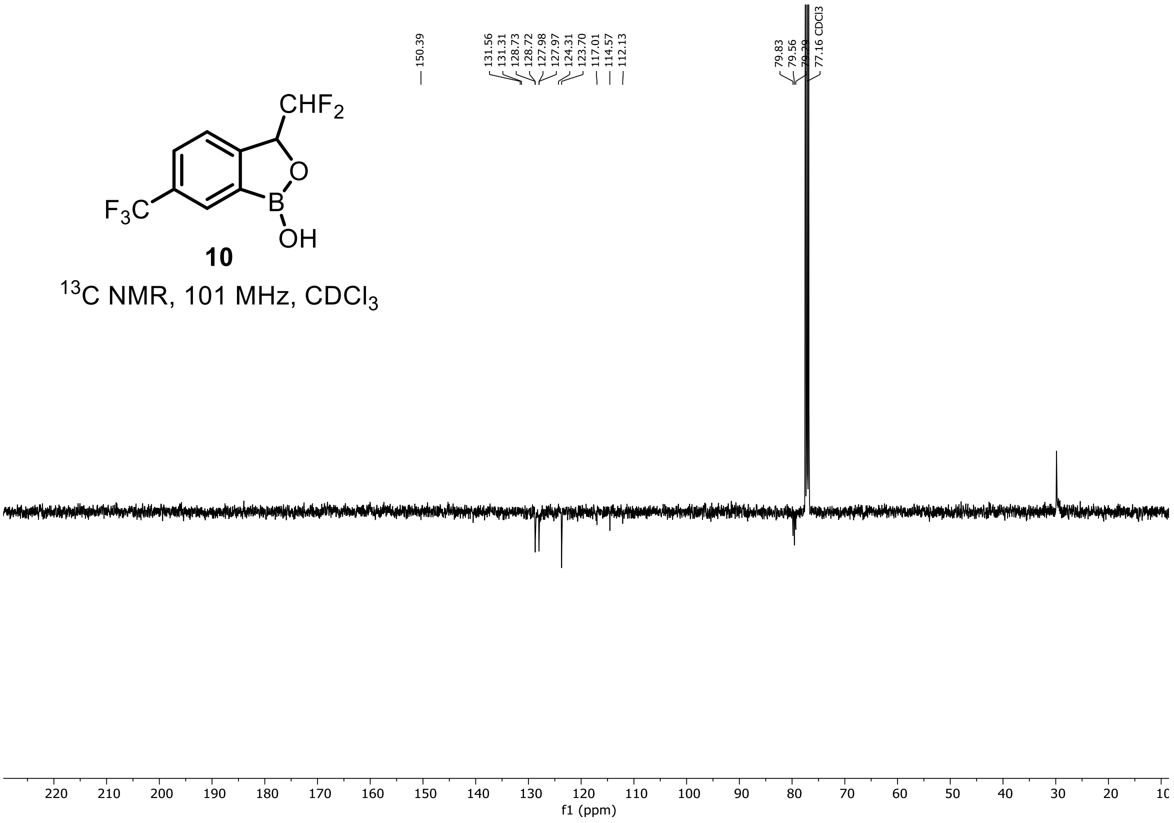

^{11}B NMR (128 MHz, CDCl3) of **10**

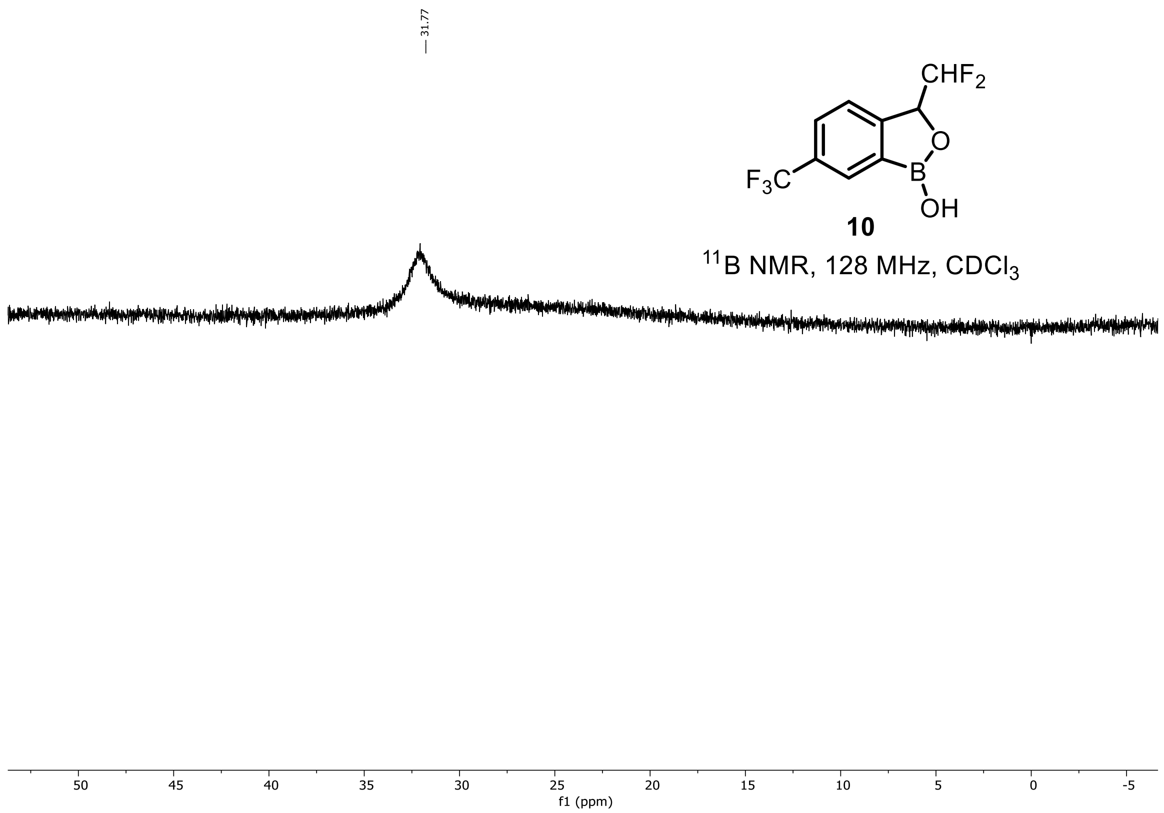

^{19}F NMR (376 MHz, $CDCl_3$) of **10**

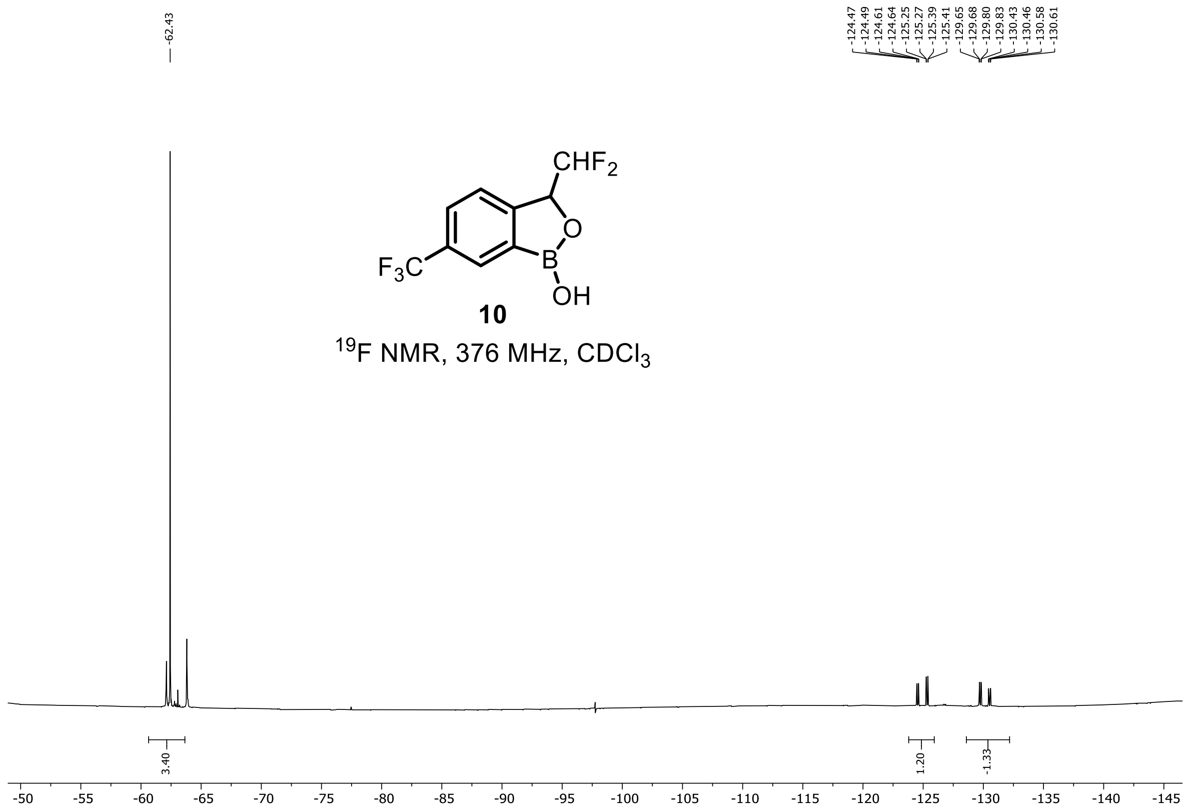

^{1}H NMR (400 MHz, $CDCl_3$) of **I-C** (see procedure)

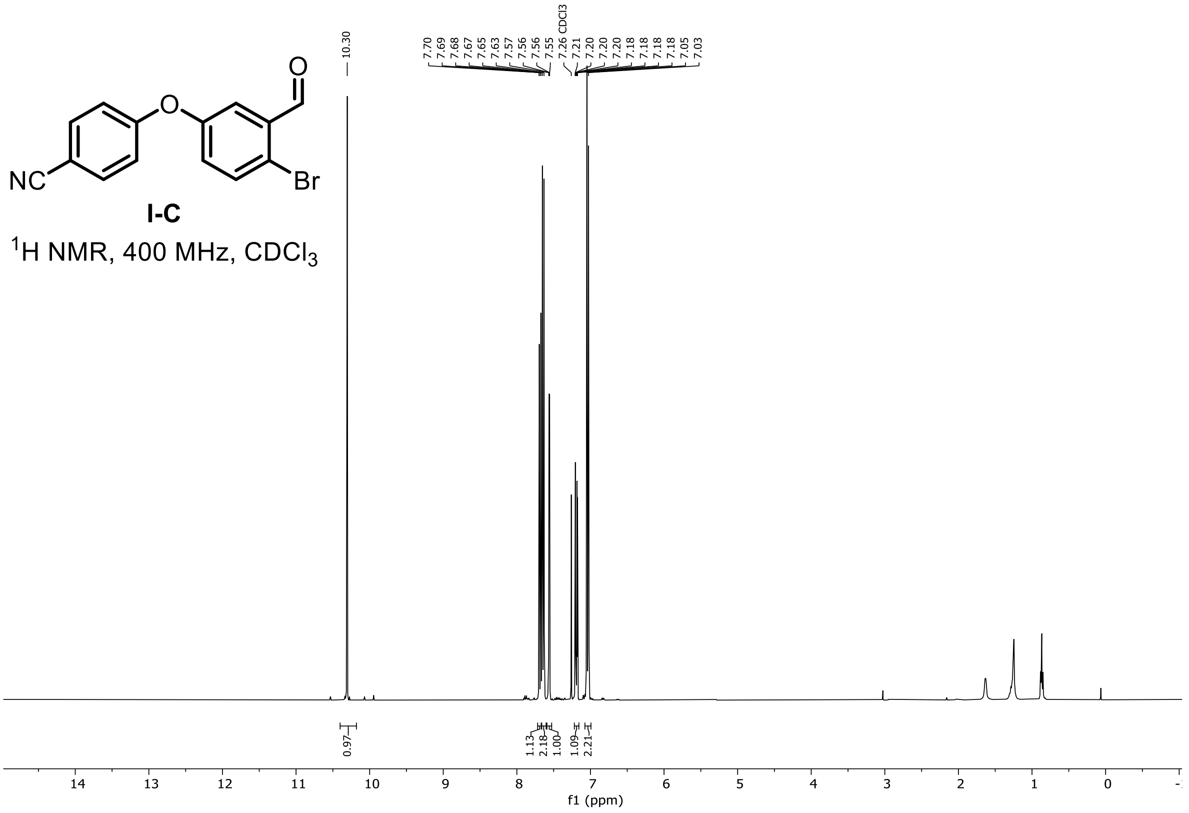

^{1}H NMR (400 MHz, $CDCl_3$) of **II-C** (see procedure)

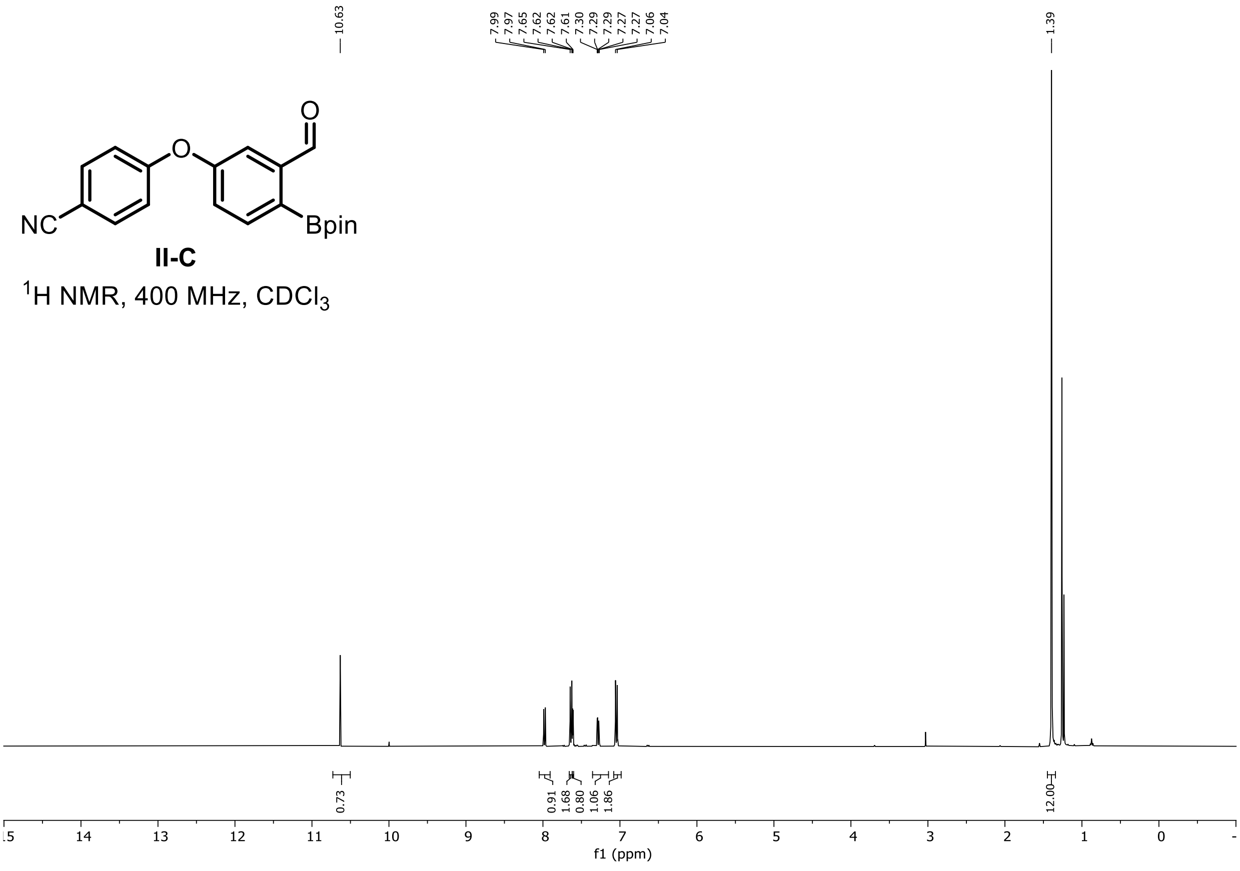

^{11}B NMR (128 MHz, $CDCl_3$) of **II-C**

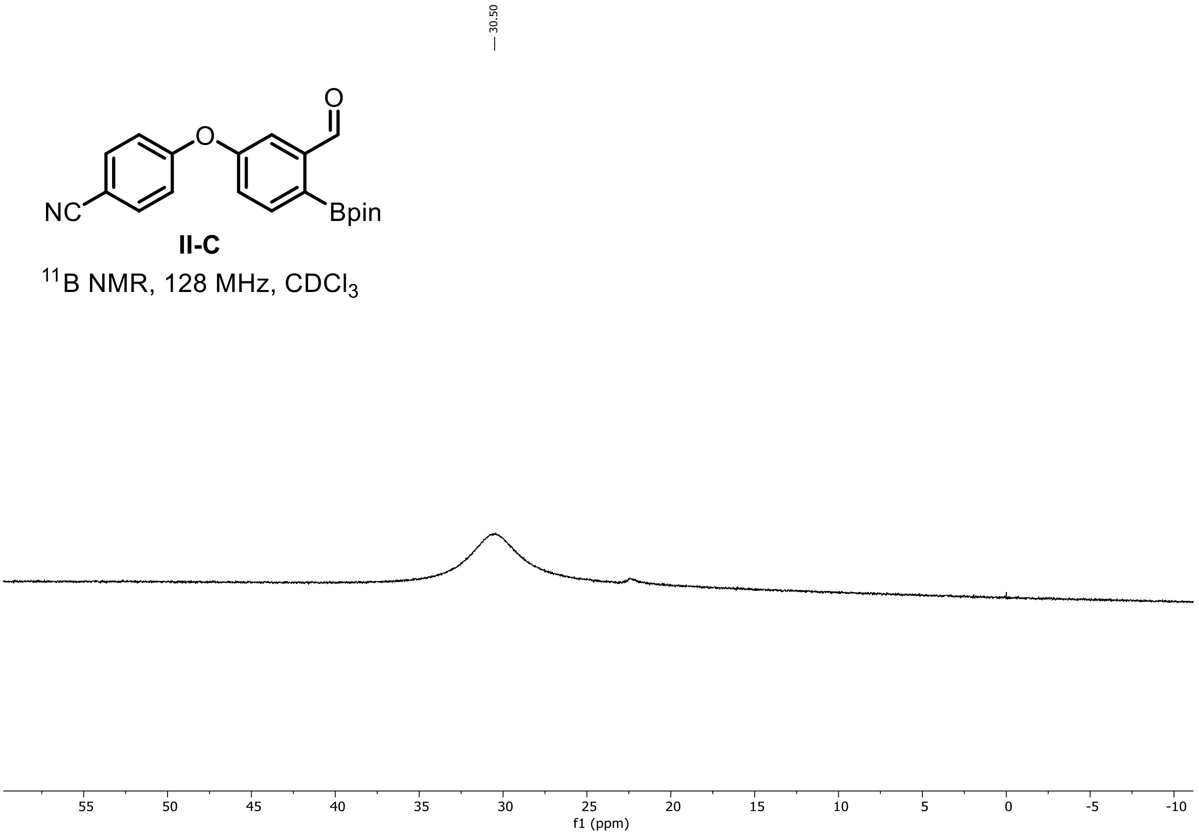

^{1}H NMR (400 MHz, CD_3OD) of **III-C** (see procedure)

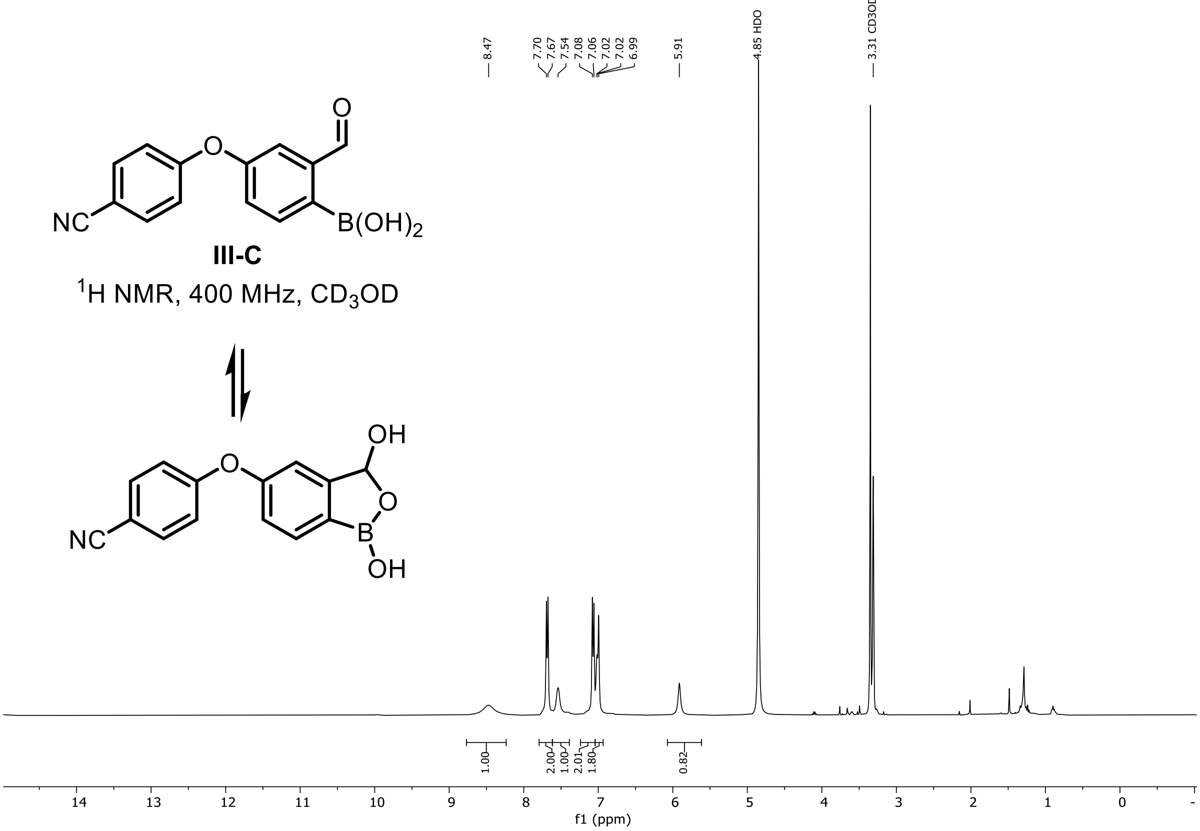

^{11}B NMR (128 MHz, CD_3OD) of **III-C**

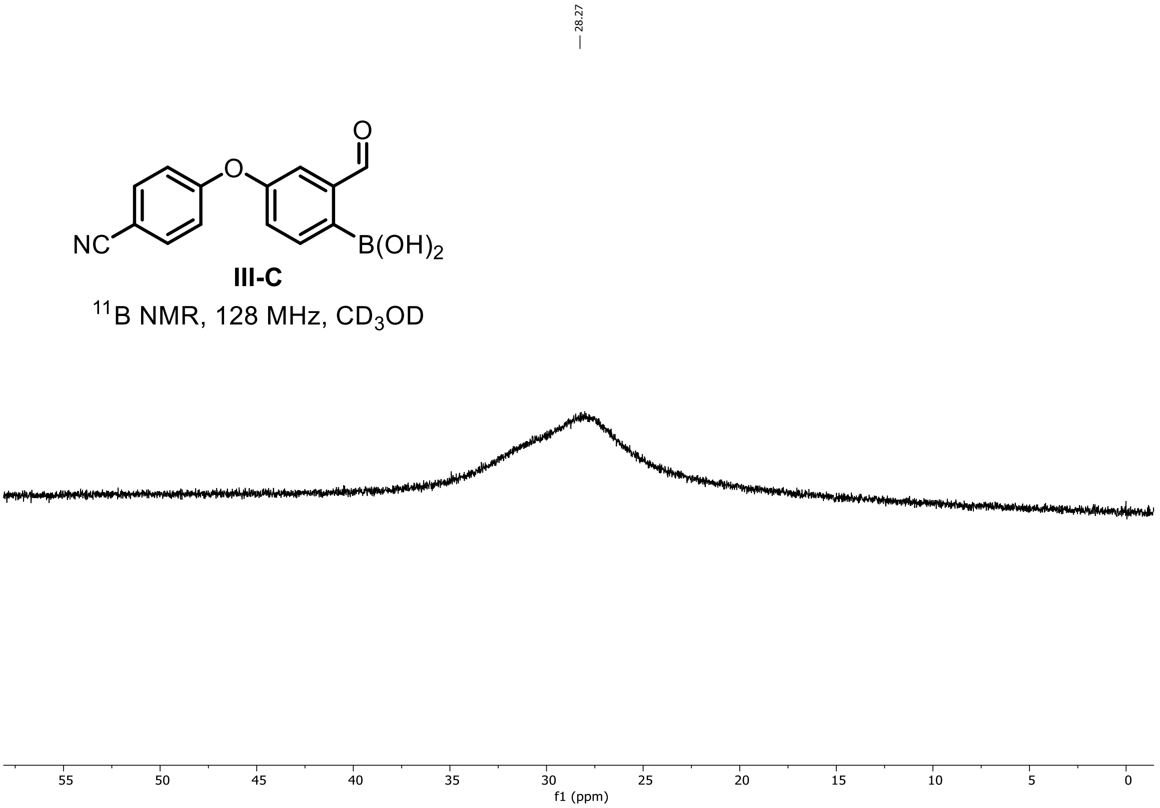

^{1}H NMR (400 MHz, Acetone-d_6) of **XI-B(BDEA)** (see procedure)

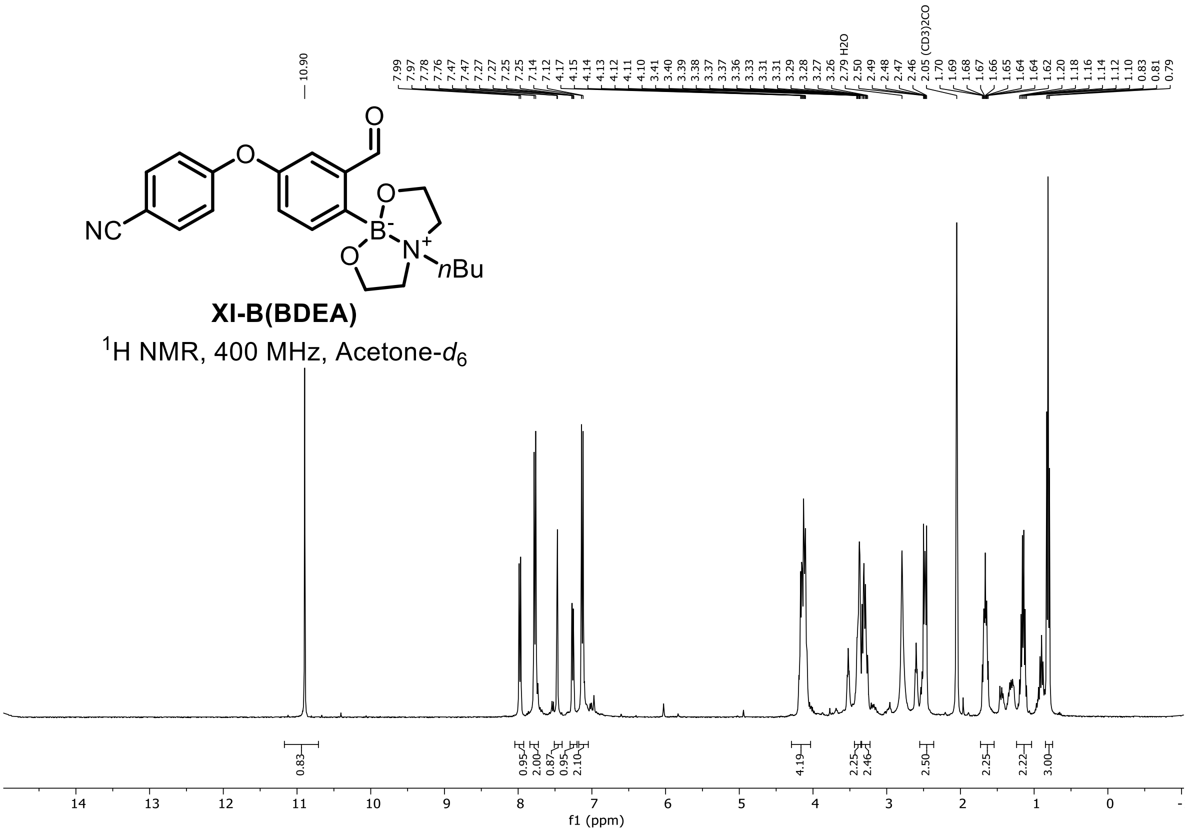

^{13}C NMR (101 MHz, Acetone-d_6) of **XI-B(BDEA)**

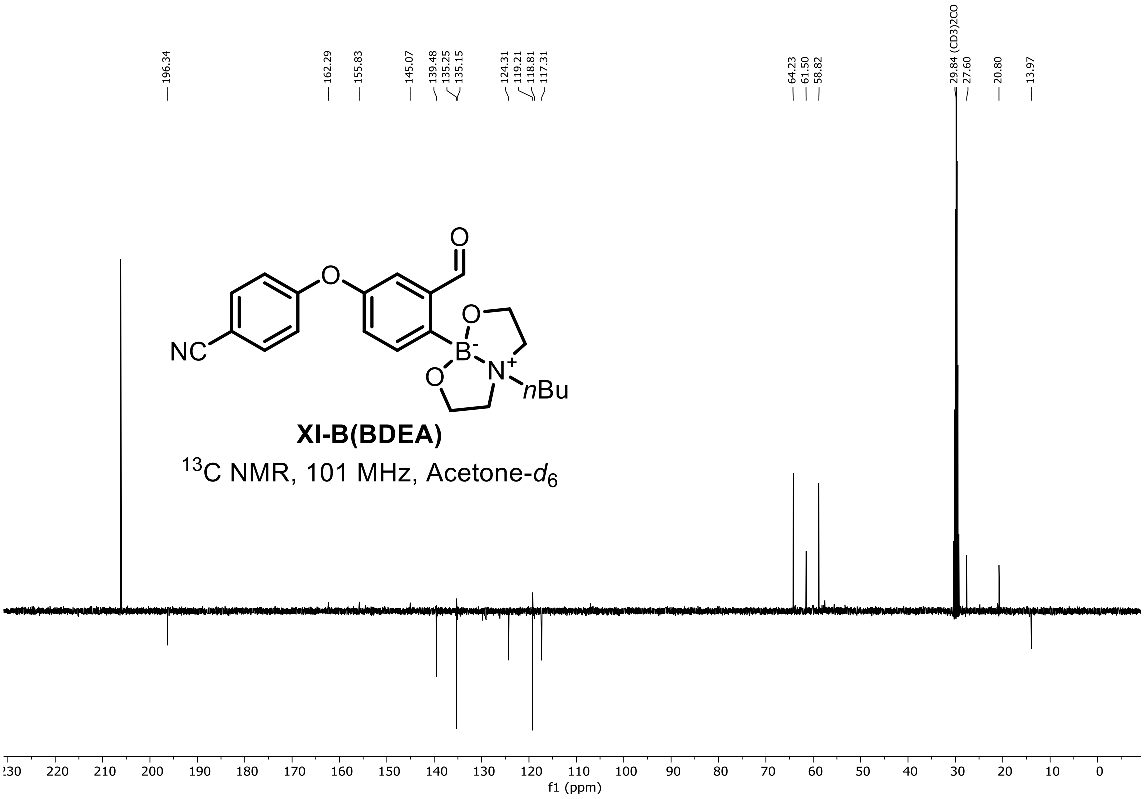

XI-B(BDEA)

^{13}C NMR, 101 MHz, Acetone-d_6

^{11}B NMR (128 MHz, Acetone-d_6) of **XI-B(BDEA)**

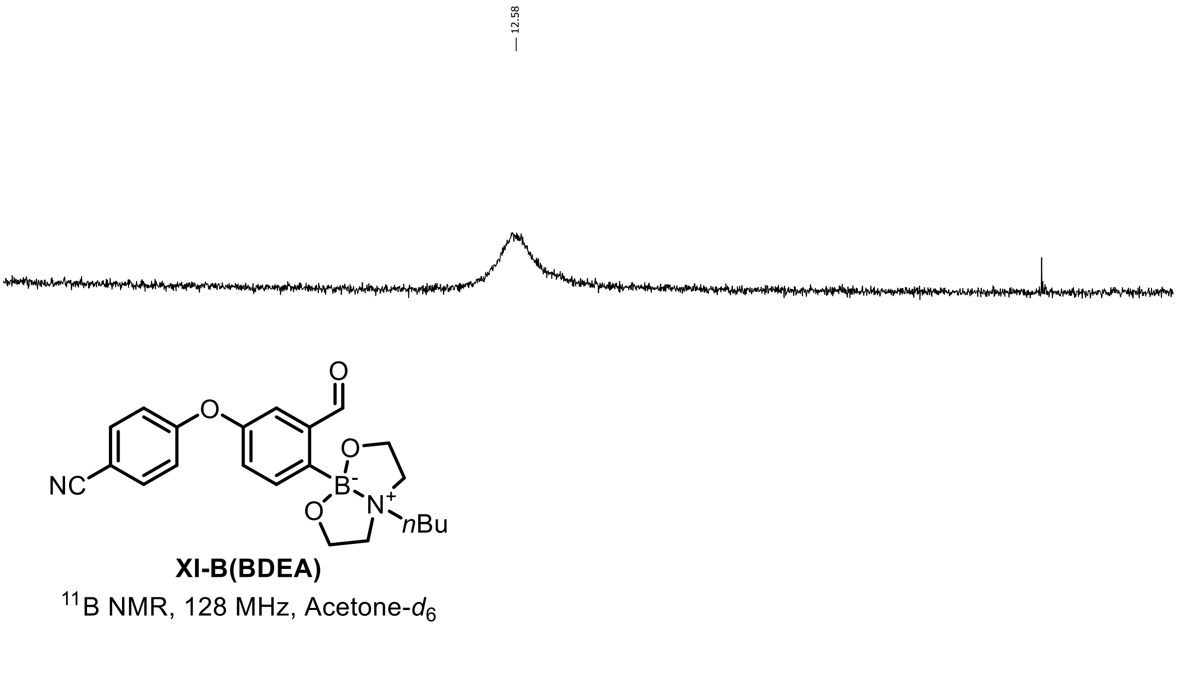

XI-B(BDEA)

^{11}B NMR, 128 MHz, Acetone-d_6

^{1}H NMR (400 MHz, $CDCl_3$) of **11** (see procedure)

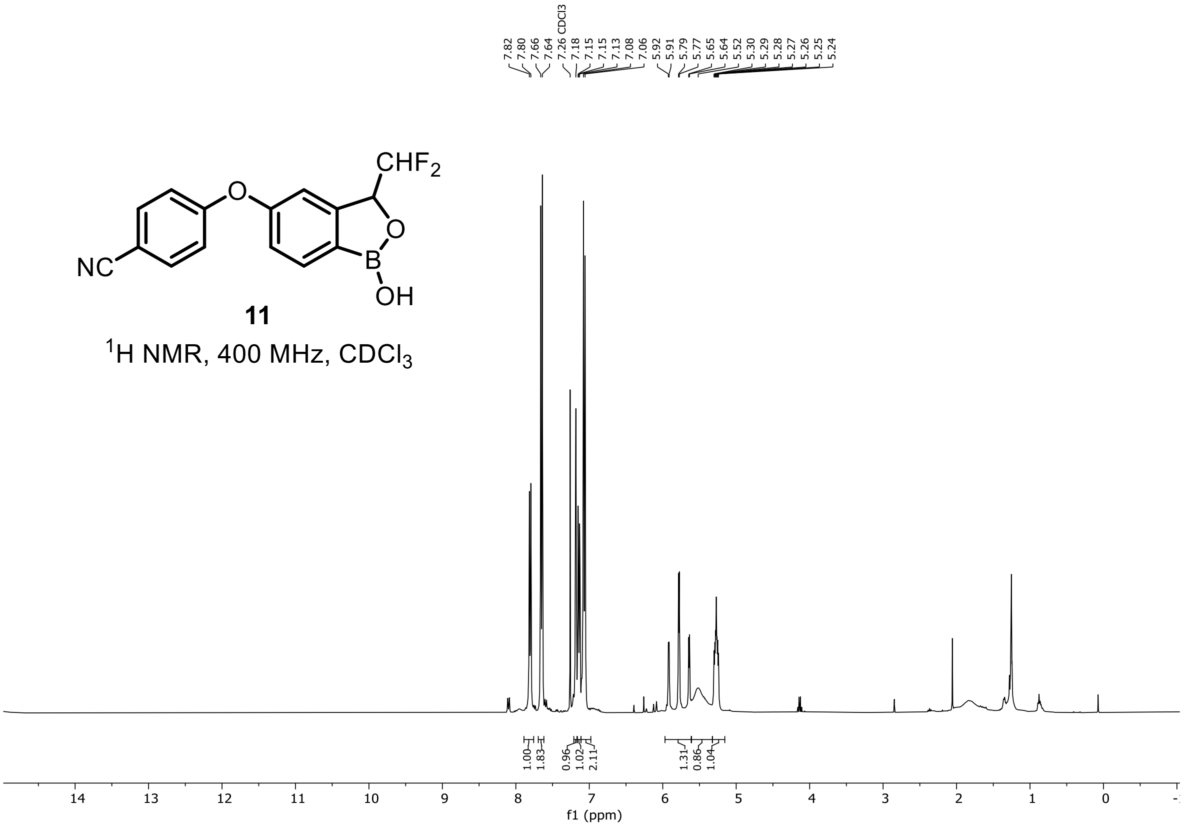

^{13}C NMR (101 MHz, $CDCl_3$) of **11**

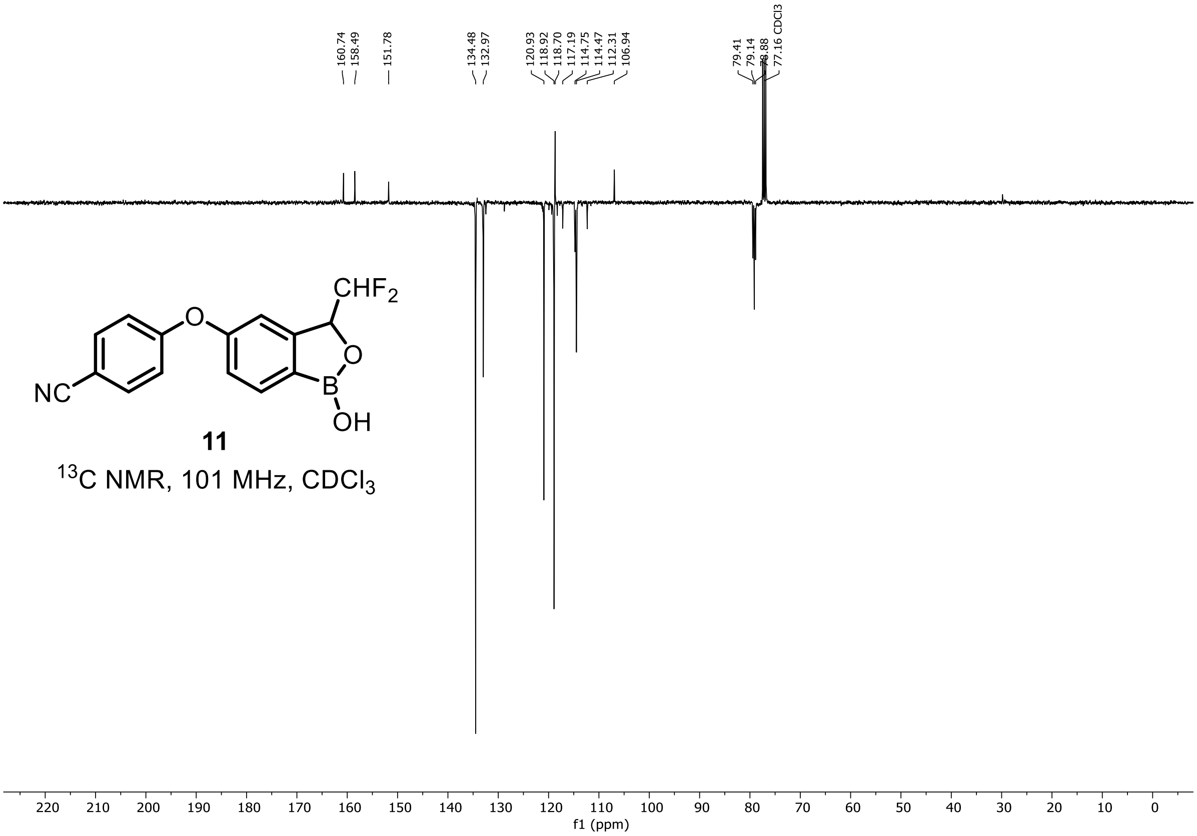

^{11}B NMR (128 MHz, $CDCl_3$) of **11**

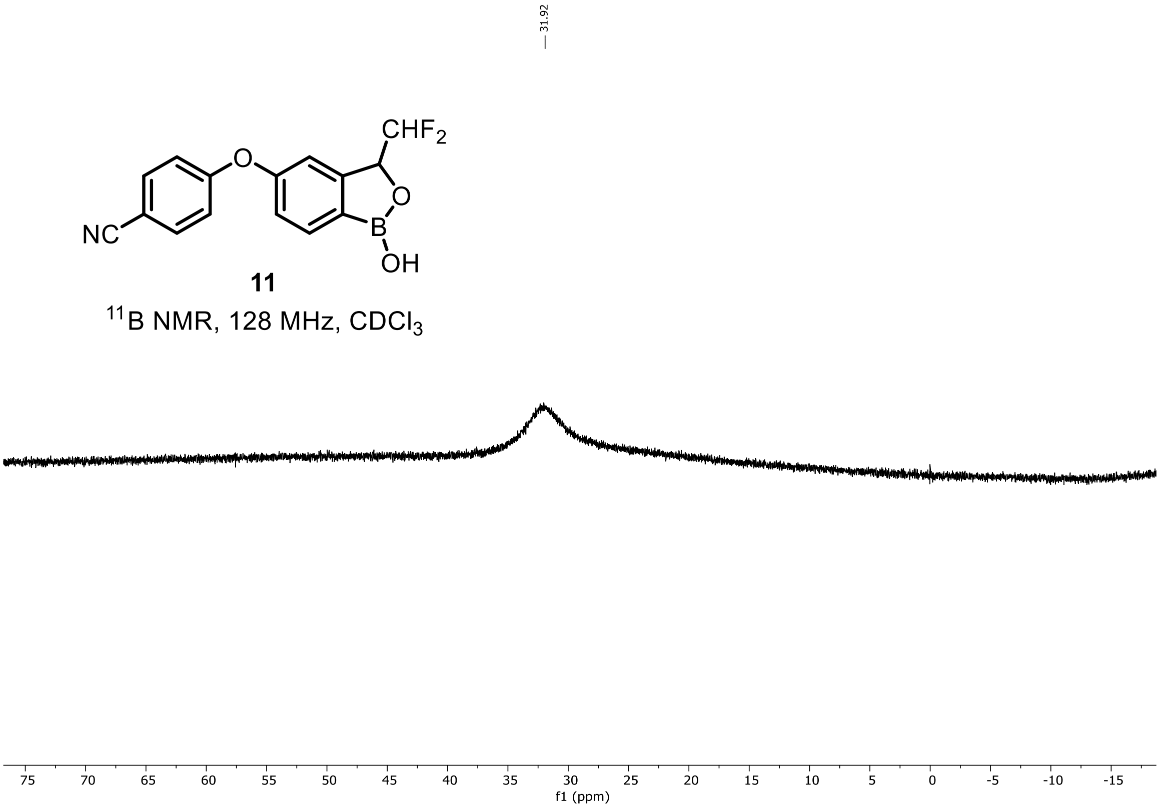

^{19}F NMR (376 MHz, $CDCl_3$) of **11**

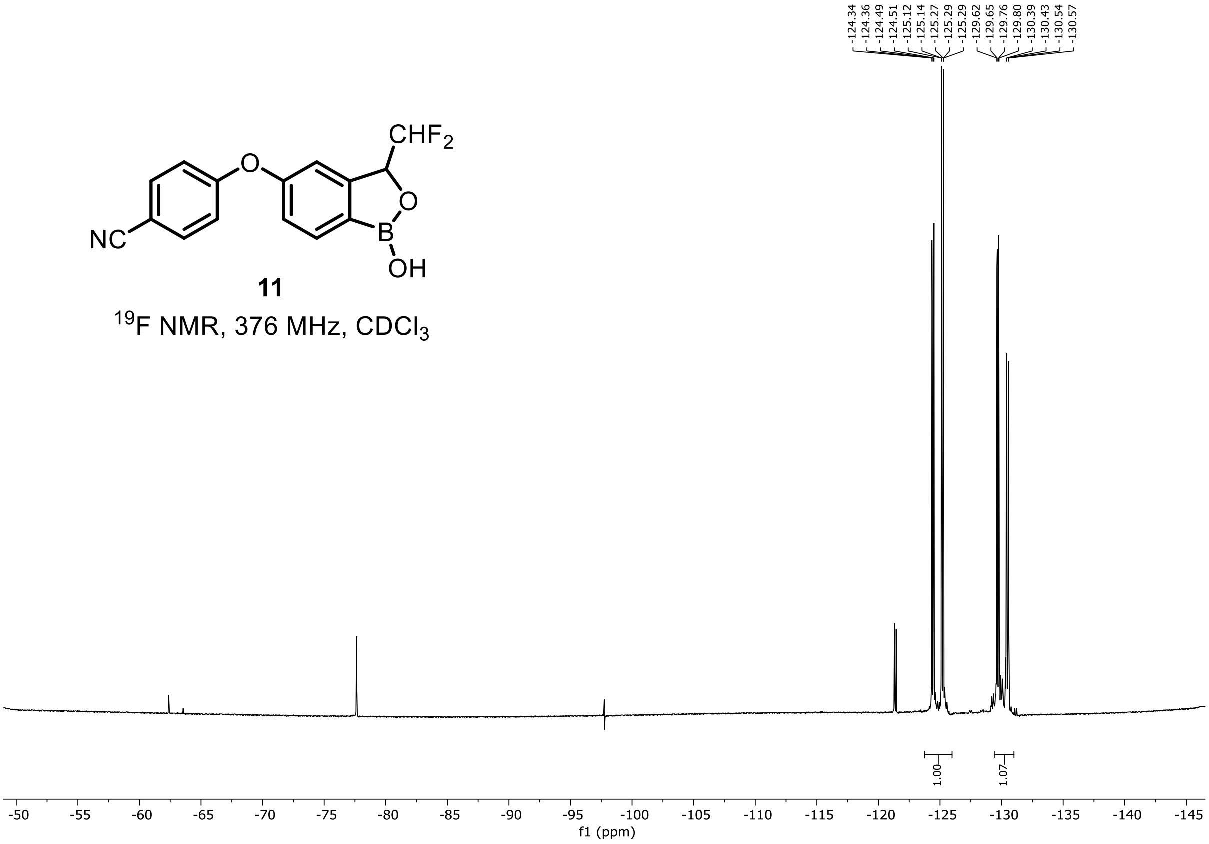

^{1}H NMR (400 MHz, Acetone-d_6) of **12** (see procedure)

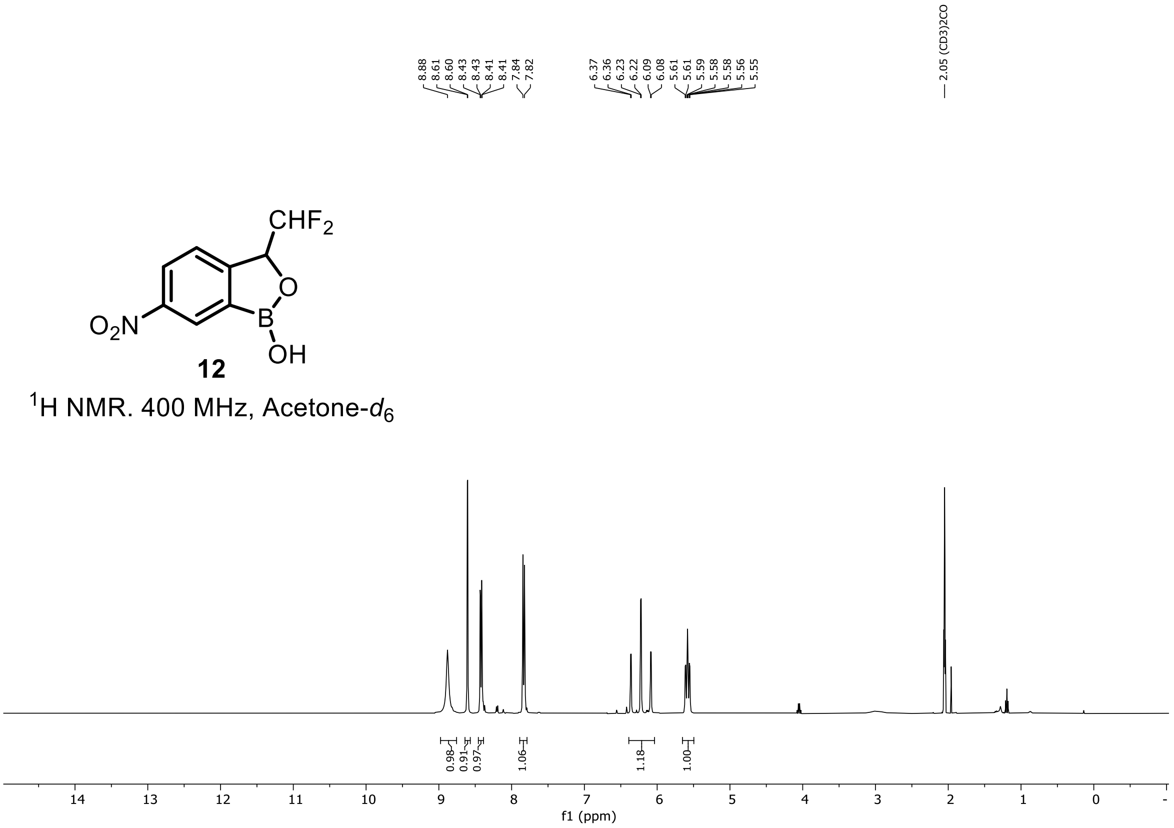

^{13}C NMR (101 MHz, Acetone-d_6) of **12**

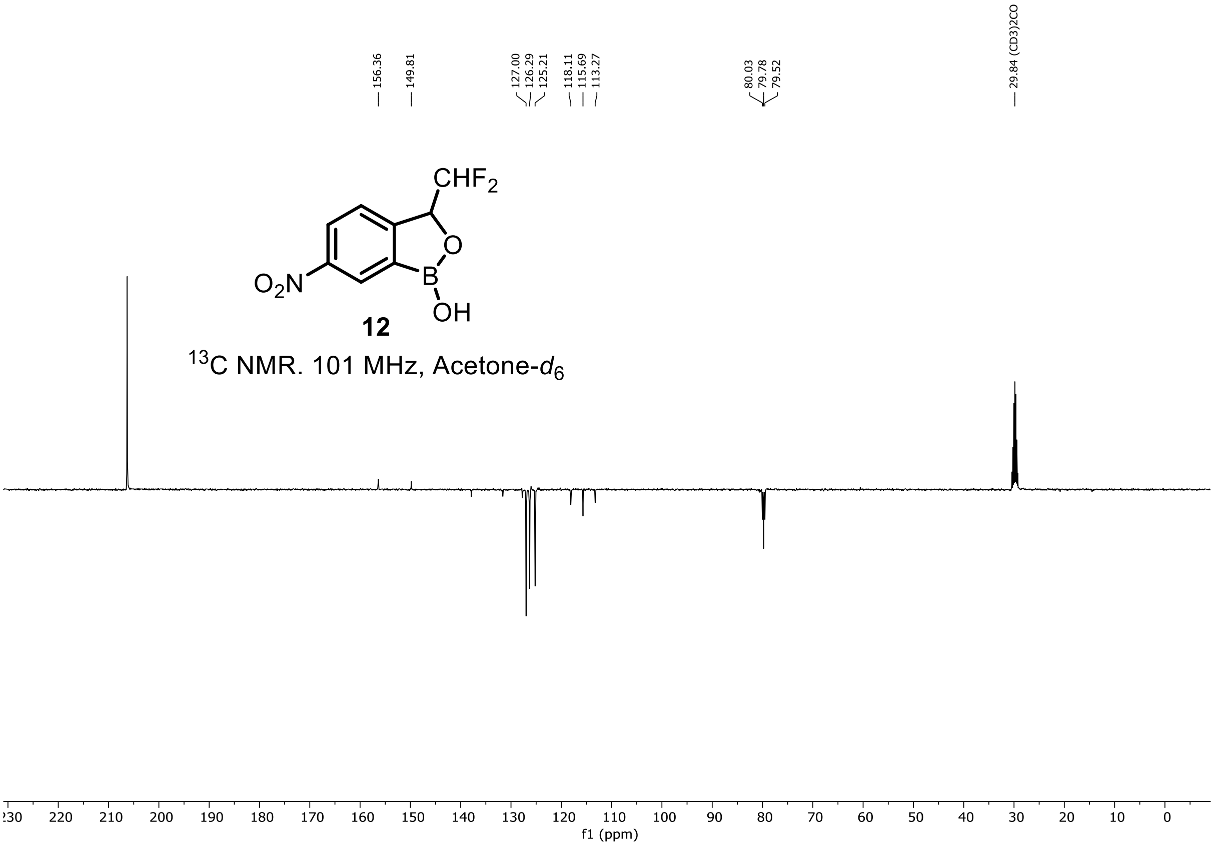

^{11}B NMR (128 MHz, Acetone-d_6) of **12**

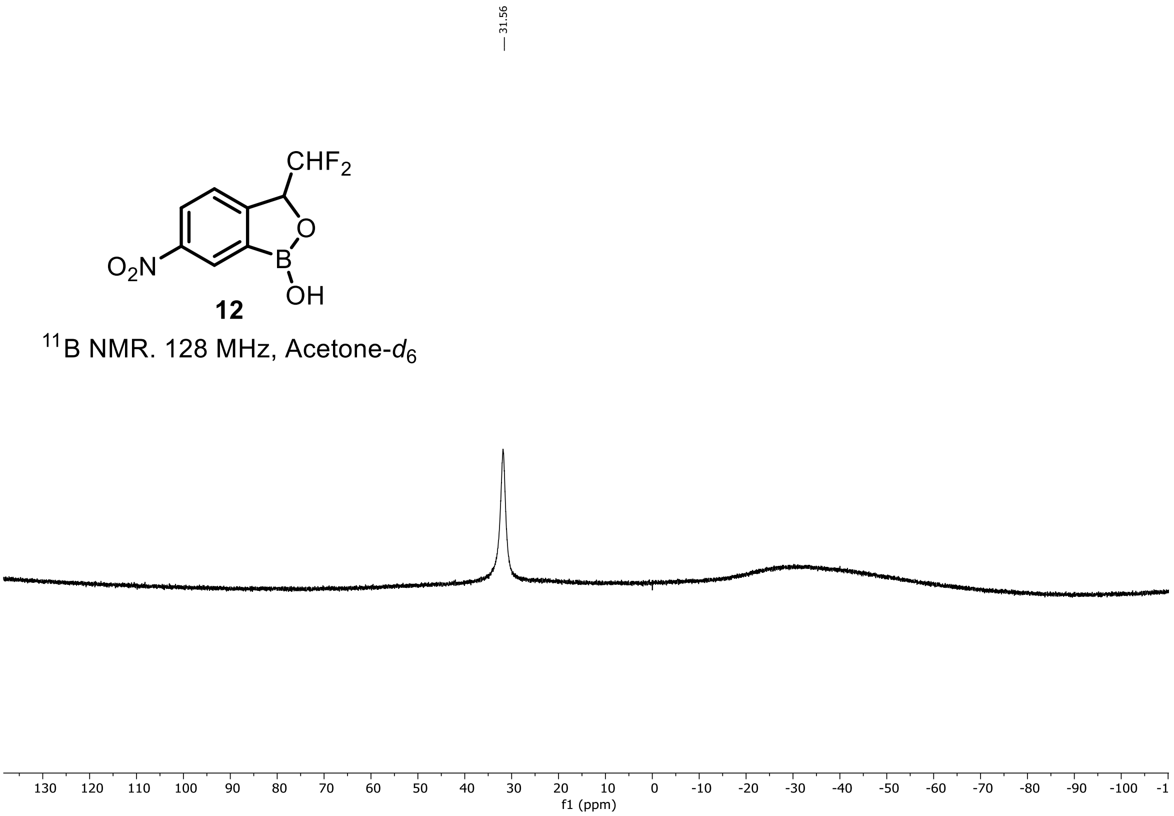

^{19}F NMR (376 MHz, Acetone-d_6) of **12**

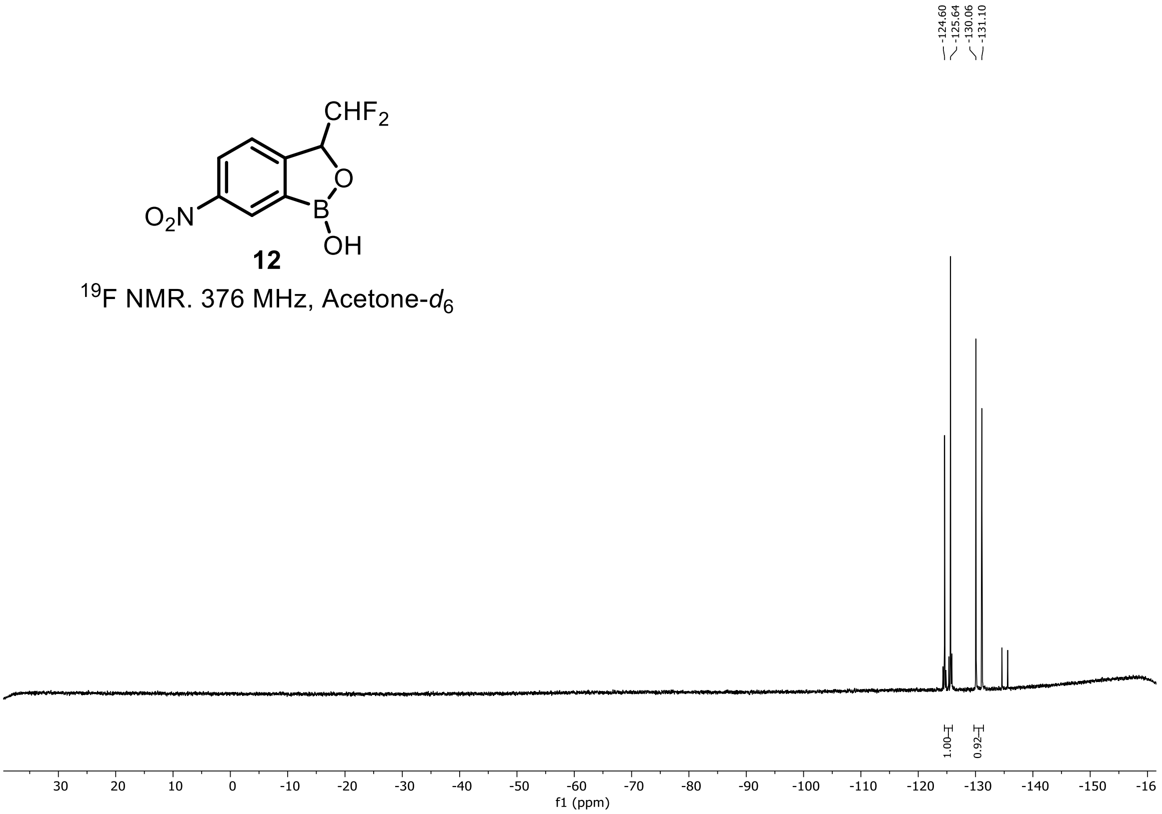

^1H NMR (400 MHz, CD_3OD) of **13** (see procedure)

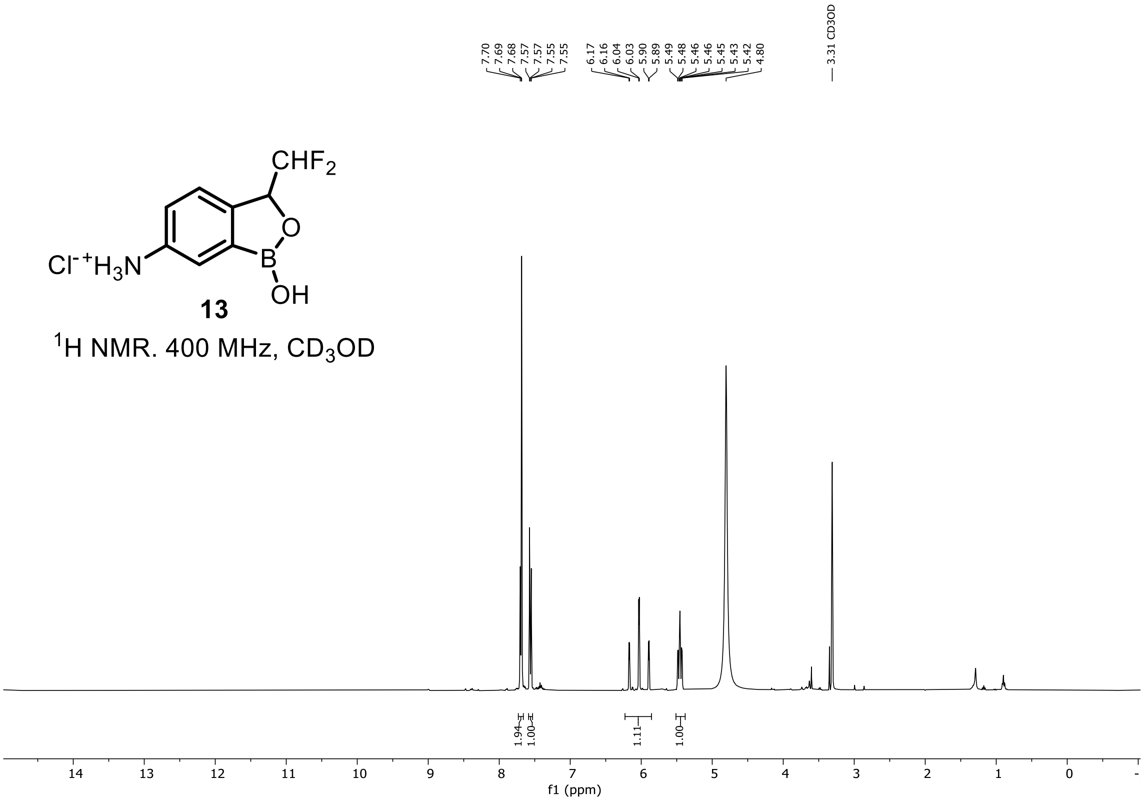

^{13}C NMR (101 MHz, CD_3OD) of **13**

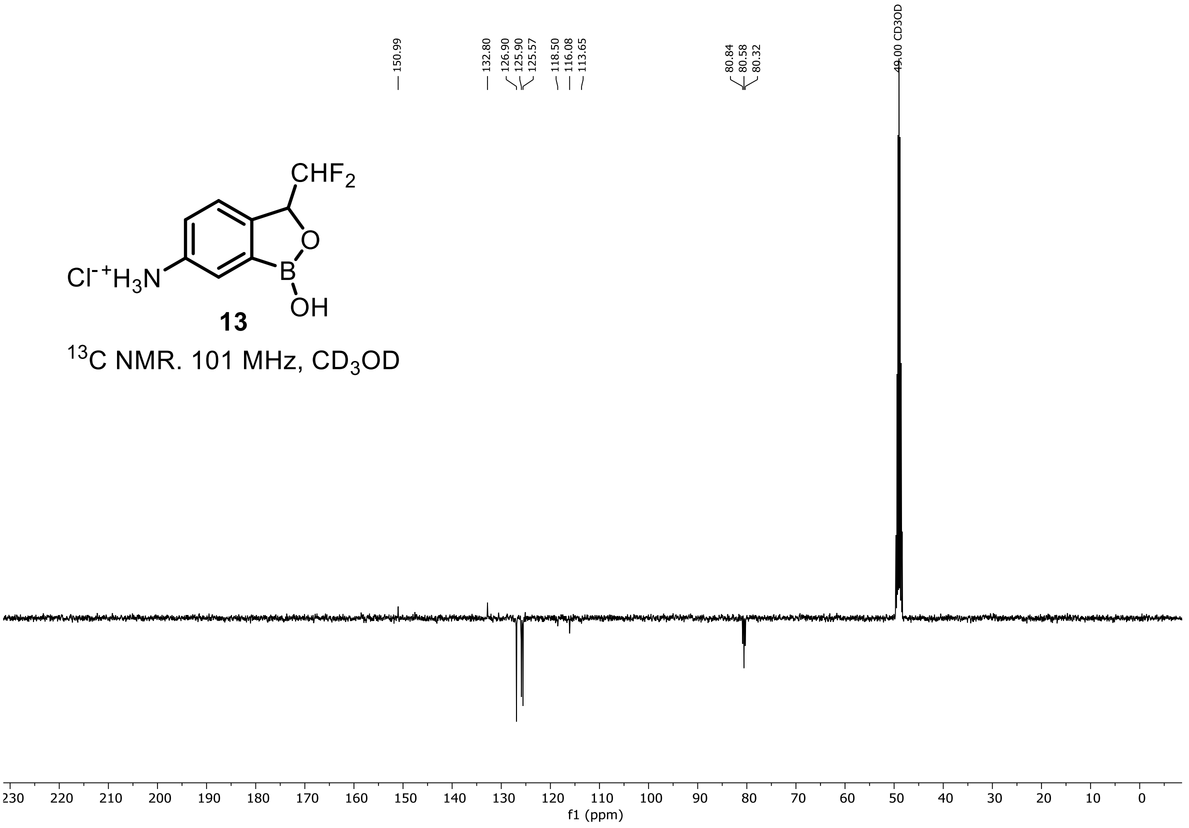

^{11}B NMR (128 MHz, CD_3OD) of **13**

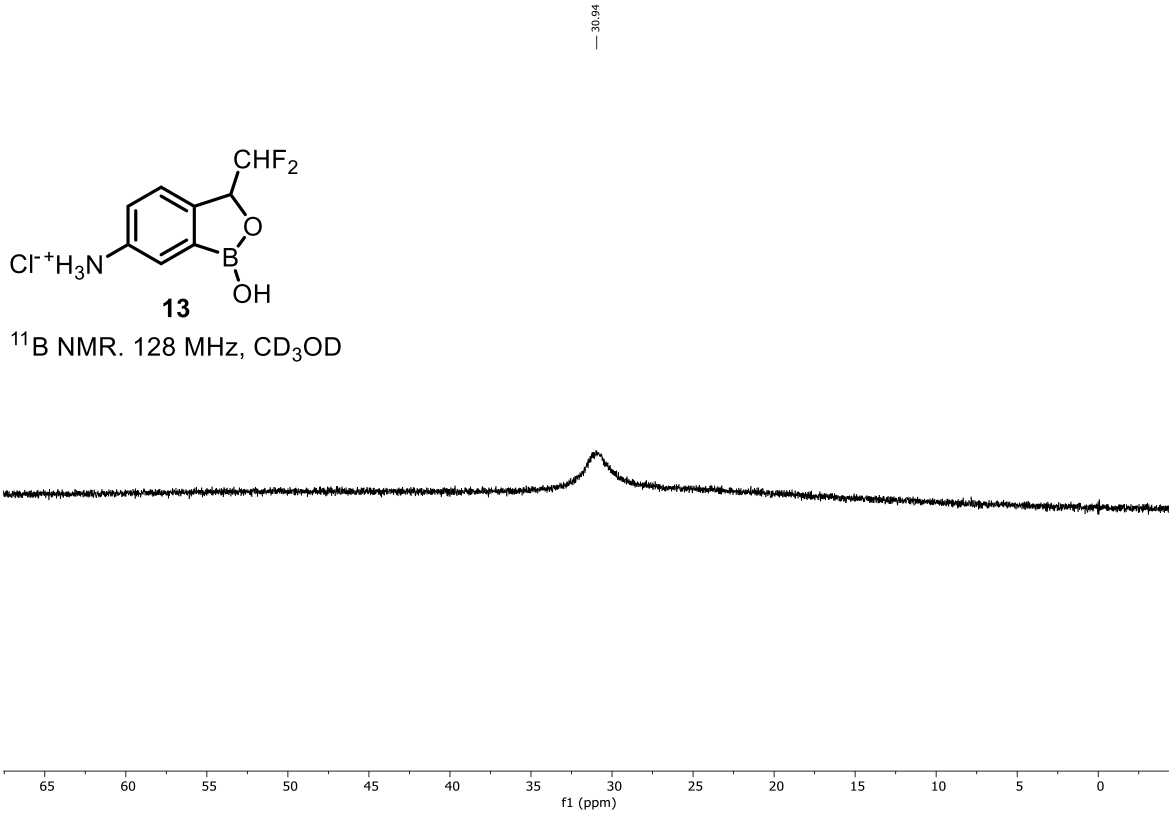

^{19}F NMR (376 MHz, CD_3OD) of **13**

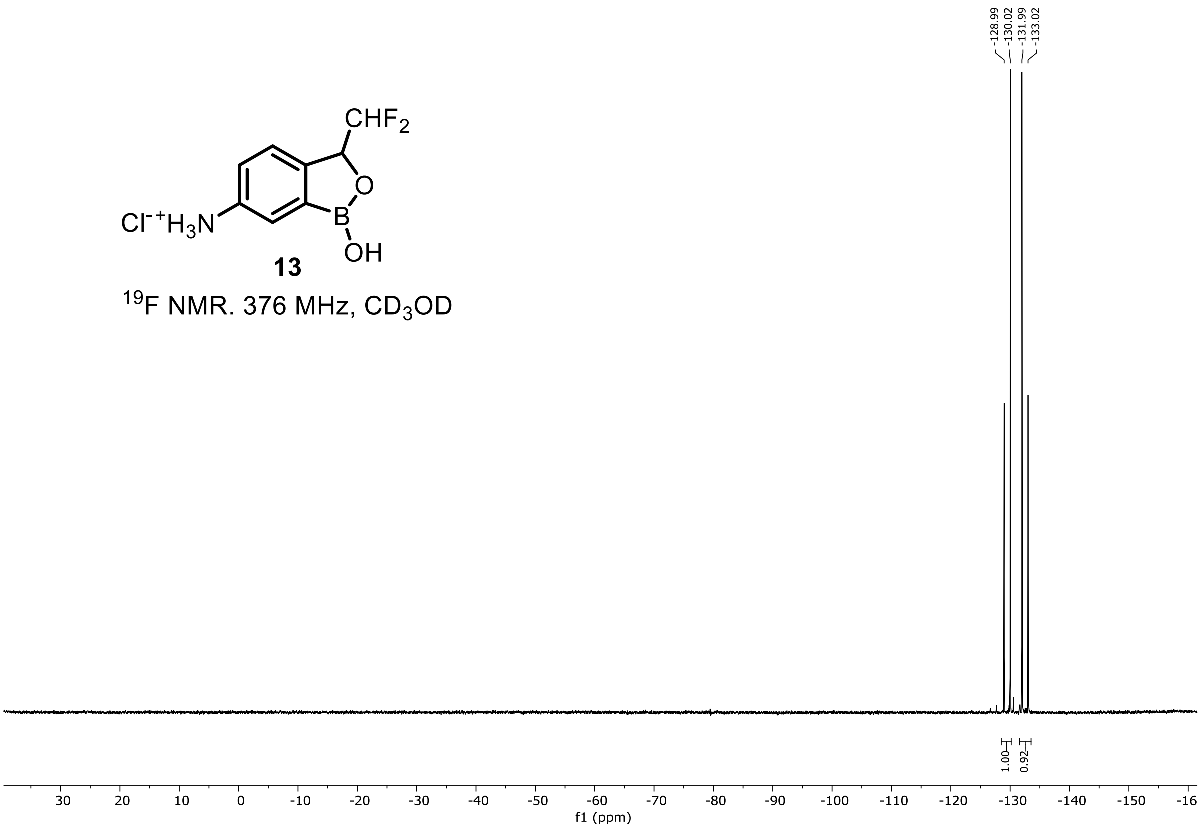

^{1}H NMR (400 MHz, CD_3OD) of **14** (see procedure)

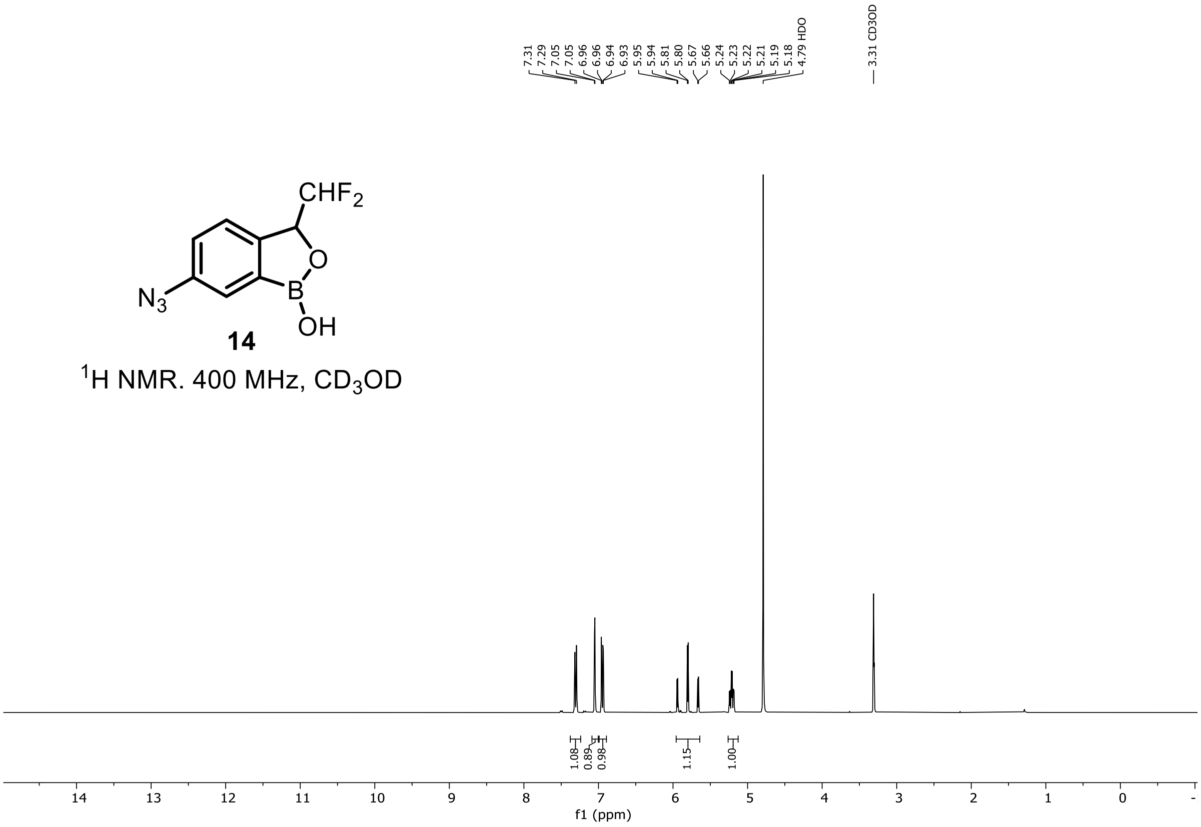

^{13}C NMR (101 MHz, CD_3OD) of **14**

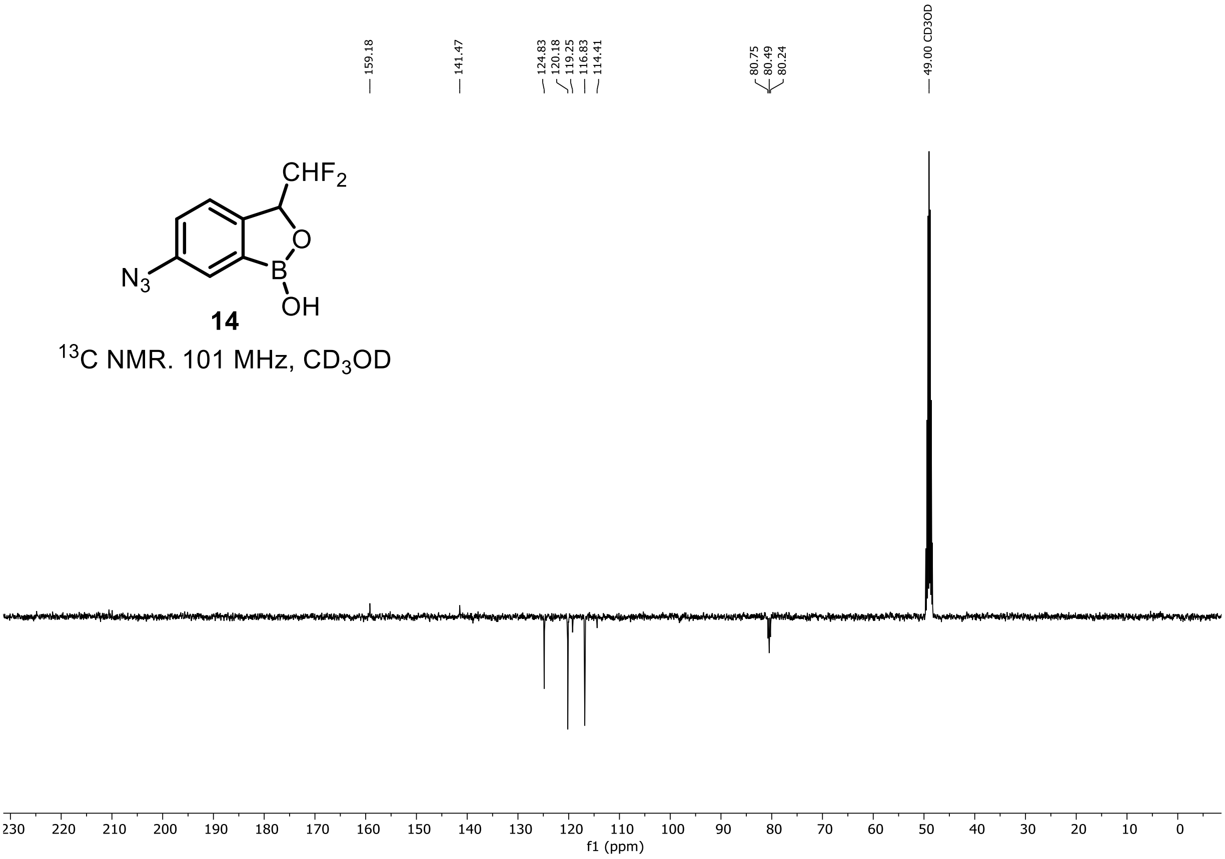

^{11}B NMR (128 MHz, CD_3OD) of **14**

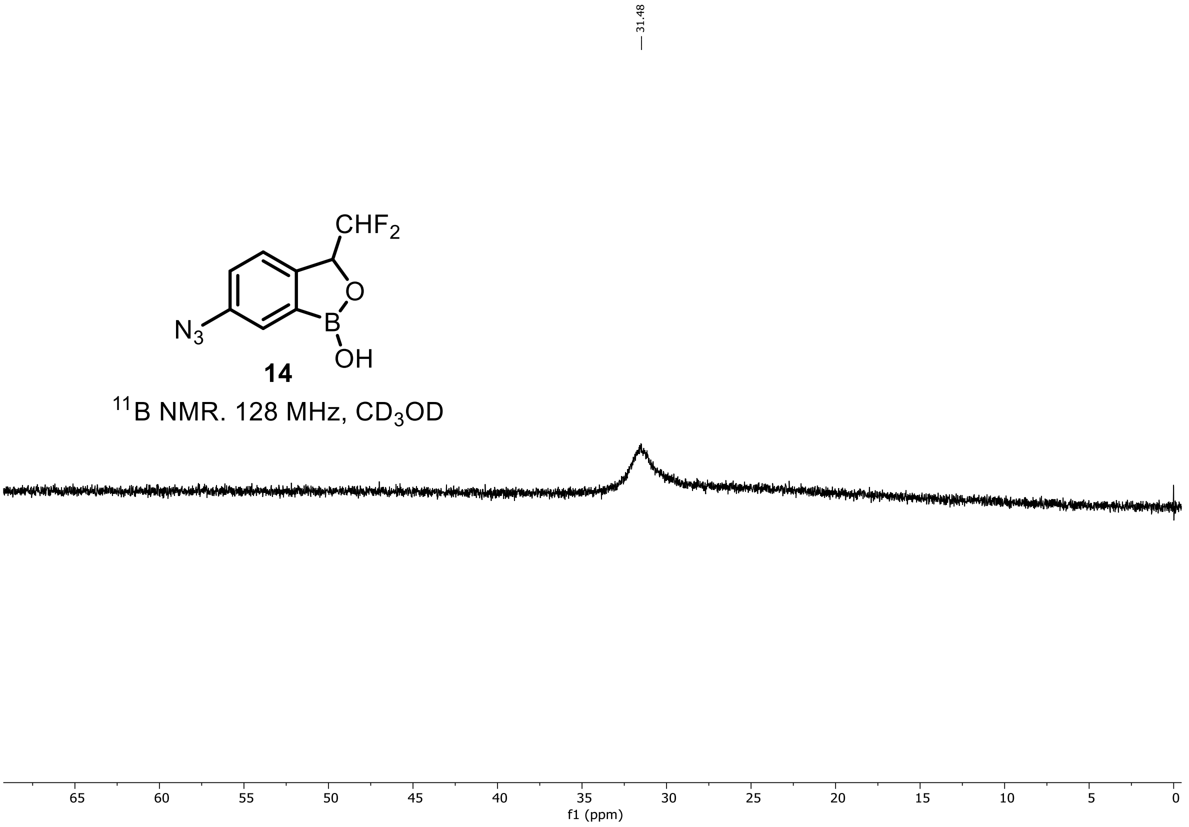

^{19}F NMR (376 MHz, CD_3OD) of **14**

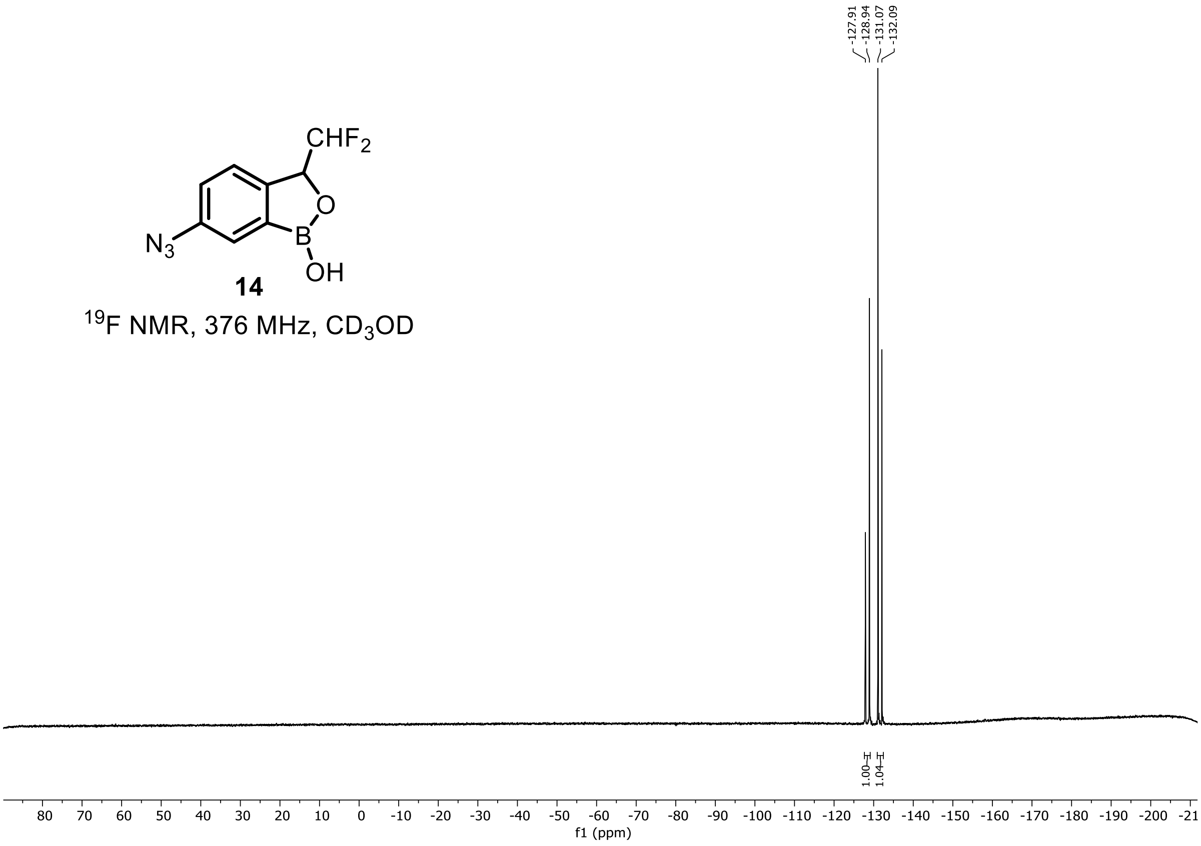

^{1}H NMR (400 MHz, $CDCl_3$) of **I-[B$_a$*]** (see procedure)

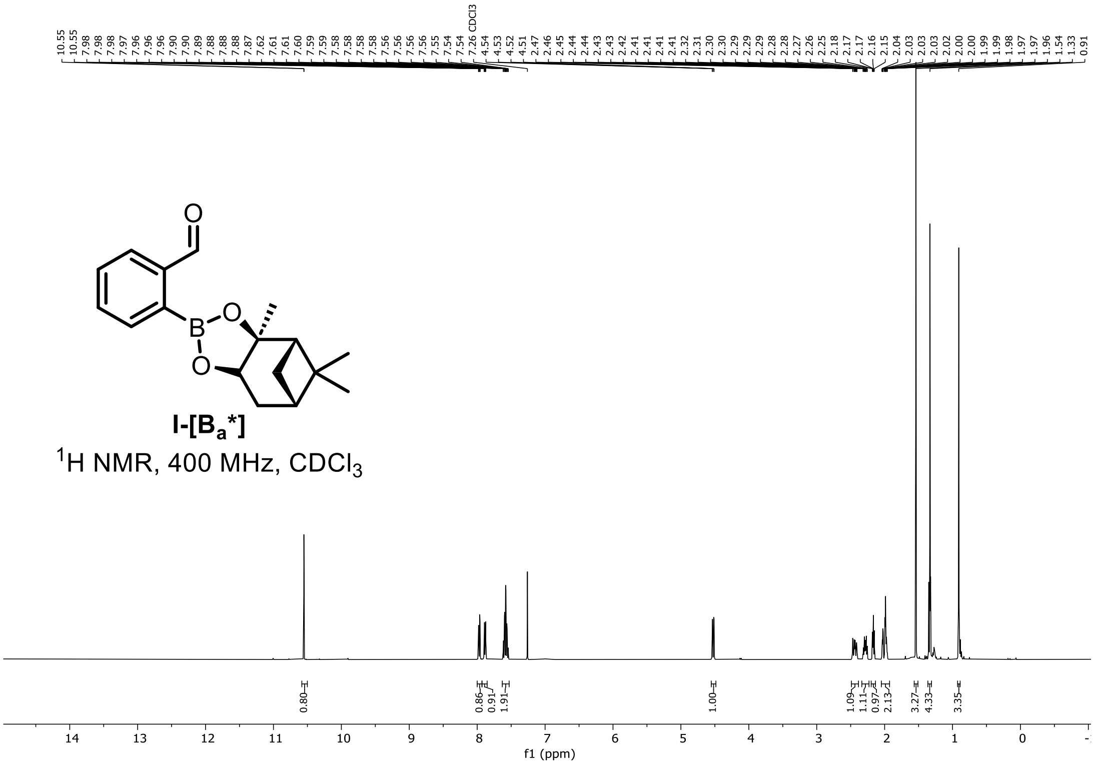

^{13}C NMR (101 MHz, $CDCl_3$) of **I-[B$_a$*]**

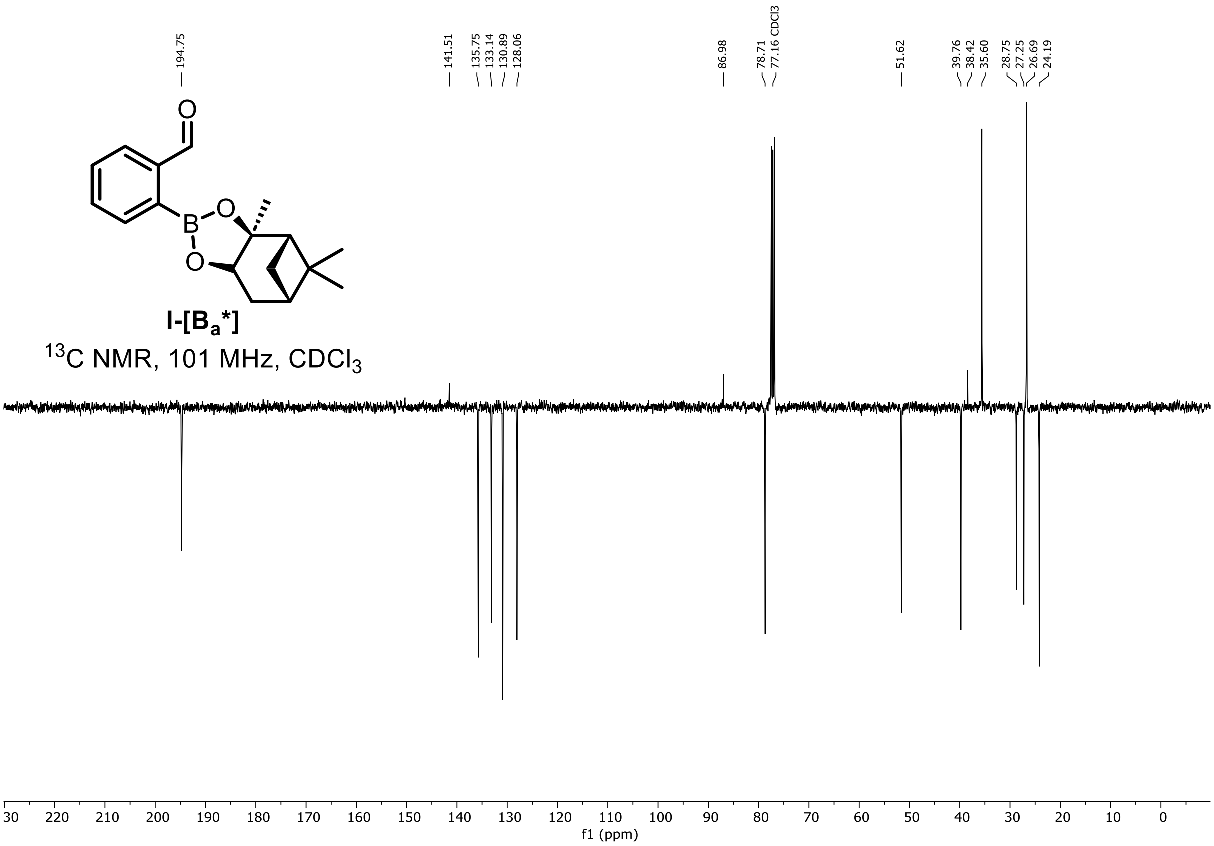

^{11}B NMR (128 MHz, CDCl$_3$) of **I-[B$_a$*]**

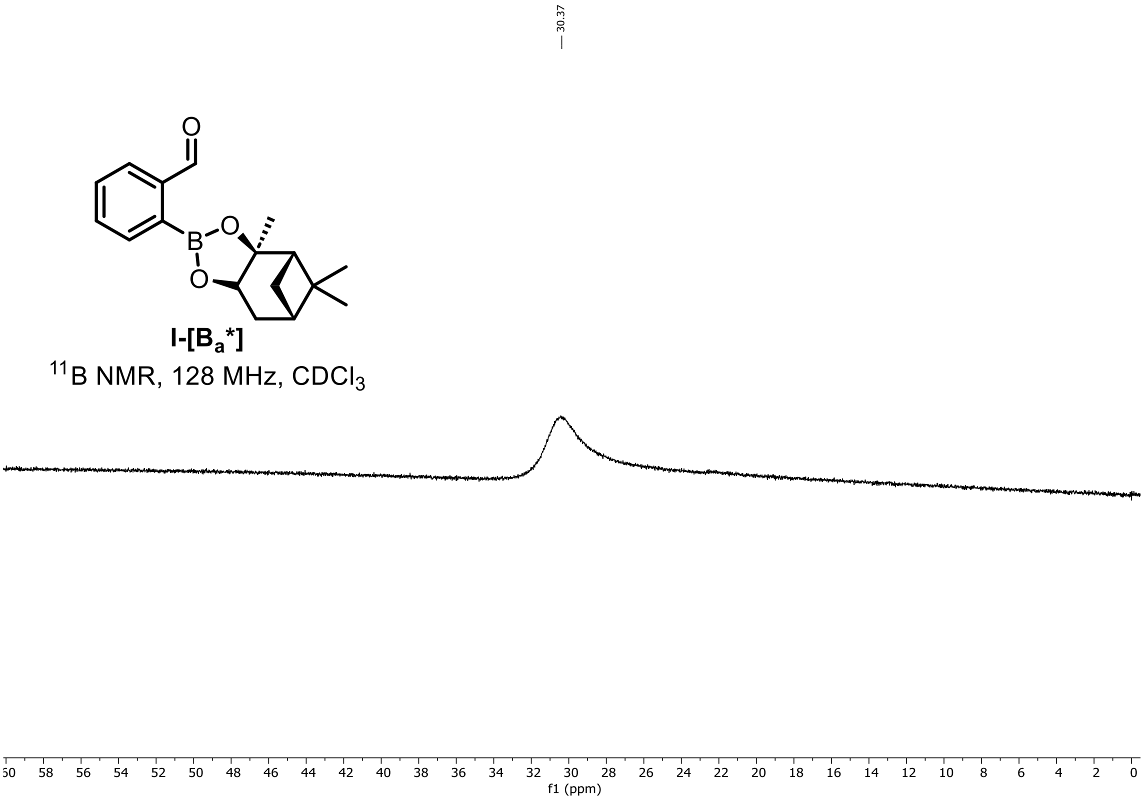

^{1}H NMR (400 MHz, CDCl$_3$) of ***n*-BDIA** (see procedure)

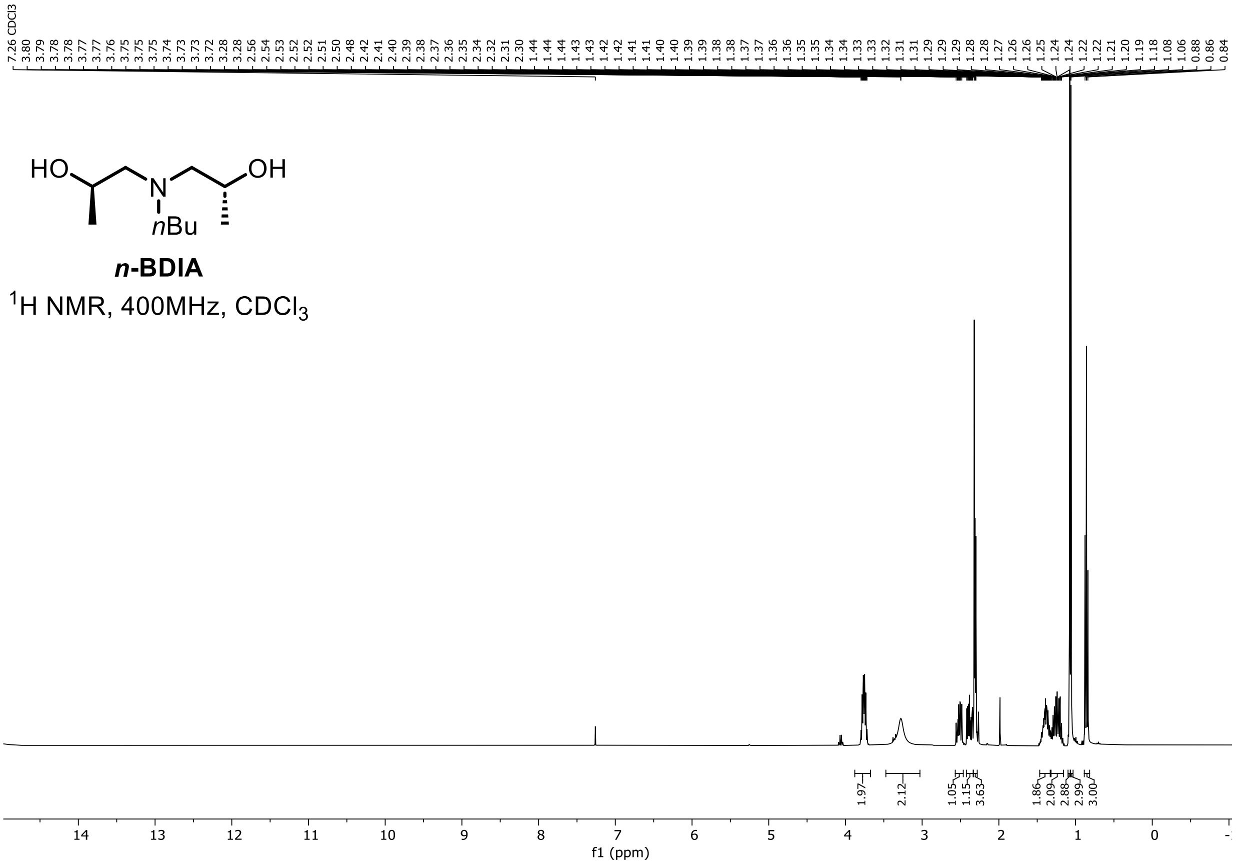

^{1}H NMR (400 MHz, Acetone-d_6) of **I-[B$_b$*]** (see procedure)

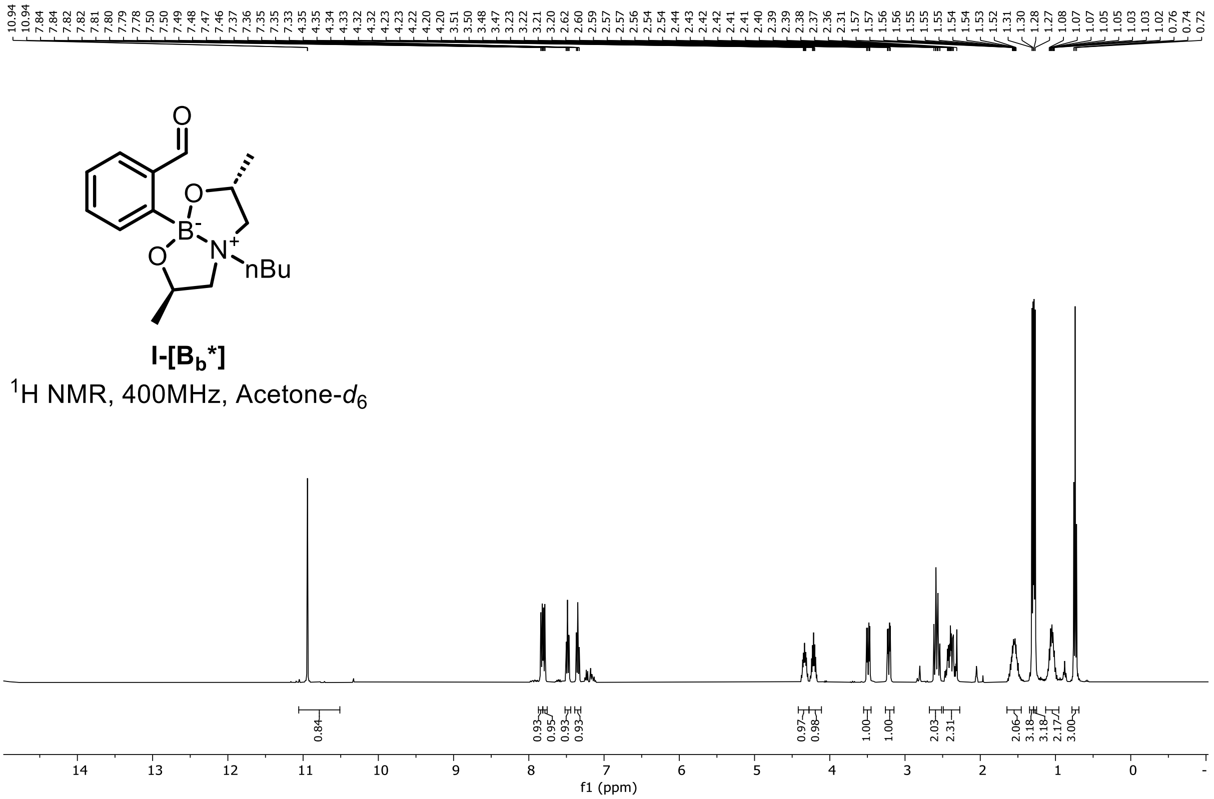

^{13}C NMR (101 MHz, Acetone-d_6) of **I-[B$_b$*]**

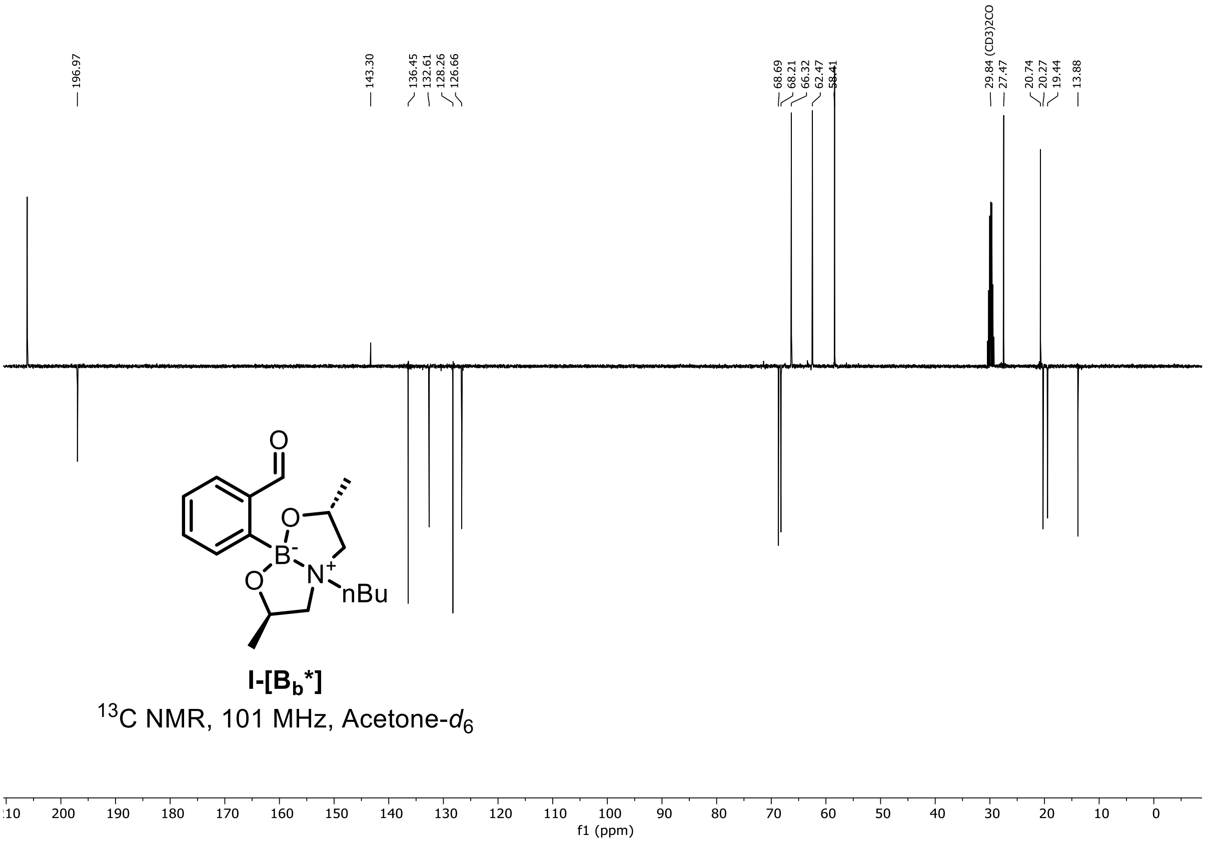

^{11}B NMR (128 MHz, Acetone-d_6) of **I-[B$_b$*]**

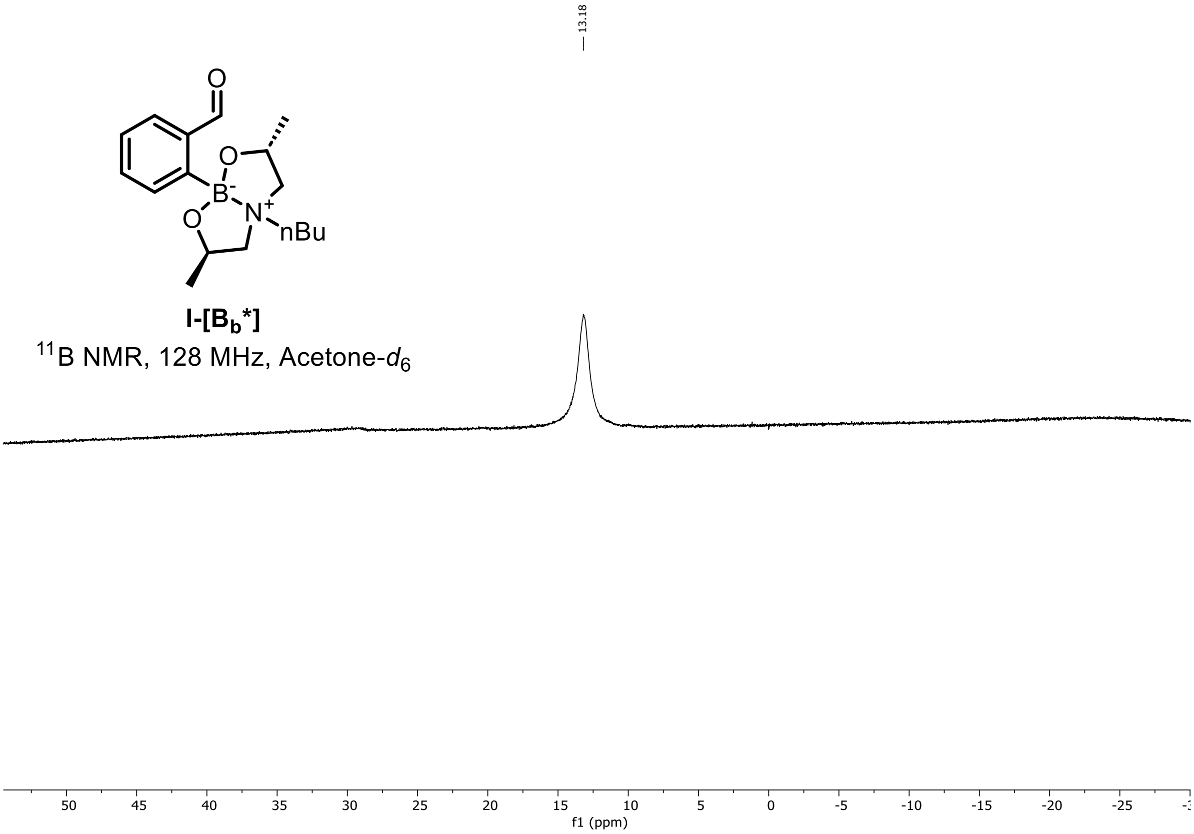

^{1}H NMR (400 MHz, $CDCl_3$) of **15** (see procedure)

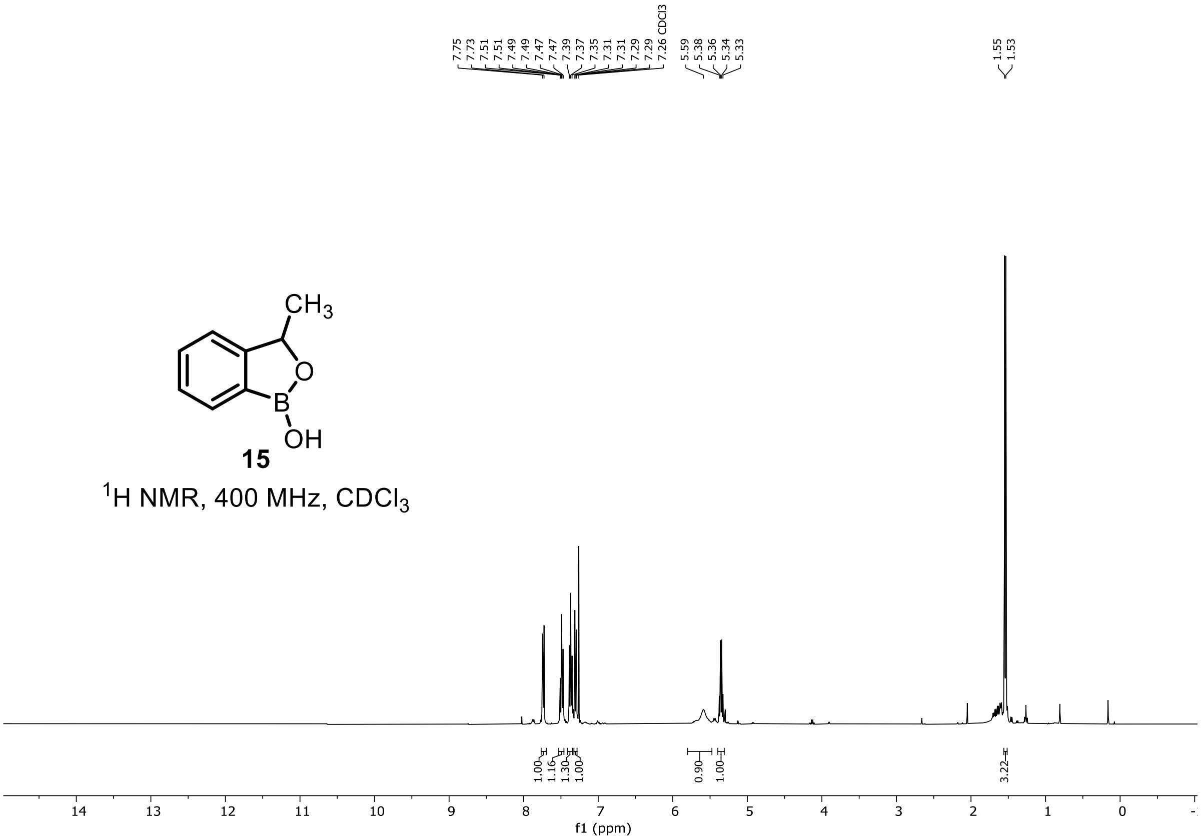

^{11}B NMR (128 MHz, $CDCl_3$) of **15**

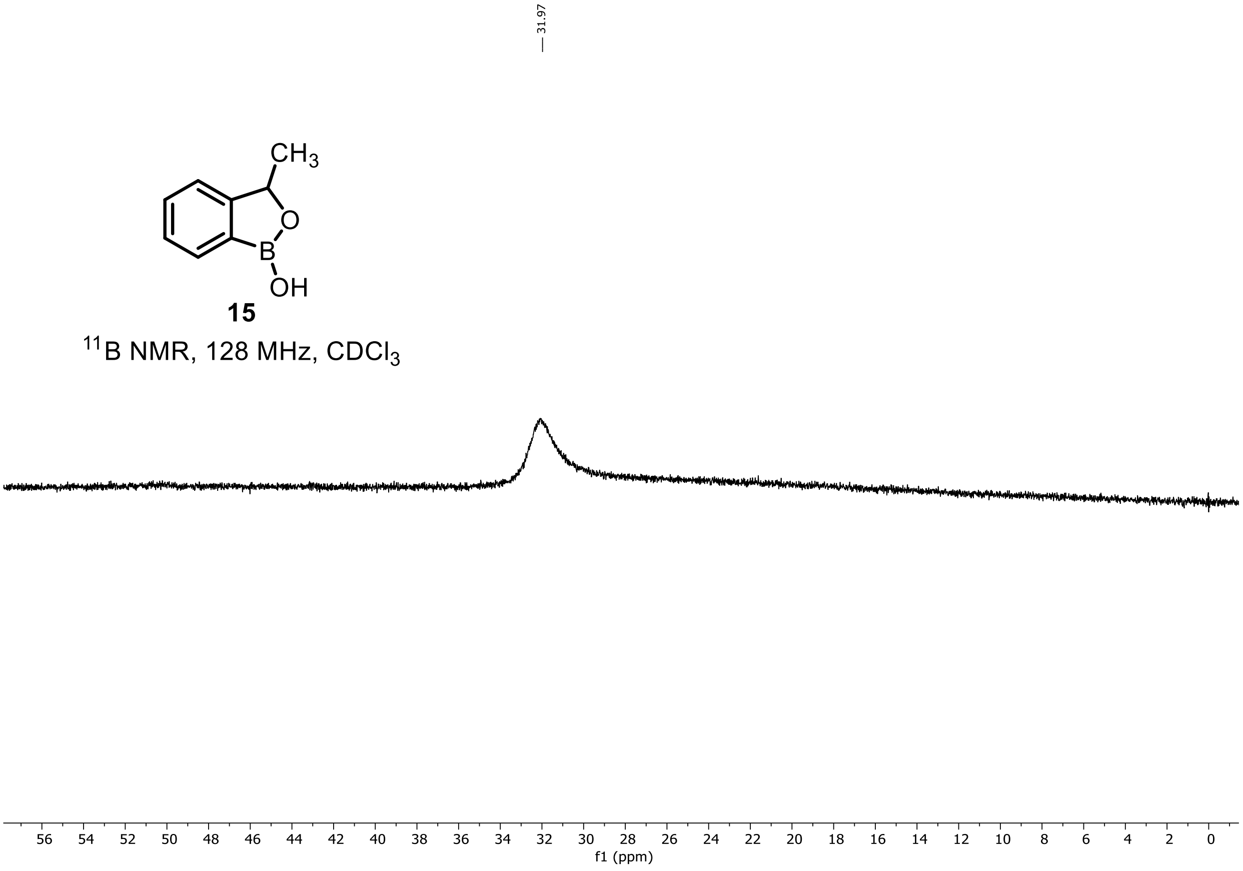

^{1}H NMR (400 MHz, DMSO-d_6) of **1** vs **1** after one week (see procedure)

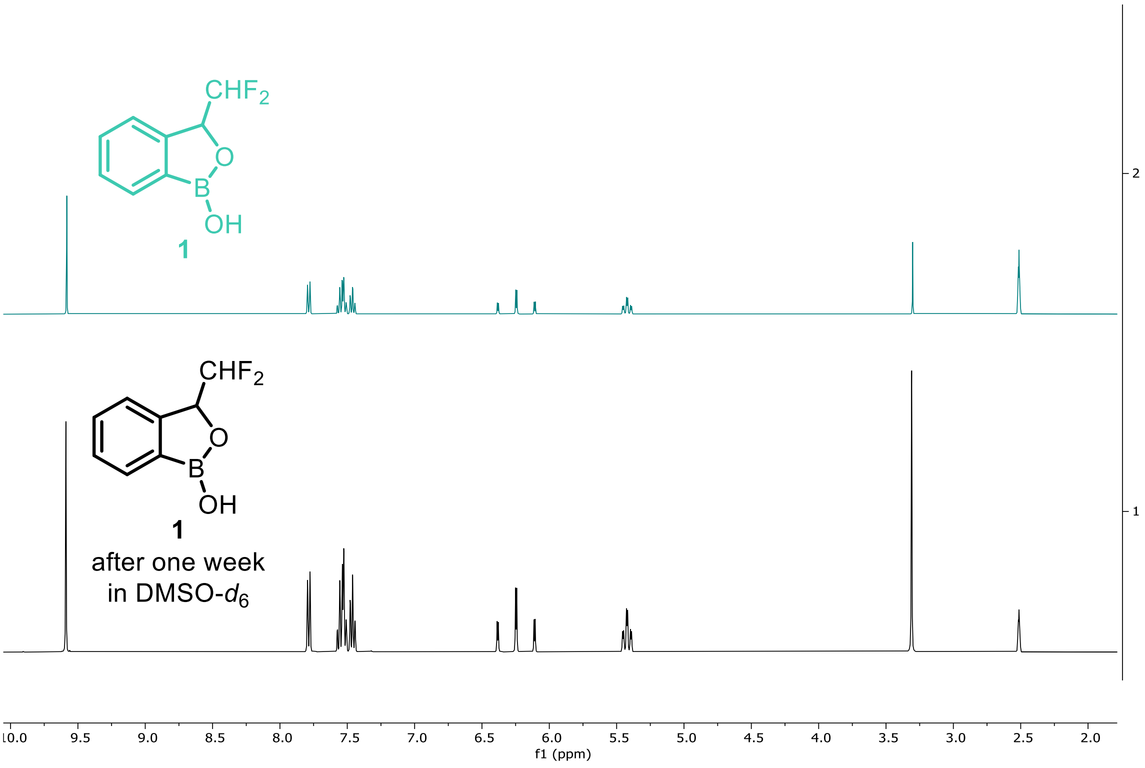

^{1}H NMR (400 MHz, DMSO-d$_6$) of **1** (see procedure)

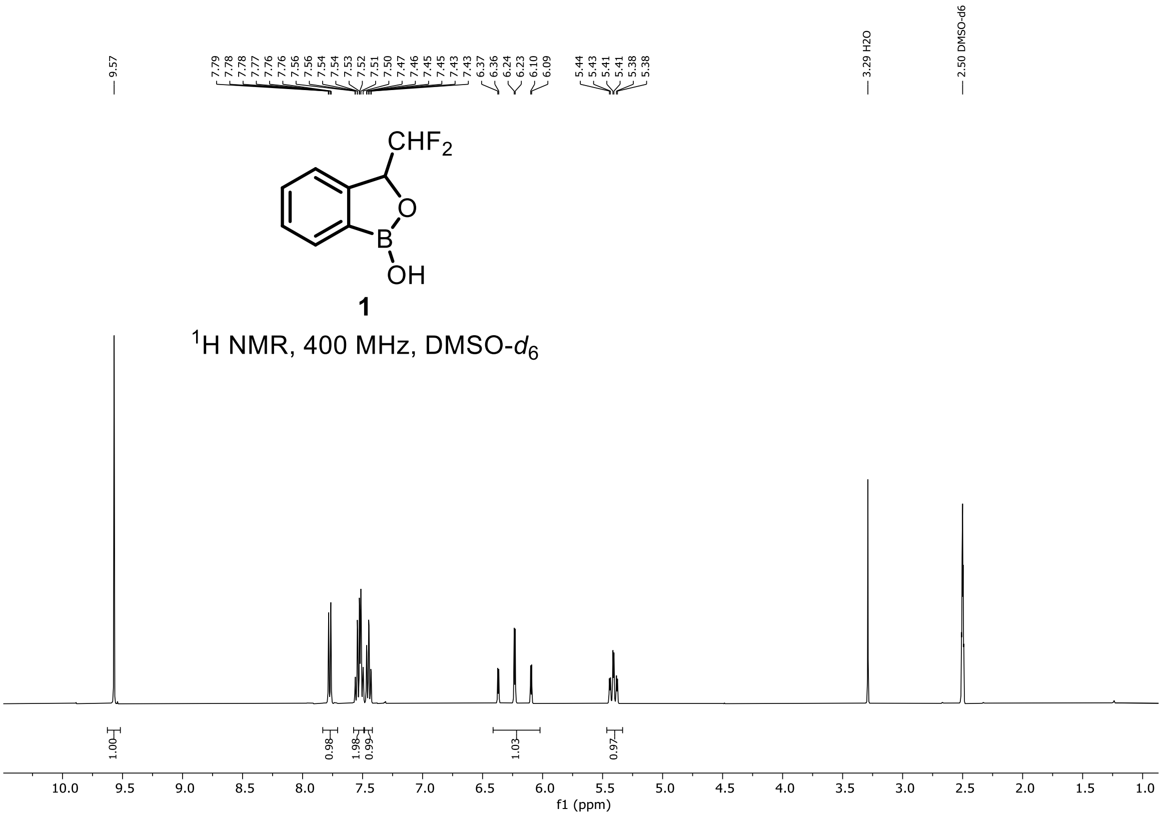

^{1}H NMR (400 MHz, $CDCl_3$) of **1** vs **1** after two months on air

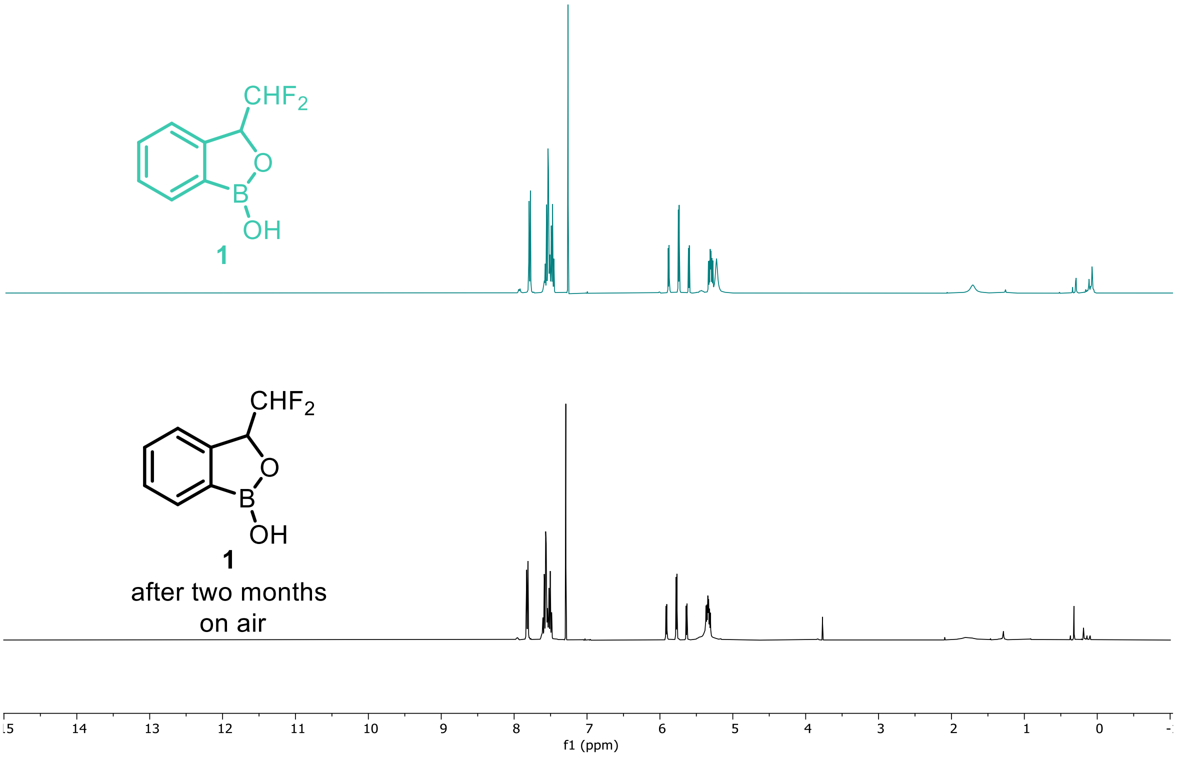

^{1}H NMR of **16a** in equilibrium whit **16b** (400 MHz, DMSO-d_6) (see biological evaluation)

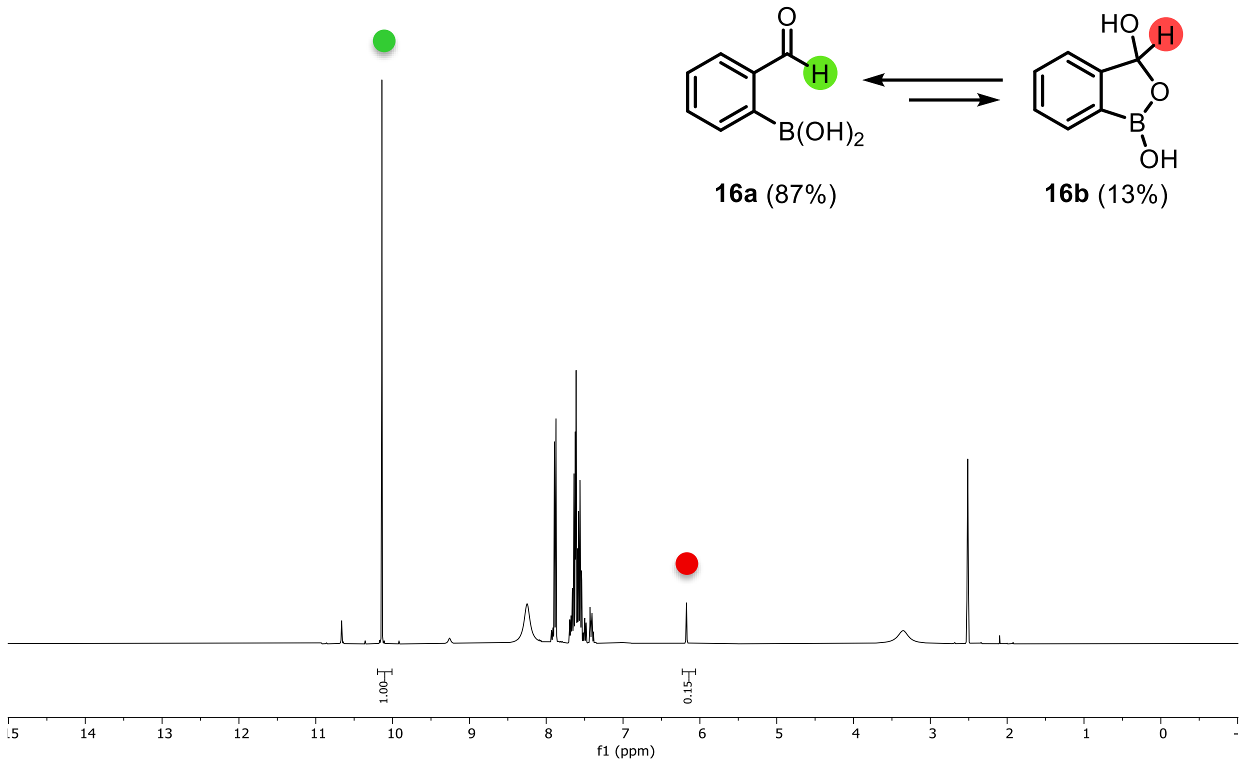

^{1}H NMR of **17a** in equilibrium whit **17b** (400 MHz, DMSO-d_6) (see biological evaluation)

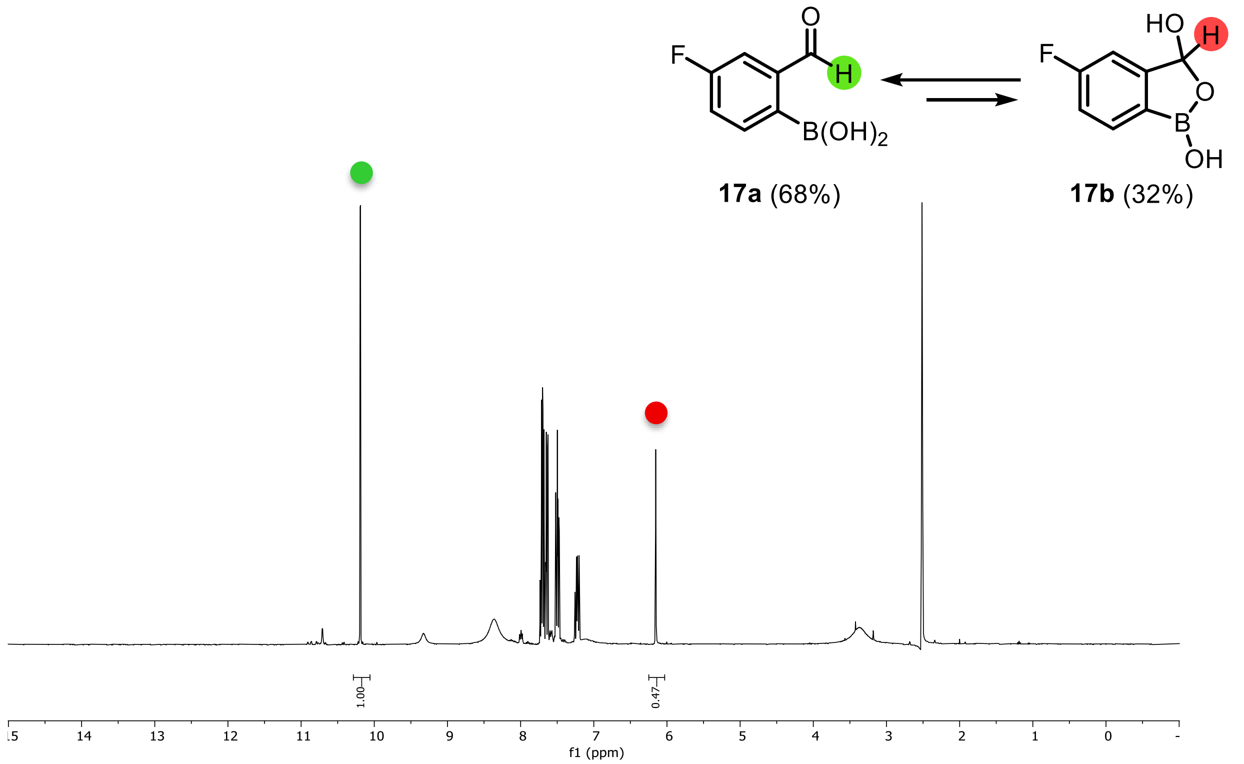

4. HPLC TRACES

Chiral HPLC trace of **1** (see procedure)

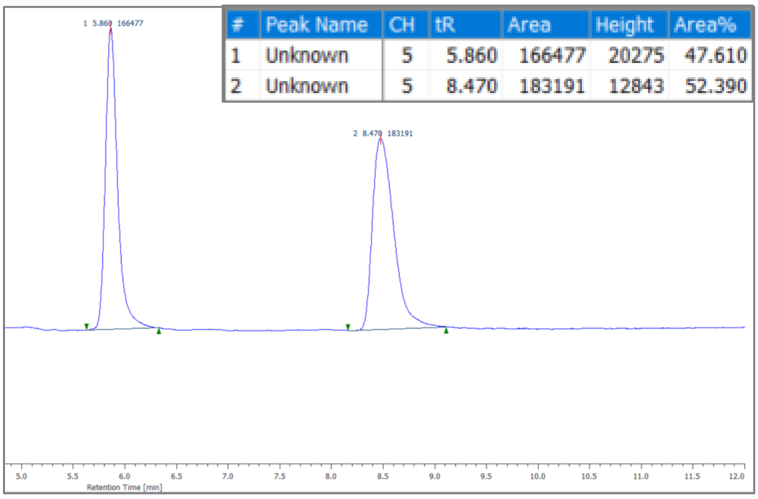

#	Peak Name	CH	tR	Area	Height	Area%
1	Unknown	5	5.860	166477	20275	47.610
2	Unknown	5	8.470	183191	12843	52.390

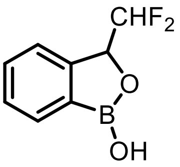

1
racemic mixture

Chiral HPLC trace of **(+)-1** (e.r 53:47) (see procedure)

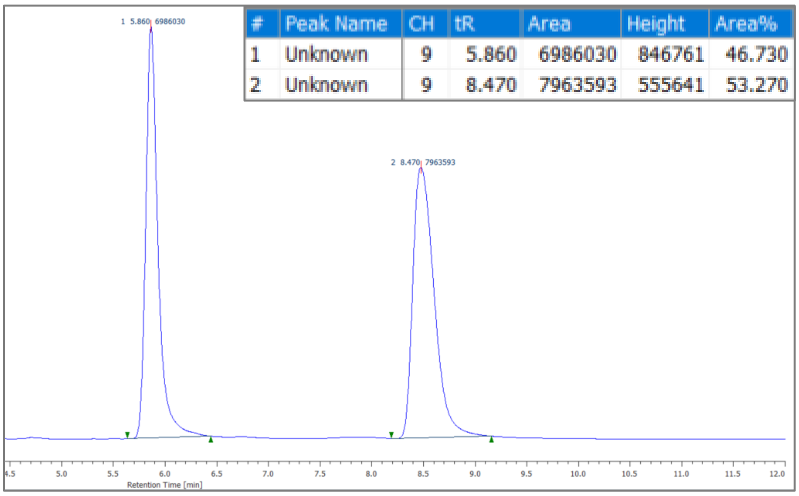

#	Peak Name	CH	tR	Area	Height	Area%
1	Unknown	9	5.860	6986030	846761	46.730
2	Unknown	9	8.470	7963593	555641	53.270

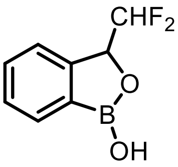

(+)-1
(er 53:47)

Chiral HPLC trace of **(-)-1** (e.r 81:19) (see procedure)

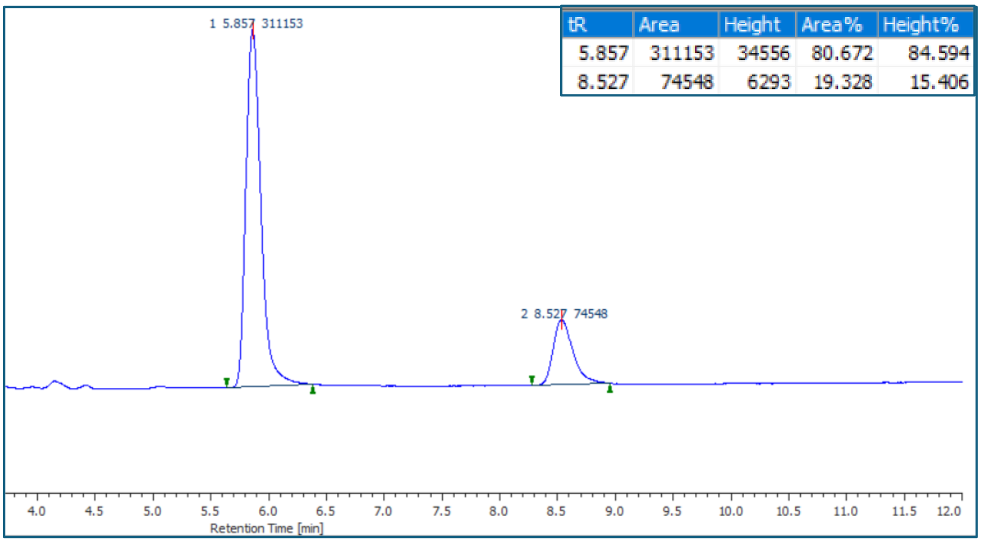

tR	Area	Height	Area%	Height%
5.857	311153	34556	80.672	84.594
8.527	74548	6293	19.328	15.406

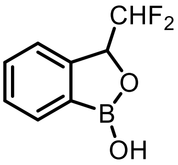

(–)-1
(er 81:19)

Chiral HPLC trace of **(-)-1**

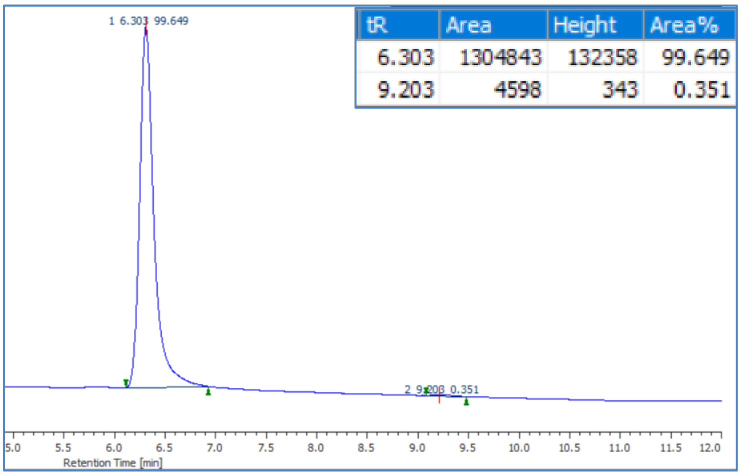

tR	Area	Height	Area%
6.303	1304843	132358	99.649
9.203	4598	343	0.351

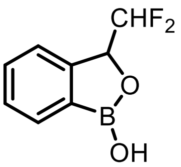

(–)-1

ee 99.3%

$[\alpha]^{22}_{D}$ = -11.8 (c 0.013 M in $CHCl_3$)

Chiral HPLC trace of **(+)-1**

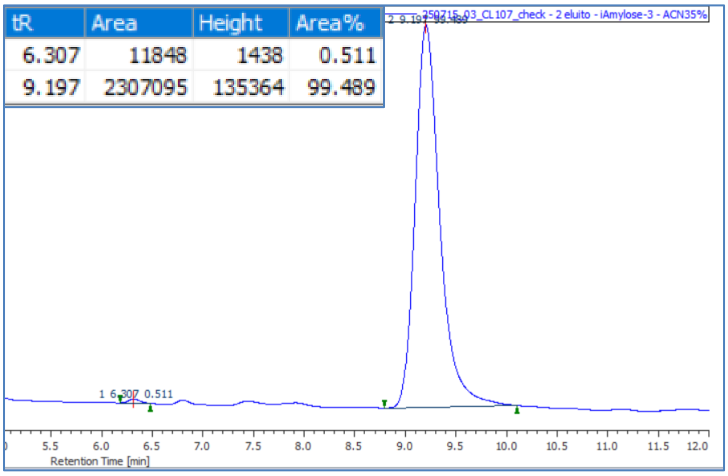

tR	Area	Height	Area%
6.307	11848	1438	0.511
9.197	2307095	135364	99.489

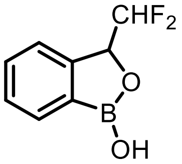

(+)-1

ee 98.9%

$[\alpha]^{22}_{D}$ = +11.1 (c 0.008 M in $CHCl_3$)

5. MINIMAL INHIBITORY CONCENTRATION ASSAYS

Minimal inhibitory concentration assays for benzoxaboroles **1-17b** (see procedure)

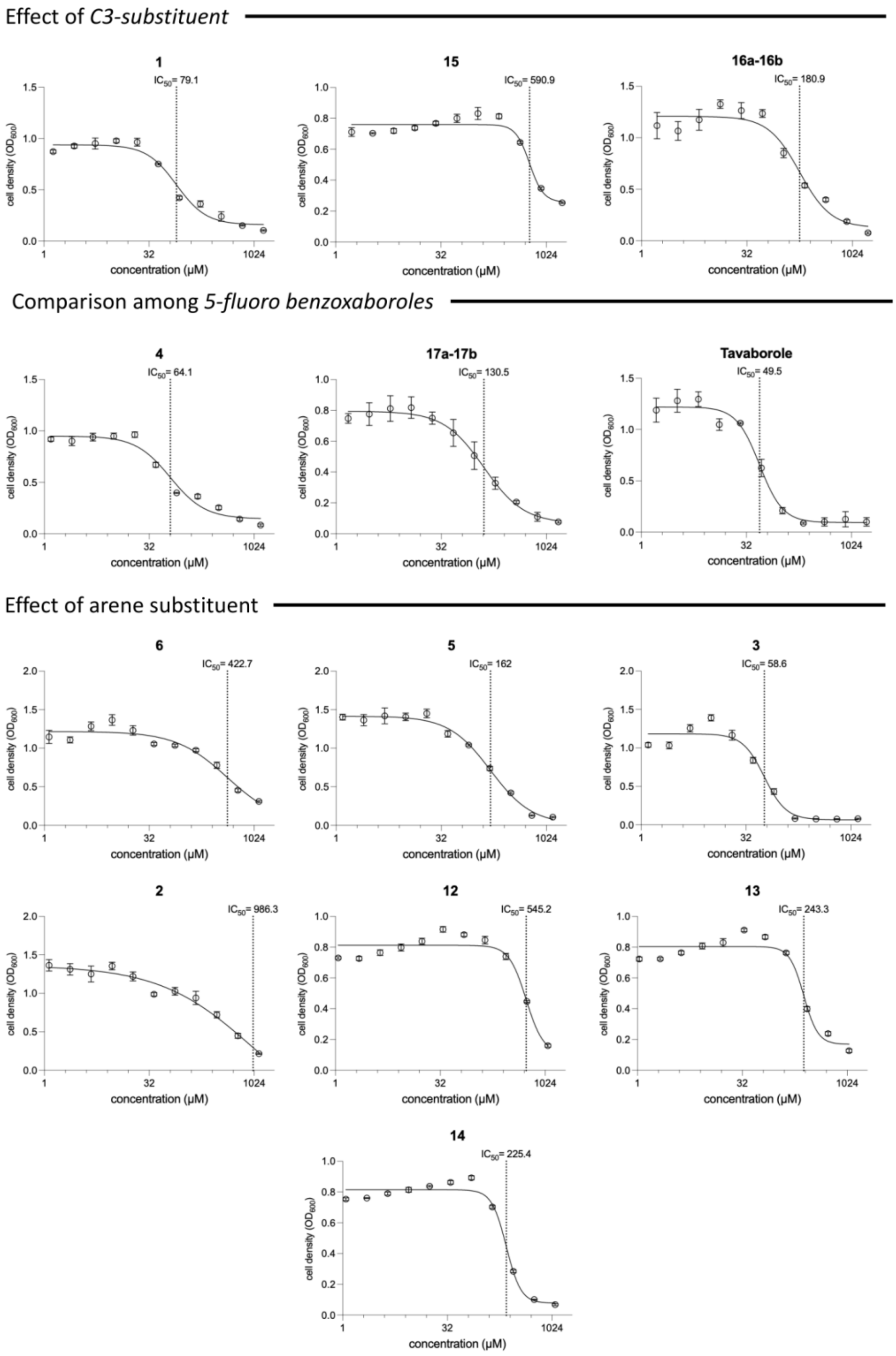

Figure S7: Dose-response curves of the benzoxaboroles against the growth of *Escherichia coli* MG1655. The dose-response curves for each benzoxaborole compound, tested at increasing concentrations against the growth of *Escherichia coli* MG1655, was reported as a function of the bacterial cell density by measuring the OD_{600} after 24 hrs of growth. Briefly, *E. coli* MG1655 was grown at 37°C in static condition in the absence or in the presence of increasing concentrations of the benzoxaboroles. Since all the compounds were dissolved in dimethyl sulfoxide (DMSO), samples

grown in the presence of the same amount of the solvent vehicle were used as controls, without affecting bacterial growth. Results of at least four independent replicates, mean, and standard deviation are shown in dot plots. The half-maximal inhibitory concentrations (IC_{50}) of each compound are reported and indicated as a dash vertical line in each graph. The IC_{50} values were determined by GraphPad Prism, fitting the dose-response data to a nonlinear regression curve with a variable slope (four parameter).

6. BIOFILM FORMATION ASSAYS

Minimal inhibitory concentration assays for benzoxaboroles **1-17b** (see procedure)

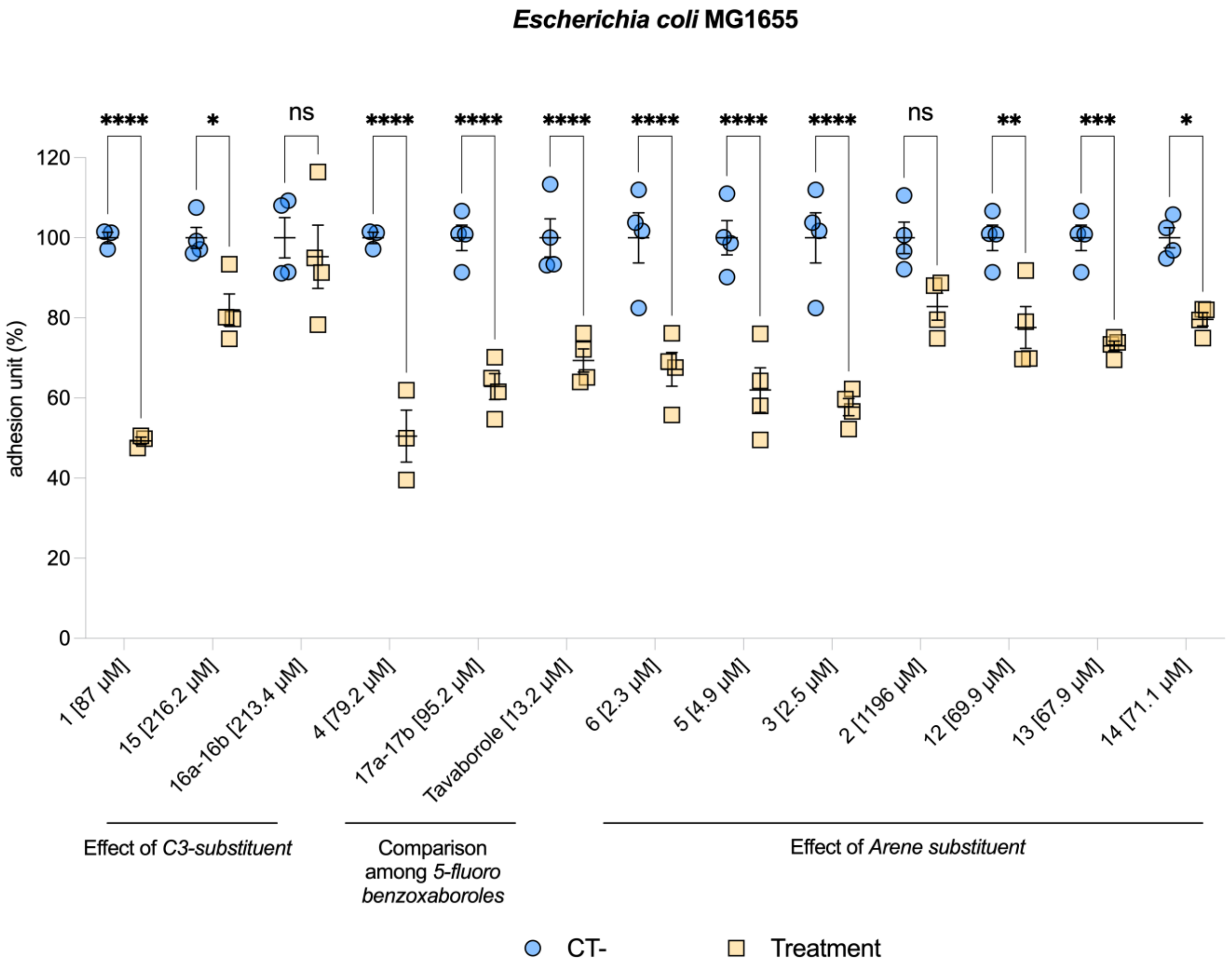

Figure S8: **The anti-biofilm activity of sub-MIC concentrations of benzoxaboroles against *Escherichia coli* MG1655.** Bacterial adhesion assay performed on *Escherichia coli* MG1655 strain grown at 30°C in static condition in the absence (blue circles) or in the presence (yellow squares) of benzoxaboroles tested at different sub-inhibitory concentrations (indicated below the graph). Since all the compounds were dissolved in dimethyl sulfoxide (DMSO), samples grown in the presence of the same amount of the solvent vehicle were used as controls and considered as 100%. Results of at least four independent replicates, mean, and standard deviation are shown in dot plots. ns, not significant; *, p-value < 0.05; ** p-value < 0.005; *** p-value < 0.0005; ****, p-value < 0.0001, two-way analysis of variance (ANOVA) with Šidák's test for multiple comparisons

7. REFERENCES

1. Methods for Dilution Antimicrobial Susceptibility Tests for Bacteria That Grow Aerobically. www.clsi.org.

2. Baldelli, V. *et al.* Identification of FDA-approved antivirulence drugs targeting the Pseudomonas aeruginosa quorum sensing effector protein PqsE. *Virulence* **11**, 652–668 (2020).

3. Tomsho, J. W., Pal, A., Hall, D. G. & Benkovic, S. J. Ring structure and aromatic substituent effects on the pKa of the benzoxaborole pharmacophore. *ACS Med Chem Lett* **3**, 48–52 (2012).

4. Kazmi, M. Z. H., Schneider, O. M. & Hall, D. G. Expanding the Role of Boron in New Drug Chemotypes: Properties, Chemistry, Pharmaceutical Potential of Hemiboronic Naphthoids. *J Med Chem* **66**, 13768–13787 (2023).

5. Jang, M., Lim, T., Park, B. Y. & Han, M. S. Metal-Free, Rapid, and Highly Chemoselective Reduction of Aromatic Nitro Compounds at Room Temperature. *Journal of Organic Chemistry* **87**, 910–919 (2022).

6. Bonardi, A. *et al.* Benzoxaboroles: New Potent Inhibitors of the Carbonic Anhydrases of the Pathogenic Bacterium Vibrio cholerae. *ACS Med Chem Lett* **11**, 2277–2284 (2020).

7. Boivin, J. L. *Can J Chem* **36**, 1405–1409 (1958).

8. Zheng, Y. & Wills, M. *Org Biomol Chem* **20**, 3742–3746 (2022).

9. Fasano, V., Radcliffe, J. E., Curless, L. D. & Ingleson, M. J. N-Methyl-Benzothiazolium Salts as Carbon Lewis Acids for Si−H σ-Bond Activation and Catalytic (De)hydrosilylation. *Chemistry – A European Journal* **23**, 187–193 (2017).

10. Mamiatis, T., Fritsch, E. F., Sambrook, J. & Engel, J. Molecular cloning–A laboratory manual. New York: Cold Spring Harbor Laboratory. 1982, 545 S., 42 $. *Acta Biotechnol* **5**, 104–104 (1985).

11. Merritt, J. H., Kadouri, D. E. & O'Toole, G. A. Growing and Analyzing Static Biofilms. *Curr Protoc Microbiol* **00**, 1B.1.1-1B.1.17 (2006).

12. Ravishankar, S. *et al.* Fluoropyrimidines affect de novo pyrimidine synthesis impairing biofilm formation in Escherichia coli. *Biofilm* **7**, 100180 (2024).
